# Targeted Degradation of Picornaviral 3C Protease via PROTACs Confers High Barrier to Viral Resistance and Broad‐Spectrum Antiviral Activity

**DOI:** 10.1002/advs.76662

**Published:** 2026-07-17

**Authors:** Weilong Deng, Junyu Chen, Yingyue Pang, Yuanyuan Zhang, Siqian Chen, Guoliang You, Xiaoman Tian, Zhongxin Xu, Jing Wang, Luqing Shang

**Affiliations:** ^1^ State Key Laboratory of Medicinal Chemical Biology College of Pharmacy KLMDASR of Tianjin and Drug Discovery Center for Infectious Disease Nankai University Tianjin People's Republic of China

**Keywords:** 3C protease, broad‐spectrum activity, drug‐resistant mutations, picornavirus, PROTAC

## Abstract

Diseases caused by picornaviruses pose a serious threat to society due to their high contagiousness and widespread prevalence. Beyond the poliovirus vaccine, antiviral therapeutics remain unavailable for most picornaviral infections. Moreover, existing inhibitors under development generally exhibit narrow‐spectrum activity and low barriers to resistance owing to the high mutability of RNA viral proteins. To address these issues, this study constructed a proteolysis‐targeting chimera (PROTAC) targeting the 3C protease (3C^Pro^) — a key viral enzyme essential for picornavirus protein processing, host shutoff, and immune suppression. In enterovirus 71 (EV71), a representative member of the picornavirus family, experimental results showed that PROTAC molecule effectively inhibits viral replication through a dual mechanism of action: directly inhibiting the catalytic function of the 3C^Pro^ and inducing its degradation via the ubiquitin‐proteasome pathway. Moreover, this PROTAC molecule not only demonstrated effective degradation of previously reported and AI‐predicted potential drug‐resistant EV71 mutants, but also exhibited degradation activity against 3C^Pro^ from multiple picornaviruses, indicating its potential for broad‐spectrum antiviral activity. By integrating multidisciplinary approaches, this work demonstrates that targeted protein degradation can effectively induce the degradation of 3C^Pro^ in picornaviruses, supporting the development of resistance‐resistant, broad‐spectrum antiviral agents and highlighting their potential to address evolving viral threats.

## Graphical abstract

This study reports **D34** as the first PROTAC degrader that targets the 3C protease of picornaviruses. **D34** effectively degrades EV71 3C protease via the ubiquitin‐proteasome pathway. More importantly, **D34** exhibits a high resistance barrier and shows broad‐spectrum antiviral activity against multiple picornaviruses, highlighting its potential as a novel antiviral agent.

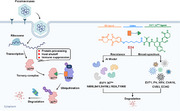

## 1 Introduction

Picornaviruses are a group of non‐enveloped, single‐stranded, positive‐sense RNA viruses characterized by their small size (approximately 20–30 nm in diameter) and icosahedral capsid symmetry [1, 2]. These pathogens pose significant threats to human health: for instance, EV71‐induced hand, foot, and mouth disease (HFMD) exhibits high transmissibility, predominantly affects pediatric populations, and may lead to severe neurological and cardiopulmonary complications. Poliovirus specifically targets motor neurons, resulting in permanent paralysis; coxsackievirus can precipitate fulminant myocarditis; hepatitis A virus causes acute liver failure; while rhinovirus provokes severe respiratory infections in individuals with asthma [3, 4]. Currently, beyond the poliovirus vaccine, specific antiviral therapeutics remain unavailable for most picornaviral infections.

The development of antiviral drugs against picornaviruses primarily focuses on small‐molecule inhibitors. For instance, Rupintrivir (AG7088) demonstrates highly potent inhibitory effects against rhinovirus infection [5]. NK‐1.8k exhibits significant activity against diverse strains of EV71 and enterovirus D68 (EV‐D68) [6, 7]. Despite their efficacy, most small‐molecule inhibitors suffer from a limited spectrum of activity—where a single drug targets only one specific virus—which substantially increases research and development costs and timelines [8]. Furthermore, single‐drug treatments readily induce viral drug resistance, thereby compromising therapeutic outcomes [9]. Consequently, developing novel antiviral agents that integrate both resistance‐resistant and broad‐spectrum properties represents not only an urgent need to counter current viral mutation threats but also a core strategy in establishing future frameworks for infectious disease prevention and control.

Targeted Protein Degradation (TPD) has emerged as a promising strategy for developing resistance‐resistant, broad‐spectrum antiviral agents by harnessing small‐molecule compounds to activate intracellular protein degradation pathways and eliminate pathogenic proteins. The most widely employed degrader molecules are PROTACs. These molecules induce the degradation of target proteins by exploiting the spatial proximity effect to facilitate interaction between the target protein and an E3 ubiquitin ligase. This interaction leads to the ubiquitination modification of the target protein, marking it for degradation [10, 11]. Unlike traditional small‐molecule inhibitors, PROTACs do not require strong binding to the target protein's active site to exert their effect [12, 13]. This “event‐driven” catalytic degradation mechanism allows PROTACs to achieve efficient clearance even with weak binding to the target. This characteristic enables PROTACs to effectively overcome drug resistance; caused by target mutations and significantly enhances their potential for broad‐spectrum drug development. Furthermore, because PROTACs do not rely on continuously occupying the target's active site, they are less likely to induce the emergence of resistance mutations. This reduces the risk of further accumulation of resistance mutations and enables more durable therapeutic effects [14]. Currently, PROTAC technology is extensively applied in the development of broad‐spectrum anti‐cancer drugs, targeting key proteins that are overexpressed or mutated in various cancers (e.g., BCL‐2, BRD4) [15, 16, 17]. However, research on antiviral agents that simultaneously overcome drug resistance and exhibit broad‐spectrum activity remains at an early stage. Notably, PROTACs targeting proteins of HIV, influenza virus, HCV, SARS‐CoV‐2, and flaviviruses have been validated [18, 19, 20, 21, 22, 23, 24]. These successful cases have laid a crucial research foundation for extending PROTAC technology to induce degradation in drug‐resistant, broad‐spectrum picornaviruses.

To enable the design of broad‐spectrum PROTACs, we selected 3C^Pro^ as the target protein. 3C^Pro^ is a cysteine protease with chymotrypsin‐like substrate specificity [25, 26, 27]. It is a key enzyme essential for picornavirus replication, responsible for viral protein processing, host shutoff, and immune suppression. [28, 29, 30]. Consequently, its central role renders 3C^Pro^ an important target for anti‐picornaviruses drug development. Furthermore, the 3C^Pro^ of picornaviruses exhibits a high degree of structural conservation across its core three‐dimensional structure (such as the chymotrypsin‐like β‐barrel fold domain), catalytic mechanism (reliant on a conserved His/Glu/Cys catalytic triad), key substrate recognition sites (such as the S1 pocket), and dimerization interface [31]. Hence, PROTAC strategies targeting this protease hold promise for achieving broad‐spectrum antiviral activity against multiple picornaviruses. Our research group has previously developed a series of potent peptidomimetic inhibitors against EV71 3C^Pro^ [6, 32, 33, 34]. These research findings have laid a solid foundation for the design of PROTACs.

In this article, we successfully design and synthesize the first highly efficient PROTAC molecule, **D34**, targeting picornaviruses 3C^Pro^. This molecule employs a reversible covalent binding strategy, which significantly prolongs the duration of action and enhances degradation efficiency. Mechanistic studies reveal that **D34**‐mediated degradation is dependent on the cellular ubiquitin‐proteasome system. Using the AI model, we predict potential resistance mutation sites of EV71 3C^Pro^ and validate D34's degradation capability against high‐risk mutants and previously reported resistance mutants. Furthermore, **D34** demonstrates potent antiviral efficacy against EV71, with its mechanism of action mediated through dual pathways: direct inhibition of protease activity and targeted protein degradation. Importantly, **D34** demonstrates broad‐spectrum degradation activity against the 3C^Pro^ of multiple picornaviruses, including EV71, Coxsackievirus A16 (CVA16), Coxsackievirus B3 (CVB3), Human Rhinovirus (HRV), Poliovirus (PV), and Echovirus (ECHO). These findings further validate the feasibility of TPD technology for developing antiviral agents with dual capabilities of overcoming drug resistance and exhibiting broad‐spectrum activity. This work not only addresses currently evolving viral threats but also provides a potential strategy for mitigating future pandemics.

## 2 Results and Discussion

### 2.1 Design of 3C^Pro^ PROTAC Degraders

**FIGURE 1:**
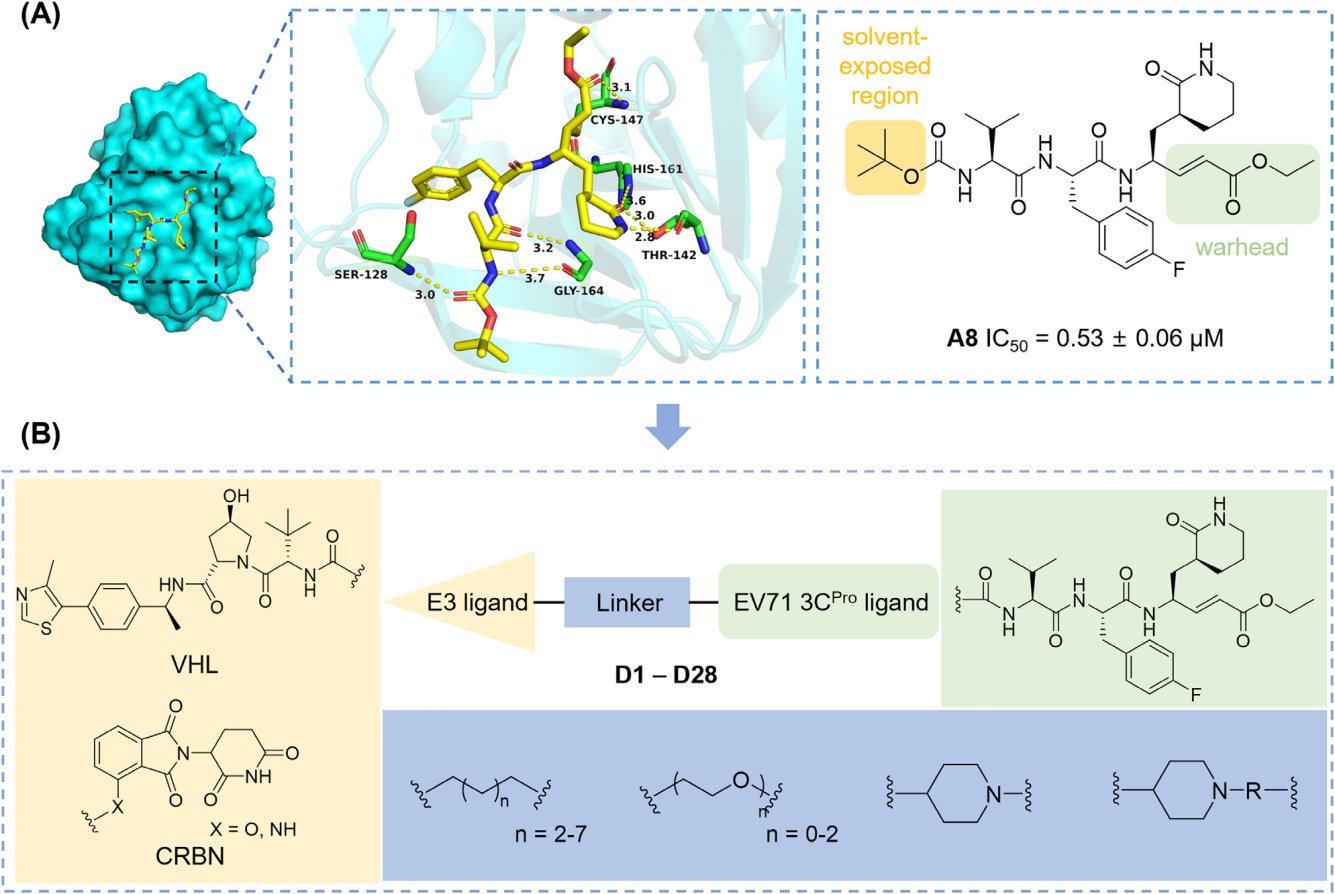
Rational design of EV71 3C^Pro^ PROTACs. (A) The chemical structure and the predicted binding mode of **A8** (colored by atom type: C yellow, O red, N blue, polar H light grey) against EV71 3C^Pro^ (PDB code: 5BPE). Hydrogen bonds are indicated as yellow dashed lines. (B) The schematic diagram of **D1**‐**D28**.

Picornaviruses 3C^Pro^ is a cysteine protease with a catalytic triad of His40‐Glu71‐Cys147 at its active site [35]. At present, the effective strategy for developing 3C^Pro^ inhibitors is to convert the peptide bonds within the substrate into electrophilic warheads (such as aldehydes, Michael receptors) to promote the formation of covalent adducts with the catalytic Cys147 of 3C^Pro^. Previously, our group developed a series of effective EV71 3C^Pro^ inhibitors, among which compound **A8** demonstrated strong inhibitory activity (IC_50_ = 0.53 ± 0.06 µM) (Figure 1A). The molecular docking results indicate that the covalent interaction between **A8** and 3C^Pro^ contributes to their high affinity binding. In addition, there are multiple hydrogen bonds between **A8** and 3C^Pro^. It is worth noting that in the docking model, the *N*‐terminal *tert*‐butoxycarbonyl group of **A8** is not well‐defined in the structure, indicating an unfitting size or relatively loosely bound pattern within the P4‐binding pocket (Figure 1A). These results guide the design of a series of 3C^Pro^ degradation agents and provide a basis for the rational introduction of linker chains (Figure 1B). Subsequently, we designed and synthesized a series of bifunctional small molecule PROTACs, namely **D1**‐**D6** based on VHL ligand and **D7**‐**D28** based on the CRBN ligand (Table 1). During the synthesis, the yields of compounds with different linkers varied. Alkyl chain linkers generally afforded the target products in moderate to good yields in the final amide condensation step, whereas rigid linkers gave relatively lower yields. Furthermore, rigid linkers require multi‐step synthesis, which makes their synthesis more challenging.

**TABLE 1:** The chemical structures of **D1**‐**D28**.

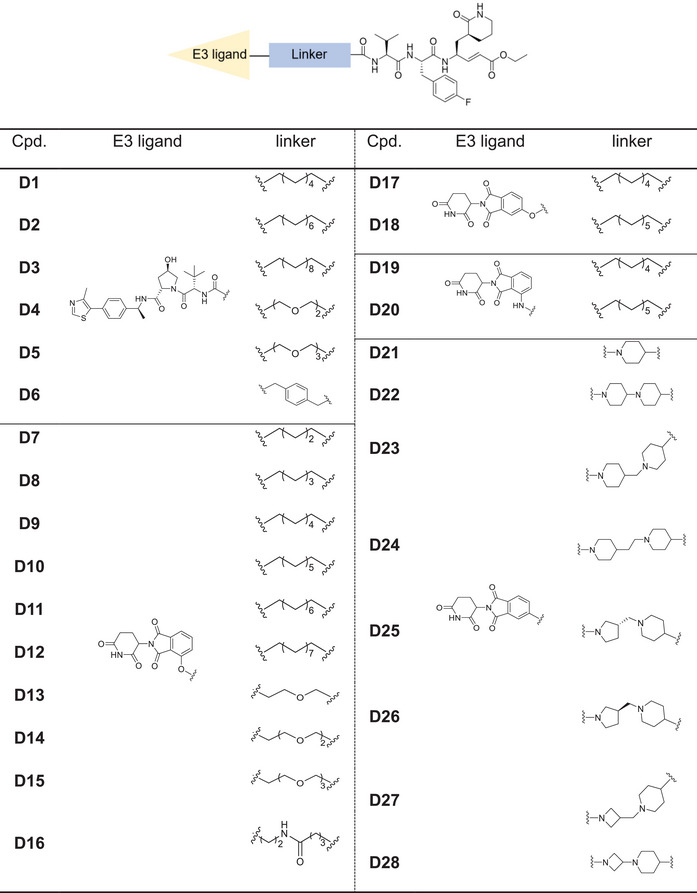

### 2.2 Irreversible Covalent PROTACs

**FIGURE 2:**
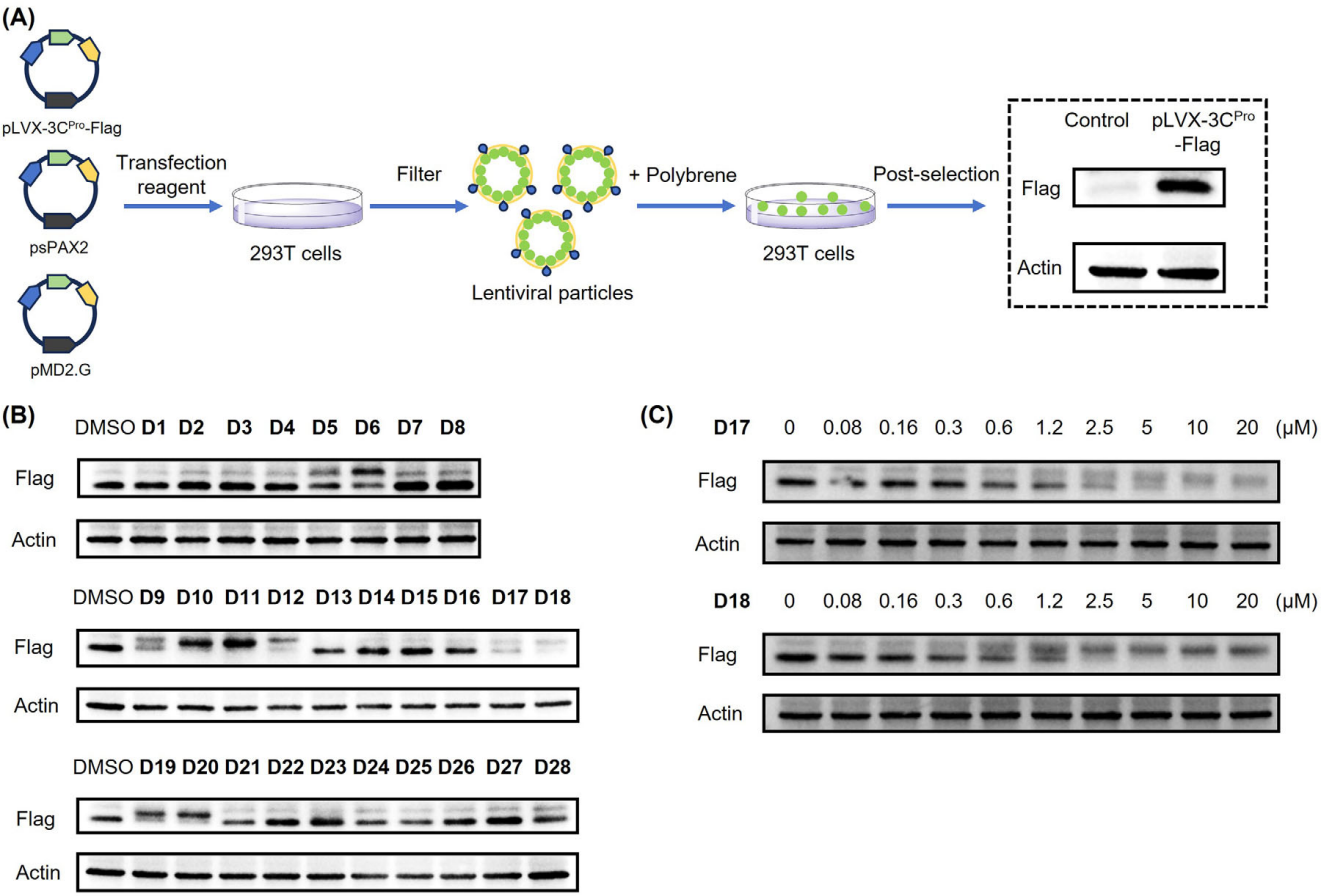
Evaluation of degradation activity for irreversible covalent PROTACs. (A) Schematic workflow for developing a stable HEK293T cell line expressing 3C^Pro^‐Flag fusion protein. (B) The potency of **D1**‐**D28** in degrading 3C^Pro^ was evaluated in the HEK293T cells stably expressing 3C^Pro^‐Flag by Western blotting after the cells were treated with 2 µM PROTACs for 24 h. Representative immunoblots are shown, and β‐actin was used as a control in all immunoblot analyses. (C) The potency of **D17** and **D18** in degrading 3C^Pro^ was evaluated in the HEK293T cells stably expressing 3C^Pro^‐Flag by Western blotting after the cells were treated with different concentrations for 24 h.

To evaluate the degradation capability of PROTACs **D1**‐**D28** against the EV71 3C^Pro^, we developed a stable cell line expressing the target protein. This system was established by constructing a lentiviral vector expressing the 3C^Pro^‐Flag fusion protein, ultimately generating HEK293T cells stably expressing 3C^Pro^‐Flag (Figure 2A). Treatment with 2 µM PROTACs for 24 h was performed on HEK293T cells stably expressing 3C^Pro^‐Flag, followed by protein extraction and Western blot analysis of 3C^Pro^ levels (Figure 2B). The results showed that PROTACs **D1**‐**D6**, designed with the VHL ligand, exhibited no degradation activity, which may be attributed to the bulky VHL ligand or inappropriate linker length, hindering the formation of the ternary complex. In contrast, **D17** and **D18**, designed with the CRBN ligand, demonstrated significant degradation activity, achieving near‐complete degradation of 3C^Pro^ at 2 µM. Further concentration‐dependent analysis revealed that both compounds had DC_50_ values below 1 µM. Based on structure‐activity relationship analysis, we speculate that the optimal linker length may be 6–7 carbon atoms. To assess their long‐term degradation efficacy, we examined the time‐dependent degradation of **D17** (Figure S1). The data showed that the degradation efficiency of 3C^Pro^ significantly decreased after 36 h and was almost lost by 48 h. We hypothesize that this phenomenon may be attributed to the irreversible covalent warhead property of the Michael acceptor: its irreversible binding could prevent the dissociation of PROTACs from the complex after target protein degradation, thereby compromising their recyclability [36, 37]. This finding suggests that developing reversible or non‐covalent warhead structures may be a critical strategy to sustain the persistent degradation capability of PROTACs.

### 2.3 Reversible Covalent PROTACs

**FIGURE 3:**
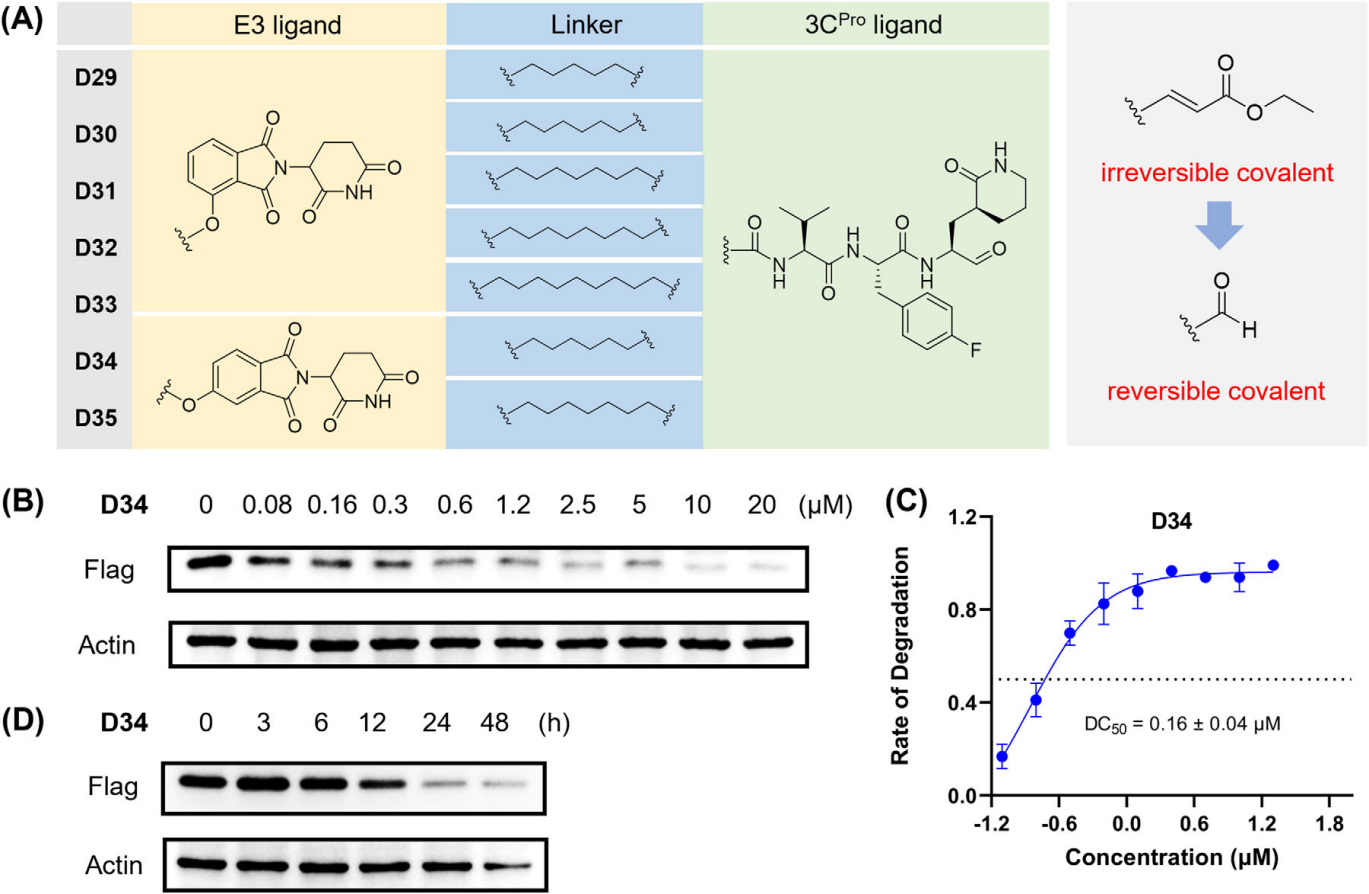
Evaluation of degradation activity for reversible covalent PROTACs. (A) The chemical structures of **D29**‐**D35**. (B) The potency of **D34** in degrading 3C^Pro^ was evaluated in the HEK293T cells stably expressing 3C^Pro^‐Flag by Western blotting after the cells were treated with different concentrations for 24 h. (C) DC_50_ fitting curves for **D34**. Quantitative results, summarized from three replicates, are expressed as mean ± SD. (D) The time course of **D34**‐mediated 3C^Pro^ degradation was evaluated in the HEK293T cells stably expressing 3C^Pro^‐Flag by Western blotting after the cells were treated with 2 µM **D34** for various time points.

The aldehyde group, as a reversible covalent warhead, is widely used in the design of cysteine protease peptidomimetic inhibitors due to its unique reversible binding properties [38]. Based on this, we designed and synthesized seven novel PROTAC compounds named **D29**‐**D35** by replacing the irreversible covalent Michael acceptor with the aldehyde group (Figure 3A). Among them, **D34** exhibited the best degradation efficacy, with a DC_50_ value as low as 0.16 µM (Figure 3B,C and Figure S2). In the HEK293T cells stably expressing 3C^Pro^‐Flag, **D34** was able to induce rapid and sustained degradation of the 3C^Pro^. Time‐dependent degradation experiments showed that significant degradation effects could be observed after 12 h of treatment with 2 µM **D34**, and near‐complete degradation was achieved after 24 h (Figure 3D). Notably, the compound maintained stable degradation activity at the 36‐ and 48‐h time points, with no significant decrease in degradation efficiency (Figure S3). These results indicate that reversible covalent PROTACs significantly outperform irreversible covalent PROTACs in terms of degradation efficacy and long‐term stability.

### 2.4 The Degradation of 3C^Pro^ Mediated by **D34** Depends on the Formation of Covalent Bond

**FIGURE 4:**
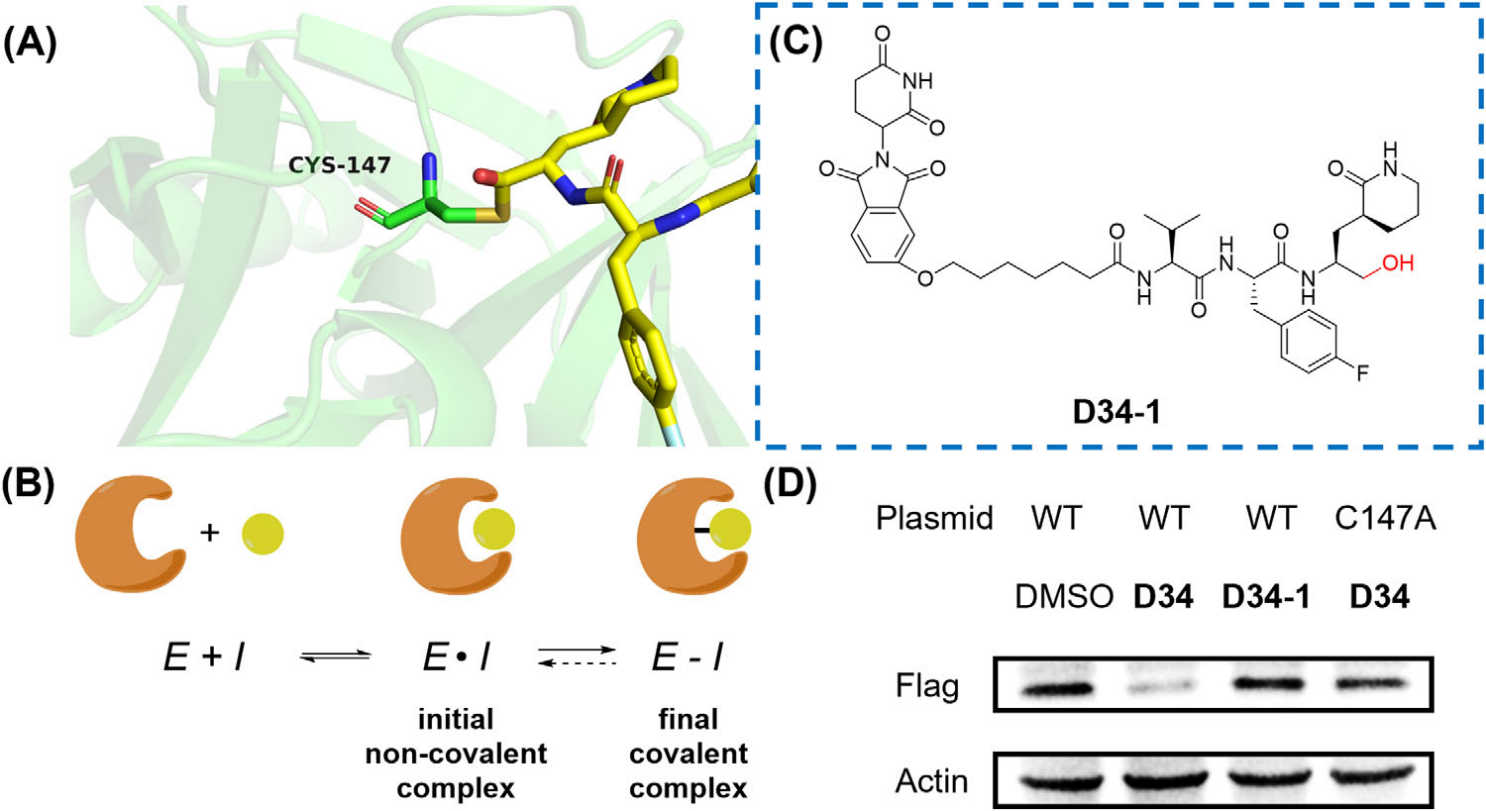
The degradation of 3C^Pro^ mediated by **D34** depends on the formation of covalent bond. (A) The predicted binding mode of **D34** (colored by atom type: C yellow, O red, N blue, polar H light grey) against EV71 3C^Pro^ (PDB code: 5BPE). The aldehyde group of **D34** forms a reversible covalent bond with Cys147. (B) The mechanism of action of covalent inhibitors. (C) The chemical structure of **D34‐1**. (D) The potency of **D34** and **D34‐1** in degrading 3C^Pro^ was evaluated in the HEK293T cells stably expressing 3C^Pro^‐Flag or 3C^Pro^(C147A)‐Flag by Western blotting after the cells were treated with different PROTACs for 24 h.

**D34**, as a covalent PROTAC, forms a reversible covalent bond with the Cys147 residue of the 3C^Pro^ (Figure 4A). Regarding the reversibility of **D34**, we verified it through dilution experiments (Figure S4). The binding process of covalent inhibitors typically involves two key steps: first, the inhibitor binds to the specific binding pocket of the target protein through an electrophilic group, bringing the active warhead close to the active site; subsequently, the active warhead undergoes a chemical reaction with the active site residue of the target protein, forming an irreversible or reversible covalent bond (Figure 4B) [39]. To further investigate the impact of covalent bond formation on protein degradation, we chemically reduced the aldehyde group of **D34** to a hydroxyl group, yielding the derivative **D34‐1** (Figure 4C). The experimental results showed that this derivative failed to induce the degradation of the 3C^Pro^ (Figure 4D). Additionally, we established a HEK293T cell line stably expressing the C147A mutant protein and found that **D34** was also unable to induce the degradation of this mutant protein (Figure 4D). These experimental results collectively confirm that the **D34**‐mediated degradation of the 3C^Pro^ is strictly dependent on the formation of covalent bonds.

### 2.5 Mechanism of 3C^Pro^ Degradation by **D34**

**FIGURE 5:**
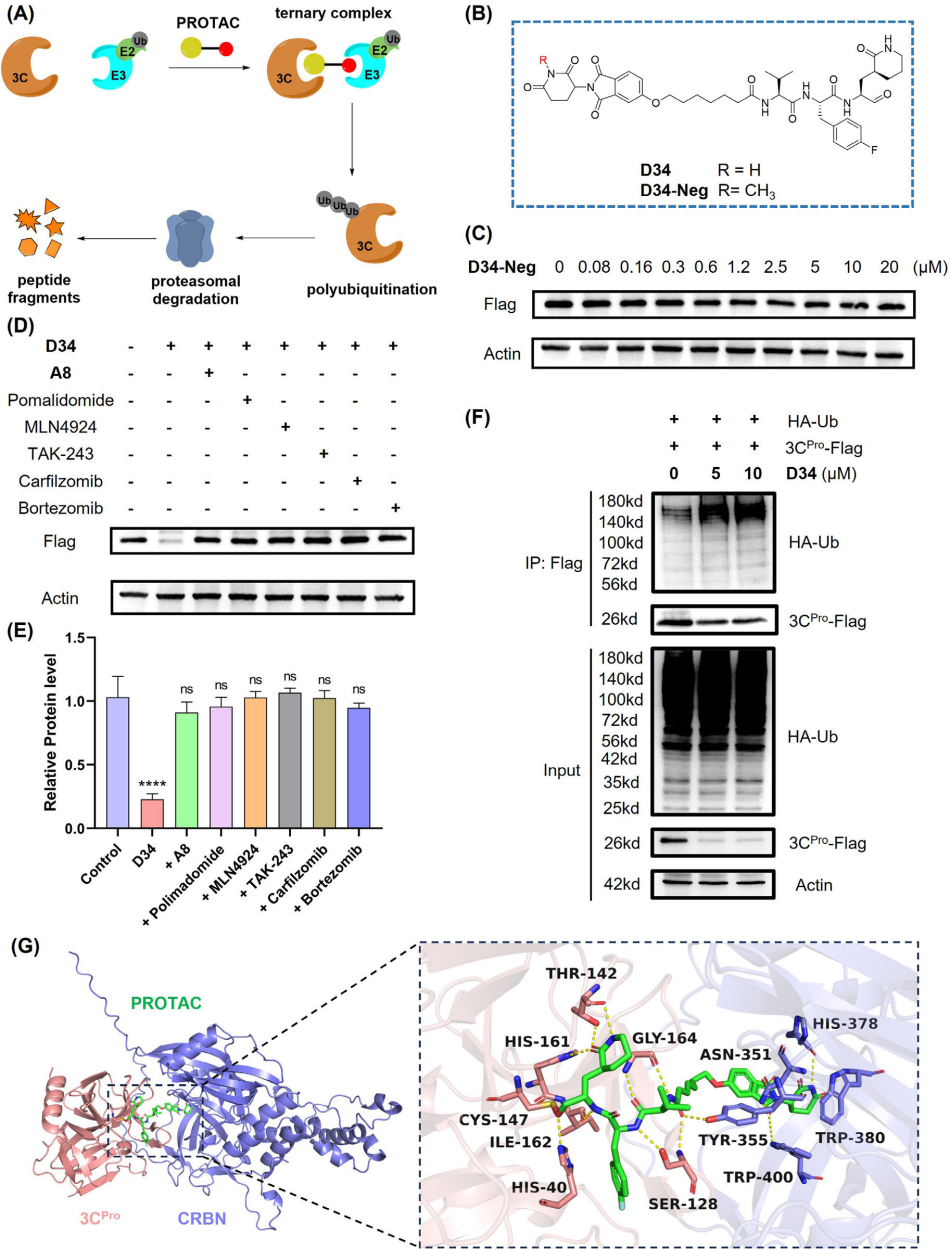
Degradation mechanism validation of **D34**. (A) Degradation mechanism of PROTAC mediated ubiquitin proteasome system. (B) The chemical structure of **D34** and **D34‐Neg**. (C) The potency of **D34‐Neg** in degrading 3C^Pro^ was evaluated in the HEK293T cells stably expressing 3C^Pro^‐Flag by Western blotting after the cells were treated with different concentrations for 24 h. (D) Prior to treatment with **D34** (2 µM) for 24 h, HEK293T cells stably expressing 3C^Pro^‐Flag were pre‐treated with DMSO, **A8** (10 µM), pomalidomide (10 µM), MLN4924 (5 µM), TAK‐243 (5 µM), Carfilzomib (5 µM), or Bortezomib (5 µM) for 4 h. The levels of the 3C^Pro^ were assessed via Western blot analysis. (E) Analysis of 3C^Pro^ levels. Quantitative results, summarized from three replicates, are expressed as mean ± SD, with significance denoted as ^*^ *p* < 0.05, ^**^ *p* < 0.01, ^***^ *p* < 0.001, ^****^ *p* < 0.0001 (Student's t‐test). (F) The HEK293T cells stably expressing 3C^Pro^‐Flag were first treated with **D34** (5 or 10 µM) for 12 h, followed by a combination of **D34** (5 or 10 µM) and MG132 (5 µM) treatment for 12 h. Whole protein lysates were used for the Co‐IP assay. (G) Diagram of 3C^Pro^‐**D34**‐CRBN complex binding pattern: CRBN (blue), 3C^Pro^ (pink) and **D34** (green stick). Hydrogen bonds are indicated as yellow dashed lines.

The ubiquitin‐proteasome system (UPS) serves as the central machinery for protein degradation in eukaryotic cells, where target proteins are tagged with ubiquitin and subsequently degraded by the proteasome (Figure 5A) [40]. To elucidate the critical role of CRBN binding in degradation activity, we designed and synthesized the negative control compound **D34‐Neg**, which lost its ability to bind CRBN due to the introduction of a methyl capping group (Figure 5B). Activity assays revealed that both **D34** and **D34‐Neg** exhibited comparable inhibitory activity against 3C^Pro^ with IC_50_ values of 0.57 and 0.66 µM, respectively (Figure S5). However, **D34‐Neg** failed to induce the degradation of 3C^Pro^ in the HEK293T cells stably expressing 3C^Pro^‐Flag (Figure 5C). This finding confirmed that the degradation depends on its binding to CRBN. To further validate the necessity of 3C^Pro^ and CRBN binding for degradation, we conducted competitive inhibition experiments. Pretreatment of cells with either **A8** (an inhibitor of 3C^Pro^) or pomalidomide (a CRBN ligand) completely blocked **D34**‐mediated degradation of 3C^Pro^ (Figure 5D,E). These results further demonstrated that **D34**‐induced degradation of 3C^Pro^ relies on the binding with 3C^Pro^ and CRBN. To investigate the molecular mechanism of the degradation pathway, we evaluated the effects of inhibitors targeting key components of the UPS on degradation. The experiments showed that MLN4924 (an inhibitor of NEDD8‐activating enzyme), TAK‐243 (an inhibitor of ubiquitin‐activating enzyme E1), and proteasome inhibitors carfilzomib and bortezomib significantly inhibited the degradation of 3C^Pro^ (Figure 5D,E). Additionally, in vivo ubiquitination assays revealed a significant increase in ubiquitination levels in cells treated with **D34** (Figure 5F), confirming that **D34** achieves specific degradation of 3C^Pro^ by activating the ubiquitin‐proteasome system.

Structural modeling of the 3C^Pro^‐**D34**‐CRBN ternary complex was performed using AlphaFold3, with binding free energy calculations conducted via the PRODIGY tool. The data revealed a direct binding energy of −9.4 kcal/mol between the target protein and CRBN, whereas the PROTAC‐mediated ternary complex exhibited a significantly reduced binding free energy of −13.11 kcal/mol (Table S1). These results demonstrate that **D34** enhances cooperative binding between CRBN and the target protein, thereby facilitating ternary complex formation and stabilization. Moreover, structural analysis identified a covalent bond formation between the aldehyde group of **D34** and Cys147 of 3C^Pro^, while multiple hydrogen bonds were observed between **D34** and key residues (His40, Ser128, Thr142, His161, Ile162, Gly164 in 3C^Pro^ and Asn351, Tyr355, His378, Trp380, Trp400 in CRBN) (Figure 5G). These interactions collectively stabilize the ternary complex conformation.

### 2.6 **D34** can Effectively Degrade the EV71 3C^Pro^‐Resistant Mutations

**FIGURE 6:**
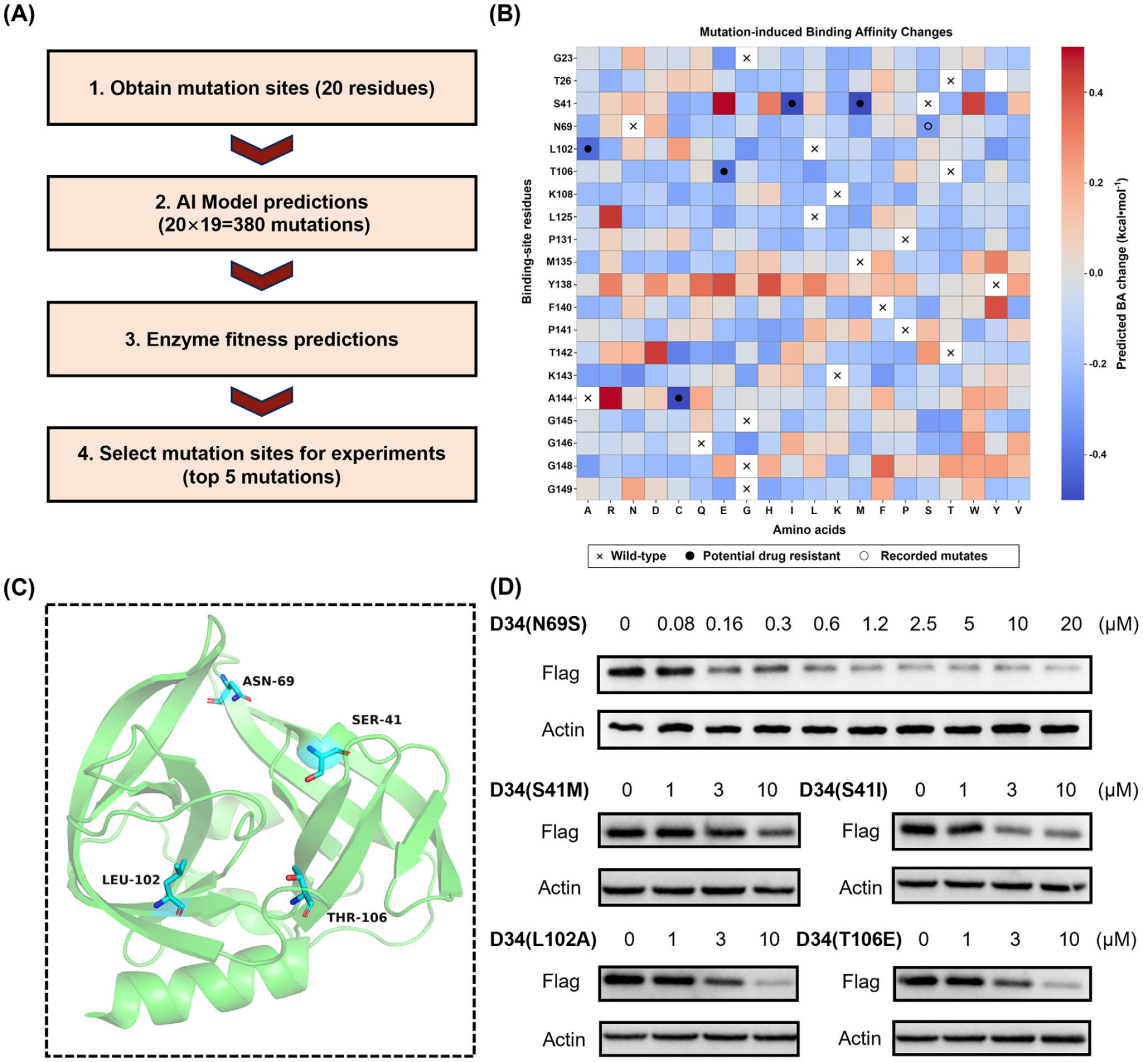
**D34** can effectively degrade the 3C^Pro^‐resistant mutations. (A) A flow chart for predicting drug resistance mutations in EV71 3C^Pro^. (B) A heat map showing the predicted BA changes for the single‐point mutations (x‐axis) at 19 binding‐site residues (y‐axis). Red and blue colors indicate increased and decreased BAs, respectively. X denotes the WT residues; circles identify the reported mutations, and filled circles indicate natural mutations predicted to be potentially drug‐resistant. (C) Locations of predicted drug‐resistance mutation sites in the EV71 3C^Pro^ structure (PDB code: 5BPE). (D) The potency of **D34** in degrading 3C^Pro^ mutations were evaluated in the HEK293T cells stably expressing 3C^Pro^ mutations by Western blotting after the cells were treated with different concentrations for 24 h.

Current research on drug resistance mutations in EV71 3C^Pro^ remains limited, with the N69S mutation against inhibitor NK‐1.8k representing the most well‐characterized case to date [6]. This mutation reduces inhibitor binding affinity by destabilizing the S2 pocket. Traditional approaches for identifying resistance mutation sites—relying on empirical structural biology and point‐to‐point experimental validation—are characterized by prolonged timelines, high costs, and significant temporal lag. These limitations become particularly pronounced when addressing rapidly evolving viruses with high mutational diversity. AI models offer a promising alternative for predicting resistance mutations. CAPTURE is an innovative framework designed to predict viral drug resistance mutations based on the binding affinity (BA) between target and ligand, and it performed well in predicting resistance mutations in SARS‐CoV‐2 3CL^Pro^ [41]. In this study, we extend the CAPTURE framework to predict drug resistance mutations in EV71 3C^Pro^ (Figure 6A). To achieve this, we fine‐tuned a publicly available pretrained model using picornavirus‐like supercluster 3C^Pro^–ligand complexes, in order to get a final model more closely aligned with our specific prediction goals. For mutation predictions, we referred to the crystal structure of the EV71 3C^Pro^‐ligand complex (PDB code: 5BPE) that we previously reported, as the ligand in this structure closely resembles the POI ligand part of our designed PROTAC. We identified 20 potential mutation sites (G23, T26, S41, N69, L102, T106, K108, L125, P131, M135, Y138, F140, P141, T142, K143, A144, G145, Q146, G148, G149) based on the distance between the ligand and the binding pocket. Notably, catalytic sites were excluded from the mutation site selection, as mutations in these regions could significantly disrupt enzyme function. Each selected site was mutated with 19 amino acids, and PyRosetta was employed to generate single‐point mutant protein structures. We then applied the fine‐tuned model to predict the binding affinity of the mutant complexes and calculated the ΔBA values. Finally, we used the ESM tool to assess the protein fitness of the mutant proteins. By combining ΔBA changes and fitness data, we selected the top 5 drug resistance mutation sites for experimental validation of **D34** degradation activity against the resistant mutant proteins.

The prediction results revealed that the previously reported N69S mutation was ranked highly, indicating the predictive accuracy of the model (Figure 6B and Table S2). Initially, the activity of the lead compound **A8** against the N69S mutant protein was tested, demonstrating an approximately 14‐fold reduction in activity (Figure S6). Subsequently, the top five ranked mutations were selected as potential resistance mutations, and the degradation activity of compound **D34** against N69S and these potential resistance mutants was evaluated (Figure 6C and Table S2). The results demonstrate that **D34** exhibits varying degrees of degradation activity against the N69S, S41M, S41I, L102A, and T106E mutants, with the most potent degradation observed for the N69S mutant (Figure 6D). These findings indicate that PROTAC‐induced degradation of viral proteases may represent a potential strategy for overcoming resistance caused by mutations in viral proteins. Furthermore, to evaluate the resistance barrier of **D34**, we conducted a long‐term resistance induction experiment: the virus was serially passaged under escalating drug pressure, and the mutations in the 3C protease of the viral strain passaged under continuous **D34** pressure were analyzed by genetic sequencing. Three amino acid substitutions were identified: I15V, V157I, and H161A. Subsequently, we determined the EC_50_ value of **D34** against this viral strain (Figure S7). The results showed that although the antiviral activity of **D34** against the serially passaged strain was reduced, the magnitude of reduction was limited, and **D34** still maintained good antiviral efficacy against this strain. This indicates that the virus cannot readily escape the antiviral effect of **D34** through mutations, reflecting that **D34** possesses a certain resistance barrier.

### 2.7 Selectivity Investigation of **D34**

**FIGURE 7:**
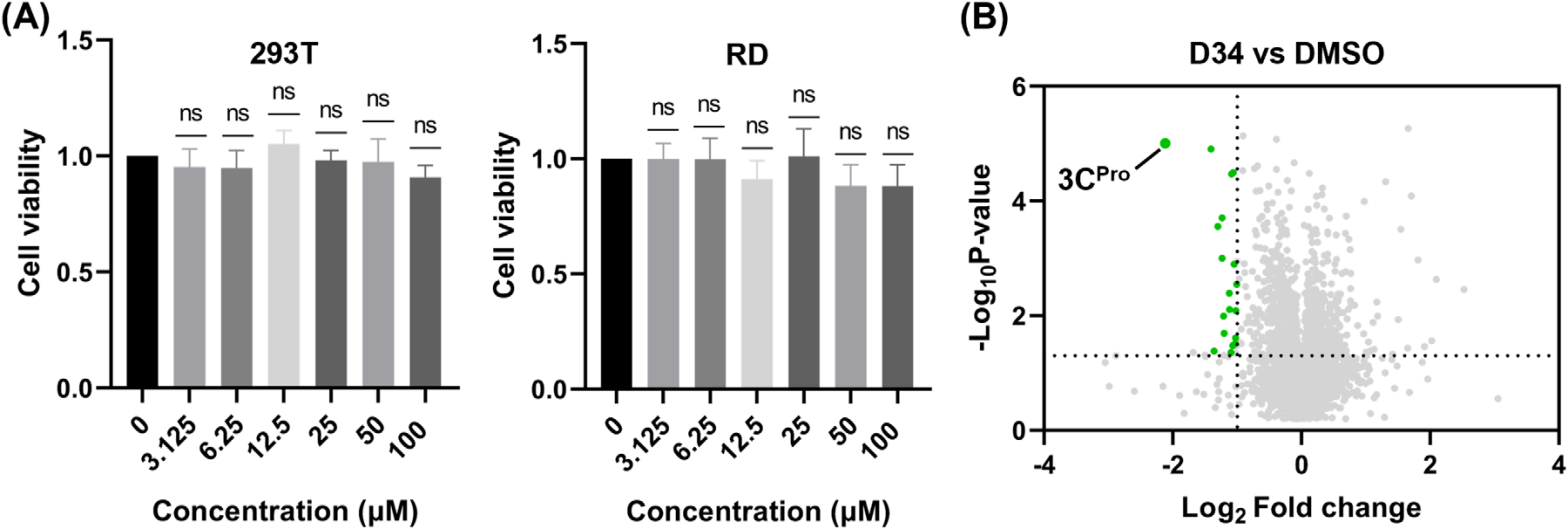
**D34** demonstrates high selectivity for EV71 3C^Pro^. (A) HEK293T and RD cells were treated with **D34** at varying concentrations for 24 h, and cell viability was assessed via cell counting assays. The inhibition percentage was calculated based on DMSO‐treated control wells. Quantitative results, summarized from three replicates, are expressed as mean ± SD, with statistical significance assessed by Student's t‐test. (B) The volcano plot illustrates the changes in protein abundance in HEK293T cells stably expressing 3C^Pro^‐Flag, following treatment with DMSO or **D34** for 24 h, as quantified by DIA‐based proteomics. Green circles represent proteins with decreased content. Quantitative results summarized from triplicate repeats are expressed as mean ± SD.

Covalent chemistry has a potential safety problem due to the high reactivity of warhead structures. This study investigated the cellular state of HEK293T and RD cells treated with **D34** at various concentrations for 24 h and found no significant reduction in cell number, indicating that **D34** exhibits no obvious cytotoxicity within the concentration range of 100 µM (Figure 7A). Compared to the normal control group, **D34** treatment for 24 h in HEK293T cells stably expressing 3C^Pro^ induced a series of protein expression changes (Figure 7B). Analysis revealed that among the differentially expressed proteins, the expression level of 3C^Pro^ was significantly downregulated. These results demonstrate that **D34** can exert highly selective effects on target proteins at the proteome level.

### 2.8 Evaluation of Antiviral Activity Against EV71

**FIGURE 8:**
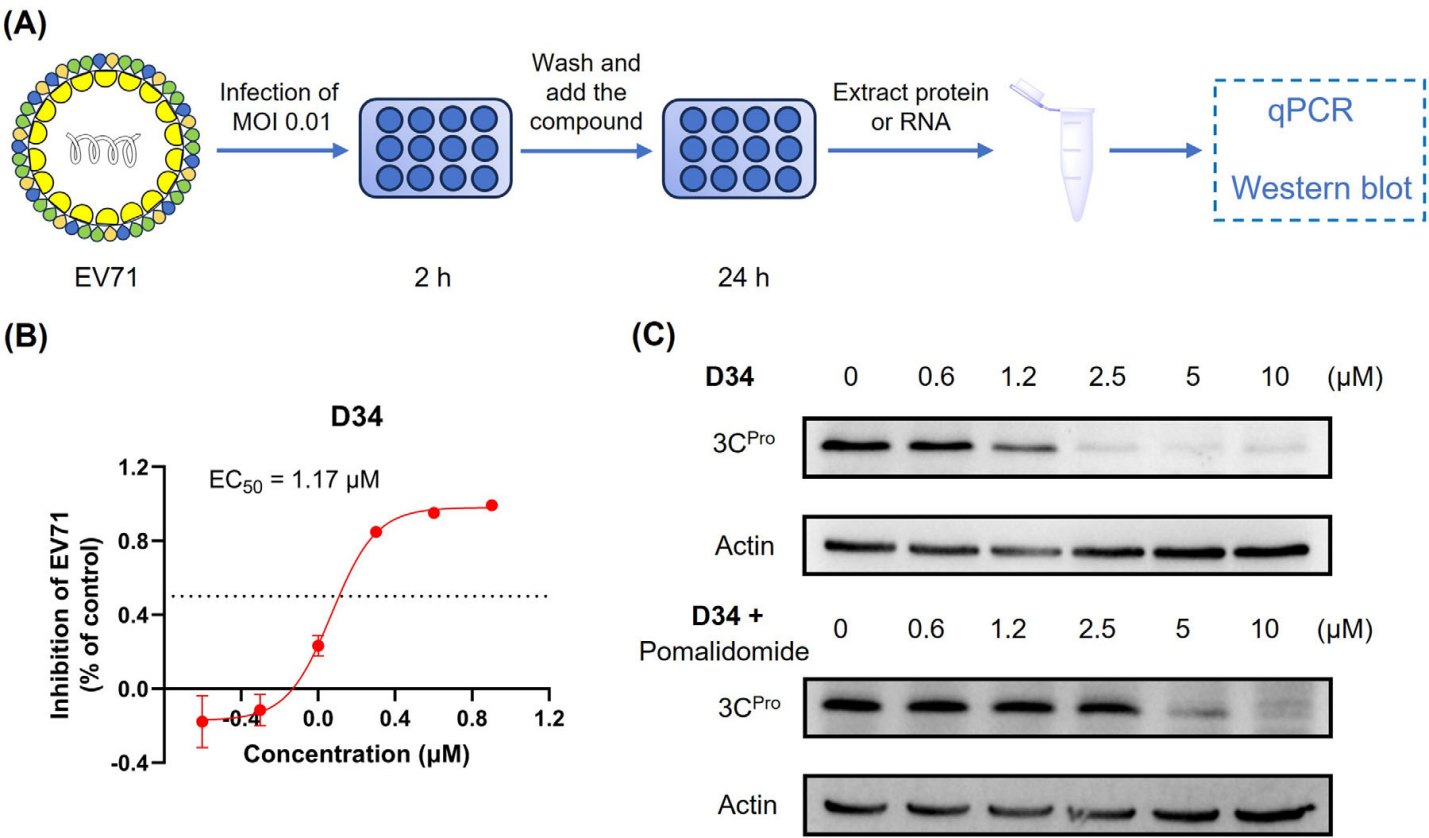
Antiviral effectiveness of **D34**. (A) Workflow for EC_50_ determination and measurement of 3C^Pro^ levels after viral infection. (B) EC_50_ fitting curves for **D34**. Quantitative results summarized from triplicate repeats are expressed as mean ± SD. (C) Prior to treatment with **D34** for 24 h, RD cells infected by EV71 were pre‐treated with DMSO or pomalidomide (10 µM) for 4 h. The levels of the 3C^Pro^ were assessed via Western blot analysis.

After successfully validating the degradation of 3C^Pro^ and understanding the degradation mechanism induced by PROTACs, we continued to evaluate the antiviral efficacy of **D34** in RD cells infected with EV71 virus (Figure 8A). Cells were treated with varying concentrations of **D34** and incubated for 24 h. Subsequently, EV71 mRNA levels were quantified using qPCR to determine the antiviral EC_50_ value. **D34** demonstrated promising antiviral activity (EC_50_  = 1.17 µM) (Figure 8B). Furthermore, we investigated the effect of **D34** treatment on the accumulation of 3C^Pro^ during EV71 infection. RD cells were infected with EV71 and then treated with different concentrations of **D34**. After 24 h, cell lysates were prepared, and protein levels were quantified by Western blot. The 3C^Pro^ protein level decreased in a dose‐dependent manner with increasing **D34** concentration. Notably, pre‐treatment of cells with the CRBN ligand pomalidomide attenuated the antiviral effect of **D34** (Figure 8C). These experiments indicate that the antiviral efficacy of **D34** results from the combined action of both inhibition and degradation mechanisms.

### 2.9 Broad‐Spectrum Degradation of Picornaviral 3C^Pro^ by **D34**

**FIGURE 9:**
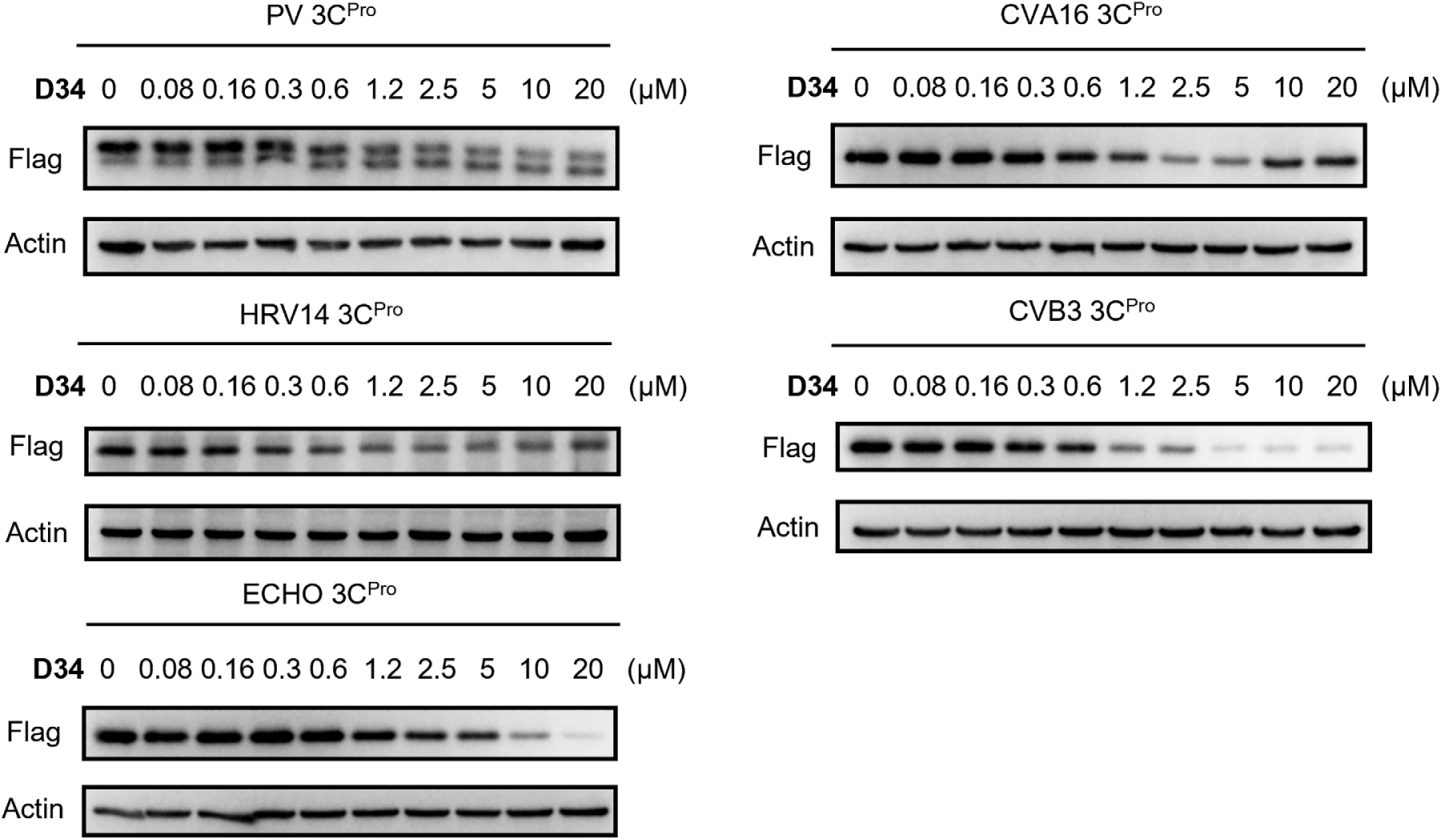
Broad‐spectrum degradation of picornaviral 3C^Pro^ by **D34**. The potency of **D34** in degrading 3C^Pro^ were evaluated in the HEK293T cells stably expressing picornaviruses 3C proteases by Western blotting after the cells were treated with different concentrations for 24 h.

The 3C^Pro^ of different picornaviruses still exhibits relatively high sequence and structural similarity. Sequence alignment reveals that most picornaviral 3C^Pro^ enzymes contain the conserved His40‐Glu71‐Cys147 catalytic triad, with considerable overall sequence homology. Moreover, superimposition of the crystal structures of 3C^Pro^ from various viruses shows that they all adopt a typical chymotrypsin‐like fold, with the core structure composed of two antiparallel β‐barrels. This high degree of conservation at both the sequence and structural levels provides an important structural basis for the broad‐spectrum antiviral activity of the **D34** degrader (Figure S8). Building upon the demonstrated potent degradation activity of **D34** against the EV71 3C^Pro^, this study extended its evaluation to other picornaviruses, including, CVA16, CVB3, HRV, PV, and ECHO, to validate the broad‐spectrum applicability of this degrader against this conserved target. The experimental results showed that **D34** induced significant degradation of 3C^Pro^ across multiple picornavirus species (Figure 9), albeit with varying efficiencies, among which the most pronounced degradation was observed for EV71. This discrepancy might be associated with the ease of **D34**‐induced formation of the **D34**‐3C^Pro^‐CRBN ternary complex. To investigate this, we modeled the ternary complexes formed by the 3C^Pro^ of different picornaviruses with **D34** and CRBN using AlphaFold3, and calculated the binding free energies among the components with the PRODIGY tool. As shown in Table S3, the EV71 ternary complex exhibited the lowest binding free energy, indicating that it is most prone to form a stable complex, which is consistent with its optimal degradation efficacy. Despite the varying degradation efficiencies, **D34** still exhibits broad‐spectrum degradation activity targeting the picornaviral 3C proteases. To further validate its broad‐spectrum antiviral activity, we tested the antiviral activity of **D34** against multiple picornaviruses (Table 2). The results showed that **D34** exhibited good activity against all tested viruses, indicating that **D34** possesses broad‐spectrum antiviral effect.

**TABLE 2:** Antiviral activity of **D34** against other picornaviruses.

Picornaviruses	EC_50_ (µM)^a^
HRV	0.83
CVA16	0.93
CVA10	0.04
CVA6	0.76
CVB5	0.28
ECHO11	2.35

^a^ Each compound was tested in triplicate, and data are presented as mean.

### 2.10 In Vivo Pharmacokinetic Evaluation of **D34**

We evaluated the pharmacokinetic profile of **D34** in rats. SD rats received a single intravenous dose of **D34** (2 mg/kg). Whole blood samples were collected before dosing and at 0.25, 0.5, 1, 2, 4, 6, 8, and 24 h post‐dose, and plasma was separated. Plasma drug concentrations were determined by LC‑MS/MS, and the primary pharmacokinetic parameters were estimated by non‑compartmental analysis. The results are summarized in Table 3. The elimination half‑life (t_1/2_) of **D34** was 3.17 h, indicating a moderate elimination rate in vivo. Such a half‑life ensures a sufficient duration for target engagement while avoiding an excessively narrow window for target protein degradation that might result from overly rapid clearance; it also reduces the risk of drug accumulation associated with prolonged half‑lives. The peak concentration (C_max_) was 407 ng/mL, and the area under the plasma concentration–time curve (AUC_0‐t_) was 392.67 h·ng/mL, demonstrating that **D34** achieved a high level of systemic exposure following intravenous administration. Overall, **D34** exhibited favorable pharmacokinetic behavior in rats, with a moderate half‑life and adequate systemic exposure, both of which are conducive to its target protein degradation activity. In addition, we assessed the passive membrane permeability of **D34** using the Lipid‑PAMPA assay. As shown in Table S4, the permeability parameter –Log P_e_ was 8.8, indicating poor membrane permeability. This observation is consistent with the general characteristics of PROTAC molecules, which often suffer from limited membrane permeation owing to their relatively large molecular weight and high polar surface area [12, 42]. Although intravenous administration can temporarily circumvent the absorption barrier, poor permeability may restrict oral bioavailability as well as effective intracellular and tissue exposure. Therefore, improving permeability should be a key focus in subsequent structural optimization.

**TABLE 3:** Pharmacokinetic parameters of **D34**.

PK parameters	D34
C_max_ (ng/mL)	407
t_1/2_ (h)	3.17
T_max_ (h)	0.25
Cl (mL/min/kg)	84.22
V_ss_ (L/kg)	4.48
MRT (h)	0.88
AUC_0‐t_ (h·ng/mL)	392.67

## 3 Conclusion

Picornavirus‐associated diseases pose a significant threat to society due to their high transmissibility and widespread prevalence, yet specific antiviral agents targeting these viruses remain unavailable. Although existing small‐molecule inhibitors demonstrate potent antiviral efficacy, they frequently induce drug‐resistant mutations and exhibit limited broad‐spectrum activity. To address these challenges, we applied PROTAC technology to develop therapeutics against picornaviruses. In this study, we successfully designed and synthesized **D34**, the first highly efficient PROTAC molecule targeting the 3C^Pro^ of picornaviruses. **D34** employs a reversible covalent binding strategy that extends its duration of action and enhances degradation efficiency through dynamic binding‐dissociation cycles. **D34** demonstrated broad‐spectrum degradation activity against the 3C^Pro^ of multiple picornaviruses, including EV71, CVA16, CVB3, HRV, PV, and ECHO. This broad‐spectrum activity suggests its potential cross‐protective efficacy against future outbreaks of structurally similar picornaviruses. Mechanistic investigations confirmed that **D34**‐mediated degradation depends on the ubiquitin‐proteasome system. Using the CAPTURE model, we identified potential drug‐resistance mutation sites in EV71 3C^Pro^ and validated **D34**’s degradation capability against both predicted high‐risk mutants and reported drug‐resistant variants. Furthermore, **D34** exhibited significant antiviral efficacy against EV71 through a dual mechanism: direct inhibition of protease activity and targeted protein degradation. Our work not only provides a promising therapeutic candidate for picornaviruses but also offers a strategy for developing drug‐resistant, broad‐spectrum antiviral agents through PROTAC technology.

## 4 Experimental Section

### 4.1 Chemistry

All reagents were purchased from commercial suppliers and used without further purification. NMR spectra were recorded on a Bruker Ascend 400 in the indicated solvent. (400 MHz for ^1^H and 101 MHz for ^13^C) (Bruker, Karlsruhe, Germany) NMR spectrometer. Molecular mass was determined on a mass spectrometer (Shimadzu (China) Co., Ltd.). HPLC analysis was performed using the general method: equipment = Agilent 1260 HPLC; column = Phenomenex Luna C18 5micron column (250 mm × 4.60 mm, 5 µm); tested temporary = 35°C; solvent = MeCN/0.1% TFA dissolved in H_2_O; Gradient = 10%–90% MeCN in 0.1% TFA solution at 1 mL/min flow rate; detector = The UV detection at 254 and 270 nm. All final compounds were > 95% pure by HPLC analysis.

**SCHEME 1:**
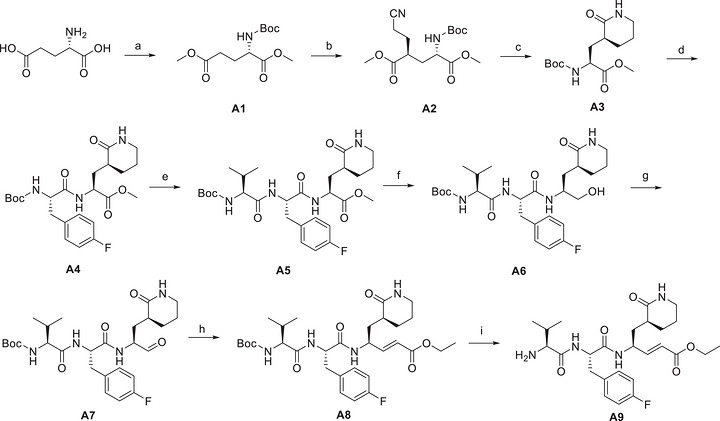
(a) (1) SOCl_2_, MeOH, reflux, 3 h; (2) (Boc)_2_O, TEA, THF, RT, 12 h, 94%; (b) (1) LiHDMS, THF, ‐78°C, 2 h; (2) 3‐Bromopropionitrile, ‐78°C, 1.5 h, 60%; (c) CoCl_2_·6H_2_O, NaBH_4_, MeOH, RT, 24 h, 52%; (d) (1) TFA, DCM, RT, 4 h; (2) Boc‐L‐4‐F‐Phe‐OH, EDCI, HOBt, TEA, DCM, RT, 12 h, 68%; (e) (1) TFA, DCM, RT, 4 h; (2) Boc‐L‐Val‐OH, EDCI, HOBt, TEA, DCM, RT, 12 h, 70%; (f) NaBH_4_, MeOH, RT, 8 h, 85%; (g) DMP, DCM, 0°C, 2 h, 70%; (h) Ethyl triphenylphosphoranylideneacetate, DCM, RT, 8 h, 75%. (i) TFA, DCM, RT, 4 h, 80%.

As shown in Scheme 1, L‐glutamic acid undergoes esterification, followed by protection of its amino group with a Boc protecting group, yielding intermediate **A1. A1** reacts with lithium hexamethyldisilazide to form a γ‐enol intermediate, which then undergoes alkylation with 3‐bromopropionitrile to produce compound **A2**. In a reduction system composed of sodium borohydride and cobalt (II) chloride hydrate, **A2** is converted into (S)‐δ‐lactam **A3**. Subsequently, **A3** undergoes Boc deprotection under trifluoroacetic acid (TFA) and condenses with Boc‐protected L‐4‐fluorophenylalanine to form **A4**. The synthesis of **A5** follows the same route as **A4**. After reduction of **A5** to alcohol **A6** with sodium borohydride, oxidation with Dess‐Martin periodinane yields aldehyde **A7. A7** then undergoes a Wittig reaction with ethyl triphenylphosphoranylideneacetate to generate alkene intermediate **A8**. Finally, A8 is subjected to Boc deprotection under TFA conditions to obtain the key intermediate **A9**.

**SCHEME 2:**
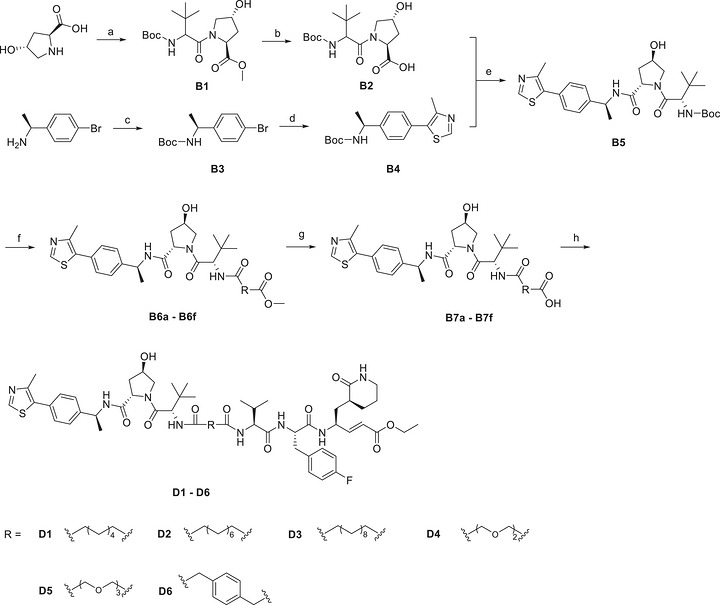
(a) (1) SOCl_2_, MeOH, 68°C, 3 h; (2) Boc‐L‐tert‐leucine, EDCI, HOBt, TEA, DCM, RT, 12 h, 65%; (b) LiOH·H_2_O, THF, H_2_O, RT, 4 h, 70%; (c) (Boc)_2_O, TEA, THF, RT, 12 h, 83%; (d) Potassium Acetate, Pd(OAc)_2_, 4‐Methythiazole, DMF, 90°C, 12 h, 60%; (e) (1) THF, DCM, RT, 4 h; (2) B2, EDCI, HOBt, TEA, DCM, RT, 12 h, 77%; (f) (1) TFA, DCM, RT, 4 h; (2) Monomethyl suberate, EDCI, HOBt, TEA, DCM, RT, 12 h, 60–70%; (g) LiOH·H_2_O, THF, H_2_O, RT, 4 h, 60–70%; (h) (1) TFA, DCM, RT, 4 h; (2) A9, EDCI, HOBt, TEA, DCM, RT, 12 h, 50–60%.

The synthesis of PROTACs **D1‐D6** is outlined in Scheme 2. First, L‐hydroxyproline was esterified and then condensed with Boc‐protected L‐isoleucine to afford intermediate **B1**. **B1** underwent deprotection under LiOH conditions to yield **B2**. Concurrently, the amine group of (S)‐(‐)‐1‐(4‐bromophenyl)ethylamine was Boc‐protected and reacted with 4‐methylthiazole to generate **B4**. **B4** was subjected to Boc‐deprotection using TFA and subsequently condensed with **B2** to form **B5**. Following Boc‐deprotection, **B5** was condensed with various linkers to afford **B6a‐B6f**. Compounds **B7a‐B7f** were synthesized following the same procedure as for **B2**. Finally, **B7a‐B7f** were condensed with intermediate **A9** to furnish the final products **D1‐D6**.

**SCHEME 3:**
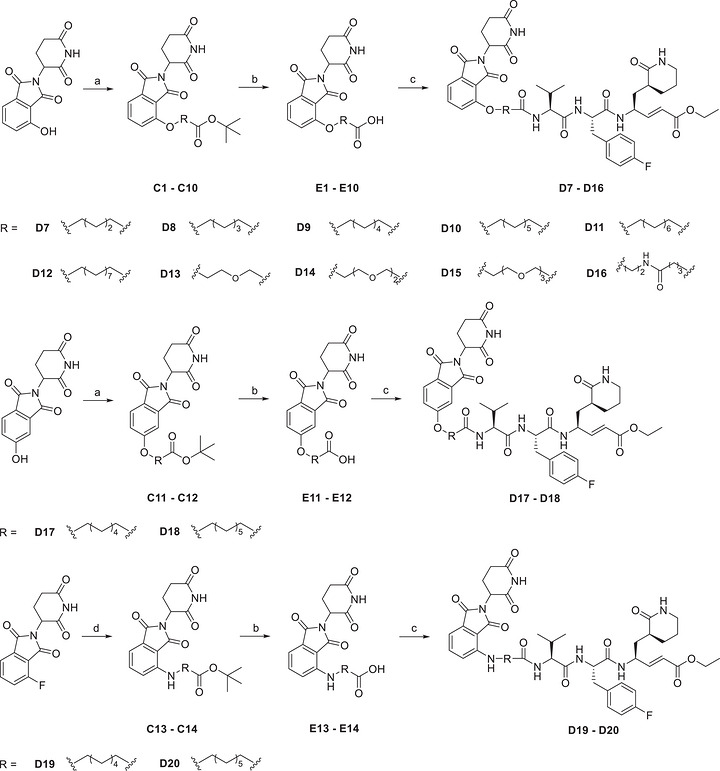
(a) K_2_CO_3_, DMF, 30%–40%. (b) CF_3_CO_2_H, DCM, 80%–90%. (c) **A9**, EDCI, HOBt, TEA, DCM, RT, 12 h, 50%–60%. (d) DIPEA, DMF, 30%–40%.

The synthesis of PROTACs **D7‐D20** is shown in Scheme 3. 2‐(2,6‐dioxopiperidin‐3‐yl)‐4‐hydroxyindole‐1,3‐dione reacts with various bromine‐substituted linker chains of different lengths to form **C1‐C10. C1‐C10** undergoes tBu deprotection under trifluoroacetic acid conditions to yield **E1‐E10. E1‐E10** condense with the intermediate **A9** to produce the final products **D7‐D16**. The synthesis of **D17‐D20** follows a similar method.

**SCHEME 4:**
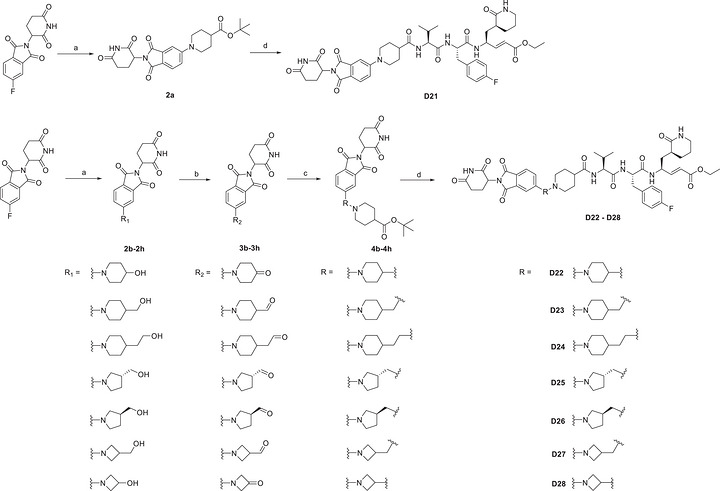
(a) DIPEA, DMF, 40%–50%. (b) DMP, DCM, 70%–80%. (c) STAB, DIPEA, DMF, 30%–40%. (d) (1) TFA, DCM; (2) A9, HATU, HOBt, DIPEA, 30%–40% for two steps.

The synthesis of PROTACs **D21‐D28** is shown in Scheme 4. 2‐(2,6‐dioxopiperidin‐3‐yl)‐5‐fluoroindole‐1,3‐dione reacts with different cyclic secondary amines to generate **2a‐2 h**. 2b‐2 h are oxidized to the corresponding aldehydes or ketones **3b‐3 h** using DMP. **3b‐3 h** undergo reductive amination with 4‐piperidinemethyl ester to form **4b‐4h**. 2a condenses with **4b‐4h** under trifluoroacetic acid conditions after tBu deprotection to yield the final products **D21‐D28**.

**SCHEME 5:**
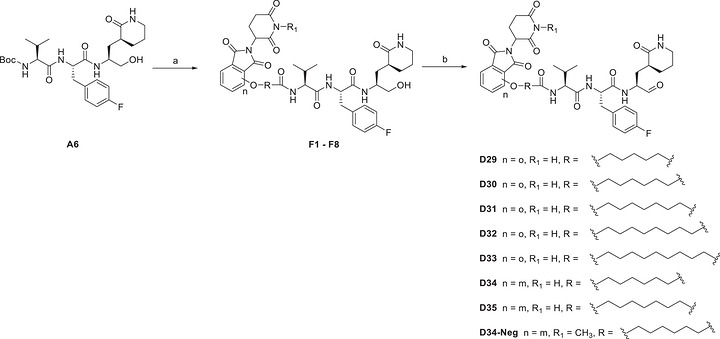
(a) (1) TFA, DCM; (2) HATU, HOBt, DIPEA, 40%–50% for two steps. (b) DMP, DCM, 70%–80%.

As shown in Scheme 5, after Boc deprotection of **A6** under trifluoroacetic acid conditions, it condenses with various acids to form **F1‐F8. F1‐F8** are oxidized using DMP to yield the corresponding aldehydes **D29‐D35** and **D34‐Neg**.

### 4.2 Cell Lines

HEK293T and RD cells were purchased from the American Type Culture Collection (ATCC) and authenticated. HEK293T cells were cultured in DMEM medium (C11995500BT, Gibco) supplemented with 10% heat‐inactivated FBS (FSD500, ExCell) and 1% penicillin‐streptomycin (P1400, Solarbio). RD cells were cultured in high‐glucose DMEM medium supplemented with 10% FBS and 1% penicillin‐streptomycin. All cell lines were maintained in a humidified atmosphere at 37°C with 5% CO_2_.

### 4.3 Western Blotting Analysis

Cells were washed twice with PBS. Subsequently, cells were lysed on ice for 10 min using RIPA lysis buffer (Solarbio, R0010) supplemented with PMSF (Solarbio, P0100) and a protease inhibitor cocktail (MCE, HY‐K0010). The lysate was centrifuged at 13500 g for 15 min at 4°C, and the supernatant was collected. The supernatant was mixed with 5× SDS sample loading buffer (Solarbio, P1040) and heated at 98°C for 10 min. Samples were then resolved by sodium dodecyl sulfate‐polyacrylamide gel electrophoresis (SDS‐PAGE) on 4%–20% gradient gels and electrophoretically transferred to PVDF membranes (Millipore, IPVH00010). Membranes were blocked with 5% non‐fat dry milk (Biosharp, BS102) in phosphate‐buffered saline containing 0.1% Tween 20 (PBST) for 2 h at room temperature, followed by incubation with primary antibodies (anti‐Flag (proteintech, 66008‐4‐Ig), anti‐EV71 3C (GeneTex, GTX132357), anti‐HA (proteintech, 66006‐2‐Ig), β‐actin (proteintech, 66009‐1‐Ig)) overnight at 4°C. After primary antibody incubation, membranes were washed four times with PBST for 5 min each at room temperature. Membranes were then incubated with horseradish peroxidase (HRP)‐conjugated secondary antibodies (mouse (Solarbio, SE131) or goat (Solarbio, SE134)) for 1 h at room temperature. Following secondary antibody incubation, membranes were washed four times with PBST for 5 min each at room temperature. Finally, the membranes were incubated with chemiluminescent substrate (Millipore, WBKLS0500) and visualized using a chemiluminescence imaging system.

### 4.4 EV71 3C^Pro^ Enzyme Inhibition Assay

The EV71 3C^Pro^ was expressed and purified as previously described. The EV71 3C^Pro^ (1 µM) was incubated with different concentrations of compounds at 37°C in the assay buffer (50 mM Tris‐HCl, 150 mM NaCl, 2 mM DTT, 1 mM EDTA, pH 7.0) for 30 min. Subsequently, the substrate NMA‐IEALFQGPPK(DNA)FR (20 µM) was added to initiate the reaction, and the mixture was incubated at 37°C for 1.5 h. The changes in relative fluorescence units were measured using a microplate reader (Thermo Varioskan Flash) at λex of 380 nm and λem of 460 nm. The percentage of inhibition was calculated based on controls without compounds (100% activity) and blank controls. The IC_50_ value was calculated using GraphPad Prism software.

### 4.5 Establishment of HEK293T Cells Stably Expressing 3C^Pro^‐Flag

The HEK293T cells were seeded in high‐glucose DMEM containing 10% FBS one day prior to transfection, ensuring that the cell confluence the next day was approximately 70%–90%. The transfer vector pLVX‐EV71 3C^Pro^‐Flag or the empty vector pLVX‐vector was co‐transfected into the HEK293T cells using the PolyJet DNA transfection reagent (SignaGen, SL100688), along with the packaging plasmids psPAX2 and pMD2.G for lentivirus production. The cell supernatants were collected 36 and 72 h post‐transfection, centrifuged to remove cell debris, and filtered through a 0.45 µm filter to obtain lentivirus particles. To establish stable expression cell lines, the target HEK293T cells were plated one day prior to infection to achieve approximately 50% confluence the next day. During infection, Polybrene (GLPBIO, GC19206) was added to the medium at a final concentration of 10 µg mL^−1^ to enhance transduction efficiency. The medium was replaced with selection medium containing puromycin (Solarbio, P8230) at a final concentration of 1 µg mL^−1^ for resistance selection 24 h post‐infection, until the untransduced control cells were completely dead. Finally, Western blot analysis was performed to detect the expression of the EV71 3C^Pro^ to confirm the successful construction of the stable cell line.

### 4.6 Protein Degradation Assay

The stable transfected cells were seeded into 12‐well plates, and when the cell density reached approximately 80%, they were treated with 2 µM of different compounds (**D1**‐**D28**) for 24 h, or with 2 µM of compounds for varying durations from 2 h to 48 h, or treated with a series of dilutions of compounds (**D34**, **D34‐Neg**) ranging from 80 nM to 20 000 nM for 24 h. Finally, Western blot analysis was conducted to detect the content of EV71 3C^Pro^. Densitometric analysis was performed using ImageJ software. The DC_50_ values were calculated using GraphPad Prism software.

### 4.7 Dilution Assay

EV71 3C^Pro^ (0.5 µM) was incubated with 5 µM of the inhibitor in assay buffer (50 mM Tris, 150 mM NaCl, 1 mM EDTA, 10% glycerol, pH 7.0) at 30°C for 1 h, with a blank control group set up in parallel. The total volume was 500 µL, containing 5% organic solvent. The mixture was transferred to an ultrafiltration spin column (Amicon Ultra‐0.5, 10 kDa MWCO) and centrifuged at 4°C and 12 000 rpm for 30 min until the remaining volume was approximately 50 µL, thereby removing the excess small molecule. Assay buffer was then added to bring the volume back to 500 µL. A 95 µL aliquot of the mixture was mixed with 5.0 µL of substrate working solution (2.0 mM) to initiate the reaction. Fluorescence changes were monitored using a microplate reader, and the extent of substrate cleavage in the experimental and control groups was recorded to calculate the percentage of recovered enzyme activity.

### 4.8 Degradation Mechanism Experiments

The stable transfected cells were seeded into 12‐well plates and treated with 10 µM **A8**, 10 µM Pomalidomide (MCE, HY‐10984), 5 µM MLN4924 (MCE, HY‐70062), 5 µM TAK‐243 (MCE, HY‐100487), 5 µM Carfilzomib (MCE, HY‐10455), or 5 µM Bortezomib (MCE, HY‐10227) for 4 h when the cell density reached approximately 80%, and DMSO was used as a vehicle control. After treatment, 1 µM **D34** was added for continued incubation for 24 h, followed by the collection of the cells. Finally, Western blot analysis was conducted to detect the content of EV71 3C^Pro^. Densitometric analysis was performed using ImageJ software. The statistical graphs were plotted using GraphPad Prism software.

### 4.9 Co‐IP Assay

The stable transfected cells were first treated with **D34** (5 µM or 10 µM) for 12 h, followed by a combination of **D34** (5 µM or 10 µM) and MG132 (5 µM) treatment for 12 h. Cell lysates were prepared. The supernatants were immunoprecipitated using Anti‐Flag Nanobody Magarose Beads (AlpalifeBio, KTSM1338) and incubated overnight at 4°C. After incubation, the beads were washed three times in wash buffer (50 mM Tris–HCl, pH 7.5, 150 mM NaCl, 1 mM EDTA) and boiled in 1× loading buffer. Finally, protein samples were analyzed by SDS‐PAGE, followed by immunoblotting with various antibodies indicated.

### 4.10 Mutation Prediction Model

The pre‐trained CAPTURE models, fine‐tuned on the PDBbind 2020 dataset, were provided by the original authors. For further fine‐tuning, we collected a dataset comprising 146 SARS‐CoV‐2 3CL^Pro^ protein‐ligand complexes as training set and 12 picornavirus‐related 3C data as validation set (including 5 EV71 3C^Pro^‐ligand complexes). For resistance mutation prediction, we used the crystal structure of the EV71 3C^Pro^‐ligand complex (PDB ID: 5BPE), in which the ligand is highly similar to the POI ligand in the PROTAC, making it suitable for subsequent predictions of changes in the binding affinity (BA) values of the mutant proteins relative to the wild‐type (WT). We focused on amino acid residues within 6 Å of the binding pocket, particularly those in the P1 and P2 binding pockets, and selected 20 mutation sites. For each of these sites, we considered 19 possible mutations, performing residue mutation and energy minimization using Rosetta based on the WT EV71 3C^Pro^ structure, resulting in 380 single‐point mutant proteins. Single‐residue mutations were introduced using the Rosetta modeling suite, in which each mutation was modeled independently while preserving the overall backbone conformation. The binding affinity change (ΔBA = BAmut—BA_WT_) was defined as the difference between the predicted binding affinity of the mutant and that of the wild‐type complex. From a drug–target interaction perspective, ΔBA quantifies mutation‐induced perturbations within the binding pocket, including alterations in steric complementarity, electrostatic interactions, and hydrogen‐bonding networks that collectively govern inhibitor binding strength. The BA changes for each mutant were predicted using the fine‐tuned model. Additionally, the fitness of the mutant proteins was assessed using the ESM tool. Based on the ranking of ΔBA values and the extent of fitness change, we identified the final resistance mutation sites for experimental validation.

### 4.11 Mutations of EV71 3C^Pro^

C147A, N69S, A144V, S41M, S41I, L102A and T106E mutations were performed by PCR‐driven overlap extension and then mutants were sequenced to confirm that mutations were made successfully. The primers used for generating the mutants as follows:

EV71 3C^Pro^:

Forward‐ ATGGGCCCGAGCCTTG

Reverse‐ TTGTTCACTAGCAAAGTAACTCCTTTTGAGACCCG

EV71 3C^Pro^(C147A):

Forward 1‐ ACTAAAGCAGGACAGGCTGGAGGAGTGGTGACA

Reverse 1‐ TGTCACCACTCCTCCAGCCTGTCCTGCTTTAGT

EV71 3C^Pro^(N69S):

Forward 2‐ GATGAGCAAGGAGTCAGCTTGGAATTAACCCTC

Reverse 2‐ GAGGGTTAATTCCAAGCTGACTCCTTGCTCATC

EV71 3C^Pro^(A144V):

Forward 3‐ ACTTTCCTACTAAATGCGGACAGTGTGGAGGAGTGGTGACA

Reverse 3‐ TGTCACCACTCCTCCACACTGTCCGCATTTAGTAGGAAAGT

EV71 3C^Pro^(S41M):

Forward 4‐ GCAGTCCTCCCTCGCCACATGCAACCCGGCAAAACGATTTG

Reverse 4‐ CAAATCGTTTTGCCGGGTTGCATGTGGCGAGGGAGGACTGC

EV71 3C^Pro^(S41I):

Forward 5‐ GTCCTCCCTCGCCACATACAACCCGGCAAAACGAT

Reverse 5‐ ATCGTTTTGCCGGGTTGTATGTGGCGAGGGAGGAC

EV71 3C^Pro^(L102A):

Forward 6‐ AGCACTGCCAGTGATGCCACCGCTGTGATCAACACGGAGCAC

Reverse 6‐ GTGCTCCGTGTTGATCACAGCGGTGGCATCACTGGCAGTGCT

EV71 3C^Pro^(T106E):

Forward 7‐ GCCACCTTAGTGATCAACGAAGAGCACATGCCCTCAATGT

Reverse 7‐ ACATTGAGGGCATGTGCTCTTCGTTGATCACTAAGGTGGC

### 4.12 Resistance Induction Experiment

EV71 virus at a MOI of 0.01 was inoculated into six‐well plates, with each well containing 2 mL of culture medium supplemented with the corresponding concentration of **D34**. Drug concentrations were increased in a stepwise manner: early passages (P1‐P5) used 1 × EC_50_, intermediate passages (P6‐P10) were raised to 2 × EC_50_, and late passages (P11‐P15) were further increased to 4 × EC_50_. At each passage, the cytopathic effect (CPE) was monitored by microscopy; when obvious CPE was observed, the supernatant was collected for the next round of infection. In the control group, an equal volume of DMSO was used instead of the compounds, and the passaging procedure was identical to that of the experimental groups. After the experiment, EC_50_ values of **D34** against each viral strain were determined.

### 4.13 Antiviral Activity Assay

EV71 (Fuyang strain) was generously provided by Associate Professor Wang Yaxin from the School of Life Sciences at Tianjin University. RD cells were treated with compounds at designated concentrations and then infected with EV71 virus at an MOI of 0.1 for 2 h. The supernatant was removed, and the cells were washed twice with PBS, then fresh culture medium containing the specified concentration of compounds was added. The cells were incubated at 37°C for 24 h. On the one hand, total RNA was extracted 24 h post‐infection. After reverse transcription to cDNA, the viral copy number in the cells was quantified by real‐time quantitative PCR (qRT‐PCR) to evaluate antiviral activity. On the other hand, the content of EV71 3C^Pro^ in the cells was detected by Western blotting.

### 4.14 Cytotoxicity Assay

Different concentrations of compounds were used to treat HEK293T and RD cells for 24 h, and cell viability was assessed using a cell counting assay. The percentage of inhibition was calculated based on the control wells containing DMSO. Graphs were generated using GraphPad Prism software.

### 4.15 Quantitative Proteomic Analysis

The stable transfected cells were treated with **D34** or DMSO. After 24 h, proteins were extracted. Protein samples were mixed with DB solubilization buffer (6 M urea, 100 mM TEAB, pH = 8.5) to a final volume of 100 µL, followed by the addition of trypsin and 100 mM TEAB buffer. The mixture was incubated at 37°C for 4 h for enzymatic digestion. Formic acid was added to adjust the pH to below 3, and the mixture was centrifuged at 12 000 g for 5 min at room temperature. The supernatant was slowly passed through a C18 desalting column, followed by three washes with wash solution (0.1% formic acid, 3% acetonitrile). Then, an appropriate amount of elution solution (0.1% formic acid, 70% acetonitrile) was added, and the filtrate was collected and freeze‐dried. For peptide and protein identification, LC−MS/MS spectra were collected using a Vanquish Neo UHPLC—Astral LC/MS DIA. The raw data files were analyzed using DIA‐NN software (Direct DIA) and compared with a merged database of the UniProt human proteome and EV71 virus. A T‐test was performed to statistically analyze the protein quantification results, and proteins with significant quantitative differences between the experimental and control groups (*p* < 0.05, |log_2_FC| > * (FC > * or FC < * [fold change, FC])) were defined as differentially expressed proteins.

### 4.16 Molecular Docking

Molecular docking was performed using the CovDock module of Schrödinger software. The receptor (PDB code: 5BPE) was prepared using the Protein Preparation Wizard, and ligands were constructed and optimized using the LigPrep module before docking. Covalent docking was used in the simulation. The original inhibitor in the crystal structure was set as a reference ligand and placed in the center of a 20 Å^3^ cubic closed box. Cys147 was selected as the active residue.

Computational modeling of the CRBN‐**D34**‐3C^Pro^ ternary complex was performed. Based on the amino acid sequences of the CRBN protein and the EV71 3C^Pro^, along with the two‐dimensional structure (in SDF format) of the PROTAC molecule, ternary complex modeling was conducted using AlphaFold3. During the modeling process, a covalent bond was specified between the aldehyde group of the PROTAC molecule and Cys147 of the target protein. Subsequently, the binding free energy of the CRBN‐**D34**‐3C^Pro^ ternary complex was calculated using the PRODIGY tool. A negative binding free energy value indicates that the complex formation process is exergonic, signifying a thermodynamically favorable process where the system tends toward stability. Furthermore, a more negative value correlates with higher thermodynamic stability of the complex.

### 4.17 In Vivo Pharmacokinetics in SD Rats

Pharmacokinetic studies were performed in SD rats (*n* = 3). Pharmacokinetic parameters were obtained following intravenous administration (2.0 mg/kg). After dosing, blood samples were collected at the following specified time points: 0, 0.25, 0.5, 1, 2, 4, 6, 8, and 24 h post‐injection. The collected whole blood was centrifuged at 4°C and 3000 g for 10 min to obtain plasma samples (0.25 mL), which were stored at −20°C until analysis. The plasma samples were analyzed by LC‑MS/MS.

## Author Contributions

**Yingyue Pang**: data curation. **Yuanyuan Zhang**: data curation. **Weilong Deng**: conceptualization, methodology, software, data curation, investigation, formal analysis, writing – original draft, validation. **Guoliang You**: data curation. **Junyu Chen**: software, data curation. **Zhongxin Xu**: writing – review and editing, supervision. **Siqian Chen**: data curation. **Xiaoman Tian**: data curation. **Luqing Shang**: project administration, resources, conceptualization, methodology, writing – review and editing, funding acquisition. **Jing Wang**: writing – review and editing, software, supervision, visualization.

## Conflicts of Interest

The authors declare no conflicts of interest.

## Supporting information




**Supporting File**: advs76662‐sup‐0001‐SuppMat.pdf.

## Data Availability

The data that support the findings of this study are available in the supplementary material of this article.
