# Supplementary material for: Targeted Degradation of Picornaviral 3C Protease via PROTACs Confers High Barrier to Viral Resistance and Broad‐Spectrum Antiviral Activity
Source: Adv Sci (Weinh). 2026 Jul 17;13(57):e76662. doi: 10.1002/advs.76662 (PMC13379210; doi:10.1002/advs.76662)

Supporting Information

Targeted Degradation of Picornaviral 3C Protease via PROTACs Confers High Barrier to Viral Resistance and Broad-Spectrum Antiviral Activity

Weilong Deng, Junyu Chen, Yingyue Pang, Yuanyuan Zhang, Siqian Chen, Guoliang You, Xiaoman Tian, Zhongxin Xu*, Jing Wang*, Luqing Shang*

[*] W. Deng, J. Chen, Y. Pang, Y. Zhang, S. Chen, G. You, X. Tian, Dr. Z. Xu*, Dr. J. Wang*, Prof. Dr. L. Shang*

State Key Laboratory of Medicinal Chemical Biology, College of Pharmacy, KLMDASR of Tianjin and Drug Discovery Center for Infectious Disease

Nankai University

Tianjin, 300350, P. R. China

E-mail: shanglq@nankai.edu.cn; wangjing@nankai.edu.cn; xuzx@nankai.edu.cn

Content

(78 pages)

Part A. Supporting Tables and Figures.. S3-S8

Part B. Chemistry S9-S42

Part C. Copies of Spectra.. S43-S78

Part A. Supporting Tables and Figures

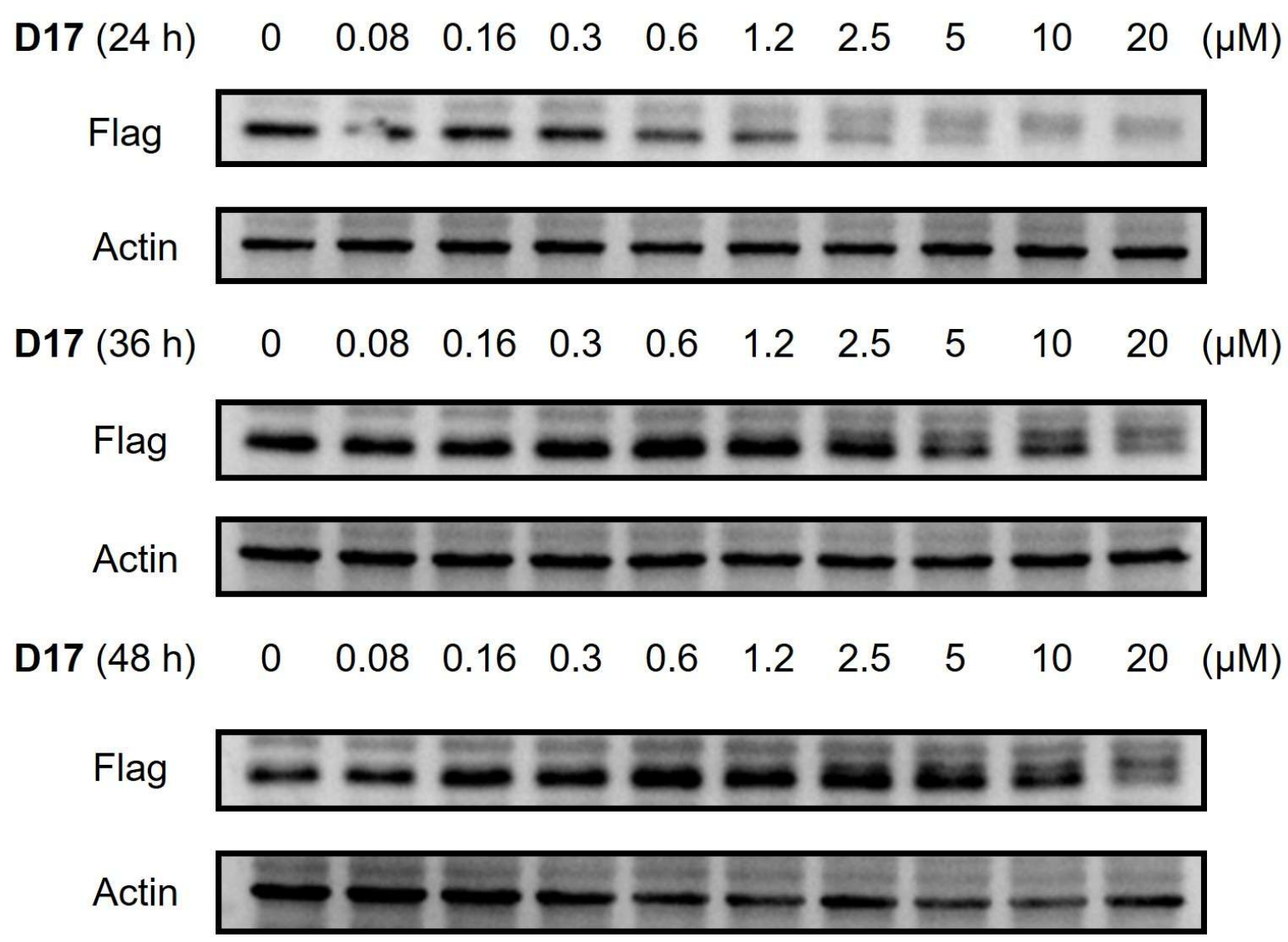

Figure S1. The potency of **D17** in degrading $3C^{Pro}$ was evaluated in the HEK293T cells stably expressing $3C^{Pro}$-Flag by Western blotting after the cells were treated with different concentrations for 24 h, 36 h and 48 h.

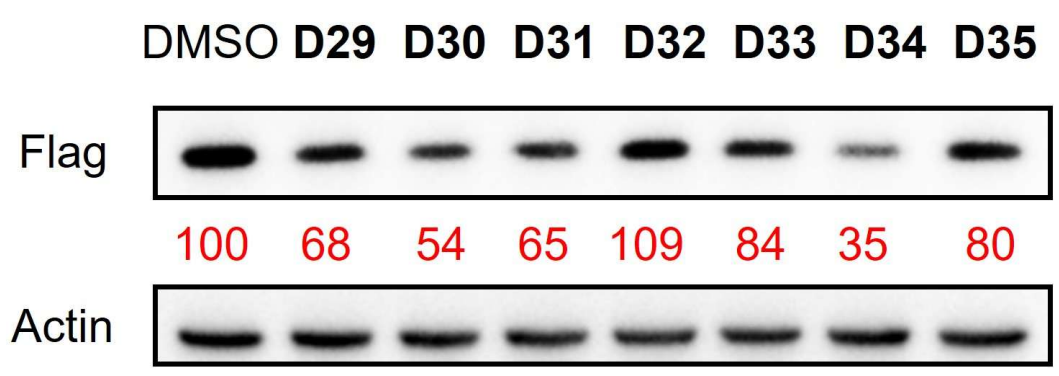

Figure S2. The potency of **D29-D35** in degrading $3C^{Pro}$ was evaluated in the HEK293T cells stably expressing $3C^{Pro}$-Flag by Western blotting after the cells were treated with 0.5 μM PROTACs for 24 h.

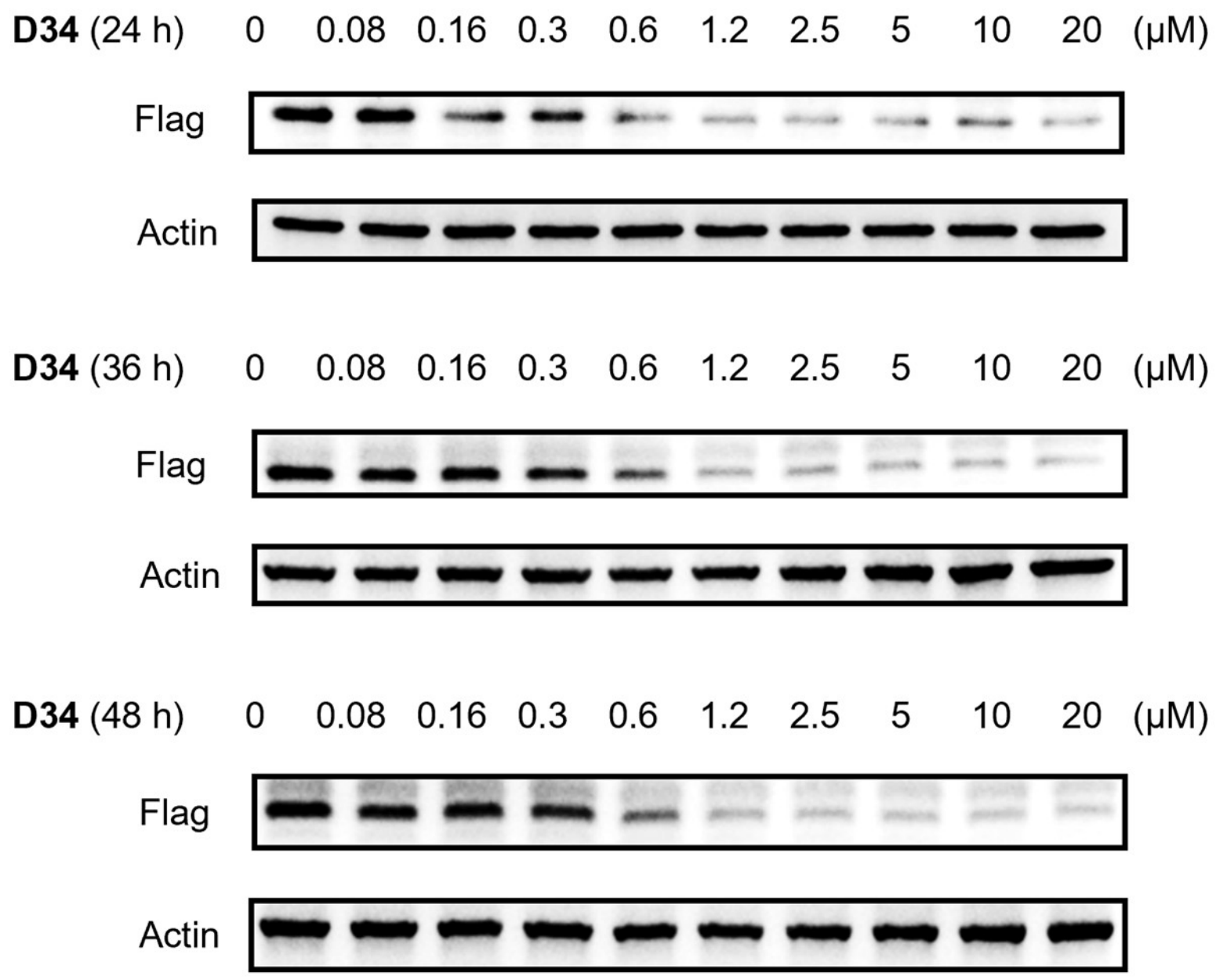

Figure S3. The potency of **D34** in degrading $3C^{Pro}$ was evaluated in the HEK293T cells stably expressing $3C^{Pro}$-Flag by Western blotting after the cells were treated with different concentrations for 24 h, 36 h and 48 h.

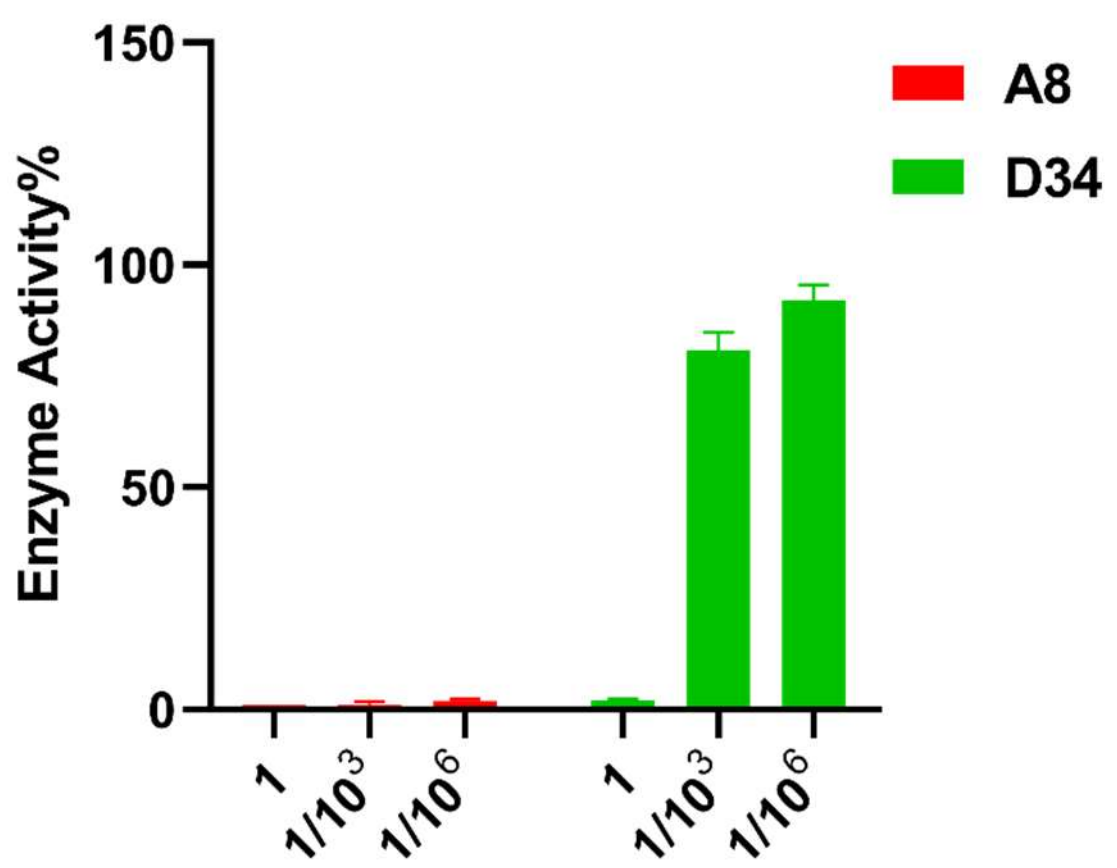

Figure S4. Recovery of enzyme activity (%) after ultrafiltration dilution of the $3C^{Pro}$-inhibitor complex (n = 3).

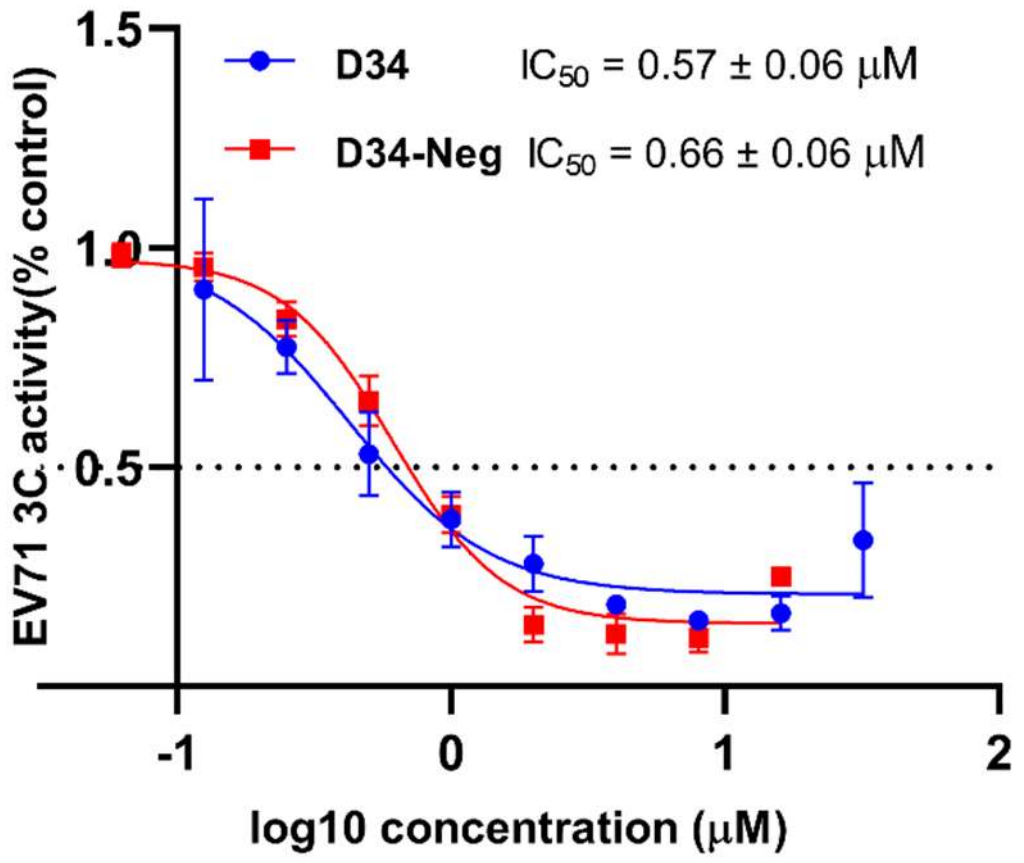

Figure S5. IC_{50} fitting curves for **D34** and **D34-Neg**. Quantitative results, summarized from three replicates, are expressed as mean ± SD.

Table S1. The predicted binding affinity of $3C^{Pro}$-CRBN and $3C^{Pro}$-**D34**-CRBN via PRODIGY.

combination mode	$3C^{Pro}$-CRBN	$3C^{Pro}$-D34-CRBN
Predicted binding affinity ($kcal.mol^{-1}$)	-9.4	-13.11

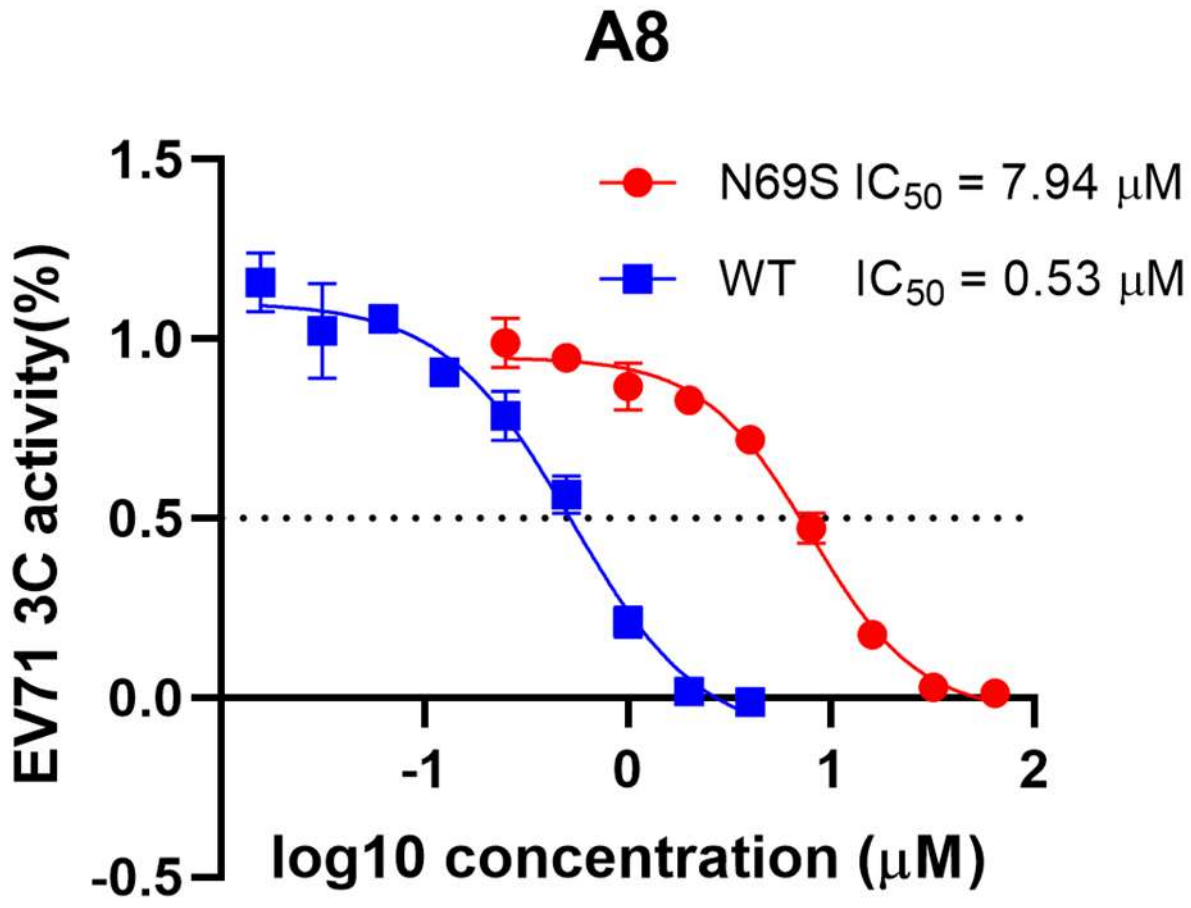

Figure S6. IC_{50} fitting curves for **A8**. Quantitative results, summarized from three replicates, are expressed as mean ± SD.

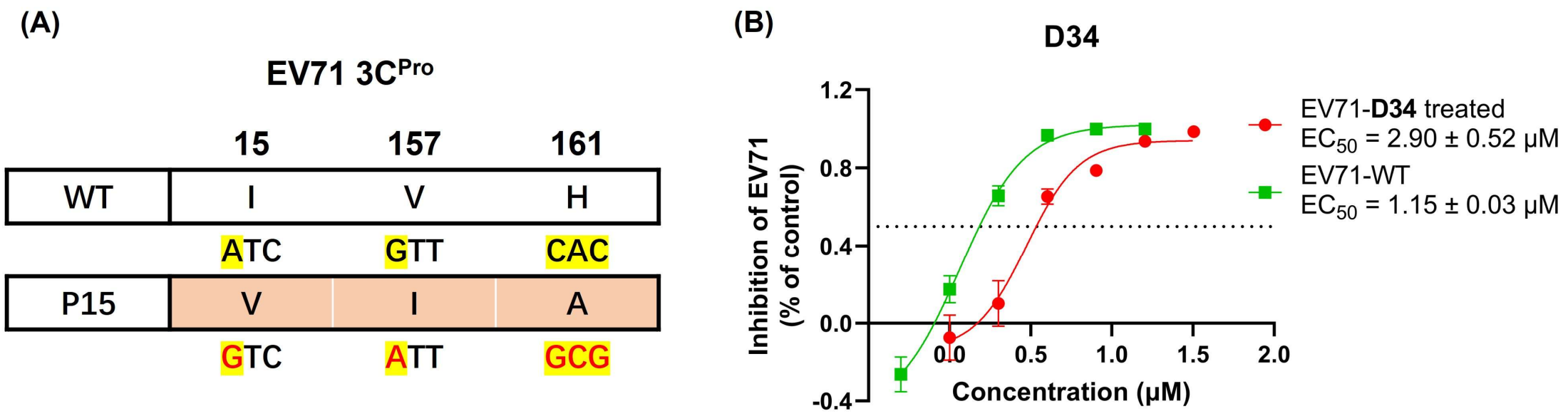

Figure S7. (A) Mutations in the 3C protease of the viral strain following serial passaging under high-concentration **D34** pressure. (B) EC_{50} fitting curves for **D34**. Quantitative results, summarized from three replicates, are expressed as mean ± SD.

Table S2. Potential drug-resistant mutation sites predicted by CAPURE model (displayed in the top 20, ranked by ΔBA value).

Ranking	mutation site	ΔBA
1	A144C	-0.72128963
2	S41M	-0.56528234
3	S41I	-0.54531765
4	L102A	-0.42271185
5	T106E	-0.40055323
6	T142C	-0.37662268
7	K143N	-0.34453535
8	L102G	-0.3211646
9	Q146G	-0.32011557
10	K143F	-0.3198638
11	G23E	-0.31985617
12	S41Y	-0.3175931
13	L102Y	-0.3162861
14	T142E	-0.31553507
15	N69S	-0.3099618
16	T106L	-0.30820417
17	G145S	-0.3076377
18	G145T	-0.300035
19	G148A	-0.29843473
20	K143A	-0.29410124

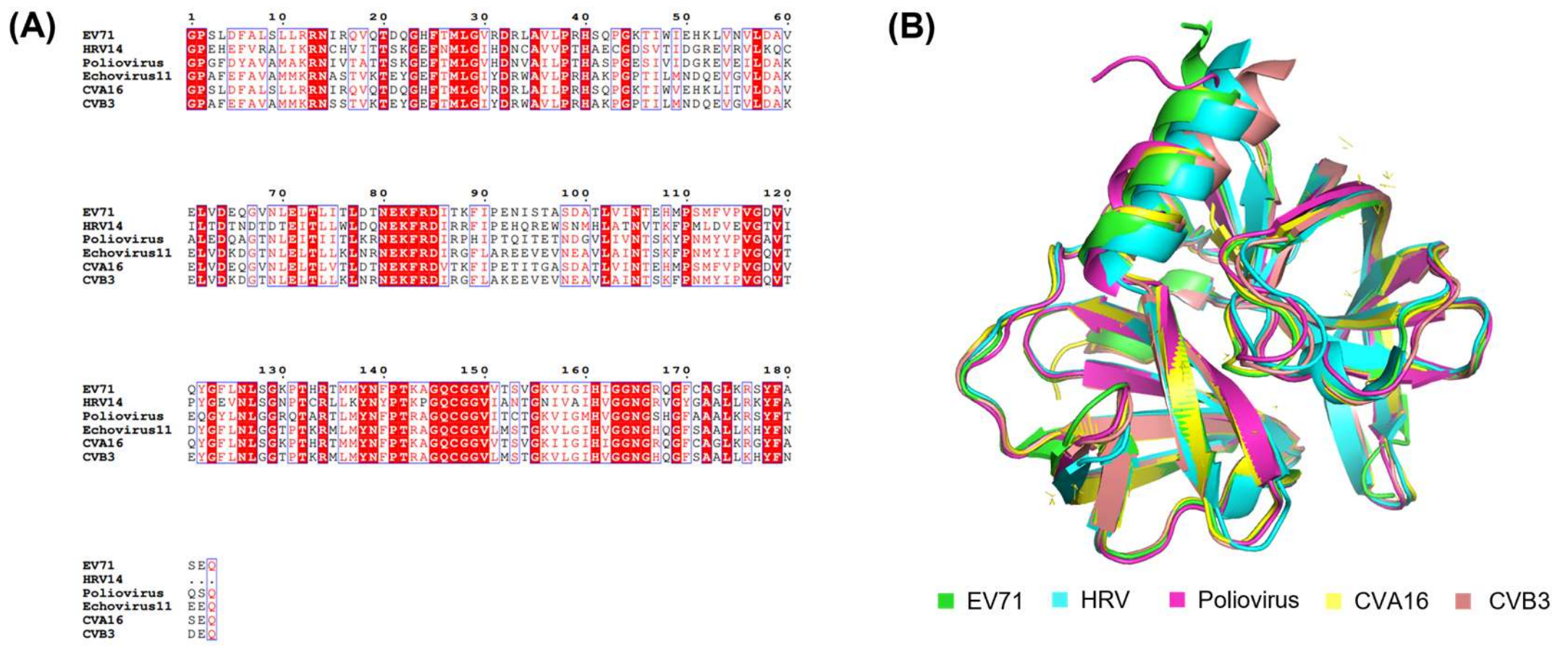

Figure S8. (A) Amino acid sequence alignment of EV71, CVA16, CVAB3, HRV14, PV, and ECHO 3C proteases. (B) Comparison diagram of crystal structures of EV71, CVA16, CVAB3, HRV and PV 3C proteases.

Table S3. The predicted binding affinity of $3C^{Pro}$-**D34**-CRBN via PRODIGY.

Binding mode	Predicted binding affinity ($kcal.mol^{-1}$)
EV71 $3C^{Pro}$-**D34**-CRBN	-13.11
HRV $3C^{Pro}$-**D34**-CRBN	-8.3
PV $3C^{Pro}$-**D34**-CRBN	-9.8
ECHO11 $3C^{Pro}$-**D34**-CRBN	-9.5
CVA16 $3C^{Pro}$-**D34**-CRBN	-9.0
CVB3 $3C^{Pro}$-**D34**-CRBN	-9.2

Table S4. Permeability Determination of **D34** by Lipid-PAMPA Method.

Compound	-Log Pe	Recovery%
**D34**	8.80	72.00

Part B. Chemistry

All reagents were purchased from commercial suppliers and used without further purification. NMR spectra were recorded on a Bruker Ascend 400 in the indicated solvent. (400 MHz for ^{1}H and 101 MHz for ^{13}C) (Bruker, Karlsruhe, Germany) NMR spectrometer. Molecular mass was determined on a mass spectrometry (Shimadzu (China) Co., Ltd.). HPLC analysis was performed using the general method: equipment = Agilent 1260 HPLC; column = Phenomenex Luna C18 5micron column (250 mm × 4.60 mm, 5 μm); tested temporary = 35 °C; solvent = MeCN/0.1% TFA dissolved in H_2O; Gradient = 10-90% MeCN in 0.1% TFA solution at 1 mL/min flow rate; detector = The UV detection at 254 nm and 270 nm. All final compounds were > 95% pure by HPLC analysis.

Synthesis of compound A1: L-Glutamic acid (20.00 g, 136.00 mmol) was dissolved in methanol (300 mL). Thionyl chloride (19.90 g, 163.20 mmol) was added dropwise to the solution under ice-bath cooling. After complete addition, the mixture was stirred at room temperature for 10 min, then heated to reflux at 78°C for 3 h in an oil bath. Upon completion, the solvent was removed under reduced pressure to afford a colorless oil. This crude product was dissolved in THF (300 mL), followed by sequential addition of di-tert-butyl dicarbonate (35.60 g, 163.20 mmol) and triethylamine (48.30 mL, 340.00 mmol) under ice-bath cooling. The reaction mixture was stirred at room temperature overnight. After solvent evaporation under reduced pressure, the residue was dissolved in dichloromethane (300 mL) and washed successively with saturated citric acid solution, saturated sodium bicarbonate solution, and brine. The combined organic phases were concentrated under reduced pressure, and the residue was purified by silica gel column chromatography to yield compound **A1** as a yellow oil (35.30 g, 128.5 mmol, 94% yield).

Dimethyl (tert-butoxycarbonyl)-L-glutamate (**A1**): ^{1}H NMR (400 MHz, $CDCl_3$) δ 5.20 (d, J = 6.5 Hz, 1H), 4.24 (d, J = 3.9 Hz, 1H), 3.66 (s, 3H), 3.59 (s, 3H), 2.41 – 2.25 (m, 2H), 2.15 – 2.04 (m, 1H), 1.94 – 1.81 (m, 1H), 1.35 (s, 9H). ^{13}C NMR (101 MHz, $CDCl_3$) δ 173.11, 172.63, 155.35, 79.85, 52.56, 52.27, 52.15, 51.68, 30.00, 28.21, 27.62.

Synthesis of compound A2: **A1** (20.00 g, 72.70 mmol) was dissolved in anhydrous THF (300 mL) under argon atmosphere (three vacuum/argon cycles). The solution was cooled to -78°C and stirred for 10 min. LiHDMS solution in THF (160.0 mL, 1.00 mol/L, 160.00 mmol) was added dropwise while maintaining the low temperature. After stirring for 2 h, 3-bromopropionitrile (9.10 mL, 109.00 mmol) was added dropwise, and the reaction was continued for 1.5 h. The reaction was quenched with a mixture of

methanol (30 mL) and glacial acetic acid (30 mL), then warmed to room temperature and stirred overnight. The precipitate was removed by filtration, and the filtrate was concentrated under reduced pressure. The residue was dissolved in DCM (200 mL) and extracted successively with saturated citric acid solution, saturated sodium bicarbonate solution, and brine. The combined organic layers were concentrated under reduced pressure, and the residue was purified by silica gel column chromatography to afford **A2** as a yellow oil (14.30 g, 43.60 mmol, 60% yield).

Dimethyl (2S,4S)-2-((tert-butoxycarbonyl)amino)-4-(2-cyanoethyl)pentanedioate (**A2**): 1H NMR (400 MHz, $CDCl_3$) δ 5.07 (d, J = 8.4 Hz, 1H), 4.33 (dd, J = 13.7, 8.0 Hz, 1H), 3.69 (s, 3H), 3.67 (s, 3H), 2.62 – 2.53 (m, 1H), 2.35 (dd, J = 12.3, 7.3 Hz, 2H), 2.02 – 1.90 (m, 4H), 1.40 (s, 9H). 13C NMR (101 MHz, $CDCl_3$) δ 174.39, 172.34, 155.37, 118.71, 80.26, 52.54, 52.16, 51.54, 40.78, 34.37, 28.24, 27.29, 15.12.

Synthesis of compound A3: **A2** (12.00 g, 36.60 mmol) was dissolved in methanol (200 mL) and stirred in an ice bath for 30 min. Cobalt(II) chloride hexahydrate (6.15 g, 21.96 mmol) was added slowly, and the mixture was stirred for an additional 30 min. Sodium borohydride (8.50 g, 219.60 mmol) was then added portionwise. The reaction was maintained at 0°C for 24 h, warmed to room temperature, and stirred for further 24 h. After completion, the solvent was removed under reduced pressure. The residue was dissolved in DCM (100 mL) and washed successively with saturated citric acid solution, saturated sodium bicarbonate solution, and brine. The combined organic layers were concentrated under reduced pressure, and the residue was purified by silica gel column chromatography to afford **A3** as a yellow oil (5.70 g, 19.10 mmol, 52% yield).

Methyl (S)-2-((tert-butoxycarbonyl)amino)-3-((S)-2-oxopiperidin-3-yl)propanoate (**A3**): ^{1}H NMR (400 MHz, $CDCl_3$) δ 6.60 (s, 1H), 5.71 (d, *J* = 8.3 Hz, 1H), 4.35 – 4.26 (m, 1H), 3.72 (s, 3H), 3.31 (d, *J* = 3.1 Hz, 2H), 2.41 – 2.32 (m, 1H), 2.30 – 2.22 (m, 1H), 2.14 (d, *J* = 12.6 Hz, 1H), 1.93 – 1.81 (m, 2H), 1.75 (d, *J* = 8.6 Hz, 1H), 1.57 (dd, *J* = 16.8, 7.0 Hz, 1H), 1.43 (s, 9H). ^{13}C NMR (101 MHz, $CDCl_3$) δ 174.56, 173.22, 155.92, 79.75, 52.28, 51.74, 42.30, 38.06, 34.26, 28.29, 26.58, 21.57.

Synthesis of compound A4: **A3** (3.00 g, 10.00 mmol) was dissolved in DCM (100 mL). TFA (10.90 mL, 150.00 mmol) was added dropwise under ice-bath cooling, and the mixture was stirred at room temperature for 4 h. After removal of the solvent under reduced pressure, the resulting oily intermediate was dissolved in DCM (250 mL). The solution was adjusted to pH 7.5 by dropwise addition of triethylamine in an ice bath, followed by sequential addition of Boc-L-4-fluorophenylalanine (2.68 g, 10.00 mmol), EDCI (2.30 g, 12.00 mmol), HOBt (1.63 g, 12.00 mmol), and triethylamine (6.00 mL, 40.00 mmol). The reaction mixture was stirred at room temperature overnight. The

solution was washed successively with saturated citric acid solution, saturated sodium bicarbonate solution, and brine. The combined organic layers were concentrated under reduced pressure, and the residue was purified by silica gel column chromatography to afford **A4** as a white solid (3.11 g, 6.68 mmol, 67% yield).

Methyl (S)-2-((S)-2-((tert-butoxycarbonyl)amino)-3-(4-fluorophenyl)propanamido)-3-((S)-2-oxopiperidin-3-yl)propanoate (**A4**): ^{1}H NMR (400 MHz, DMSO-*d6*) δ 8.45 (d, *J* = 8.1 Hz, 1H), 7.45 (s, 1H), 7.36 – 7.23 (m, 2H), 7.07 (t, *J* = 8.6 Hz, 2H), 6.92 (d, *J* = 8.4 Hz, 1H), 4.41 (t, *J* = 9.5 Hz, 1H), 4.24 – 4.06 (m, 1H), 3.61 (s, 3H), 3.10 (s, 2H), 2.99 – 2.89 (m, 1H), 2.78 – 2.67 (m, 1H), 2.32 – 2.17 (m, 2H), 1.87 (d, *J* = 7.6 Hz, 1H), 1.69 (dd, *J* = 26.9, 12.6 Hz, 2H), 1.55 (s, 1H), 1.36 (d, *J* = 10.7 Hz, 1H), 1.29 (s, 9H). ^{13}C NMR (101 MHz, DMSO-*d6*) δ 172.99 (d, *J* = 11.1 Hz), 172.46, 162.64, 160.24, 155.74, 134.68, 131.49 (d, *J* = 7.8 Hz), 115.20, 114.99, 78.45, 56.23, 55.30, 52.33, 49.57, 41.64, 37.46, 36.85, 33.20, 28.52, 25.95, 21.89. ^{19}F NMR (376 MHz, DMSO-*d6*) δ -116.48.

Synthesis of compound A5: Following the synthetic procedure for compound **A4**, **A5** was obtained as a white solid (2.40 g, 4.25 mmol, 63% yield).

Methyl (6S,9S,12S)-9-(4-fluorobenzyl)-6-isopropyl-2,2-dimethyl-4,7,10-trioxo-12-(((S)-2-oxopiperidin-3-yl)methyl)-3-oxa-5,8,11-triazatridecan-13-oate (**A5**): ^{1}H NMR (400 MHz, DMSO-*d6*) δ 8.57 (d, *J* = 7.7 Hz, 1H), 7.94 (d, *J* = 8.0 Hz, 1H), 7.44 (s, 1H), 7.33 – 7.22 (m, 2H), 7.04 (t, *J* = 8.6 Hz, 2H), 6.59 (d, *J* = 9.0 Hz, 1H), 4.57 (d, *J* = 4.6 Hz, 1H), 4.36 (t, *J* = 7.0 Hz, 1H), 3.71 (t, *J* = 8.0 Hz, 1H), 3.59 (s, 3H), 3.09 (s, 2H), 2.94 (dd, *J* = 13.6, 4.1 Hz, 1H), 2.82 – 2.69 (m, 1H), 2.23 (t, *J* = 12.7 Hz, 1H), 2.14 (s, 1H), 1.89 – 1.76 (m, 2H), 1.73 – 1.60 (m, 2H), 1.52 (s, 1H), 1.36 (s, 9H), 1.23 (s, 1H), 0.70 (t, *J* = 6.9 Hz, 6H). ^{13}C NMR (101 MHz, DMSO-*d6*) δ 172.79 (d, *J* = 14.1 Hz), 171.73, 171.51, 160.25, 155.69, 134.08, 131.49 (d, *J* = 7.7 Hz), 115.35 – 115.21 (m), 115.09 (d, *J* = 21.1 Hz), 78.46, 60.17, 53.89, 52.35, 49.57, 41.62, 37.39 (d, *J* = 9.1 Hz), 33.11, 31.02, 28.60, 25.89, 21.83, 19.51, 18.60.

Synthesis of compound A6: **A5** (2.00 g, 3.66 mmol) was dissolved in methanol (100 mL). Sodium borohydride (2.10 g, 54.90 mmol) was added slowly under ice-bath cooling, and the mixture was stirred for 1 h. The reaction was then warmed to room temperature and stirred for an additional 3 h. After quenching with saturated ammonium chloride solution (25 mL), methanol was removed under reduced pressure. The residue was dissolved in DCM (100 mL) and washed with brine. The organic layer was concentrated under reduced pressure, and the residue was purified by silica gel column chromatography to afford **A6** as a white solid (1.70 g, 3.19 mmol, 87% yield).

Tert-butyl ((S)-1-(((S)-3-(4-fluorophenyl)-1-(((S)-1-hydroxy-3-((S)-2-oxopiperidin-3-yl)propan-2-yl)amino)-1-oxopropan-2-yl)amino)-3-methyl-1-oxobutan-2-yl)carbamate (**A6**): ^{1}H NMR (400 MHz, DMSO-*d6*) δ 7.92 (d, *J* = 8.1 Hz, 1H), 7.78 (d, *J* = 8.8 Hz, 1H), 7.31 (s, 1H), 7.25 (dd, *J* = 8.2, 5.8 Hz, 2H), 7.03 (t, *J* = 8.8 Hz, 2H), 6.58 (d, *J* = 9.0 Hz, 1H), 4.64 (s, 1H), 4.50 (dd, *J* = 14.0, 8.0 Hz, 1H), 3.83 – 3.69 (m, 2H), 3.20 (dd, *J* = 10.2, 4.7 Hz, 1H), 3.12 – 3.05 (m, 3H), 2.89 (dd, *J* = 13.7, 5.6 Hz, 1H), 2.78 (dd, *J* = 13.6, 8.8 Hz, 1H), 2.12 – 2.00 (m, 1H), 1.98 – 1.86 (m, 2H), 1.83 (dd, *J* = 13.5, 6.8 Hz, 1H), 1.68 (dd, *J* = 8.9, 4.4 Hz, 1H), 1.47 (dd, *J* = 17.4, 14.4 Hz, 2H), 1.36 (s, 9H), 1.31 – 1.24 (m, 2H), 0.77 – 0.70 (m, 6H). ^{13}C NMR (101 MHz, DMSO-*d6*) δ 173.73, 171.45, 171.13, 162.64, 160.23, 155.74, 134.14, 131.50 (d, *J* = 7.9 Hz), 115.16, 114.95, 78.48, 64.37, 60.16, 54.32, 47.96, 41.72, 37.57 (d, *J* = 16.3 Hz), 33.18, 31.02, 28.60, 26.23, 21.72, 19.52, 18.58.

Synthesis of compound A7: **A6** (1.20 g, 2.31 mmol) was dissolved in DCM (60 mL). Dess-Martin periodinane (1.96 g, 4.62 mmol) was added slowly under ice-bath cooling, and the mixture was stirred for 2 h. Upon completion, the reaction was quenched with saturated sodium bicarbonate solution (15 mL) and sodium thiosulfate (3.0 g). The mixture was washed with brine (3 × 50 mL), and the combined organic layers were concentrated under reduced pressure. The residue was purified by silica gel column chromatography to afford **A7** as a white solid (0.80 g, 1.50 mmol, 65% yield).

Tert-butyl ((S)-1-(((S)-3-(4-fluorophenyl)-1-oxo-1-(((S)-1-oxo-3-((S)-2-oxopiperidin-3-yl)propan-2-yl)amino)propan-2-yl)amino)-3-methyl-1-oxobutan-2-yl)carbamate (**A7**): ^{1}H NMR (400 MHz, $CDCl_3$) δ 9.28 (s, 1H), 8.30 (d, *J* = 5.1 Hz, 1H), 7.21 – 7.14 (m, 3H), 6.93 (t, *J* = 8.6 Hz, 2H), 6.68 (s, 1H), 5.11 (d, *J* = 8.4 Hz, 1H), 4.86 (dd, *J* = 14.9, 6.9 Hz, 1H), 4.34 – 4.24 (m, 1H), 3.96 – 3.84 (m, 1H), 3.26 (s, 2H), 3.06 (d, *J* = 6.8 Hz, 2H), 2.44 (s, 2H), 2.21 – 2.12 (m, 1H), 2.10 – 2.01 (m, 2H), 1.99 – 1.92 (m, 1H), 1.88 – 1.75 (m, 2H), 1.72 – 1.64 (m, 1H), 1.42 (s, 9H), 0.85 (dd, *J* = 20.4, 6.4 Hz, 6H). ^{13}C NMR (101 MHz, $CDCl_3$) δ 199.68, 174.93, 171.70, 163.11, 160.68, 155.95, 132.11, 131.04 (d, *J* = 7.8 Hz), 115.37, 115.16, 80.06, 60.07, 57.19, 54.00, 42.18, 37.70, 37.25, 30.73 (d, *J* = 7.5 Hz), 28.31, 27.17, 21.24, 19.20, 17.77.

Synthesis of compound A8: **A7** (0.80 g, 1.55 mmol) was dissolved in DCM (50 mL). Ethyl (triphenylphosphoranylidene)acetate (0.81 g, 2.33 mmol) was added, and the mixture was stirred at room temperature for 12 h. The reaction mixture was washed successively with saturated citric acid solution, saturated sodium bicarbonate solution, and brine. The combined organic layers were concentrated under reduced pressure. Purification by silica gel column chromatography afforded **A8** as a white solid (0.56 g, 0.93 mmol, 60% yield).

Ethyl (6S,9S,12S,E)-9-(4-fluorobenzyl)-6-isopropyl-2,2-dimethyl-4,7,10-trioxo-12-(((S)-2-oxopiperidin-3-yl)methyl)-3-oxa-5,8,11-triazapentadec-13-en-15-oate (**A8**): ^{1}H NMR (400 MHz, DMSO-*d6*) δ 8.25 (d, *J* = 8.5 Hz, 1H), 8.07 (d, *J* = 7.8 Hz, 1H), 7.36 (s, 1H), 7.29 – 7.20 (m, 2H), 7.02 (t, *J* = 8.6 Hz, 2H), 6.69 (dd, *J* = 15.7, 4.7 Hz, 1H), 6.59 (d, *J* = 8.9 Hz, 1H), 5.44 (d, *J* = 15.7 Hz, 1H), 4.58 – 4.46 (m, 2H), 4.10 (q, *J* = 7.0 Hz, 2H), 3.78 (t, *J* = 7.8 Hz, 1H), 3.08 (s, 2H), 2.84 (ddd, *J* = 27.4, 13.5, 7.8 Hz, 2H), 2.17 – 2.09 (m, 1H), 2.00 (t, *J* = 11.2 Hz, 1H), 1.91 – 1.81 (m, 2H), 1.68 (dd, *J* = 8.8, 4.1 Hz, 1H), 1.54 – 1.43 (m, 2H), 1.37 (s, 9H), 1.30 (s, 1H), 1.22 (t, *J* = 6.9 Hz, 3H), 0.76 (d, *J* = 6.4 Hz, 6H). ^{13}C NMR (101 MHz, DMSO-*d6*) δ 173.02, 171.49, 170.90, 166.03, 162.74, 160.33, 155.75, 149.73, 133.67, 131.43 (d, *J* = 7.8 Hz), 119.87, 115.25 (d, *J* = 21.1 Hz), 78.42, 60.32, 60.00, 54.54, 46.79, 41.69, 37.56, 35.71, 31.12, 28.62, 26.06, 21.72, 19.54, 18.63, 14.53.

Synthesis of compound A9: **A8** (2.00 g, 3.30 mmol) was dissolved in DCM (100 mL). TFA (2.46 mL, 33.00 mmol) was added, and the mixture was stirred at room temperature for 2 h. After removal of the solvent under reduced pressure, the residue was dissolved in ethyl acetate (100 mL). The solution was washed successively with saturated sodium bicarbonate solution and brine. The combined organic layers were concentrated under reduced pressure to afford **A9** as a white solid (1.30 g, 2.64 mmol, 80% yield) for further use.

Synthesis of compound B1: L-Hydroxyproline (10.00 g, 76.30 mmol) was dissolved in methanol (200 mL). Thionyl chloride (10.89 g, 91.56 mmol) was added dropwise under ice-bath cooling. After stirring for 10 min, the mixture was heated to reflux at 68°C for 3 h. The solvent was removed under reduced pressure to give a white solid intermediate. This intermediate was dissolved in DCM (500 mL), and the pH was adjusted to 7.5 by dropwise addition of triethylamine in an ice bath. Boc-L-tert-leucine (17.60 g, 76.30 mmol), EDCI (17.50 g, 91.50 mmol), HOBt (12.40 g, 91.50 mmol), and triethylamine (42.00 mL, 305.20 mmol) were added sequentially. The reaction mixture was stirred at room temperature overnight, then washed successively with saturated citric acid solution, saturated sodium bicarbonate solution, and brine. The combined organic layers were concentrated under reduced pressure. Purification by silica gel column chromatography afforded **B1** as a white solid (17.90 g, 49.94 mmol, 65% yield).

Methyl (2S,4R)-1-(2-((tert-butoxycarbonyl)amino)-3,3-dimethylbutanoyl)-4-hydroxypyrrolidine-2-carboxylate (**B1**): ^{1}H NMR (400 MHz, $CDCl_3$) δ 5.37 (d, *J* = 9.4 Hz, 1H), 4.61 (t, *J* = 8.5 Hz, 1H), 4.47 (s, 1H), 4.18 (d, *J* = 9.5 Hz, 1H), 3.91 (d, *J* = 11.1 Hz, 1H), 3.72 – 3.65 (m, 5H), 2.30 (dd, *J* = 13.2, 8.0 Hz, 1H), 1.95 (ddd, *J* = 13.3, 9.2, 4.3 Hz, 1H), 1.38 (s, 9H), 0.99 (s, 9H). ^{13}C NMR (101 MHz, $CDCl_3$) δ 172.64,

171.08, 156.08, 79.90, 69.95, 58.59, 57.80, 56.31, 52.12, 37.44, 35.72, 28.31, 26.22.

Synthesis of compound B2: **B1** (11.00 g, 30.69 mmol) was dissolved in THF (120 mL). Aqueous lithium hydroxide solution (1 mol/L, 46.00 mL, 46.04 mmol) was added under ice-bath cooling, and the mixture was stirred at room temperature for 4 h. After removal of THF under reduced pressure, the aqueous residue was slowly acidified to pH ≈6 by dropwise addition of 1 mol/L HCl. The solution was extracted with ethyl acetate (3 × 50 mL). The combined organic layers were concentrated under reduced pressure. Purification by silica gel column chromatography afforded **B2** as a white solid (7.40 g, 21.48 mmol, 70% yield).

(2S,4R)-1-(2-((tert-butoxycarbonyl)amino)-3,3-dimethylbutanoyl)-4-hydroxypyrrolidine-2-carboxylic acid (**B2**): ^{1}H NMR (400 MHz, DMSO-*d*6) δ 6.41 (d, *J* = 8.1 Hz, 1H), 4.28 (d, *J* = 23.1 Hz, 2H), 4.14 (d, *J* = 8.5 Hz, 1H), 3.59 (d, *J* = 19.7 Hz, 2H), 2.09 (s, 1H), 1.87 (s, 1H), 1.37 (s, 9H), 0.92 (s, 9H). ^{13}C NMR (101 MHz, DMSO-*d*6) δ 174.01, 170.30, 155.72, 78.56, 69.22, 58.50, 56.44, 37.80, 35.89, 28.63, 26.67.

Synthesis of compound B3: (S)-(-)-1-(4-Bromophenyl)ethylamine (10.00 g, 49.98 mmol) was dissolved in THF (250 mL). Di-tert-butyl dicarbonate (16.36 g, 74.97 mmol) was added slowly under ice-bath cooling. After stirring for 5 min, triethylamine (10.40 mL, 74.97 mmol) was added dropwise. The mixture was stirred at room temperature for 12 h. The solvent was removed under reduced pressure, and the residue was dissolved in DCM (300 mL). The solution was washed successively with saturated citric acid solution, saturated sodium bicarbonate solution, and brine. The combined organic layers were concentrated under reduced pressure. Purification by silica gel column chromatography afforded **B3** as a white solid (12.50 g, 41.64 mmol, 83% yield).

Tert-butyl (S)-(1-(4-bromophenyl)ethyl)carbamate (**B3**): ^{1}H NMR (400 MHz, DMSO-*d*6) δ 7.49 (d, *J* = 8.2 Hz, 2H), 7.41 (d, *J* = 6.8 Hz, 1H), 7.25 (d, *J* = 8.1 Hz, 2H), 4.58 (s, 1H), 1.35 (s, 9H), 1.27 (d, *J* = 6.9 Hz, 3H). ^{13}C NMR (101 MHz, DMSO-*d*6) δ 155.24, 145.45, 131.50, 128.56, 119.90, 78.24, 49.58, 28.70, 23.10.

Synthesis of compound B4: **B3** (10.00 g, 33.31 mmol) was dissolved in N,N-dimethylformamide (250 mL). Potassium acetate (7.93 g, 80.62 mmol) and palladium(II) acetate (0.15 g, 0.67 mmol) were added. The reaction system was purged with argon (three cycles) before adding 4-methylthiazole (6.60 g, 66.63 mmol). After stirring for 20 min, the mixture was heated at 90°C in an oil bath for 12 h. Upon completion, the solvent was removed by vacuum distillation. The residue was dissolved in ethyl acetate (150 mL) and washed with brine (3 × 50 mL). The combined organic

layers were concentrated under reduced pressure. Purification by silica gel column chromatography afforded **B4** as a white solid (6.37 g, 19.99 mmol, 60% yield).

Tert-butyl (S)-(1-(4-(4-methylthiazol-5-yl)phenyl)ethyl)carbamate (**B4**): ^{1}H NMR (400 MHz, DMSO-*d6*) δ 8.98 (s, 1H), 7.44 (d, *J* = 8.2 Hz, 2H), 7.38 (d, *J* = 7.9 Hz, 2H), 4.65 (s, 1H), 2.45 (s, 3H), 1.40 – 1.29 (m, 12H). ^{13}C NMR (101 MHz, DMSO-*d6*) δ 155.30, 151.90, 148.21, 145.82, 131.62, 130.12, 129.28, 126.83, 78.21, 49.75, 28.73, 23.24, 16.45.

Synthesis of compound B5: B5 was obtained as a white solid (6.60 g, 12.12 mmol, 77% yield) via an amide condensation reaction following the synthetic procedure for compound **A4**.

Tert-butyl ((S)-1-((2S,4R)-4-hydroxy-2-(((S)-1-(4-(4-methylthiazol-5-yl)phenyl)ethyl)carbamoyl)pyrrolidin-1-yl)-3,3-dimethyl-1-oxobutan-2-yl)carbamate (**B5**): ^{1}H NMR (400 MHz, DMSO-*d6*) δ 8.98 (s, 1H), 8.41 (s, 1H), 7.40 (d, *J* = 16.6 Hz, 4H), 6.41 (s, 1H), 5.13 (s, 1H), 4.90 (s, 1H), 4.46 (s, 1H), 4.29 (s, 1H), 4.15 (s, 1H), 3.59 (s, 2H), 2.45 (s, 3H), 2.03 (s, 1H), 1.79 (s, 1H), 1.38 (s, 12H), 0.93 (s, 9H). ^{13}C NMR (101 MHz, DMSO-*d6*) δ 171.06, 170.18, 155.79, 151.91, 148.21, 145.20, 131.58, 130.15, 129.28, 126.80, 78.56, 69.26, 58.94, 56.75, 48.21, 38.18, 35.83, 28.65, 26.79, 22.94, 16.45.

Synthesis of compounds B6a-B6f: **B6a-B6f** were obtained as white solids via an amide condensation reaction following the synthetic procedure for compound **A4**.

Methyl 8-(((S)-1-((2S,4R)-4-hydroxy-2-(((S)-1-(4-(4-methylthiazol-5-yl)phenyl)ethyl)carbamoyl)pyrrolidin-1-yl)-3,3-dimethyl-1-oxobutan-2-yl)amino)-8-oxooctanoate (**B6a**): ^{1}H NMR (400 MHz, DMSO-*d6*) δ 8.98 (s, 1H), 8.38 (d, *J* = 7.7 Hz, 1H), 7.80 (d, *J* = 9.2 Hz, 1H), 7.43 (d, *J* = 8.1 Hz, 2H), 7.38 (d, *J* = 8.2 Hz, 2H), 4.98 – 4.86 (m, 1H), 4.51 (d, *J* = 9.3 Hz, 1H), 4.42 (t, *J* = 8.0 Hz, 1H), 4.27 (s, 1H), 3.62 – 3.55 (m, 5H), 2.45 (s, 3H), 2.28 (t, *J* = 7.4 Hz, 2H), 2.25 – 2.19 (m, 1H), 2.11 (dd, *J* = 14.0, 6.9 Hz, 1H), 2.05 – 1.96 (m, 1H), 1.79 (ddd, *J* = 12.9, 8.5, 4.7 Hz, 1H), 1.48 (dd, *J* = 14.4, 7.1 Hz, 4H), 1.37 (d, *J* = 6.9 Hz, 3H), 1.23 (s, 4H), 0.93 (s, 9H). ^{13}C NMR (101 MHz, DMSO-*d6*) δ 173.84, 172.49, 171.10, 170.08, 151.96, 148.22, 145.14, 131.59, 130.15, 129.29, 126.85, 69.22, 59.01, 56.81, 51.64, 48.16, 38.19, 35.65, 35.29, 33.70, 28.70, 26.91, 25.71, 24.80, 22.90, 16.45.

Methyl 10-(((S)-1-((2S,4R)-4-hydroxy-2-(((S)-1-(4-(4-methylthiazol-5-yl)phenyl)ethyl)carbamoyl)pyrrolidin-1-yl)-3,3-dimethyl-1-oxobutan-2-yl)amino)-10-oxodecanoate (**B6b**): ^{1}H NMR (400 MHz, $CDCl_3$) δ 8.68 (s, 1H), 7.46 (d, *J* = 7.8 Hz, 1H), 7.37 (q, *J* = 8.3 Hz, 4H), 6.20 (d, *J* = 8.7 Hz, 1H), 5.07 (p, *J* = 6.9 Hz, 1H), 4.69

(t, *J* = 7.8 Hz, 1H), 4.56 (d, *J* = 8.7 Hz, 1H), 4.49 (s, 1H), 4.04 (d, *J* = 11.2 Hz, 1H), 3.67 – 3.57 (m, 5H), 2.51 (s, 3H), 2.49 – 2.42 (m, 1H), 2.28 (t, *J* = 7.5 Hz, 2H), 2.15 (t, *J* = 7.5 Hz, 2H), 2.04 (dd, *J* = 12.7, 8.4 Hz, 1H), 1.57 (d, *J* = 6.8 Hz, 4H), 1.46 (d, *J* = 6.9 Hz, 3H), 1.25 (d, *J* = 7.7 Hz, 8H), 1.03 (s, 9H). ^{13}C NMR (101 MHz, $CDCl_3$) δ 174.33, 173.67, 172.07, 169.75, 150.37, 148.41, 143.20, 131.61, 130.86, 129.56, 126.47, 69.93, 58.54, 57.45, 56.64, 51.47, 48.83, 36.47, 35.56, 35.14, 34.05, 31.92, 29.69, 29.59, 29.38, 29.38, 28.85, 26.52, 25.56, 24.88, 22.69, 22.24, 16.06.

Methyl 12-(((S)-1-((2S,4R)-4-hydroxy-2-(((S)-1-(4-(4-methylthiazol-5-yl)phenyl)ethyl)carbamoyl)pyrrolidin-1-yl)-3,3-dimethyl-1-oxobutan-2-yl)amino)-12-oxododecanoate (**B6c**): ^{1}H NMR (400 MHz, DMSO-*d*6) δ 8.97 (s, 1H), 8.36 (d, *J* = 7.7 Hz, 1H), 7.77 (d, *J* = 9.2 Hz, 1H), 7.43 (d, *J* = 7.9 Hz, 2H), 7.38 (d, *J* = 7.8 Hz, 2H), 5.09 (s, 1H), 4.99 – 4.86 (m, 1H), 4.52 (d, *J* = 9.2 Hz, 1H), 4.43 (t, *J* = 7.9 Hz, 1H), 4.28 (s, 1H), 3.60 (s, 2H), 3.57 (s, 3H), 2.45 (s, 3H), 2.27 (t, *J* = 7.4 Hz, 3H), 2.10 (dt, *J* = 14.1, 7.1 Hz, 1H), 2.05 – 1.97 (m, 1H), 1.85 – 1.75 (m, 1H), 1.49 (d, *J* = 6.6 Hz, 4H), 1.38 (d, *J* = 6.8 Hz, 3H), 1.23 (s, 12H), 0.93 (s, 9H). ^{13}C NMR (101 MHz, DMSO-*d*6) δ 173.82, 172.54, 171.09, 170.10, 151.90, 148.22, 145.11, 131.58, 130.17, 129.28, 126.85, 69.23, 59.02, 56.76, 51.60, 48.17, 38.18, 35.65, 35.37, 33.75, 29.63, 28.83, 26.91, 25.89, 24.90, 22.87, 16.44.

Methyl 2-(2-(2-(((S)-1-((2S,4R)-4-hydroxy-2-(((S)-1-(4-(4-methylthiazol-5-yl)phenyl)ethyl)carbamoyl)pyrrolidin-1-yl)-3,3-dimethyl-1-oxobutan-2-yl)amino)-2-oxoethoxy)ethoxy)acetate (**B6d**): ^{1}H NMR (400 MHz, DMSO-*d*6) δ 8.98 (s, 1H), 8.43 (d, *J* = 7.7 Hz, 1H), 7.41 (dt, *J* = 19.2, 6.4 Hz, 5H), 4.98 – 4.84 (m, 1H), 4.55 (d, *J* = 9.5 Hz, 1H), 4.44 (t, *J* = 8.1 Hz, 1H), 4.28 (s, 1H), 4.18 (d, *J* = 5.5 Hz, 2H), 3.97 (s, 2H), 3.69 – 3.57 (m, 9H), 2.45 (s, 3H), 2.05 (dd, *J* = 12.1, 8.4 Hz, 1H), 1.77 (ddd, *J* = 13.0, 8.8, 4.5 Hz, 1H), 1.37 (d, *J* = 7.0 Hz, 3H), 0.94 (s, 9H). ^{13}C NMR (101 MHz, DMSO-*d*6) δ 171.01 (d, *J* = 15.0 Hz), 169.48, 168.96, 151.95, 148.22, 145.19, 131.59, 130.16, 129.31, 126.81, 70.78, 70.32, 70.03, 69.25, 68.10, 59.03, 56.99, 56.20, 51.84, 48.22, 38.19, 36.20, 26.68, 22.93, 16.45.

Methyl (S)-13-((2S,4R)-4-hydroxy-2-(((S)-1-(4-(4-methylthiazol-5-yl)phenyl)ethyl)carbamoyl)pyrrolidine-1-carbonyl)-14,14-dimethyl-11-oxo-3,6,9-trioxa-12-azapentadecanoate (**B6e**): ^{1}H NMR (400 MHz, DMSO-*d*6) δ 8.98 (s, 1H), 8.43 (d, *J* = 7.5 Hz, 1H), 7.44 (d, *J* = 7.8 Hz, 2H), 7.37 (d, *J* = 8.1 Hz, 3H), 5.12 (s, 1H), 4.95 – 4.87 (m, 1H), 4.54 (d, *J* = 9.5 Hz, 1H), 4.44 (t, *J* = 8.1 Hz, 1H), 4.28 (s, 1H), 4.14 (s, 2H), 3.96 (s, 2H), 3.65 – 3.57 (m, 13H), 2.45 (s, 3H), 2.10 – 2.00 (m, 1H), 1.83 – 1.70 (m, 1H), 1.37 (d, *J* = 6.6 Hz, 3H), 0.94 (s, 9H). ^{13}C NMR (101 MHz, DMSO-*d*6) δ 171.01, 169.48, 168.98, 151.95, 148.23, 145.20, 131.58, 130.16, 129.31,

126.80, 70.88, 70.50, 70.42, 69.86, 69.24, 68.10, 59.02, 56.99, 56.16, 51.81, 48.22, 38.20, 36.21, 26.70, 22.94, 16.46.

Methyl 2-(4-(2-(((S)-1-((2S,4R)-4-hydroxy-2-(((S)-1-(4-(4-methylthiazol-5-yl)phenyl)ethyl)carbamoyl)pyrrolidin-1-yl)-3,3-dimethyl-1-oxobutan-2-yl)amino)-2-oxoethyl)phenyl)acetate (**B6f**): ^{1}H NMR (400 MHz, DMSO-*d6*) δ 8.98 (s, 1H), 8.41 (d, *J* = 7.8 Hz, 1H), 8.04 (d, *J* = 9.2 Hz, 1H), 7.41 (dd, *J* = 22.4, 8.3 Hz, 4H), 7.19 (dd, *J* = 18.0, 8.1 Hz, 4H), 5.11 (s, 1H), 4.92 (p, *J* = 7.0 Hz, 1H), 4.49 (d, *J* = 9.2 Hz, 1H), 4.43 (t, *J* = 8.1 Hz, 1H), 4.27 (s, 1H), 3.63 (s, 3H), 3.59 (d, *J* = 3.0 Hz, 4H), 3.45 (s, 1H), 3.42 (s, 1H), 2.45 (s, 3H), 2.01 (dd, *J* = 12.4, 7.9 Hz, 1H), 1.78 (ddd, *J* = 12.9, 8.6, 4.6 Hz, 1H), 1.38 (d, *J* = 7.0 Hz, 3H), 0.92 (s, 9H). ^{13}C NMR (101 MHz, DMSO-*d6*) δ 172.17, 171.09, 170.39, 169.89, 151.97, 148.23, 145.13, 135.62, 132.74, 131.59, 130.16, 129.58, 129.30, 126.85, 69.23, 59.05, 57.19, 57.07, 56.91, 52.14, 48.18, 41.88, 38.17, 35.88, 26.87, 22.93, 16.45.

Synthesis of compounds B7a-B7f: **B7a-B7f** were obtained as white solids following the synthetic procedure for compound **B2**.

8-(((S)-1-((2S,4R)-4-hydroxy-2-(((S)-1-(4-(4-methylthiazol-5-yl)phenyl)ethyl)carbamoyl)pyrrolidin-1-yl)-3,3-dimethyl-1-oxobutan-2-yl)amino)-8-oxooctanoic acid (**B7a**): ^{1}H NMR (400 MHz, DMSO-*d6*) δ 11.97 (s, 1H), 8.98 (s, 1H), 8.37 (d, *J* = 7.7 Hz, 1H), 7.79 (d, *J* = 9.3 Hz, 1H), 7.43 (d, *J* = 8.0 Hz, 2H), 7.37 (d, *J* = 8.0 Hz, 2H), 5.10 (s, 1H), 4.96 – 4.87 (m, 1H), 4.51 (d, *J* = 9.3 Hz, 1H), 4.42 (t, *J* = 8.0 Hz, 1H), 4.27 (s, 1H), 3.60 (s, 2H), 2.45 (s, 3H), 2.25 (dd, *J* = 14.2, 7.4 Hz, 1H), 2.18 (t, *J* = 7.4 Hz, 2H), 2.10 (dt, *J* = 14.0, 7.1 Hz, 1H), 2.04 – 1.96 (m, 1H), 1.79 (ddd, *J* = 12.6, 8.3, 4.5 Hz, 1H), 1.46 (d, *J* = 6.7 Hz, 4H), 1.37 (d, *J* = 6.9 Hz, 3H), 1.24 (d, *J* = 5.8 Hz, 4H), 0.93 (s, 9H). ^{13}C NMR (101 MHz, DMSO-*d6*) δ 174.96, 172.51, 171.10, 170.09, 151.94, 148.23, 145.13, 131.59, 130.16, 129.29, 126.85, 69.23, 59.01, 56.76, 48.16, 38.18, 35.65, 35.33, 34.09, 28.80, 26.91, 25.76, 24.87, 22.90, 16.45.

10-(((S)-1-((2S,4R)-4-hydroxy-2-(((S)-1-(4-(4-methylthiazol-5-yl)phenyl)ethyl)carbamoyl)pyrrolidin-1-yl)-3,3-dimethyl-1-oxobutan-2-yl)amino)-10-oxodecanoic acid (**B7b**): ^{1}H NMR (400 MHz, DMSO-*d6*) δ 11.95 (s, 1H), 8.98 (s, 1H), 8.36 (d, *J* = 7.7 Hz, 1H), 7.77 (d, *J* = 9.2 Hz, 1H), 7.40 (dd, *J* = 21.3, 7.8 Hz, 4H), 5.09 (s, 1H), 4.97 – 4.86 (m, 1H), 4.52 (d, *J* = 9.2 Hz, 1H), 4.42 (t, *J* = 7.9 Hz, 1H), 4.28 (s, 1H), 3.60 (s, 2H), 2.45 (s, 3H), 2.25 (dd, *J* = 14.2, 7.2 Hz, 1H), 2.18 (t, *J* = 7.2 Hz, 2H), 2.10 (dt, *J* = 13.6, 6.8 Hz, 1H), 2.04 – 1.97 (m, 1H), 1.85 – 1.75 (m, 1H), 1.47 (d, *J* = 6.1 Hz, 4H), 1.37 (d, *J* = 6.8 Hz, 3H), 1.23 (s, 8H), 0.93 (s, 9H). ^{13}C NMR (101 MHz, DMSO-*d6*) δ 174.95, 172.54, 171.10, 170.11, 151.91, 148.22,

145.12, 131.59, 130.17, 129.29, 126.85, 69.23, 59.02, 56.77, 48.17, 38.18, 35.65, 35.36, 34.15, 29.08, 26.91, 25.87, 24.96, 22.88, 16.44.

12-(((S)-1-((2S,4R)-4-hydroxy-2-(((S)-1-(4-(4-methylthiazol-5-yl)phenyl)ethyl)carbamoyl)pyrrolidin-1-yl)-3,3-dimethyl-1-oxobutan-2-yl)amino)-12-oxododecanoic acid (**B7c**): ^{1}H NMR (400 MHz, DMSO-*d*6) δ 11.96 (s, 1H), 8.97 (s, 1H), 8.37 (d, *J* = 7.4 Hz, 1H), 7.77 (d, *J* = 9.0 Hz, 1H), 7.40 (dd, *J* = 19.7, 7.6 Hz, 4H), 5.10 (s, 1H), 5.01 – 4.86 (m, 1H), 4.52 (d, *J* = 9.1 Hz, 1H), 4.43 (t, *J* = 7.7 Hz, 1H), 4.28 (s, 1H), 3.61 (s, 2H), 2.45 (s, 3H), 2.26 (dd, *J* = 14.0, 7.4 Hz, 1H), 2.18 (t, *J* = 7.0 Hz, 2H), 2.14 – 2.05 (m, 1H), 2.05 – 1.97 (m, 1H), 1.80 (s, 1H), 1.48 (s, 4H), 1.37 (d, *J* = 6.5 Hz, 3H), 1.23 (s, 12H), 0.93 (s, 9H). ^{13}C NMR (101 MHz, DMSO-*d*6) δ 174.96, 172.56, 171.10, 170.11, 151.89, 148.22, 145.11, 131.59, 130.17, 129.28, 126.85, 69.24, 59.03, 56.77, 48.18, 38.18, 35.65, 35.38, 34.14, 29.66, 28.88, 26.91, 25.90, 24.97, 22.87, 16.44.

2-(2-(2-(((S)-1-((2S,4R)-4-hydroxy-2-(((S)-1-(4-(4-methylthiazol-5-yl)phenyl)ethyl)carbamoyl)pyrrolidin-1-yl)-3,3-dimethyl-1-oxobutan-2-yl)amino)-2-oxoethoxy)ethoxy)acetic acid (**B7d**): ^{1}H NMR (400 MHz, $CDCl_3$) δ 8.63 (s, 1H), 8.28 (s, 1H), 7.44 (s, 1H), 7.35 (s, 4H), 5.08 (s, 1H), 4.70 – 4.61 (m, 2H), 4.38 (s, 1H), 3.99 (dd, *J* = 25.9, 11.8 Hz, 3H), 3.72 – 3.55 (m, 8H), 2.48 (s, 3H), 2.15 (s, 1H), 2.04 (d, *J* = 9.4 Hz, 1H), 1.47 (d, *J* = 4.8 Hz, 3H), 1.01 (s, 9H). ^{13}C NMR (101 MHz, DMSO-*d*6) δ 173.33, 170.98, 169.57, 169.39, 169.26, 151.96, 148.21, 145.22, 131.60, 130.14, 129.29, 126.82, 70.53, 69.82, 69.22, 59.06, 56.99, 56.53, 48.24, 38.16, 36.06, 26.73, 22.94, 16.45.

(S)-13-((2S,4R)-4-hydroxy-2-(((S)-1-(4-(4-methylthiazol-5-yl)phenyl)ethyl)carbamoyl)pyrrolidine-1-carbonyl)-14,14-dimethyl-11-oxo-3,6,9-trioxa-12-azapentadecanoic acid (**B7e**): ^{1}H NMR (400 MHz, DMSO-*d*6) δ 8.98 (s, 1H), 8.56 (d, *J* = 6.8 Hz, 1H), 7.85 (d, *J* = 8.3 Hz, 1H), 7.41 (dd, *J* = 19.1, 6.5 Hz, 4H), 4.96 – 4.87 (m, 1H), 4.52 (d, *J* = 8.4 Hz, 1H), 4.45 (t, *J* = 7.3 Hz, 1H), 4.29 (s, 1H), 4.01 (q, *J* = 14.7 Hz, 2H), 3.78 (d, *J* = 9.9 Hz, 1H), 3.66 (s, 2H), 3.62 – 3.53 (m, 9H), 2.45 (s, 3H), 2.06 – 1.99 (m, 1H), 1.79 – 1.72 (m, 1H), 1.38 (d, *J* = 5.4 Hz, 3H), 0.95 (s, 9H). ^{13}C NMR (101 MHz, DMSO-*d*6) δ 173.10, 171.11, 170.07, 169.65, 151.94, 148.20, 145.28, 131.61, 130.11, 129.27, 126.84, 71.27, 70.22, 70.22, 69.17, 69.05, 59.16, 57.74, 57.34, 57.05, 48.24, 38.07, 35.65, 26.77, 22.93, 16.45.

2-(4-(2-(((S)-1-((2S,4R)-4-hydroxy-2-(((S)-1-(4-(4-methylthiazol-5-yl)phenyl)ethyl)carbamoyl)pyrrolidin-1-yl)-3,3-dimethyl-1-oxobutan-2-yl)amino)-2-oxoethyl)phenyl)acetic acid (**B7f**): ^{1}H NMR (400 MHz, DMSO-*d*6) δ 12.27 (s, 1H), 8.98 (s, 1H), 8.41 (d, *J* = 7.7 Hz, 1H), 8.04 (d, *J* = 9.1 Hz, 1H), 7.40 (dd, *J* = 23.0, 8.0

Hz, 4H), 7.18 (dd, J = 16.7, 7.8 Hz, 4H), 4.98 – 4.85 (m, 1H), 4.49 (d, J = 9.1 Hz, 1H), 4.43 (t, J = 7.9 Hz, 1H), 4.26 (s, 1H), 3.60 (d, J = 14.1 Hz, 3H), 3.52 (s, 3H), 3.44 (s, 2H), 2.45 (s, 3H), 2.11 – 1.90 (m, 1H), 1.77 (ddd, J = 12.6, 8.4, 4.4 Hz, 1H), 1.37 (d, J = 6.9 Hz, 3H), 0.92 (s, 9H). ^{13}C NMR (101 MHz, DMSO-*d6*) δ 173.23, 170.40, 169.90, 151.96, 148.22, 145.13, 133.39, 131.59, 130.15, 129.98, 129.15, 126.85, 69.23, 59.47, 59.05, 57.02, 56.78, 55.38, 48.17, 41.89, 40.77, 38.16, 35.87, 29.47, 26.87, 22.93, 16.45.

Synthesis of compounds D1-D6: **D1-D6** were obtained as white solids following the synthetic procedure for compound **A4**.

Ethyl (3S,14S,17S,20S,E)-17-(4-fluorobenzyl)-3-((2S,4R)-4-hydroxy-2-(((S)-1-(4-(4-methylthiazol-5-yl)phenyl)ethyl)carbamoyl)pyrrolidine-1-carbonyl)-14-isopropyl-2,2-dimethyl-5,12,15,18-tetraoxo-20-(((S)-2-oxopiperidin-3-yl)methyl)-4,13,16,19-tetraazatricos-21-en-23-oate (**D1**): ^1H NMR (400 MHz, DMSO-*d6*) δ 8.97 (s, 1H), 8.36 (d, J = 7.8 Hz, 1H), 8.23 – 8.12 (m, 2H), 7.76 (dd, J = 8.8, 5.8 Hz, 2H), 7.39 (dt, J = 12.7, 6.3 Hz, 5H), 7.23 (dd, J = 8.4, 5.6 Hz, 2H), 7.02 (t, J = 8.8 Hz, 2H), 6.69 (dd, J = 15.7, 4.8 Hz, 1H), 5.42 (dd, J = 15.7, 1.3 Hz, 1H), 5.09 (d, J = 3.5 Hz, 1H), 5.01 – 4.86 (m, 1H), 4.59 – 4.39 (m, 4H), 4.29 (d, J = 2.3 Hz, 1H), 4.22 – 4.04 (m, 3H), 3.61 (s, 2H), 3.08 (s, 2H), 2.86 (ddd, J = 27.3, 13.6, 7.7 Hz, 2H), 2.45 (s, 3H), 2.29 – 1.95 (m, 8H), 1.94 – 1.76 (m, 3H), 1.69 (dd, J = 8.9, 4.4 Hz, 1H), 1.45 (dd, J = 7.0, 3.2 Hz, 6H), 1.37 (d, J = 7.1 Hz, 3H), 1.21 (t, J = 7.1 Hz, 7H), 0.94 (s, 9H), 0.79 (dd, J = 6.5, 4.4 Hz, 6H). ^{13}C NMR (101 MHz, DMSO-*d6*) δ 173.12 , 172.62 (d, J = 18.3 Hz), 171.51, 171.10, 170.88, 170.14 , 166.05, 162.73 , 160.33 , 151.91 , 149.81 , 148.23 , 145.11 , 133.77 , 131.73 – 131.23 (m), 130.18 , 129.29 , 126.85 , 119.83 , 115.37 , 115.17 , 69.25 , 60.33 , 59.04 , 57.96 , 56.78 (d, J = 8.5 Hz), 54.79 , 48.17, 46.72, 41.73, 38.19, 37.41 (d, J = 17.7 Hz), 35.72 (d, J = 11.4 Hz), 35.41, 31.11, 28.95, 26.92, 25.97 (d, J = 25.2 Hz), 25.82 – 25.66 (m), 22.89, 21.72, 19.56, 18.81, 16.44, 14.54. LC-MS: $C_{57}H_{80}FN_8O_{10}S$ $[M+H]^+$, calcd: 1087.56, found: 1087.38.

Ethyl (3S,16S,19S,22S,E)-19-(4-fluorobenzyl)-3-((2S,4R)-4-hydroxy-2-(((S)-1-(4-(4-methylthiazol-5-yl)phenyl)ethyl)carbamoyl)pyrrolidine-1-carbonyl)-16-isopropyl-2,2-dimethyl-5,14,17,20-tetraoxo-22-(((S)-2-oxopiperidin-3-yl)methyl)-4,15,18,21-tetraazapentacos-23-en-25-oate (**D2**): ^1H NMR (400 MHz, DMSO-*d6*) δ 8.98 (s, 1H), 8.36 (d, J = 7.7 Hz, 1H), 8.22 – 8.10 (m, 2H), 7.76 (dd, J = 8.6, 5.5 Hz, 2H), 7.39 (dt, J = 16.3, 8.1 Hz, 5H), 7.29 – 7.17 (m, 2H), 7.02 (t, J = 8.7 Hz, 2H), 6.69 (dd, J = 15.7, 4.7 Hz, 1H), 5.43 (d, J = 15.6 Hz, 1H), 5.02 – 4.85 (m, 1H), 4.58 – 4.39 (m, 4H), 4.28 (s, 1H), 4.11 (dt, J = 14.0, 7.5 Hz, 3H), 3.60 (s, 2H), 3.08 (s, 2H), 2.85 (ddd, J = 27.7, 13.6, 7.9 Hz, 2H), 2.45 (s, 3H), 2.30 – 1.95 (m, 8H), 1.94 – 1.76 (m, 3H), 1.69 (dd, J

= 8.3, 3.9 Hz, 1H), 1.46 (d, *J* = 7.0 Hz, 6H), 1.37 (d, *J* = 6.9 Hz, 3H), 1.29 (s, 1H), 1.22 (d, *J* = 6.3 Hz, 10H), 0.93 (s, 9H), 0.79 (dd, *J* = 6.2, 3.9 Hz, 6H). ^{13}C NMR (101 MHz, DMSO-*d6*) δ 173.14, 172.65 (d, *J* = 18.5 Hz), 171.50, 171.10, 170.89, 170.13, 166.05, 162.73, 160.32, 151.97, 149.78, 148.14, 145.13, 133.76, 131.91 – 131.10 (m), 130.13, 129.29, 126.86, 119.84, 115.37, 115.16, 69.24, 60.33, 59.04, 57.98, 56.78 (d, *J* = 10.5 Hz), 54.78, 48.18, 46.74, 41.73, 38.19, 37.41 (d, *J* = 18.6 Hz), 36.05 – 35.81 (m), 35.61 (dd, *J* = 20.6, 15.7 Hz), 31.09, 29.16 (d, *J* = 4.0 Hz), 26.91, 25.99 (d, *J* = 19.8 Hz), 22.87, 21.71, 19.55, 18.80, 16.40, 14.53. LC-MS: $C_{59}H_{84}FN_8O_{10}S$ [M+H]$^+$, calcd: 1115.59, found: 1115.46.

Ethyl (3S,18S,21S,24S,E)-21-(4-fluorobenzyl)-3-((2S,4R)-4-hydroxy-2-(((S)-1-(4-(4-methylthiazol-5-yl)phenyl)ethyl)carbamoyl)pyrrolidine-1-carbonyl)-18-isopropyl-2,2-dimethyl-5,16,19,22-tetraoxo-24-(((S)-2-oxopiperidin-3-yl)methyl)-4,17,20,23-tetraazaheptacos-25-en-27-oate (**D3**): ^{1}H NMR (400 MHz, DMSO-*d6*) δ 8.98 (s, 1H), 8.35 (d, *J* = 7.8 Hz, 1H), 8.15 (dd, *J* = 8.1, 5.4 Hz, 2H), 7.75 (dd, *J* = 8.8, 6.9 Hz, 2H), 7.39 (dt, *J* = 17.0, 8.5 Hz, 5H), 7.23 (dd, *J* = 8.5, 5.6 Hz, 2H), 7.02 (t, *J* = 8.8 Hz, 2H), 6.69 (dd, *J* = 15.7, 4.8 Hz, 1H), 5.43 (dd, *J* = 15.7, 1.5 Hz, 1H), 5.08 (d, *J* = 3.5 Hz, 1H), 4.99 – 4.86 (m, 1H), 4.47 (ddd, *J* = 22.1, 16.1, 8.7 Hz, 4H), 4.28 (s, 1H), 4.12 (dt, *J* = 14.1, 7.7 Hz, 3H), 3.61 (d, *J* = 3.7 Hz, 2H), 3.08 (s, 2H), 2.85 (ddd, *J* = 28.0, 13.6, 7.7 Hz, 2H), 2.45 (s, 3H), 2.31 – 1.95 (m, 8H), 1.93 – 1.78 (m, 3H), 1.69 (dd, *J* = 8.9, 4.4 Hz, 1H), 1.52 – 1.42 (m, 6H), 1.40 – 1.36 (m, 3H), 1.22 (t, *J* = 7.0 Hz, 15H), 0.93 (s, 9H), 0.79 (dd, *J* = 6.6, 4.3 Hz, 6H). ^{13}C NMR (101 MHz, DMSO-*d6*) δ 173.09, 172.61 (d, *J* = 17.0 Hz), 171.49, 171.09, 170.87, 170.11, 166.05, 160.32, 151.92, 149.81, 148.23, 145.12, 133.79, 131.86 – 131.10 (m), 130.17, 129.29, 126.85, 119.84, 115.37, 115.16, 69.23, 60.33, 59.02, 57.95, 56.76 (d, *J* = 9.7 Hz), 54.76, 48.17, 46.73, 41.72, 38.19, 37.42 (d, *J* = 17.8 Hz), 35.66, 35.38, 31.09, 29.70 – 28.86 (m), 26.91, 26.10, 25.87, 22.88, 21.72, 19.56, 18.79, 16.45, 14.54. LC-MS: $C_{61}H_{88}FN_8O_{10}S$ [M+H]$^+$, calcd: 1143.63, found: 1143.47.

Ethyl (3S,14S,17S,20S,E)-17-(4-fluorobenzyl)-3-((2S,4R)-4-hydroxy-2-(((S)-1-(4-(4-methylthiazol-5-yl)phenyl)ethyl)carbamoyl)pyrrolidine-1-carbonyl)-14-isopropyl-2,2-dimethyl-5,12,15,18-tetraoxo-20-(((S)-2-oxopiperidin-3-yl)methyl)-7,10-dioxa-4,13,16,19-tetraazatricos-21-en-23-oate (**D4**): ^{1}H NMR (400 MHz, DMSO-*d6*) δ 8.97 (s, 1H), 8.42 (d, *J* = 7.7 Hz, 1H), 8.37 (d, *J* = 7.6 Hz, 1H), 8.22 (d, *J* = 8.8 Hz, 1H), 7.45 (dd, *J* = 12.1, 8.7 Hz, 4H), 7.37 (d, *J* = 8.2 Hz, 3H), 7.23 (dd, *J* = 8.4, 5.7 Hz, 2H), 7.03 (t, *J* = 8.8 Hz, 2H), 6.69 (dd, *J* = 15.7, 4.8 Hz, 1H), 5.41 (dd, *J* = 15.7, 1.5 Hz, 1H), 5.13 (d, *J* = 3.5 Hz, 1H), 4.95 – 4.86 (m, 1H), 4.58 – 4.43 (m, 4H), 4.31 – 4.21 (m, 2H), 4.10 (q, *J* = 7.0 Hz, 2H), 3.98 (dd, *J* = 9.9, 5.8 Hz, 4H), 3.69 – 3.57 (m,

6H), 3.09 (s, 2H), 2.86 (ddd, *J* = 31.4, 13.5, 7.7 Hz, 2H), 2.45 (s, 3H), 2.16 (dt, *J* = 14.8, 8.4 Hz, 1H), 2.04 (dd, *J* = 17.4, 12.5 Hz, 2H), 1.98 – 1.89 (m, 2H), 1.89 – 1.83 (m, 1H), 1.78 (ddd, *J* = 12.9, 8.7, 4.5 Hz, 1H), 1.70 (dd, *J* = 8.9, 4.4 Hz, 1H), 1.50 – 1.42 (m, 2H), 1.37 (d, *J* = 7.0 Hz, 3H), 1.28 (d, *J* = 11.3 Hz, 1H), 1.21 (t, *J* = 7.1 Hz, 3H), 0.94 (s, 9H), 0.79 (dd, *J* = 11.1, 6.8 Hz, 6H). ^{13}C NMR (101 MHz, DMSO-*d*6) δ 173.12, 171.09 – 170.67 (m), 169.73 – 169.61 (m), 169.46 (d, *J* = 24.9 Hz), 169.03, 166.05, 162.75, 160.34, 151.92, 149.79, 148.23, 145.16, 133.70, 131.86 – 131.24 (m), 130.17, 129.30, 126.81, 119.82, 115.56 – 115.42 (m), 115.30 (d, *J* = 20.9 Hz), 71.11 – 70.27 (m), 70.27 – 69.73 (m), 69.26, 60.34, 59.06, 57.07 (d, *J* = 17.5 Hz), 56.29, 54.81, 48.24, 46.73, 41.71, 38.18, 37.43 (d, *J* = 16.9 Hz), 36.15, 35.70, 31.57, 26.71, 26.03, 22.90, 21.74, 19.52, 18.44, 16.44, 14.54. LC-MS: $C_{55}H_{76}FN_8O_{12}S$ $[M+H]^+$, calcd: 1091.52, found: 1091.46.

Ethyl (3S,17S,20S,23S,E)-20-(4-fluorobenzyl)-3-((2S,4R)-4-hydroxy-2-(((S)-1-(4-(4-methylthiazol-5-yl)phenyl)ethyl)carbamoyl)pyrrolidine-1-carbonyl)-17-isopropyl-2,2-dimethyl-5,15,18,21-tetraoxo-23-(((S)-2-oxopiperidin-3-yl)methyl)-7,10,13-trioxa-4,16,19,22-tetraazahexacos-24-en-26-oate (**D5**): ^{1}H NMR (400 MHz, DMSO-*d*6) δ 8.97 (s, 1H), 8.42 (d, *J* = 7.5 Hz, 1H), 8.38 (d, *J* = 7.4 Hz, 1H), 8.22 (d, *J* = 8.4 Hz, 1H), 7.40 (dd, *J* = 24.3, 8.4 Hz, 7H), 7.27 – 7.20 (m, 2H), 7.03 (t, *J* = 8.6 Hz, 2H), 6.69 (dd, *J* = 15.7, 4.1 Hz, 1H), 5.42 (d, *J* = 15.6 Hz, 1H), 5.13 (d, *J* = 2.9 Hz, 1H), 4.96 – 4.82 (m, 1H), 4.59 – 4.39 (m, 4H), 4.26 (dd, *J* = 15.6, 6.8 Hz, 2H), 4.09 (q, *J* = 6.9 Hz, 2H), 3.94 (d, *J* = 14.0 Hz, 4H), 3.61 (s, 10H), 3.09 (s, 2H), 2.85 (dt, *J* = 21.2, 13.4 Hz, 2H), 2.45 (s, 3H), 2.20 – 2.11 (m, 1H), 2.09 – 1.97 (m, 2H), 1.97 – 1.84 (m, 2H), 1.84 – 1.74 (m, 1H), 1.70 (d, *J* = 12.6 Hz, 1H), 1.46 (d, *J* = 7.1 Hz, 3H), 1.37 (d, *J* = 6.8 Hz, 3H), 1.28 (d, *J* = 11.3 Hz, 1H), 1.21 (t, *J* = 7.0 Hz, 3H), 0.94 (s, 9H), 0.79 (dd, *J* = 10.8, 6.8 Hz, 6H). ^{13}C NMR (101 MHz, DMSO-*d*6) δ 173.09, 170.88 (d, *J* = 10.4 Hz), 169.36, 169.01, 166.05, 160.34, 151.91, 149.78, 148.23, 145.16, 133.71, 131.68 – 131.23 (m), 130.17, 129.30, 126.81, 70.89 (d, *J* = 8.7 Hz), 70.12, 69.26, 60.33, 59.05, 57.07, 56.21, 54.80, 48.23, 46.73, 41.71, 38.34 – 38.20 (m), 38.20 – 37.18 (m), 36.20, 35.71, 31.61, 26.69, 26.04, 22.89, 21.75, 19.50, 18.42, 16.44, 14.53. LCMS: $C_{57}H_{80}FN_8O_{13}S$ $[M+H]^+$, calcd: 1135.55, found: 1135.41.

Ethyl (S,E)-4-((S)-3-(4-fluorophenyl)-2-((S)-2-(2-(4-(2-(((S)-1-((2S,4R)-4-hydroxy-2-(((S)-1-(4-(4-methylthiazol-5-yl)phenyl)ethyl)carbamoyl)pyrrolidin-1-yl)-3,3-dimethyl-1-oxobutan-2-yl)amino)-2-oxoethyl)phenyl)acetamido)-3-methylbutanamido)propanamido)-5-((S)-2-oxopiperidin-3-yl)pent-2-enoate (**D6**): ^{1}H NMR (400 MHz, DMSO-*d*6) δ 8.97 (s, 1H), 8.37 (d, *J* = 7.5 Hz, 1H), 8.23 (d, *J* = 6.8 Hz, 1H), 8.17 (d, *J* = 8.5 Hz, 1H), 8.02 (d, *J* = 8.4 Hz, 1H), 7.96 (d, *J* = 8.9 Hz, 1H),

7.41 (dd, J = 20.3, 7.7 Hz, 4H), 7.35 (s, 1H), 7.22 (d, J = 5.5 Hz, 3H), 7.17 (s, 3H), 7.01 (t, J = 8.3 Hz, 2H), 6.68 (dd, J = 15.7, 3.4 Hz, 1H), 5.41 (d, J = 15.6 Hz, 1H), 5.08 (s, 1H), 4.99 – 4.85 (m, 1H), 4.47 (dd, J = 16.1, 8.4 Hz, 4H), 4.27 (s, 1H), 4.18 – 4.07 (m, 3H), 3.59 (s, 3H), 3.51 (d, J = 13.9 Hz, 1H), 3.42 (s, 2H), 3.39 (s, 1H), 3.09 (s, 2H), 2.91 – 2.81 (m, 2H), 2.45 (s, 3H), 2.19 (d, J = 6.7 Hz, 1H), 2.01 (t, J = 10.5 Hz, 2H), 1.90 (d, J = 5.7 Hz, 2H), 1.83 – 1.75 (m, 1H), 1.69 (d, J = 10.5 Hz, 1H), 1.45 (d, J = 12.4 Hz, 2H), 1.38 (d, J = 6.7 Hz, 3H), 1.23 (d, J = 5.4 Hz, 4H), 0.91 (s, 9H), 0.78 (t, J = 5.8 Hz, 6H). ^{13}C NMR (101 MHz, DMSO-*d6*) δ 173.21, 171.35, 171.09, 170.77 (d, J = 18.3 Hz), 170.47, 169.91, 166.07, 162.73, 160.32, 151.95, 149.83, 148.21, 145.11, 134.98 (d, J = 12.1 Hz), 133.68, 132.00 – 131.10 (m), 130.15, 129.28, 126.84, 119.74, 115.40, 115.19, 69.23, 60.34, 59.05, 57.95, 56.89 (d, J = 21.6 Hz), 54.87, 48.17, 46.62, 42.68 – 41.31 (m), 38.14, 37.44, 37.21, 35.80 (d, J = 15.1 Hz), 31.38, 29.47, 26.84, 26.03, 22.90, 21.68, 19.50, 18.73, 16.42, 14.52. LC-MS: $C_{59}H_{76}FN_8O_{10}S$ [M+H]$^+$, calcd: 1107.53, found: 1107.42.

Synthesis of Compounds C1-C12: 2-(2,6-Dioxopiperidin-3-yl)-4-hydroxyisoindoline-1,3-dione (1.00 g, 3.65 mmol) was dissolved in N,N-dimethylformamide (20 mL). Tert-Butyl 5-bromovalerate (1.30 g, 5.49 mmol) and potassium carbonate (1.00 g, 7.24 mmol) were added. The reaction mixture was stirred at 80°C in an oil bath for 8 h. After completion, insoluble salts were removed by filtration. The filtrate was concentrated under reduced pressure. The residue was dissolved in dichloromethane (100 mL) and washed successively with saturated citric acid solution, saturated sodium bicarbonate solution, and brine. The combined organic layers were concentrated under reduced pressure. Purification by silica gel column chromatography afforded **C1** as a white solid (0.63 g, 1.46 mmol, 40% yield). Compounds **C2-C12** were synthesized following the procedure for **C1**.

Tert-butyl 5-((2-(2,6-dioxopiperidin-3-yl)-1,3-dioxoisoindolin-4-yl)oxy)pentanoate (**C1**): ^{1}H NMR (400 MHz, Chloroform-*d*) δ 8.72 (s, 1H), 7.65 (t, J = 7.8 Hz, 1H), 7.40 (d, J = 7.1 Hz, 1H), 7.23 (d, J = 8.6 Hz, 1H), 5.04 (dd, J = 12.4, 5.2 Hz, 1H), 4.24 (t, J = 6.6 Hz, 2H), 3.02 – 2.75 (m, 3H), 2.43 (t, J = 7.2 Hz, 2H), 2.05 (d, J = 6.0 Hz, 1H), 2.01 (t, J = 7.5 Hz, 2H), 1.67 (p, J = 7.6 Hz, 2H), 1.43(s, 9H). ^{13}C NMR (101 MHz, $CDCl_3$) δ 173.06, 171.63, 168.54, 167.24, 165.69, 156.58, 136.40, 133.53, 118.96, 117.03, 115.71, 80.23, 69.56, 49.13, 35.48, 31.35, 28.74, 28.12, 25.41, 22.58.

Tert-butyl 6-((2-(2,6-dioxopiperidin-3-yl)-1,3-dioxoisoindolin-4-yl)oxy)hexanoate (**C2**): ^{1}H NMR (400 MHz, Chloroform-*d*) δ 8.74 (s, 1H), 7.64 (t, J = 7.9 Hz, 1H), 7.41 (d, J = 7.2 Hz, 1H), 7.19 (d, J = 8.4 Hz, 1H), 4.96 (dd, J = 11.8, 5.4 Hz, 1H), 4.15 (t, J = 6.3 Hz, 2H), 2.95 – 2.68 (m, 3H), 2.24 (t, J = 7.4 Hz, 2H), 2.09 (d, J = 5.2 Hz, 1H),

1.88 (t, J = 7.3 Hz, 2H), 1.65 (p, J = 7.4 Hz, 2H), 1.53 (q, J = 8.1 Hz, 2H), 1.42 (s, 9H). ^{13}C NMR (101 MHz, $CDCl_3$) δ 173.07, 171.54, 168.50, 167.13, 165.70, 156.57, 136.51, 133.75, 118.90, 117.03, 115.71, 80.12, 69.08, 49.04, 35.35, 31.36, 28.61, 28.09, 25.29, 24.66, 22.58.

Tert-butyl 7-((2-(2,6-dioxopiperidin-3-yl)-1,3-dioxoisoindolin-4-yl)oxy)heptanoate (**C3**): ^{1}H NMR (400 MHz, Chloroform-*d*) δ 8.42 (s, 1H), 7.67 (dd, J = 8.5, 7.3 Hz, 1H), 7.45 (d, J = 7.2 Hz, 1H), 7.22 (d, J = 8.5 Hz, 1H), 4.97 (dd, J = 12.2, 5.3 Hz, 1H), 4.17 (t, J = 6.5 Hz, 2H), 2.94 – 2.72 (m, 3H), 2.23 (t, J = 7.4 Hz, 2H), 1.92 – 1.85 (m, 2H), 1.66 – 1.59 (m, 2H), 1.57 – 1.50 (m, 2H), 1.44 (s, 9H), 1.39 (dd, J = 16.0, 7.6 Hz, 2H). ^{13}C NMR (101 MHz, $CDCl_3$) δ 173.22, 171.22, 168.26, 167.13, 165.73, 156.68, 136.52, 133.80, 118.93, 117.09, 115.73, 80.05, 69.30, 49.06, 35.47, 31.39, 28.71, 28.11, 25.52, 24.93, 22.62.

Tert-butyl 8-((2-(2,6-dioxopiperidin-3-yl)-1,3-dioxoisoindolin-4-yl)oxy)octanoate (**C4**): ^{1}H NMR (400 MHz, Chloroform-*d*) δ 8.50 (s, 1H), 7.67 (dd, J = 8.5, 7.3 Hz, 1H), 7.44 (d, J = 7.2 Hz, 1H), 7.21 (d, J = 8.5 Hz, 1H), 4.97 (dd, J = 12.1, 5.4 Hz, 1H), 4.17 (t, J = 6.6 Hz, 2H), 2.93 – 2.72 (m, 3H), 2.21 (td, J = 7.6, 3.2 Hz, 2H), 2.15 – 2.09 (m, 1H), 1.88 (t, J = 7.4 Hz, 2H), 1.59 (t, J = 7.4 Hz, 2H), 1.50 (d, J = 7.8 Hz, 2H), 1.44 (s, 9H), 1.36 (tt, J = 10.4, 5.2 Hz, 4H). ^{13}C NMR (101 MHz, $CDCl_3$) δ 173.32, 171.29, 168.31, 167.13, 165.73, 156.69, 136.50, 133.79, 118.93, 117.07, 115.70, 79.99, 69.40, 49.06, 35.53, 31.38, 28.96, 28.81, 28.12, 25.67, 24.99, 22.62.

Tert-butyl 9-((2-(2,6-dioxopiperidin-3-yl)-1,3-dioxoisoindolin-4-yl)oxy)nonanoate (**C5**): ^{1}H NMR (400 MHz, Chloroform-*d*) δ 8.53 (s, 1H), 7.66 (dd, J = 8.5, 7.3 Hz, 1H), 7.43 (d, J = 7.2 Hz, 1H), 7.21 (d, J = 8.4 Hz, 1H), 4.97 (dd, J = 12.0, 5.3 Hz, 1H), 4.16 (t, J = 6.6 Hz, 2H), 2.94 – 2.71 (m, 3H), 2.20 (t, J = 7.5 Hz, 2H), 2.15 – 2.08 (m, 1H), 1.87 (p, J = 6.7 Hz, 2H), 1.58 (q, J = 7.3, 6.9 Hz, 2H), 1.49 (t, J = 7.4 Hz, 2H), 1.44 (s, 9H), 1.37 – 1.29 (m, 6H). ^{13}C NMR (101 MHz, $CDCl_3$) δ 173.36, 171.32, 168.33, 167.14, 165.73, 156.70, 136.49, 133.80, 118.92, 117.07, 115.68, 79.95, 69.41, 49.05, 35.58, 31.38, 29.15, 29.08, 28.99, 28.84, 28.12, 25.74, 25.04, 22.62.

Tert-butyl 10-((2-(2,6-dioxopiperidin-3-yl)-1,3-dioxoisoindolin-4-yl)oxy)decanoate (**C6**): ^{1}H NMR (400 MHz, Chloroform-*d*) δ 8.31 (s, 1H), 7.60 (dd, J = 8.5, 7.2 Hz, 1H), 7.37 (d, J = 7.3 Hz, 1H), 7.14 (d, J = 8.5 Hz, 1H), 4.90 (dd, J = 12.1, 5.4 Hz, 1H), 4.10 (t, J = 6.6 Hz, 2H), 2.86 – 2.66 (m, 3H), 2.13 (t, J = 7.5 Hz, 2H), 2.08 – 2.01 (m, 1H), 1.83 – 1.76 (m, 2H), 1.51 (d, J = 7.4 Hz, 2H), 1.42 (d, J = 7.7 Hz, 2H), 1.37 (s, 9H), 1.27 – 1.20 (m, 8H). ^{13}C NMR (101 MHz, $CDCl_3$) δ 173.44, 171.17, 168.22, 167.14, 165.71, 156.73, 136.49, 133.81, 118.90, 117.09, 115.67, 79.96, 69.43, 49.06, 35.61, 31.40, 29.30, 29.19, 29.06, 28.86, 28.12, 25.75, 25.09, 22.63.

Tert-butyl 2-(2-((2-(2,6-dioxopiperidin-3-yl)-1,3-dioxoisoindolin-4-yl)oxy)ethoxy)acetate (**C7**): ^{1}H NMR (400 MHz, Chloroform-*d*) δ 8.83 (s, 1H), 7.69 – 7.60 (m, 1H), 7.42 (d, *J* = 7.3 Hz, 1H), 7.26 (d, *J* = 8.5 Hz, 1H), 4.96 (dd, *J* = 11.3, 5.8 Hz, 1H), 4.37 (dd, *J* = 5.7, 3.8 Hz, 2H), 4.13 (s, 2H), 4.02 – 3.95 (m, 2H), 2.87 – 2.70 (m, 3H), 2.12 – 2.05 (m, 1H), 1.43 (s, 9H). ^{13}C NMR (101 MHz, $CDCl_3$) δ 171.61, 169.70, 168.51, 167.05, 165.63, 156.27, 136.54, 133.67, 119.49, 117.24, 116.17, 81.71, 69.57, 69.32, 69.13, 49.07, 31.35, 28.07, 22.55.

Tert-butyl 2-(2-(2-((2-(2,6-dioxopiperidin-3-yl)-1,3-dioxoisoindolin-4-yl)oxy)ethoxy)ethoxy)acetate (**C8**): ^{1}H NMR (400 MHz, DMSO-d_6) δ 11.11 (s, 1H), 7.81 (dd, *J* = 8.5, 7.2 Hz, 1H), 7.54 (d, *J* = 8.5 Hz, 1H), 7.46 (d, *J* = 7.2 Hz, 1H), 5.09 (dd, *J* = 12.8, 5.4 Hz, 1H), 4.41 – 4.31 (m, 2H), 3.98 (s, 2H), 3.87 – 3.77 (m, 2H), 3.67 (dd, *J* = 5.9, 3.3 Hz, 2H), 3.60 (dd, *J* = 5.8, 3.3 Hz, 2H), 2.89 (ddd, *J* = 17.3, 14.0, 5.3 Hz, 1H), 2.66 – 2.52 (m, 2H), 2.09 – 1.98 (m, 1H), 1.41 (s, 9H). ^{13}C NMR (101 MHz, DMSO) δ 173.27, 170.41, 169.85, 167.30, 165.75, 156.31, 137.47, 133.72, 120.50, 116.80, 115.88, 81.09, 70.54, 70.40, 69.35, 69.14, 68.56, 49.22, 31.42, 28.20, 22.47.

Tert-butyl 2-(2-(2-(2-((2-(2,6-dioxopiperidin-3-yl)-1,3-dioxoisoindolin-4-yl)oxy)ethoxy)ethoxy)ethoxy)acetate (**C9**): ^{1}H NMR (400 MHz, Chloroform-*d*) δ 8.58 (s, 1H), 7.27 (t, *J* = 7.7 Hz, 1H), 7.22 – 7.15 (m, 1H), 6.72 (d, *J* = 8.1 Hz, 1H), 5.12 (dd, *J* = 13.3, 5.1 Hz, 1H), 3.93 (s, 2H), 3.67 (t, *J* = 5.1 Hz, 2H), 3.61 (d, *J* = 3.2 Hz, 4H), 3.32 (t, *J* = 5.1 Hz, 2H), 2.78 – 2.67 (m, 2H), 2.23 (tt, *J* = 12.7, 6.6 Hz, 1H), 2.11 – 2.02 (m, 1H), 1.39 (s, 9H). ^{13}C NMR (101 MHz, $CDCl_3$) δ 171.58, 170.03, 169.97, 169.73, 143.06, 131.96, 129.63, 126.95, 113.32, 112.88, 81.73, 70.62, 70.52, 70.48, 70.23, 69.20, 68.93, 51.79, 45.16, 43.29, 31.55, 28.11, 23.38.

Tert-butyl 5-((2-((2-(2,6-dioxopiperidin-3-yl)-1,3-dioxoisoindolin-4-yl)oxy)ethyl)amino)-5-oxopentanoate (**C10**): ^{1}H NMR (400 MHz, DMSO-d_6) δ 11.13 (s, 1H), 8.14 (d, *J* = 6.1 Hz, 1H), 7.82 (t, *J* = 6.8 Hz, 1H), 7.54 (d, *J* = 11.1 Hz, 1H), 7.47 (d, *J* = 4.9 Hz, 1H), 5.09 (dd, *J* = 15.1, 4.9 Hz, 1H), 4.24 (s, 2H), 2.89 (t, *J* = 13.1 Hz, 1H), 2.62 (s, 1H), 2.40 (d, *J* = 6.0 Hz, 2H), 2.33 (d, *J* = 7.0 Hz, 2H), 2.09 – 1.97 (m, 1H), 1.37 (s, 9H), 1.24 (d, *J* = 5.9 Hz, 1H). ^{13}C NMR (101 MHz, DMSO) δ 173.31, 172.06, 171.90, 170.43, 167.30, 165.76, 156.19, 137.54, 133.72, 120.57, 116.97, 116.04, 80.03, 67.98, 49.20, 38.50, 31.40, 30.61, 30.42, 29.44, 28.18, 22.47.

Tert-butyl 7-((2-(2,6-dioxopiperidin-3-yl)-1,3-dioxoisoindolin-5-yl)oxy)heptanoate (**C11**): ^{1}H NMR (400 MHz, DMSO-d_6) δ 11.14 (s, 1H), 7.81 (d, *J* = 8.4 Hz, 1H), 7.39 (s, 1H), 7.33 (d, *J* = 8.2 Hz, 1H), 5.13 (d, *J* = 11.2 Hz, 1H), 4.26 – 4.06 (m, 2H), 2.99 – 2.81 (m, 1H), 2.68 – 2.53 (m, 2H), 2.26 – 2.15 (m, 2H), 2.06 (d, *J* = 13.8 Hz, 1H), 1.74

(t, J = 7.5 Hz, 2H), 1.51 (t, J = 7.7 Hz, 2H), 1.42 (s, 2H), 1.38 (s, 9H), 1.35 – 1.27 (m, 2H). ^{13}C NMR (101 MHz, DMSO) δ 173.26, 172.75, 170.40, 167.35, 167.28, 164.54, 134.38, 125.76, 123.30, 121.10, 109.25, 79.80, 69.15, 49.42, 35.12, 31.42, 28.65, 28.50, 28.19, 25.49, 24.97, 22.55.

Tert-butyl 8-((2-(2,6-dioxopiperidin-3-yl)-1,3-dioxoisoindolin-5-yl)oxy)octanoate (**C12**): ^{1}H NMR (400 MHz, DMSO-d_6) δ 11.13 (s, 1H), 7.83 (d, J = 8.3 Hz, 1H), 7.42 (s, 1H), 7.35 (d, J = 8.5 Hz, 1H), 5.12 (d, J = 12.8 Hz, 1H), 4.17 (d, J = 6.7 Hz, 2H), 2.89 (t, J = 13.8 Hz, 1H), 2.58 (t, J = 15.1 Hz, 2H), 2.18 (t, J = 7.9 Hz, 2H), 2.05 (d, J = 12.4 Hz, 1H), 1.75 (t, J = 7.4 Hz, 2H), 1.49 (t, J = 7.0 Hz, 4H), 1.38 (s, 9H), 1.28 (d, J = 16.6 Hz, 4H). ^{13}C NMR (101 MHz, DMSO) δ 173.29, 172.82, 170.44, 167.38, 167.32, 164.58, 134.42, 125.79, 123.31, 121.20, 109.28, 79.82, 69.22, 64.07, 49.41, 35.18, 31.40, 29.47, 28.75, 28.50, 28.21, 25.64, 24.98, 22.53.

Synthesis of Compounds C13-C14: 2-(2,6-Dioxopiperidin-3-yl)-4-fluoroisoindoline-1,3-dione (1.00 g, 3.62 mmol) was dissolved in N,N-dimethylformamide (20 mL). Tert-Butyl 7-aminoheptanoate (0.87 g, 4.34 mmol) and N,N-diisopropylethylamine (1.89 mL, 10.86 mmol) were added sequentially. The reaction mixture was stirred at 80°C in an oil bath for 16 h. After removal of the solvent under reduced pressure, the residue was dissolved in ethyl acetate (100 mL) and washed with brine (3 × 30 mL). The combined organic layers were concentrated under reduced pressure. Purification by silica gel column chromatography afforded **C13** as a white solid (0.42 g, 0.92 mmol, 25% yield). **C14** was synthesized following the procedure for **C13**.

Tert-butyl 7-((2-(2,6-dioxopiperidin-3-yl)-1,3-dioxoisoindolin-4-yl)amino)heptanoate (**C13**): ^{1}H NMR (400 MHz, Chloroform-d) δ 8.84 (s, 1H), 7.34 (t, J = 7.7 Hz, 1H), 7.21 (d, J = 7.4 Hz, 1H), 6.77 (d, J = 8.0 Hz, 1H), 5.16 (dd, J = 13.2, 5.1 Hz, 1H), 4.24 (d, J = 15.8 Hz, 1H), 4.10 (d, J = 15.9 Hz, 1H), 2.77 (dd, J = 11.0, 4.6 Hz, 2H), 2.21 (t, J = 7.5 Hz, 3H), 2.05 (dq, J = 9.0, 4.6 Hz, 1H), 1.61 (dt, J = 20.4, 7.1 Hz, 4H), 1.45 (s, 9H), 1.36 – 1.30 (m, 4H). ^{13}C NMR (101 MHz, $CDCl_3$) δ 173.31, 172.03, 170.27, 170.20, 143.33, 131.84, 129.69, 126.37, 112.89, 112.18, 80.02, 51.72, 45.18, 43.72, 35.53, 31.49, 29.33, 29.09, 29.02, 28.13, 26.98, 24.98, 23.20.

Tert-butyl 8-((2-(2,6-dioxopiperidin-3-yl)-1,3-dioxoisoindolin-4-yl)amino)octanoate (**C14**): ^{1}H NMR (400 MHz, DMSO-d_6) δ 10.89 (s, 1H), 8.58 (d, J = 7.6 Hz, 1H), 8.37 (s, 1H), 7.53 (d, J = 7.8 Hz, 1H), 7.48 – 7.34 (m, 2H), 4.74 (d, J = 7.9 Hz, 1H), 3.17 (q, J = 7.0 Hz, 2H), 2.85 – 2.71 (m, 1H), 2.57 (s, 1H), 2.18 (t, J = 7.3 Hz, 2H), 1.99 (d, J = 11.2 Hz, 2H), 1.55 – 1.44 (m, 4H), 1.40 (s, 9H), 1.30 – 1.25 (m, 4H). ^{13}C NMR (101 MHz, DMSO) δ 173.47, 172.84, 172.44, 166.45, 163.61, 160.12, 157.68, 136.70, 136.67, 130.83, 130.74, 126.19, 125.99, 123.95, 118.24, 118.01, 79.82, 49.92, 35.23,

31.27, 29.16, 28.90, 28.86, 28.22, 26.65, 25.05, 24.57.

Synthesis of Compounds E1-E14: C1 (0.63 g, 1.46 mmol) was dissolved in dichloromethane (10 mL). TFA (5 mL) was added, and the mixture was stirred at room temperature for 2 h. After removal of the solvent under reduced pressure, **E1** was obtained as a white solid (0.49 g, 1.31 mmol, 90% yield) for further use. **E2-E14** were synthesized following the procedure for **E1**.

Synthesis of Compounds D7-D20: D7-D20 were synthesized following the procedure for **D1**.

Ethyl (4S,E)-4-((2S)-2-((2S)-2-(5-((2-(2,6-dioxopiperidin-3-yl)-1,3-dioxoisoindolin-4-yl)oxy)pentanamido)-3-methylbutanamido)-3-(4-fluorophenyl)propanamido)-5-((S)-2-oxopiperidin-3-yl)pent-2-enoate (**D7**): ^{1}H NMR (400 MHz, DMSO-d_6) δ 11.11 (s, 1H), 8.19 (dd, *J* = 16.6, 8.2 Hz, 2H), 7.87 – 7.76 (m, 2H), 7.51 (d, *J* = 8.6 Hz, 1H), 7.44 (d, *J* = 7.2 Hz, 1H), 7.36 (s, 1H), 7.23 (dd, *J* = 8.6, 5.6 Hz, 2H), 7.03 (t, *J* = 8.9 Hz, 2H), 6.69 (dd, *J* = 15.7, 4.8 Hz, 1H), 5.40 (dd, *J* = 15.7, 1.7 Hz, 1H), 5.08 (dd, *J* = 12.8, 5.4 Hz, 1H), 4.47 (q, *J* = 10.6, 7.7 Hz, 2H), 4.25 – 4.14 (m, 3H), 4.10 (q, *J* = 7.0 Hz, 2H), 3.07 (p, *J* = 5.3, 4.3 Hz, 2H), 2.86 (qd, *J* = 13.7, 7.8 Hz, 3H), 2.64 – 2.55 (m, 1H), 2.31 – 2.14 (m, 3H), 2.01 (ddd, *J* = 16.6, 12.2, 4.5 Hz, 2H), 1.88 (dt, *J* = 13.3, 6.7 Hz, 2H), 1.71 (dq, *J* = 15.2, 4.5, 3.8 Hz, 5H), 1.44 (ddd, *J* = 14.1, 11.0, 3.6 Hz, 2H), 1.22 (t, *J* = 7.1 Hz, 4H), 0.84 – 0.75 (m, 6H). ^{13}C NMR (101 MHz, DMSO) δ 173.28, 173.13, 172.64, 171.49, 170.89, 170.43, 167.34, 166.08, 165.83, 156.44, 149.88, 137.52, 133.73, 131.48, 131.40, 120.22, 119.77, 116.68, 115.64, 115.40, 115.19, 68.87, 60.34, 58.00, 55.01, 49.17, 46.90, 41.79, 37.46, 35.74, 35.00, 31.42, 31.15, 28.37, 26.34, 22.51, 22.08, 21.57, 19.55, 18.81, 15.13. LC-MS: $C_{44}H_{53}FN_6O_{11}$ $[M+H]^+$, calcd: 861.38, found: 861.31.

Ethyl (4S,E)-4-((2S)-2-((2S)-2-(6-((2-(2,6-dioxopiperidin-3-yl)-1,3-dioxoisoindolin-4-yl)oxy)hexanamido)-3-methylbutanamido)-3-(4-fluorophenyl)propanamido)-5-((S)-2-oxopiperidin-3-yl)pent-2-enoate (**D8**): ^{1}H NMR (400 MHz, DMSO-d_6) δ 11.14 (s, 1H), 8.20 (t, *J* = 8.4 Hz, 2H), 7.90 – 7.75 (m, 2H), 7.50 (d, *J* = 8.5 Hz, 1H), 7.44 (d, *J* = 7.3 Hz, 1H), 7.38 (s, 1H), 7.22 (t, *J* = 6.2 Hz, 2H), 7.02 (t, *J* = 7.8 Hz, 2H), 6.69 (dd, *J* = 16.6, 3.8 Hz, 1H), 5.37 (d, *J* = 15.7 Hz, 1H), 5.08 (dd, *J* = 12.8, 5.3 Hz, 1H), 4.47 (dt, *J* = 15.4, 6.6 Hz, 2H), 4.13 (dq, *J* = 29.9, 8.6, 7.4 Hz, 5H), 3.07 (s, 2H), 2.86 (q, *J* = 12.9, 11.1 Hz, 3H), 2.59 (d, *J* = 18.6 Hz, 1H), 2.18 (dq, *J* = 13.8, 7.1, 6.5 Hz, 3H), 2.01 (dd, *J* = 12.6, 7.4 Hz, 2H), 1.87 (q, *J* = 6.0 Hz, 2H), 1.80 – 1.66 (m, 3H), 1.56 – 1.37 (m, 6H), 1.26 – 1.18 (m, 4H), 0.79 (d, *J* = 5.8 Hz, 6H). ^{13}C NMR (101 MHz, DMSO) δ 173.35, 173.17, 172.68, 171.52, 170.91, 170.48, 167.35, 166.10, 165.80, 160.30, 156.46, 137.56, 133.69, 131.48, 131.40, 120.20, 119.71, 116.60, 115.62,

115.41, 115.20, 69.11, 60.37, 57.94, 54.81, 49.17, 46.62, 41.71, 37.43, 37.25, 35.69, 35.47, 31.40, 31.13, 28.61, 26.00, 25.53, 25.38, 22.46, 21.67, 19.53, 18.81, 14.53. LC-MS: $C_{45}H_{55}FN_6O_{11}$ $[M+H]^+$, calcd: 875.39, found: 875.45.

Ethyl (4S,E)-4-((2S)-2-((2S)-2-(7-((2-(2,6-dioxopiperidin-3-yl)-1,3-dioxoisoindolin-4-yl)oxy)heptanamido)-3-methylbutanamido)-3-(4-fluorophenyl)propanamido)-5-((S)-2-oxopiperidin-3-yl)pent-2-enoate (**D9**): ^{1}H NMR (400 MHz, DMSO-d_6) δ 11.11 (s, 1H), 8.23 – 8.12 (m, 2H), 7.80 (q, *J* = 8.0 Hz, 2H), 7.51 (d, *J* = 8.6 Hz, 1H), 7.44 (d, *J* = 7.3 Hz, 1H), 7.35 (s, 1H), 7.23 (dd, *J* = 8.4, 5.5 Hz, 2H), 7.02 (t, *J* = 8.7 Hz, 2H), 6.69 (dd, *J* = 15.7, 4.8 Hz, 1H), 5.41 (d, *J* = 15.6 Hz, 1H), 5.08 (dd, *J* = 12.7, 5.4 Hz, 1H), 4.57 – 4.42 (m, 2H), 4.22 – 4.06 (m, 5H), 3.09 (s, 2H), 2.93 – 2.79 (m, 3H), 2.59 (d, *J* = 17.8 Hz, 1H), 2.16 (td, *J* = 14.6, 6.8 Hz, 3H), 2.02 (d, *J* = 12.9 Hz, 2H), 1.87 (t, *J* = 7.0 Hz, 2H), 1.74 (t, *J* = 7.3 Hz, 3H), 1.53 – 1.40 (m, 6H), 1.31 (d, *J* = 8.0 Hz, 3H), 1.22 (t, *J* = 7.1 Hz, 3H), 0.83 – 0.75 (m, 6H). ^{13}C NMR (101 MHz, DMSO) δ 173.31, 173.15, 172.72, 171.51, 170.88, 170.45, 167.36, 166.07, 165.81, 156.50, 149.85, 137.53, 133.72, 131.47, 131.39, 120.23, 119.79, 116.99, 115.62, 115.39, 115.18, 69.20, 60.35, 57.94, 54.76, 49.20, 46.68, 41.71, 37.47, 37.30, 35.73, 35.53, 31.42, 31.09, 28.74, 26.04, 25.79, 25.44, 22.50, 21.68, 19.54, 18.78, 14.53. LC-MS: $C_{46}H_{57}FN_6O_{11}$ $[M+H]^+$, calcd: 889.41, found: 889.40.

Ethyl (4S,E)-4-((2S)-2-((2S)-2-(8-((2-(2,6-dioxopiperidin-3-yl)-1,3-dioxoisoindolin-4-yl)oxy)octanamido)-3-methylbutanamido)-3-(4-fluorophenyl)propanamido)-5-((S)-2-oxopiperidin-3-yl)pent-2-enoate (**D10**): ^{1}H NMR (400 MHz, DMSO-d_6) δ 11.11 (s, 1H), 8.23 – 8.13 (m, 2H), 7.79 (q, *J* = 8.8, 8.3 Hz, 2H), 7.51 (d, *J* = 8.6 Hz, 1H), 7.44 (d, *J* = 7.2 Hz, 1H), 7.36 (s, 1H), 7.23 (dd, *J* = 8.3, 5.5 Hz, 2H), 7.02 (t, *J* = 8.6 Hz, 2H), 6.69 (dd, *J* = 15.7, 4.7 Hz, 1H), 5.42 (d, *J* = 15.8 Hz, 1H), 5.08 (dd, *J* = 12.8, 5.3 Hz, 1H), 4.49 (dt, *J* = 22.9, 9.3 Hz, 2H), 4.24 – 4.05 (m, 5H), 3.08 (t, *J* = 4.9 Hz, 2H), 2.95 – 2.78 (m, 3H), 2.59 (d, *J* = 18.2 Hz, 1H), 2.16 (ddt, *J* = 24.7, 16.7, 7.4 Hz, 3H), 2.07 – 1.97 (m, 2H), 1.88 (q, *J* = 6.7 Hz, 2H), 1.75 (t, *J* = 7.2 Hz, 3H), 1.48 (dt, *J* = 21.2, 6.1 Hz, 6H), 1.33 – 1.21 (m, 8H), 0.84 – 0.70 (m, 6H). ^{13}C NMR (101 MHz, DMSO) δ 173.29, 173.13, 172.72, 171.51, 170.89, 170.45, 167.34, 166.07, 165.80, 162.73, 160.32, 156.50, 149.86, 137.51, 133.72, 131.47, 131.39, 120.24, 119.78, 116.67, 115.60, 115.38, 115.17, 69.24, 60.35, 57.93, 54.79, 49.20, 46.68, 41.71, 37.47, 37.29, 35.73, 35.54, 31.42, 31.11, 29.05, 28.89, 26.05, 25.81, 25.75, 25.67, 24.90, 22.47, 21.69, 19.54, 18.79, 14.53. LC-MS: $C_{47}H_{59}FN_6O_{11}$ $[M+H]^+$, calcd: 903.43, found: 903.43.

Ethyl (4S,E)-4-((2S)-2-((2S)-2-(9-((2-(2,6-dioxopiperidin-3-yl)-1,3-dioxoisoindolin-4-yl)oxy)nonanamido)-3-methylbutanamido)-3-(4-fluorophenyl)propanamido)-5-((S)-

2-oxopiperidin-3-yl)pent-2-enoate (**D11**): ^{1}H NMR (400 MHz, DMSO-d_6) δ 11.11 (s, 1H), 8.17 (d, *J* = 8.2 Hz, 2H), 7.85 – 7.73 (m, 2H), 7.51 (d, *J* = 8.6 Hz, 1H), 7.44 (d, *J* = 7.2 Hz, 1H), 7.36 (s, 1H), 7.23 (dd, *J* = 8.5, 5.6 Hz, 2H), 7.02 (t, *J* = 8.8 Hz, 2H), 6.70 (dd, *J* = 15.7, 4.8 Hz, 1H), 5.42 (d, *J* = 15.8 Hz, 1H), 5.08 (dd, *J* = 12.8, 5.4 Hz, 1H), 4.47 (q, *J* = 9.9, 7.7 Hz, 2H), 4.24 – 4.06 (m, 5H), 3.15 – 3.01 (m, 2H), 2.95 – 2.78 (m, 3H), 2.63 – 2.56 (m, 1H), 2.21 – 2.08 (m, 3H), 2.06 – 1.98 (m, 2H), 1.88 (q, *J* = 6.7 Hz, 2H), 1.80 – 1.65 (m, 3H), 1.47 (ddd, *J* = 19.1, 15.4, 4.5 Hz, 8H), 1.23 (d, *J* = 7.1 Hz, 8H), 0.79 (dd, *J* = 6.7, 4.3 Hz, 6H). ^{13}C NMR (101 MHz, DMSO) δ 173.30, 173.13, 172.74, 171.52, 170.89, 170.46, 167.35, 166.07, 165.80, 162.75, 160.33, 156.51, 149.86, 137.51, 133.77, 133.72, 131.47, 131.39, 120.25, 119.79, 116.67, 115.60, 115.39, 115.18, 69.27, 60.35, 57.93, 54.78, 49.20, 46.68, 41.71, 37.48, 37.30, 35.74, 35.56, 31.42, 31.10, 29.19, 29.09, 29.01, 28.88, 28.58, 26.05, 25.83, 25.75, 24.95, 22.47, 21.69, 19.54, 18.79, 14.53. LC-MS: $C_{48}H_{61}FN_6O_{11}$ $[M+H]^+$, calcd: 917.44, found: 917.43.

Ethyl (4S,E)-4-((2S)-2-((2S)-2-(10-((2-(2,6-dioxopiperidin-3-yl)-1,3-dioxoisoindolin-4-yl)oxy)decanamido)-3-methylbutanamido)-3-(4-fluorophenyl)propanamido)-5-((S)-2-oxopiperidin-3-yl)pent-2-enoate (**D12**): ^{1}H NMR (400 MHz, DMSO-d_6) δ 11.11 (s, 1H), 8.17 (d, *J* = 8.3 Hz, 2H), 7.86 – 7.73 (m, 2H), 7.51 (d, *J* = 8.6 Hz, 1H), 7.44 (d, *J* = 7.3 Hz, 1H), 7.36 (s, 1H), 7.23 (dd, *J* = 8.5, 5.6 Hz, 2H), 7.02 (t, *J* = 8.6 Hz, 2H), 6.70 (dd, *J* = 15.7, 4.8 Hz, 1H), 5.42 (dd, *J* = 15.7, 1.6 Hz, 1H), 5.08 (dd, *J* = 12.7, 5.4 Hz, 1H), 4.47 (q, *J* = 7.6 Hz, 2H), 4.23 – 4.06 (m, 5H), 3.07 (d, *J* = 14.4 Hz, 2H), 2.94 – 2.79 (m, 3H), 2.60 (d, *J* = 18.2 Hz, 1H), 2.20 – 1.97 (m, 5H), 1.92 – 1.83 (m, 2H), 1.73 (dt, *J* = 18.8, 9.8 Hz, 3H), 1.45 (dd, *J* = 23.4, 12.0 Hz, 6H), 1.23 (dd, *J* = 14.9, 7.8 Hz, 12H), 0.79 (dd, *J* = 6.7, 4.0 Hz, 6H). ^{13}C NMR (101 MHz, DMSO) δ 173.30, 173.16, 172.77, 171.51, 170.89, 170.45, 167.35, 166.08, 165.80, 162.73, 160.32, 156.51, 149.83, 137.52, 133.75, 133.71, 131.47, 131.39, 120.24, 119.80, 116.67, 115.61, 115.38, 115.17, 69.26, 60.35, 57.95, 54.77, 49.19, 46.70, 41.71, 37.47, 37.29, 35.72, 35.57, 31.41, 31.07, 29.37, 29.18, 29.13, 29.08, 28.89, 26.04, 25.85, 25.73, 23.17, 21.68, 19.53, 18.78, 14.52. LC-MS: $C_{49}H_{63}FN_6O_{11}$ $[M+H]^+$, calcd: 931.46, found: 931.44.

Ethyl (7S,10S,13S,E)-1-((2-(2,6-dioxopiperidin-3-yl)-1,3-dioxoisoindolin-4-yl)oxy)-10-(4-fluorobenzyl)-7-isopropyl-5,8,11-trioxo-13-(((S)-2-oxopiperidin-3-yl)methyl)-3-oxa-6,9,12-triazahexadec-14-en-16-oate (**D13**): ^{1}H NMR (400 MHz, DMSO-d_6) δ 11.14 (s, 1H), 8.41 (d, *J* = 7.7 Hz, 1H), 8.24 (d, *J* = 8.6 Hz, 1H), 7.82 (t, *J* = 6.8 Hz, 1H), 7.55 (dd, *J* = 8.6, 2.4 Hz, 1H), 7.47 (d, *J* = 7.7 Hz, 2H), 7.39 (s, 1H), 7.23 (t, *J* = 6.0 Hz, 2H), 7.08 – 6.99 (m, 2H), 6.68 (dd, *J* = 15.7, 2.4 Hz, 1H), 5.37 (d, *J* = 15.7

Hz, 1H), 5.09 (dd, *J* = 12.1, 4.9 Hz, 1H), 4.47 (d, *J* = 7.9 Hz, 2H), 4.40 (s, 2H), 4.22 (t, *J* = 7.7 Hz, 1H), 4.13 – 4.04 (m, 4H), 3.87 (s, 2H), 3.07 (s, 2H), 2.93 – 2.76 (m, 3H), 2.61 (s, 1H), 2.13 (d, *J* = 9.3 Hz, 1H), 2.06 – 1.79 (m, 5H), 1.67 (s, 1H), 1.45 (d, *J* = 10.6 Hz, 2H), 1.29 – 1.20 (m, 5H), 0.76 (dd, *J* = 12.0, 5.6 Hz, 6H). ^{13}C NMR (101 MHz, DMSO) δ 173.47, 172.80, 171.07, 170.51, 169.25, 167.46, 165.88, 155.57, 150.22, 140.39, 135.37, 132.45, 122.14, 116.47, 115.38, 115.06, 70.91, 69.95, 68.99, 61.27, 57.65, 55.29, 48.89, 44.36, 37.02, 32.54, 26.62, 21.81, 19.46, 18.53, 15.15. LC-MS: $C_{43}H_{51}FN_6O_{12}$ [M+H]$^+$, calcd: 863.36, found: 863.32.

Ethyl (10S,13S,16S,E)-1-((2-(2,6-dioxopiperidin-3-yl)-1,3-dioxoisoindolin-4-yl)oxy)-13-(4-fluorobenzyl)-10-isopropyl-8,11,14-trioxo-16-(((S)-2-oxopiperidin-3-yl)methyl)-3,6-dioxa-9,12,15-triazanonadec-17-en-19-oate (**D14**): ^{1}H NMR (400 MHz, DMSO-*d*$_6$) δ 11.12 (s, 1H), 8.40 (d, *J* = 7.7 Hz, 1H), 8.23 (d, *J* = 8.8 Hz, 1H), 7.81 (dd, *J* = 8.6, 7.2 Hz, 1H), 7.52 (d, *J* = 8.5 Hz, 1H), 7.49 – 7.41 (m, 2H), 7.38 (s, 1H), 7.23 (dd, *J* = 8.6, 5.7 Hz, 2H), 7.02 (d, *J* = 8.8 Hz, 2H), 6.70 (dd, *J* = 15.7, 4.8 Hz, 1H), 5.40 (dd, *J* = 15.7, 1.7 Hz, 1H), 5.09 (dd, *J* = 12.8, 5.4 Hz, 1H), 4.49 (q, *J* = 7.8 Hz, 2H), 4.36 (t, *J* = 4.5 Hz, 2H), 4.24 (dd, *J* = 9.0, 6.8 Hz, 1H), 4.10 (q, *J* = 7.1, 6.7 Hz, 2H), 3.92 (s, 2H), 3.83 (q, *J* = 3.9 Hz, 2H), 3.71 (t, *J* = 4.6 Hz, 2H), 3.61 (q, *J* = 5.0, 4.5 Hz, 2H), 3.15 – 3.00 (m, 2H), 2.86 (ddt, *J* = 32.0, 13.4, 6.8 Hz, 3H), 2.64 – 2.56 (m, 1H), 2.20 – 2.12 (m, 1H), 2.06 – 1.98 (m, 2H), 1.93 – 1.83 (m, 2H), 1.69 (dt, *J* = 8.9, 4.6 Hz, 1H), 1.45 (ddd, *J* = 14.1, 10.7, 3.7 Hz, 2H), 1.22 (t, *J* = 7.1 Hz, 5H), 0.77 (dd, *J* = 9.7, 6.7 Hz, 6H). ^{13}C NMR (101 MHz, DMSO) δ 173.29, 173.09, 170.86, 170.81, 170.43, 169.38, 167.25, 166.06, 165.76, 160.41, 156.27, 150.47, 137.49, 134.31, 131.48, 131.40, 120.50, 119.77, 116.79, 115.89, 115.42, 115.21, 70.90, 70.47, 70.12, 69.38, 69.21, 60.35, 57.06, 54.57, 49.21, 46.93, 41.70, 37.51, 37.34, 31.62, 31.42, 26.00, 22.46, 21.73, 19.46, 18.43, 14.53. LC-MS: $C_{45}H_{55}FN_6O_{13}$ [M+H]$^+$, calcd: 907.38, found: 907.36.

Ethyl (13S,16S,19S,E)-1-((2-(2,6-dioxopiperidin-3-yl)-1,3-dioxoisoindolin-4-yl)oxy)-16-(4-fluorobenzyl)-13-isopropyl-11,14,17-trioxo-19-(((S)-2-oxopiperidin-3-yl)methyl)-3,6,9-trioxa-12,15,18-triazadocos-20-en-22-oate (**D15**): ^{1}H NMR (400 MHz, DMSO-*d*$_6$) δ 11.12 (s, 1H), 8.40 (d, *J* = 7.7 Hz, 1H), 8.23 (d, *J* = 8.8 Hz, 1H), 7.81 (dd, *J* = 8.5, 7.3 Hz, 1H), 7.53 (d, *J* = 8.5 Hz, 1H), 7.46 (d, *J* = 7.2 Hz, 1H), 7.41 (d, *J* = 9.0 Hz, 1H), 7.37 (s, 1H), 7.23 (dd, *J* = 8.6, 5.7 Hz, 2H), 7.03 (t, *J* = 8.9 Hz, 2H), 6.69 (dd, *J* = 15.7, 4.8 Hz, 1H), 5.40 (dd, *J* = 15.7, 1.7 Hz, 1H), 5.09 (dd, *J* = 12.7, 5.4 Hz, 1H), 4.49 (q, *J* = 7.6 Hz, 2H), 4.38 – 4.31 (m, 2H), 4.24 (dd, *J* = 9.0, 6.7 Hz, 1H), 4.10 (q, *J* = 7.0 Hz, 2H), 3.94 – 3.89 (m, 2H), 3.83 – 3.77 (m, 2H), 3.65 (t, *J* = 4.7 Hz, 2H), 3.57 (d, *J* = 2.2 Hz, 6H), 3.15 – 3.00 (m, 2H), 2.86 (ddt, *J* = 31.6, 13.4,

7.2 Hz, 3H), 2.64 – 2.56 (m, 1H), 2.15 (dp, *J* = 10.2, 4.6 Hz, 1H), 2.05 – 1.84 (m, 4H), 1.75 – 1.64 (m, 1H), 1.54 – 1.43 (m, 2H), 1.22 (t, *J* = 7.1 Hz, 5H), 0.78 (dd, *J* = 11.4, 6.7 Hz, 6H). ^{13}C NMR (101 MHz, DMSO) δ 173.29, 173.07, 170.85, 170.43, 169.39, 167.19, 166.06, 165.76, 159.65, 156.32, 149.88, 137.47, 133.72, 131.48, 120.48, 119.76, 116.78, 116.12, 115.42, 115.22, 70.79, 70.65, 70.31, 70.13, 70.04, 69.28, 69.15, 60.35, 57.04, 54.71, 48.59, 46.50, 41.43, 37.51, 37.36, 31.60, 31.42, 26.81, 25.78, 22.46, 21.87, 19.49, 18.43, 14.53. LC-MS: $C_{47}H_{59}FN_6O_{14}$ $[M+H]^+$, calcd: 951.41, found: 951.40.

Ethyl (10S,13S,16S,E)-1-((2-(2,6-dioxopiperidin-3-yl)-1,3-dioxoisoindolin-4-yl)oxy)-13-(4-fluorobenzyl)-10-isopropyl-4,8,11,14-tetraoxo-16-(((S)-2-oxopiperidin-3-yl)methyl)-3,9,12,15-tetraazanonadec-17-en-19-oate (**D16**): ^{1}H NMR (400 MHz, DMSO-*d*$_6$) δ 11.13 (s, 1H), 8.19 (d, *J* = 8.7 Hz, 2H), 7.96 (dd, *J* = 16.3, 8.3 Hz, 2H), 7.81 (t, *J* = 7.9 Hz, 1H), 7.50 (dd, *J* = 23.6, 7.9 Hz, 2H), 7.36 (s, 1H), 7.24 (dd, *J* = 8.2, 5.4 Hz, 2H), 7.03 (t, *J* = 8.6 Hz, 2H), 6.73 (dd, *J* = 15.7, 4.7 Hz, 1H), 5.51 (d, *J* = 15.6 Hz, 1H), 5.09 (dd, *J* = 12.6, 5.3 Hz, 1H), 4.55 (s, 1H), 4.43 (q, *J* = 7.9 Hz, 1H), 4.24 (t, *J* = 6.1 Hz, 2H), 4.16 – 4.01 (m, 3H), 3.54 – 3.41 (m, 2H), 3.15 – 2.81 (m, 5H), 2.65 – 2.52 (m, 2H), 2.42 (dq, *J* = 18.3, 10.1, 9.4 Hz, 4H), 2.15 (t, *J* = 5.3 Hz, 1H), 2.08 – 1.97 (m, 2H), 1.91 (dt, *J* = 13.3, 6.6 Hz, 2H), 1.69 (s, 1H), 1.56 – 1.42 (m, 2H), 1.29 (d, *J* = 10.9 Hz, 1H), 1.22 (t, *J* = 7.1 Hz, 3H), 0.75 (dd, *J* = 26.9, 6.7 Hz, 6H). ^{13}C NMR (101 MHz, DMSO) δ 173.29, 173.15, 172.91, 172.86, 171.46, 170.91, 170.43, 167.26, 166.12, 165.74, 162.69, 160.28, 156.14, 149.79, 137.51, 134.12, 133.73, 131.36, 131.29, 120.55, 119.91, 116.98, 116.05, 115.39, 115.18, 67.90, 60.34, 58.67, 55.03, 49.22, 46.78, 41.71, 38.56, 37.50, 36.77, 35.70, 31.41, 31.12, 30.98, 30.59, 26.02, 22.47, 21.71, 19.41, 18.23, 14.55. LC-MS: $C_{46}H_{56}FN_7O_{12}$ $[M+H]^+$, calcd: 918.40, found: 918.40.

Ethyl (4S,E)-4-((2S)-2-((2S)-2-(7-((2-(2,6-dioxopiperidin-3-yl)-1,3-dioxoisoindolin-5-yl)oxy)heptanamido)-3-methylbutanamido)-3-(4-fluorophenyl)propanamido)-5-((S)-2-oxopiperidin-3-yl)pent-2-enoate (**D17**): ^{1}H NMR (400 MHz, DMSO-*d*$_6$) δ 11.11 (s, 1H), 8.17 (d, *J* = 8.2 Hz, 2H), 7.80 (dd, *J* = 18.3, 8.5 Hz, 2H), 7.41 (d, *J* = 2.2 Hz, 1H), 7.34 (dd, *J* = 8.3, 2.3 Hz, 2H), 7.23 (dd, *J* = 8.4, 5.6 Hz, 2H), 7.02 (t, *J* = 8.7 Hz, 2H), 6.69 (dd, *J* = 15.7, 4.8 Hz, 1H), 5.42 (d, *J* = 16.0 Hz, 1H), 5.12 (dd, *J* = 12.9, 5.3 Hz, 1H), 4.47 (q, *J* = 7.7 Hz, 2H), 4.23 – 4.04 (m, 5H), 3.15 – 3.00 (m, 2H), 2.84 (tt, *J* = 13.5, 6.5 Hz, 3H), 2.60 (d, *J* = 17.8 Hz, 1H), 2.15 (dt, *J* = 15.7, 7.3 Hz, 3H), 2.08 – 1.96 (m, 2H), 1.88 (q, *J* = 6.6 Hz, 2H), 1.73 (t, *J* = 7.3 Hz, 3H), 1.54 – 1.38 (m, 6H), 1.35 – 1.27 (m, 3H), 1.22 (t, *J* = 7.0 Hz, 3H), 0.79 (dd, *J* = 6.7, 4.4 Hz, 6H). ^{13}C NMR (101 MHz, DMSO) δ 173.26, 173.09, 172.56, 171.50, 170.88, 170.42,

168.07, 167.31, 165.92, 164.58, 151.00, 134.43, 131.47, 131.39, 125.79, 122.70, 120.87, 119.80, 116.29, 115.17, 109.86, 68.76, 60.33, 57.89, 55.16, 51.17, 46.82, 41.44, 37.57, 35.93, 35.44, 31.42, 31.10, 29.39, 26.07, 25.75, 25.54, 22.54, 21.73, 19.55, 18.78, 15.10. LC-MS: $C_{46}H_{57}FN_6O_{11}$ $[M+H]^+$, calcd: 889.41, found: 889.42.

Ethyl (4S,E)-4-((2S)-2-((2S)-2-(8-((2-(2,6-dioxopiperidin-3-yl)-1,3-dioxoisoindolin-5-yl)oxy)octanamido)-3-methylbutanamido)-3-(4-fluorophenyl)propanamido)-5-((S)-2-oxopiperidin-3-yl)pent-2-enoate (**D18**): ^{1}H NMR (400 MHz, DMSO-d_6) δ 11.13 (s, 1H), 8.19 (dd, *J* = 8.3, 6.2 Hz, 2H), 7.81 (dd, *J* = 13.6, 8.5 Hz, 2H), 7.41 (d, *J* = 2.3 Hz, 1H), 7.39 – 7.31 (m, 2H), 7.23 (q, *J* = 4.7, 3.6 Hz, 2H), 7.02 (t, *J* = 8.8 Hz, 2H), 6.70 (dd, *J* = 15.7, 4.8 Hz, 1H), 5.41 (dd, *J* = 15.8, 1.7 Hz, 1H), 5.12 (dd, *J* = 12.9, 5.4 Hz, 1H), 4.47 (q, *J* = 7.7 Hz, 2H), 4.19 – 4.12 (m, 3H), 4.09 (t, *J* = 7.0 Hz, 2H), 3.07 (d, *J* = 8.5 Hz, 2H), 2.87 (dtt, *J* = 21.4, 13.5, 6.4 Hz, 3H), 2.65 – 2.57 (m, 1H), 2.21 – 1.96 (m, 5H), 1.87 (dt, *J* = 13.9, 7.0 Hz, 2H), 1.79 – 1.64 (m, 3H), 1.57 – 1.35 (m, 7H), 1.32 – 1.19 (m, 8H), 0.79 (dd, *J* = 6.7, 4.8 Hz, 6H). ^{13}C NMR (101 MHz, DMSO) δ 173.29, 173.11, 172.69, 171.54, 170.90, 170.44, 167.39, 167.31, 166.07, 164.58, 159.74, 149.88, 134.43, 133.87, 131.48, 131.39, 125.78, 123.32, 121.19, 119.77, 115.39, 115.18, 109.28, 69.25, 60.35, 57.90, 54.79, 50.02, 46.65, 41.71, 37.47, 37.30, 35.74, 35.52, 31.42, 31.13, 29.02, 28.89, 28.81, 26.05, 25.79, 25.73, 22.54, 21.70, 19.55, 18.80, 14.53. LC-MS: $C_{47}H_{59}FN_6O_{11}$ $[M+H]^+$, calcd: 903.43, found: 903.41.

Ethyl (4S,E)-4-((2S)-2-((2S)-2-(7-((2-(2,6-dioxopiperidin-3-yl)-1,3-dioxoisoindolin-4-yl)amino)heptanamido)-3-methylbutanamido)-3-(4-fluorophenyl)propanamido)-5-((S)-2-oxopiperidin-3-yl)pent-2-enoate (**D19**): ^{1}H NMR (400 MHz, DMSO-d_6) δ 10.88 (s, 1H), 8.57 (d, *J* = 8.3 Hz, 1H), 8.36 (t, *J* = 5.6 Hz, 1H), 8.19 (t, *J* = 9.2 Hz, 2H), 7.79 (d, *J* = 8.8 Hz, 1H), 7.56 – 7.48 (m, 1H), 7.44 (d, *J* = 7.5 Hz, 1H), 7.41 – 7.34 (m, 2H), 7.23 (dd, *J* = 8.5, 5.7 Hz, 2H), 7.03 (t, *J* = 8.8 Hz, 2H), 6.69 (dd, *J* = 15.7, 4.7 Hz, 1H), 5.39 (dd, *J* = 15.7, 1.7 Hz, 1H), 4.77 – 4.68 (m, 1H), 4.47 (dt, *J* = 15.4, 7.8 Hz, 2H), 4.19 – 4.05 (m, 3H), 3.20 – 3.12 (m, 2H), 3.11 – 3.01 (m, 2H), 2.92 – 2.72 (m, 3H), 2.56 (t, *J* = 3.8 Hz, 1H), 2.13 (dq, *J* = 25.9, 6.8 Hz, 3H), 2.03 – 1.93 (m, 3H), 1.86 (p, *J* = 6.8 Hz, 2H), 1.74 – 1.64 (m, 1H), 1.52 – 1.39 (m, 6H), 1.34 – 1.26 (m, 3H), 1.22 (t, *J* = 7.1 Hz, 5H), 0.79 (dd, *J* = 6.7, 4.8 Hz, 6H). ^{13}C NMR (101 MHz, DMSO) δ 173.49, 173.11, 171.53, 170.89, 166.45, 166.07, 163.44, 160.37, 149.92, 133.77, 131.49, 131.41, 119.73, 115.95, 115.20, 60.34, 57.87, 54.82, 50.52, 47.74, 42.41, 37.45, 35.74, 35.57, 31.17, 29.15, 28.89, 26.58, 26.04, 25.89, 24.59, 21.70, 19.55, 18.82, 15.42. LC-MS: $C_{46}H_{58}FN_7O_{10}$ $[M+H]^+$, calcd: 888.43, found: 888.40.

Ethyl (4S,E)-4-((2S)-2-((2S)-2-(8-((2-(2,6-dioxopiperidin-3-yl)-1,3-dioxoisoindolin-4-yl)amino)octanamido)-3-methylbutanamido)-3-(4-fluorophenyl)propanamido)-5-((S)-2-oxopiperidin-3-yl)pent-2-enoate (**D20**): ^{1}H NMR (400 MHz, DMSO-d_6) δ 10.87 (d, *J* = 11.3 Hz, 1H), 8.19 (t, *J* = 7.5 Hz, 2H), 7.78 (d, *J* = 8.7 Hz, 1H), 7.53 (dq, *J* = 13.7, 7.1 Hz, 2H), 7.45 – 7.34 (m, 3H), 7.24 (t, *J* = 7.0 Hz, 2H), 7.03 (t, *J* = 8.6 Hz, 2H), 6.70 (dd, *J* = 15.7, 4.7 Hz, 1H), 5.41 (d, *J* = 15.6 Hz, 1H), 4.74 (t, *J* = 7.9 Hz, 1H), 4.48 (dt, *J* = 15.0, 8.2 Hz, 2H), 4.12 (dq, *J* = 14.0, 7.4, 6.9 Hz, 3H), 3.23 – 3.03 (m, 4H), 2.92 – 2.69 (m, 3H), 2.58 (s, 1H), 2.22 – 1.94 (m, 6H), 1.88 (q, *J* = 6.6 Hz, 2H), 1.76 – 1.64 (m, 1H), 1.46 (q, *J* = 10.9, 6.7 Hz, 6H), 1.23 (dd, *J* = 15.0, 8.1 Hz, 10H), 0.79 (t, *J* = 5.7 Hz, 6H). ^{13}C NMR (101 MHz, DMSO) δ 173.50, 173.46, 173.13, 172.72, 172.43, 172.17, 171.52, 170.89, 166.07, 163.95, 163.59, 162.74, 160.33, 149.86, 133.75, 131.48, 131.40, 128.56, 126.21, 124.14, 123.06, 119.78, 115.39, 115.18, 110.84, 103.24, 60.34, 57.91, 54.81, 49.92, 47.47, 41.72, 37.46, 37.28, 35.74, 35.58, 31.27, 31.12, 30.97, 29.40, 29.24, 29.14, 29.00, 26.82, 26.76, 26.05, 25.85, 24.58, 21.69, 19.54, 18.80, 15.31. LC-MS: $C_{47}H_{60}FN_7O_{10}$ [M+H]$^+$, calcd: 902.44, found: 902.43.

Synthesis of Compounds 2a-2h: 2a-2h were synthesized following the procedure for **C13**.

Tert-butyl 1-(2-(2,6-dioxopiperidin-3-yl)-1,3-dioxoisoindolin-5-yl)piperidine-4-carboxylate (**2a**): ^{1}H NMR (400 MHz, Chloroform-*d*) δ 8.39 (s, 1H), 7.68 (d, *J* = 8.5 Hz, 1H), 7.28 (d, *J* = 3.4 Hz, 1H), 7.05 (dd, *J* = 8.6, 2.4 Hz, 1H), 4.95 (dd, *J* = 12.4, 5.4 Hz, 1H), 3.87 (dt, *J* = 13.3, 4.0 Hz, 2H), 3.08 (ddd, *J* = 13.6, 11.1, 3.0 Hz, 2H), 2.95 – 2.68 (m, 4H), 2.48 (td, *J* = 10.6, 5.3 Hz, 1H), 2.17 – 2.09 (m, 1H), 2.00 (dd, *J* = 13.5, 3.6 Hz, 2H), 1.83 (d, *J* = 3.9 Hz, 1H), 1.46 (s, 9H). ^{13}C NMR (101 MHz, $CDCl_3$) δ 173.56, 171.31, 168.48, 168.02, 167.95, 167.29, 155.83, 131.56, 124.36, 118.95, 118.00, 108.09, 80.76, 49.11, 46.59, 40.70, 32.22, 28.05, 27.43, 22.74.

Synthesis of Compounds 3b-3h: 3b-3h were synthesized following the procedure for **A7**.

Synthesis of Compounds 4b-4h: Tert-Butyl 4-piperidinecarboxylate (0.46 g, 2.48 mmol) was dissolved in N,N-dimethylformamide (50 mL). N,N-Diisopropylethylamine (1.18 mL, 6.75 mmol), compound 3b (0.80 g, 2.25 mmol), and sodium triacetoxyborohydride (0.72 g, 3.38 mmol) were added sequentially. The reaction mixture was stirred at room temperature overnight. After removal of the solvent under reduced pressure, the residue was dissolved in ethyl acetate (100 mL) and washed successively with saturated citric acid solution, saturated sodium bicarbonate solution, and brine. The combined organic layers were concentrated under reduced

pressure. Purification by silica gel column chromatography afforded **4b** as a yellow solid (0.64 g, 1.22 mmol, 54% yield). Compounds **4c-4h** were synthesized following the procedure for **4b**.

Tert-butyl 1'-(2-(2,6-dioxopiperidin-3-yl)-1,3-dioxoisoindolin-5-yl)-[1,4'-bipiperidine]-4-carboxylate (**4b**): ^{1}H NMR (400 MHz, DMSO-d_6) δ 11.08 (s, 1H), 7.55 (dd, *J* = 8.6, 6.9 Hz, 1H), 7.11 (t, *J* = 8.8 Hz, 2H), 5.06 (dd, *J* = 12.8, 5.4 Hz, 1H), 3.62 – 3.50 (m, 4H), 3.31 (dd, *J* = 10.6, 7.2 Hz, 1H), 2.92 – 2.74 (m, 3H), 2.58 (d, *J* = 17.1 Hz, 1H), 2.30 (d, *J* = 7.4 Hz, 2H), 2.20 – 2.11 (m, 1H), 2.08 – 1.91 (m, 4H), 1.74 (d, *J* = 12.7 Hz, 2H), 1.69 – 1.61 (m, 1H), 1.54 (t, *J* = 11.7 Hz, 2H), 1.39 (s, 9H). ^{13}C NMR (101 MHz, DMSO) δ 174.37, 173.34, 169.87, 167.82, 167.02, 163.03, 146.42, 135.29, 134.22, 120.11, 111.31, 110.19, 78.35, 61.73, 56.11, 53.32, 53.06, 48.82, 41.73, 34.94, 28.17.

Tert-butyl 1-((1-(2-(2,6-dioxopiperidin-3-yl)-1,3-dioxoisoindolin-5-yl)piperidin-4-yl)methyl)piperidine-4-carboxylate (**4c**): ^{1}H NMR (400 MHz, DMSO-d_6) δ 11.08 (s, 1H), 7.65 (d, *J* = 9.3 Hz, 1H), 7.30 (s, 1H), 7.23 (d, *J* = 8.8 Hz, 1H), 5.07 (dd, *J* = 13.3, 5.2 Hz, 1H), 4.03 (d, *J* = 12.5 Hz, 2H), 2.92 (dt, *J* = 26.3, 13.9 Hz, 3H), 2.75 (d, *J* = 11.4 Hz, 2H), 2.56 (d, *J* = 7.5 Hz, 1H), 2.12 (t, *J* = 10.1 Hz, 3H), 2.06 – 1.98 (m, 1H), 1.91 (d, *J* = 11.0 Hz, 2H), 1.76 (t, *J* = 11.3 Hz, 5H), 1.53 (t, *J* = 10.6 Hz, 2H), 1.40 (s, 9H), 1.12 (d, *J* = 12.1 Hz, 2H).

Tert-butyl 1-(2-(1-(2-(2,6-dioxopiperidin-3-yl)-1,3-dioxoisoindolin-5-yl)piperidin-4-yl)ethyl)piperidine-4-carboxylate (**4d**): ^{1}H NMR (400 MHz, DMSO-d_6) δ 11.07 (s, 1H), 7.64 (d, *J* = 8.5 Hz, 1H), 7.30 (d, *J* = 2.3 Hz, 1H), 7.22 (dd, *J* = 8.7, 2.3 Hz, 1H), 5.06 (dd, *J* = 12.9, 5.4 Hz, 1H), 4.03 (d, *J* = 12.7 Hz, 2H), 2.90 (dd, *J* = 37.4, 12.8 Hz, 5H), 2.56 (q, *J* = 4.5, 3.9 Hz, 1H), 2.39 (d, *J* = 7.6 Hz, 2H), 2.25 – 2.15 (m, 1H), 2.13 – 1.97 (m, 3H), 1.91 (s, 3H), 1.74 (d, *J* = 11.6 Hz, 4H), 1.56 (t, *J* = 11.9 Hz, 3H), 1.39 (s, 9H), 1.17 (d, *J* = 10.2 Hz, 2H). ^{13}C NMR (101 MHz, DMSO) δ 174.10, 173.29, 172.51, 170.59, 168.12, 167.44, 155.41, 134.53, 125.47, 118.04, 117.81, 108.16, 79.98, 55.66, 52.60, 49.19, 47.89, 41.36, 33.92, 32.86, 31.53, 31.45, 28.17, 28.05, 22.66, 21.54.

Tert-butyl 1-(((3S)-1-(2-(2,6-dioxopiperidin-3-yl)-1,3-dioxoisoindolin-5-yl)pyrrolidin-3-yl)methyl)piperidine-4-carboxylate (**4e**): ^{1}H NMR (400 MHz, Chloroform-*d*) δ 8.36 (s, 1H), 7.65 (d, *J* = 8.4 Hz, 1H), 6.94 (d, *J* = 2.0 Hz, 1H), 6.68 (dd, *J* = 8.5, 2.2 Hz, 1H), 4.94 (dd, *J* = 12.0, 5.3 Hz, 1H), 3.55 (dd, *J* = 10.1, 7.3 Hz, 1H), 3.48 (td, *J* = 9.0, 8.1, 4.1 Hz, 1H), 3.44 – 3.36 (m, 1H), 3.16 (dd, *J* = 10.1, 7.0 Hz, 1H), 2.93 – 2.73 (m, 5H), 2.61 (p, *J* = 7.3 Hz, 1H), 2.38 (t, *J* = 7.9 Hz, 2H), 2.23 – 2.00 (m, 5H), 1.88 – 1.76 (m, 5H), 1.45 (s, 9H). ^{13}C NMR (101 MHz, $CDCl_3$) δ

174.52, 171.31, 168.58, 168.28, 167.68, 152.14, 134.46, 125.44, 116.39, 115.09, 106.16, 80.13, 61.78, 53.75, 53.14, 52.72, 49.02, 47.54, 42.00, 36.41, 31.47, 29.81, 28.26, 28.09, 22.81.

Tert-butyl 1-(((3R)-1-(2-(2,6-dioxopiperidin-3-yl)-1,3-dioxoisoindolin-5-yl)pyrrolidin-3-yl)methyl)piperidine-4-carboxylate (**4f**): ^{1}H NMR (400 MHz, DMSO-d_6) δ 11.08 (s, 1H), 7.63 (d, *J* = 8.4 Hz, 1H), 6.89 (d, *J* = 2.2 Hz, 1H), 6.80 (dd, *J* = 8.5, 2.1 Hz, 1H), 5.06 (dd, *J* = 12.9, 5.4 Hz, 1H), 3.57 – 3.36 (m, 6H), 3.11 (dd, *J* = 10.4, 6.9 Hz, 1H), 2.92 – 2.73 (m, 3H), 2.61 – 2.54 (m, 2H), 2.33 (d, *J* = 7.4 Hz, 2H), 2.21 – 2.07 (m, 2H), 2.00 (dd, *J* = 12.1, 6.2 Hz, 2H), 1.78 – 1.71 (m, 2H), 1.56 (t, *J* = 12.6 Hz, 2H), 1.40 (s, 9H). ^{13}C NMR (101 MHz, DMSO) δ 174.31, 173.32, 170.66, 168.20, 167.72, 152.33, 134.48, 125.42, 115.91, 115.70, 105.91, 79.87, 61.48, 53.36, 52.92, 52.61, 49.13, 47.66, 41.69, 36.10, 31.46, 29.66, 28.53, 28.18, 22.72.

Tert-butyl 1-((1-(2-(2,6-dioxopiperidin-3-yl)-1,3-dioxoisoindolin-5-yl)azetidin-3-yl)methyl)piperidine-4-carboxylate (**4g**): ^{1}H NMR (400 MHz, Chloroform-*d*) δ 8.40 (s, 1H), 7.63 (d, *J* = 8.3 Hz, 1H), 6.78 (d, *J* = 2.1 Hz, 1H), 6.52 (dd, *J* = 8.3, 2.2 Hz, 1H), 4.92 (dd, *J* = 12.2, 5.3 Hz, 1H), 4.09 (t, *J* = 7.5 Hz, 2H), 3.90 (dd, *J* = 8.2, 5.3 Hz, 2H), 3.35 (dq, *J* = 12.3, 6.6, 6.0 Hz, 1H), 2.87 – 2.70 (m, 5H), 2.25 (td, *J* = 10.8, 5.4 Hz, 1H), 2.11 (ddd, *J* = 9.9, 6.1, 2.4 Hz, 1H), 2.06 – 1.98 (m, 2H), 1.93 (dd, *J* = 13.1, 3.6 Hz, 2H), 1.82 – 1.71 (m, 2H), 1.44 (s, 9H). ^{13}C NMR (101 MHz, $CDCl_3$) δ 174.02, 171.24, 168.48, 167.88, 167.49, 154.96, 134.25, 125.27, 118.02, 114.17, 105.10, 80.36, 55.41, 54.91, 49.38, 49.06, 41.60, 31.44, 29.69, 28.07, 27.69, 22.77.

Tert-butyl 1-(1-(2-(2,6-dioxopiperidin-3-yl)-1,3-dioxoisoindolin-5-yl)azetidin-3-yl)piperidine-4-carboxylate (**4h**): ^{1}H NMR (400 MHz, Chloroform-*d*) δ 9.85 (s, 1H), 7.63 (d, *J* = 8.3 Hz, 1H), 6.99 (s, 1H), 6.47 (dd, *J* = 8.4, 2.1 Hz, 1H), 4.93 (dd, *J* = 11.9, 5.0 Hz, 1H), 4.18 – 4.08 (m, 2H), 3.68 (ddd, *J* = 17.3, 8.2, 5.2 Hz, 2H), 3.08 – 2.99 (m, 1H), 2.97 – 2.71 (m, 5H), 2.67 (dd, *J* = 7.3, 4.7 Hz, 2H), 2.24 – 2.05 (m, 4H), 1.86 (d, *J* = 12.9 Hz, 2H), 1.70 (q, *J* = 11.8, 10.9 Hz, 2H), 1.44 (s, 9H). ^{13}C NMR (101 MHz, $CDCl_3$) δ 174.34, 171.82, 169.10, 168.11, 167.62, 155.15, 134.36, 125.22, 117.58, 113.70, 105.25, 80.22, 63.03, 56.33, 55.64, 53.25, 53.20, 48.96, 41.67, 31.56, 29.70, 28.08, 27.92, 27.86, 27.60, 22.84.

Synthesis of Compounds D21-D28: D21-D28 were synthesized following the procedure for **D1**.

Ethyl (4S,E)-4-((2S)-2-((2S)-2-(1-(2-(2,6-dioxopiperidin-3-yl)-1,3-dioxoisoindolin-5-yl)piperidine-4-carboxamido)-3-methylbutanamido)-3-(4-fluorophenyl)propanamido)-5-((S)-2-oxopiperidin-3-yl)pent-2-enoate (**D21**): ^{1}H

NMR (400 MHz, DMSO-d6) δ 11.09 (s, 1H), 8.23 – 8.13 (m, 2H), 7.85 (d, J = 8.8 Hz, 1H), 7.66 (d, J = 8.5 Hz, 1H), 7.40 – 7.30 (m, 2H), 7.28 – 7.19 (m, 3H), 7.03 (t, J = 8.9 Hz, 2H), 6.69 (dd, J = 15.7, 4.8 Hz, 1H), 5.40 (dd, J = 15.7, 1.7 Hz, 1H), 5.07 (dd, J = 12.8, 5.4 Hz, 1H), 4.57 – 4.43 (m, 2H), 4.11 (tt, J = 16.8, 9.3 Hz, 5H), 3.07 (d, J = 13.7 Hz, 2H), 3.04 – 2.95 (m, 2H), 2.85 (td, J = 13.8, 6.6 Hz, 3H), 2.64 – 2.55 (m, 2H), 2.22 – 2.11 (m, 1H), 2.06 – 1.95 (m, 2H), 1.89 (td, J = 13.8, 6.9 Hz, 2H), 1.78 (d, J = 11.9 Hz, 1H), 1.73 – 1.65 (m, 2H), 1.58 (t, J = 10.4 Hz, 2H), 1.51 – 1.39 (m, 2H), 1.23 (q, J = 7.7, 7.1 Hz, 5H), 0.80 (dd, J = 6.8, 3.8 Hz, 6H). ^{13}C NMR (101 MHz, DMSO) δ 174.41, 173.31, 173.12, 171.41, 170.88, 170.59, 168.10, 167.45, 166.07, 162.74, 160.20, 155.93, 149.85, 134.53, 133.78, 131.48, 131.41, 125.49, 119.78, 118.17, 118.07, 115.42, 114.96, 108.90, 60.35, 57.57, 54.69, 50.03, 47.68, 46.65, 41.72, 41.60, 37.47, 35.42, 31.45, 31.21, 27.19, 25.32, 21.70, 19.57, 18.09, 13.90. LC-MS: $C_{45}H_{54}FN_7O_{10}$ $[M+H]^+$, calcd: 872.39, found: 872.40.

Ethyl (4S,E)-4-((2S)-2-((2S)-2-(1'-(2-(2,6-dioxopiperidin-3-yl)-1,3-dioxoisoindolin-5-yl)-[1,4'-bipiperidine]-4-carboxamido)-3-methylbutanamido)-3-(4-fluorophenyl)propanamido)-5-((S)-2-oxopiperidin-3-yl)pent-2-enoate (**D22**): ^{1}H NMR (400 MHz, DMSO-d_6) δ 11.09 (s, 1H), 8.19 (d, *J* = 8.8 Hz, 1H), 8.13 (d, *J* = 7.6 Hz, 1H), 7.72 (d, *J* = 8.7 Hz, 1H), 7.64 (d, *J* = 8.5 Hz, 1H), 7.37 (s, 1H), 7.29 (s, 1H), 7.27 – 7.18 (m, 3H), 7.02 (t, *J* = 8.7 Hz, 2H), 6.69 (dd, *J* = 15.7, 4.7 Hz, 1H), 5.40 (d, *J* = 15.6 Hz, 1H), 5.06 (dd, *J* = 12.9, 5.3 Hz, 1H), 4.47 (dd, *J* = 15.3, 7.7 Hz, 2H), 4.10 (p, *J* = 7.1 Hz, 3H), 4.03 (d, *J* = 11.9 Hz, 2H), 3.08 (s, 2H), 2.99 – 2.79 (m, 7H), 2.59 (d, *J* = 16.8 Hz, 1H), 2.23 (s, 1H), 2.12 (d, *J* = 5.8 Hz, 3H), 2.06 – 1.96 (m, 2H), 1.93 – 1.73 (m, 7H), 1.71 – 1.61 (m, 2H), 1.50 (d, *J* = 42.9 Hz, 5H), 1.32 – 1.19 (m, 5H), 1.12 (d, *J* = 11.9 Hz, 2H), 0.83 – 0.71 (m, 6H). ^{13}C NMR (101 MHz, DMSO) δ 175.09, 173.34, 173.20, 171.45, 170.88, 170.61, 168.14, 167.47, 166.09, 160.34, 155.47, 149.77, 134.50, 133.66, 131.48, 131.40, 124.87, 119.17, 118.06, 117.73, 115.41, 115.20, 107.35, 63.97, 60.37, 57.86, 54.72, 53.79, 53.68, 49.19, 47.73, 46.73, 41.71, 37.47, 37.36, 35.75, 33.11, 31.43, 31.09, 30.07, 29.39, 26.02, 22.65, 21.65, 19.54, 18.69, 15.23. LC-MS: $C_{50}H_{63}FN_8O_{10}$ $[M+H]^+$, calcd: 955.47, found: 955.45.

Ethyl (4S,E)-4-((2S)-2-((2S)-2-(1-((1-(2-(2,6-dioxopiperidin-3-yl)-1,3-dioxoisoindolin-5-yl)piperidin-4-yl)methyl)piperidine-4-carboxamido)-3-methylbutanamido)-3-(4-fluorophenyl)propanamido)-5-((S)-2-oxopiperidin-3-yl)pent-2-enoate (**D23**): ^{1}H NMR (400 MHz, DMSO-d_6) δ 11.08 (s, 1H), 8.16 (dd, *J* = 21.3, 8.2 Hz, 2H), 7.68 (dd, *J* = 18.8, 8.6 Hz, 2H), 7.37 (s, 1H), 7.31 (d, *J* = 2.2 Hz, 1H), 7.21 (d, *J* = 8.4 Hz, 3H), 7.02 (t, *J* = 8.7 Hz, 2H), 6.68 (dd, *J* = 15.7, 4.7 Hz, 1H), 5.39 (dd, *J* = 15.7, 1.6 Hz, 1H), 5.06 (dd, *J* = 12.9, 5.4 Hz, 1H), 4.56 – 4.42 (m, 2H), 4.09

(q, J = 10.9 Hz, 5H), 3.07 (d, J = 8.3 Hz, 2H), 2.99 – 2.81 (m, 7H), 2.63 – 2.53 (m, 2H), 2.25 – 2.06 (m, 4H), 2.05 – 1.94 (m, 2H), 1.92 – 1.76 (m, 4H), 1.72 – 1.62 (m, 2H), 1.50 (dd, J = 27.5, 13.8 Hz, 7H), 1.31 – 1.18 (m, 5H), 0.78 (dd, J = 6.8, 4.0 Hz, 6H). ^{13}C NMR (101 MHz, DMSO) δ 175.03, 173.34, 173.18, 171.46, 170.87, 170.60, 168.11, 167.46, 166.08, 160.67, 155.21, 149.78, 134.50, 133.75, 131.48, 131.40, 125.51, 119.81, 118.12, 117.99, 115.41, 115.20, 108.21, 61.47, 60.36, 57.85, 54.73, 50.10, 49.02, 48.69, 47.23, 46.71, 42.36, 41.71, 38.10, 37.35, 35.74, 31.43, 31.10, 30.04, 29.04, 27.41, 26.02, 22.65, 21.65, 19.54, 18.71, 14.52. LC-MS: $C_{51}H_{65}FN_8O_{10}$ $[M+H]^+$, calcd: 969.48, found: 969.46.

Ethyl (4S,E)-4-((2S)-2-((2S)-2-(1-(2-(1-(2-(2,6-dioxopiperidin-3-yl)-1,3-dioxoisoindolin-5-yl)piperidin-4-yl)ethyl)piperidine-4-carboxamido)-3-methylbutanamido)-3-(4-fluorophenyl)propanamido)-5-((S)-2-oxopiperidin-3-yl)pent-2-enoate (**D24**): ^{1}H NMR (400 MHz, DMSO-d_6) δ 11.10 (s, 1H), 8.18 (dd, J = 16.3, 8.2 Hz, 2H), 7.75 (d, J = 8.8 Hz, 1H), 7.65 (d, J = 8.5 Hz, 1H), 7.39 (s, 1H), 7.30 (s, 1H), 7.23 (t, J = 7.0 Hz, 3H), 7.03 (t, J = 8.7 Hz, 2H), 6.70 (dd, J = 15.7, 4.7 Hz, 1H), 5.40 (d, J = 15.6 Hz, 1H), 5.07 (dd, J = 12.9, 5.4 Hz, 1H), 4.48 (q, J = 7.6 Hz, 2H), 4.18 – 3.97 (m, 5H), 3.13 – 3.03 (m, 2H), 3.00 – 2.76 (m, 7H), 2.64 – 2.52 (m, 2H), 2.35 (s, 2H), 2.25 (s, 1H), 2.15 (q, J = 10.0, 7.3 Hz, 1H), 2.01 (d, J = 11.5 Hz, 2H), 1.90 (q, J = 6.8 Hz, 4H), 1.75 (d, J = 11.2 Hz, 2H), 1.66 (d, J = 15.7 Hz, 2H), 1.56 (t, J = 9.7 Hz, 5H), 1.38 (d, J = 17.0 Hz, 3H), 1.22 (t, J = 7.1 Hz, 8H), 0.79 (t, J = 5.8 Hz, 6H). ^{13}C NMR (101 MHz, DMSO) δ 173.32, 173.10, 171.45, 170.88, 170.62, 168.13, 167.45, 166.07, 162.94, 160.02, 155.40, 148.95, 134.52, 133.52, 131.49, 131.41, 126.25, 121.53, 118.03, 117.24, 115.41, 115.20, 108.15, 60.35, 57.77, 55.92, 54.75, 49.18, 47.92, 46.66, 41.72, 38.16, 37.38, 35.08, 33.97, 31.58, 31.45, 31.18, 29.46, 26.05, 22.66, 21.69, 19.56, 18.72, 14.53. LC-MS: $C_{52}H_{67}FN_8O_{10}$ $[M+H]^+$, calcd: 983.50, found: 983.48.

Ethyl (4S,E)-4-((2S)-2-((2S)-2-(1-(((3S)-1-(2-(2,6-dioxopiperidin-3-yl)-1,3-dioxoisoindolin-5-yl)pyrrolidin-3-yl)methyl)piperidine-4-carboxamido)-3-methylbutanamido)-3-(4-fluorophenyl)propanamido)-5-((S)-2-oxopiperidin-3-yl)pent-2-enoate (**D25**): ^{1}H NMR (400 MHz, DMSO-d_6) δ 11.08 (s, 1H), 8.18 (dd, J = 20.2, 8.3 Hz, 2H), 7.73 (d, J = 8.9 Hz, 1H), 7.63 (d, J = 8.4 Hz, 1H), 7.38 (s, 1H), 7.23 (dd, J = 8.6, 5.7 Hz, 2H), 7.03 (t, J = 8.8 Hz, 2H), 6.89 (d, J = 2.2 Hz, 1H), 6.81 (dd, J = 8.7, 2.2 Hz, 1H), 6.70 (dd, J = 15.7, 4.8 Hz, 1H), 5.40 (dd, J = 15.7, 1.6 Hz, 1H), 5.06 (dd, J = 12.8, 5.4 Hz, 1H), 4.48 (q, J = 7.7 Hz, 2H), 4.12 (dq, J = 14.1, 7.5 Hz, 3H), 3.59 – 3.46 (m, 3H), 3.10 (h, J = 7.6 Hz, 3H), 2.98 – 2.79 (m, 5H), 2.64 – 2.54 (m, 3H), 2.32 (d, J = 7.0 Hz, 2H), 2.27 – 2.21 (m, 1H), 2.14 (ddd, J = 17.5, 10.9, 4.4 Hz,

2H), 2.05 – 1.97 (m, 2H), 1.90 (q, *J* = 7.1 Hz, 3H), 1.68 – 1.46 (m, 6H), 1.25 – 1.19 (m, 5H), 0.79 (dd, *J* = 6.7, 4.4 Hz, 6H). ^{13}C NMR (101 MHz, DMSO) δ 175.07, 173.32, 173.10, 171.46, 170.88, 170.66, 168.41, 167.81, 166.07, 160.82, 152.66, 149.84, 134.37, 131.49, 131.41, 125.37, 119.78, 115.90, 115.42, 115.21, 105.39, 60.35, 57.50, 54.69, 48.92, 47.60, 46.34, 41.80, 38.13, 35.86, 31.85, 31.18, 29.14, 25.71, 23.06, 21.70, 19.57, 18.73, 15.67. LC-MS: $C_{50}H_{63}FN_8O_{10}$ $[M+H]^+$, calcd: 955.47, found: 955.48.

Ethyl (4S,E)-4-((2S)-2-((2S)-2-(1-(((3R)-1-(2-(2,6-dioxopiperidin-3-yl)-1,3-dioxoisoindolin-5-yl)pyrrolidin-3-yl)methyl)piperidine-4-carboxamido)-3-methylbutanamido)-3-(4-fluorophenyl)propanamido)-5-((S)-2-oxopiperidin-3-yl)pent-2-enoate (**D26**): ^{1}H NMR (400 MHz, DMSO-d_6) δ 11.09 (s, 1H), 8.18 (dd, *J* = 19.0, 8.2 Hz, 2H), 7.73 (d, *J* = 8.8 Hz, 1H), 7.63 (d, *J* = 8.4 Hz, 1H), 7.39 (s, 1H), 7.28 – 7.19 (m, 2H), 7.03 (t, *J* = 8.8 Hz, 2H), 6.89 (d, *J* = 2.1 Hz, 1H), 6.81 (dd, *J* = 8.6, 2.2 Hz, 1H), 6.70 (dd, *J* = 15.7, 4.7 Hz, 1H), 5.40 (dd, *J* = 15.7, 1.6 Hz, 1H), 5.06 (dd, *J* = 12.8, 5.4 Hz, 1H), 4.48 (q, *J* = 7.7 Hz, 2H), 4.19 – 4.05 (m, 3H), 3.57 – 3.45 (m, 2H), 3.17 – 3.04 (m, 3H), 2.99 – 2.79 (m, 5H), 2.63 – 2.55 (m, 2H), 2.39 – 2.29 (m, 2H), 2.24 (q, *J* = 6.7, 5.7 Hz, 1H), 2.20 – 2.07 (m, 2H), 2.05 – 1.97 (m, 2H), 1.90 (p, *J* = 6.6 Hz, 3H), 1.72 (ddd, *J* = 16.8, 10.6, 6.1 Hz, 2H), 1.63 – 1.43 (m, 5H), 1.27 – 1.19 (m, 6H), 0.79 (dd, *J* = 6.8, 4.4 Hz, 6H). ^{13}C NMR (101 MHz, DMSO) δ 174.98, 173.32, 173.10, 171.46, 170.88, 170.66, 168.20, 167.72, 166.07, 163.03, 160.34, 153.09, 149.17, 134.48, 133.70, 131.49, 131.41, 125.42, 119.78, 115.90, 115.70, 115.41, 115.21, 105.11, 60.35, 57.78, 54.74, 52.61, 49.99, 47.66, 46.67, 41.72, 37.48, 36.12, 35.70, 31.76, 31.46, 31.18, 30.84, 29.66, 29.48, 26.06, 23.32, 21.70, 19.57, 18.73, 14.53. LC-MS: $C_{51}H_{64}FN_7O_{10}$ $[M+H]^+$, calcd: 955.47, found: 955.47.

Ethyl (4S,E)-4-((2S)-2-((2S)-2-(1-((1-(2-(2,6-dioxopiperidin-3-yl)-1,3-dioxoisoindolin-5-yl)azetidin-3-yl)methyl)piperidine-4-carboxamido)-3-methylbutanamido)-3-(4-fluorophenyl)propanamido)-5-((S)-2-oxopiperidin-3-yl)pent-2-enoate (**D27**): ^{1}H NMR (400 MHz, DMSO-d_6) δ 11.09 (s, 1H), 8.18 (dd, *J* = 20.1, 8.2 Hz, 2H), 7.73 (d, *J* = 8.8 Hz, 1H), 7.63 (d, *J* = 8.2 Hz, 1H), 7.38 (s, 1H), 7.23 (dd, *J* = 8.5, 5.7 Hz, 2H), 7.03 (t, *J* = 8.8 Hz, 2H), 6.77 (d, *J* = 2.1 Hz, 1H), 6.70 (dd, *J* = 15.7, 4.8 Hz, 1H), 6.64 (dd, *J* = 8.4, 2.1 Hz, 1H), 5.41 (dd, *J* = 15.7, 1.7 Hz, 1H), 5.06 (dd, *J* = 12.9, 5.4 Hz, 1H), 4.50 (dq, *J* = 15.4, 9.3, 7.7 Hz, 2H), 4.20 – 4.04 (m, 5H), 3.67 (dd, *J* = 8.5, 5.3 Hz, 2H), 3.15 – 3.04 (m, 2H), 3.00 (q, *J* = 6.7 Hz, 1H), 2.94 – 2.78 (m, 5H), 2.58 (dd, *J* = 17.8, 6.4 Hz, 3H), 2.31 – 2.21 (m, 1H), 2.20 – 2.11 (m, 1H), 2.06 – 1.83 (m, 6H), 1.76 – 1.62 (m, 2H), 1.55 (d, *J* = 11.3 Hz, 3H), 1.22 (t, *J* = 7.1 Hz, 5H), 0.79 (dd, *J* = 6.8, 4.6 Hz, 6H). ^{13}C NMR (101 MHz, DMSO) δ 173.31,

173.11, 171.45, 170.89, 170.61, 167.98, 167.67, 166.07, 160.34, 155.66, 150.56, 134.29, 131.49, 131.41, 126.27, 120.77, 116.89, 115.41, 115.21, 114.59, 104.48, 60.35, 57.78, 56.17, 54.92, 53.62, 50.31, 47.24, 42.59, 37.60, 35.56, 31.45, 31.18, 29.26, 25.82, 22.36, 21.70, 19.57, 18.72, 15.51. LC-MS: $C_{49}H_{61}FN_8O_{10}$ $[M+H]^+$, calcd: 941.45, found: 941.46.

Ethyl (4S,E)-4-((2S)-2-((2S)-2-(1-(1-(2-(2,6-dioxopiperidin-3-yl)-1,3-dioxoisoindolin-5-yl)azetidin-3-yl)piperidine-4-carboxamido)-3-methylbutanamido)-3-(4-fluorophenyl)propanamido)-5-((S)-2-oxopiperidin-3-yl)pent-2-enoate (**D28**): ^{1}H NMR (400 MHz, DMSO-d_6) δ 11.09 (s, 1H), 8.18 (dd, *J* = 15.7, 8.3 Hz, 2H), 7.75 (d, *J* = 8.8 Hz, 1H), 7.65 (d, *J* = 8.3 Hz, 1H), 7.39 (s, 1H), 7.23 (dd, *J* = 8.6, 5.7 Hz, 2H), 7.03 (t, *J* = 8.9 Hz, 2H), 6.79 (d, *J* = 2.1 Hz, 1H), 6.73 – 6.63 (m, 2H), 5.40 (dd, *J* = 15.7, 1.7 Hz, 1H), 5.06 (dd, *J* = 12.8, 5.4 Hz, 1H), 4.48 (q, *J* = 7.7 Hz, 2H), 4.19 – 4.02 (m, 5H), 3.88 – 3.76 (m, 2H), 3.28 (p, *J* = 6.1 Hz, 1H), 3.09 (s, 2H), 2.95 – 2.77 (m, 5H), 2.63 – 2.52 (m, 2H), 2.27 (td, *J* = 11.1, 5.6 Hz, 1H), 2.16 (hept, *J* = 4.8 Hz, 1H), 2.06 – 1.96 (m, 2H), 1.94 – 1.79 (m, 4H), 1.74 – 1.65 (m, 2H), 1.64 – 1.41 (m, 5H), 1.33 – 1.26 (m, 1H), 1.22 (t, *J* = 7.1 Hz, 3H), 0.79 (dd, *J* = 6.7, 4.3 Hz, 6H). ^{13}C NMR (101 MHz, DMSO) δ 174.86, 173.31, 173.11, 171.45, 170.88, 170.61, 167.96, 167.66, 166.07, 162.37, 159.47, 155.44, 150.55, 134.30, 132.61, 131.49, 131.41, 125.33, 119.78, 117.31, 115.41, 115.21, 114.67, 104.95, 60.35, 57.80, 55.85, 54.82, 54.75, 49.57, 49.43, 49.17, 46.68, 41.81, 41.71, 37.47, 35.79, 31.44, 31.18, 29.19, 28.51, 26.05, 22.67, 21.69, 19.57, 18.73, 14.54. LC-MS: $C_{48}H_{59}FN_8O_{10}$ $[M+H]^+$, calcd: 927.44, found: 927.42.

Synthesis of Compounds F1-F8: F1-F8 were synthesized following the procedure for **A4**.

Synthesis of Compounds D29-D35, D34-Neg: D29-D35, D34-Neg were synthesized following the procedure for **A7**.

6-((2-(2,6-dioxopiperidin-3-yl)-1,3-dioxoisoindolin-4-yl)oxy)-N-((S)-1-(((S)-3-(4-fluorophenyl)-1-oxo-1-(((S)-1-oxo-3-((S)-2-oxopiperidin-3-yl)propan-2-yl)amino)propan-2-yl)amino)-3-methyl-1-oxobutan-2-yl)hexanamide (**D29**): ^{1}H NMR (400 MHz, DMSO-d_6) δ 11.12 (s, 1H), 9.22 (s, 1H), 8.45 (d, *J* = 7.9 Hz, 1H), 8.17 (d, *J* = 7.8 Hz, 1H), 7.98 (dd, *J* = 17.5, 7.9 Hz, 1H), 7.84 – 7.73 (m, 2H), 7.50 (d, *J* = 8.6 Hz, 1H), 7.44 (d, *J* = 7.4 Hz, 1H), 7.33 – 7.21 (m, 2H), 7.06 (t, *J* = 8.8 Hz, 2H), 5.08 (dd, *J* = 12.8, 5.4 Hz, 1H), 4.51 (q, *J* = 8.3, 7.8 Hz, 1H), 4.22 – 4.11 (m, 3H), 3.20 – 3.03 (m, 2H), 3.01 – 2.73 (m, 3H), 2.64 – 2.52 (m, 2H), 2.25 – 1.98 (m, 5H), 1.90 (dt, *J* = 13.6, 6.5 Hz, 2H), 1.81 – 1.58 (m, 4H), 1.57 – 1.38 (m, 5H), 1.35 – 1.18 (m, 2H), 0.77 (t, *J* = 5.9 Hz, 6H). ^{13}C NMR (101 MHz, DMSO) δ 201.43, 173.30, 173.10, 172.62,

171.96, 171.62, 171.47, 170.46, 167.34, 165.79, 160.26, 156.47, 137.53, 133.71, 131.55, 131.48, 120.22, 116.65, 115.61, 115.32, 115.11, 69.12, 57.89, 55.78, 54.70, 49.18, 41.66, 36.96, 35.49, 32.35, 31.00, 30.12, 28.61, 26.06, 25.53, 25.39, 22.47, 21.77, 19.57, 18.65, 18.59. LC-MS: $C_{41}H_{49}FN_6O_{10}$ $[M+H]^+$, calcd: 805.35, found: 805.36.

7-((2-(2,6-dioxopiperidin-3-yl)-1,3-dioxoisoindolin-4-yl)oxy)-N-((S)-1-(((S)-3-(4-fluorophenyl)-1-oxo-1-(((S)-1-oxo-3-((S)-2-oxopiperidin-3-yl)propan-2-yl)amino)propan-2-yl)amino)-3-methyl-1-oxobutan-2-yl)heptanamide (**D30**): ^{1}H NMR (400 MHz, DMSO-d6) δ 11.12 (s, 1H), 9.22 (s, 1H), 8.32 (dd, J = 107.3, 7.8 Hz, 1H), 8.09 – 7.94 (m, 1H), 7.84 – 7.70 (m, 2H), 7.66 – 7.41 (m, 3H), 7.33 – 7.24 (m, 2H), 7.04 (q, J = 8.9 Hz, 2H), 5.71 (dd, J = 6.0, 4.1 Hz, 1H), 5.09 (dd, J = 12.9, 5.4 Hz, 1H), 4.51 (q, J = 7.1, 6.5 Hz, 1H), 4.23 – 4.11 (m, 3H), 3.17 – 3.03 (m, 2H), 2.98 – 2.84 (m, 3H), 2.58 (t, J = 14.1 Hz, 2H), 2.22 – 1.98 (m, 5H), 1.89 (h, J = 6.5 Hz, 2H), 1.80 – 1.55 (m, 4H), 1.53 – 1.39 (m, 5H), 1.34 – 1.16 (m, 4H), 0.77 (q, J = 6.3, 5.4 Hz, 6H). ^{13}C NMR (101 MHz, DMSO) δ 201.43, 173.90, 173.31, 173.11, 172.68, 172.57, 171.96, 171.64, 171.50, 171.43, 170.47, 167.35, 165.81, 163.58, 160.22, 156.49, 137.53, 133.71, 131.55, 131.47, 120.21, 116.67, 115.61, 115.31, 115.17, 115.11, 114.40, 91.67, 69.18, 57.85, 55.79, 53.86, 51.85, 49.19, 42.51, 36.96, 36.25, 34.66, 31.96, 31.23, 31.02, 30.12, 28.75, 26.06, 25.80, 25.44, 22.46, 21.76, 19.66, 19.57, 18.65, 18.59. LC-MS: $C_{42}H_{51}FN_6O_{10}$ $[M+H]^+$, calcd: 819.37, found: 819.36.

8-((2-(2,6-dioxopiperidin-3-yl)-1,3-dioxoisoindolin-4-yl)oxy)-N-((S)-1-(((S)-3-(4-fluorophenyl)-1-oxo-1-(((S)-1-oxo-3-((S)-2-oxopiperidin-3-yl)propan-2-yl)amino)propan-2-yl)amino)-3-methyl-1-oxobutan-2-yl)octanamide (**D31**): ^{1}H NMR (400 MHz, DMSO-d_6) δ 11.12 (s, 1H), 9.22 (s, 1H), 8.45 (d, J = 7.9 Hz, 1H), 8.18 (d, J = 7.8 Hz, 1H), 8.05 (dd, J = 26.3, 7.3 Hz, 1H), 7.85 – 7.68 (m, 2H), 7.47 (dd, J = 26.7, 7.7 Hz, 2H), 7.34 – 7.24 (m, 2H), 7.05 (t, J = 9.0 Hz, 2H), 5.82 – 5.65 (m, 1H), 5.09 (dd, J = 12.8, 5.4 Hz, 1H), 4.51 (q, J = 7.9 Hz, 1H), 4.24 – 4.10 (m, 3H), 3.14 (d, J = 39.9 Hz, 2H), 3.01 – 2.71 (m, 3H), 2.59 (d, J = 18.0 Hz, 2H), 2.22 – 1.99 (m, 5H), 1.96 – 1.82 (m, 2H), 1.80 – 1.56 (m, 4H), 1.45 (dd, J = 18.4, 11.3 Hz, 5H), 1.37 – 1.06 (m, 6H), 0.77 (d, J = 6.2 Hz, 6H). ^{13}C NMR (101 MHz, DMSO) δ 201.43, 173.91, 173.31, 173.09, 172.68, 172.58, 171.96, 171.64, 171.12, 170.47, 167.35, 165.80, 157.63, 137.51, 134.08, 133.71, 131.55, 131.47, 120.22, 116.66, 115.60, 115.31, 115.11, 69.21, 59.43, 55.78, 53.92, 49.19, 43.07, 36.96, 35.53, 31.42, 31.02, 29.05, 28.88, 26.06, 25.81, 25.66, 22.46, 21.77, 20.30, 18.66, 18.59. LC-MS: $C_{43}H_{53}FN_6O_{10}$ $[M+H]^+$, calcd: 833.38, found: 833.36.

9-((2-(2,6-dioxopiperidin-3-yl)-1,3-dioxoisoindolin-4-yl)oxy)-N-((S)-1-(((S)-3-(4-fluorophenyl)-1-oxo-1-(((S)-1-oxo-3-((S)-2-oxopiperidin-3-yl)propan-2-

yl)amino)propan-2-yl)amino)-3-methyl-1-oxobutan-2-yl)nonanamide (**D32**): ^{1}H NMR (400 MHz, DMSO-*d*$_6$) δ 11.13 (s, 1H), 9.22 (s, 1H), 8.09 – 7.95 (m, 1H), 7.83 – 7.70 (m, 2H), 7.51 (d, *J* = 8.6 Hz, 1H), 7.44 (d, *J* = 7.2 Hz, 1H), 7.33 – 7.24 (m, 2H), 7.10 – 6.98 (m, 2H), 5.09 (dd, *J* = 12.8, 5.4 Hz, 1H), 4.51 (q, *J* = 7.9, 7.2 Hz, 1H), 4.23 – 4.12 (m, 3H), 3.17 (d, *J* = 13.2 Hz, 1H), 3.13 – 3.02 (m, 2H), 3.01 – 2.71 (m, 3H), 2.65 – 2.52 (m, 2H), 2.20 – 1.99 (m, 5H), 1.88 (h, *J* = 6.5, 5.9 Hz, 2H), 1.81 – 1.64 (m, 3H), 1.58 – 1.38 (m, 6H), 1.27 (dd, *J* = 18.4, 9.5 Hz, 8H), 0.77 (q, *J* = 5.9 Hz, 6H). ^{13}C NMR (101 MHz, DMSO) δ 201.42, 173.31, 173.10, 172.70, 171.96, 171.64, 170.47, 167.35, 165.09, 160.17, 156.50, 137.51, 134.11, 133.71, 131.54, 131.47, 120.23, 116.66, 115.60, 115.31, 115.17, 115.10, 114.97, 69.25, 57.85, 55.79, 54.52, 50.02, 41.66, 36.96, 34.99, 31.42, 31.02, 29.19, 29.09, 28.88, 26.06, 25.84, 25.75, 22.47, 21.77, 19.65, 19.57, 18.66, 18.60. LC-MS: $C_{44}H_{55}FN_6O_{10}$ [M+H]$^+$, calcd: 847.40, found: 847.41.

10-((2-(2,6-dioxopiperidin-3-yl)-1,3-dioxoisoindolin-4-yl)oxy)-N-((S)-1-(((S)-3-(4-fluorophenyl)-1-oxo-1-(((S)-1-oxo-3-((S)-2-oxopiperidin-3-yl)propan-2-yl)amino)propan-2-yl)amino)-3-methyl-1-oxobutan-2-yl)decanamide (**D33**): ^{1}H NMR (400 MHz, DMSO-*d*$_6$) δ 11.12 (s, 1H), 9.22 (s, 1H), 8.45 (d, *J* = 7.8 Hz, 1H), 8.18 (d, *J* = 7.8 Hz, 1H), 7.84 – 7.70 (m, 2H), 7.51 (d, *J* = 8.6 Hz, 1H), 7.44 (d, *J* = 6.9 Hz, 1H), 7.27 (dd, *J* = 8.6, 5.5 Hz, 2H), 7.05 (t, *J* = 8.8 Hz, 2H), 5.09 (dd, *J* = 12.8, 5.4 Hz, 1H), 4.51 (q, *J* = 7.9 Hz, 1H), 4.17 (dt, *J* = 20.1, 7.6 Hz, 3H), 3.14 – 3.04 (m, 2H), 3.00 – 2.76 (m, 3H), 2.64 – 2.52 (m, 2H), 2.18 – 1.99 (m, 5H), 1.89 (h, *J* = 6.4 Hz, 2H), 1.79 – 1.57 (m, 4H), 1.53 – 1.40 (m, 5H), 1.36 – 1.14 (m, 11H), 0.78 (t, *J* = 6.1 Hz, 6H). ^{13}C NMR (101 MHz, DMSO) δ 201.42, 173.30, 173.09, 172.69, 171.96, 171.63, 170.46, 167.34, 165.80, 162.17, 160.83, 157.29, 137.51, 134.09, 133.71, 131.54, 131.47, 120.22, 116.66, 115.60, 115.31, 115.10, 68.71, 57.85, 55.79, 54.52, 49.19, 44.19, 37.18, 36.96, 35.57, 32.07, 31.02, 30.13, 29.37, 29.09, 28.90, 26.07, 25.86, 25.74, 23.06, 20.86, 19.57, 18.66. LC-MS: $C_{45}H_{57}FN_6O_{10}$ [M+H]$^+$, calcd: 861.42, found: 861.40.

7-((2-(2,6-dioxopiperidin-3-yl)-1,3-dioxoisoindolin-5-yl)oxy)-N-((S)-1-(((S)-3-(4-fluorophenyl)-1-oxo-1-(((S)-1-oxo-3-((S)-2-oxopiperidin-3-yl)propan-2-yl)amino)propan-2-yl)amino)-3-methyl-1-oxobutan-2-yl)heptanamide (**D34**): ^{1}H NMR (400 MHz, DMSO-d6) δ 11.13 (s, 1H), 9.22 (s, 1H), 8.04 (dd, J = 18.7, 8.1 Hz, 1H), 7.83 (d, J = 8.3 Hz, 1H), 7.75 (dd, J = 13.5, 9.0 Hz, 1H), 7.47 – 7.22 (m, 5H), 7.05 (t, J = 9.1 Hz, 2H), 5.12 (dd, J = 12.9, 5.3 Hz, 1H), 4.50 (t, J = 7.7 Hz, 1H), 4.16 (d, J = 6.9 Hz, 3H), 3.18 (d, J = 13.1 Hz, 1H), 3.08 (s, 2H), 3.00 – 2.71 (m, 3H), 2.66 – 2.54 (m, 2H), 2.23 – 1.98 (m, 5H), 1.88 (t, J = 6.9 Hz, 2H), 1.72 (q, J = 6.9 Hz, 3H), 1.61 – 1.37 (m, 6H), 1.35 – 1.22 (m, 3H), 0.78 (t, J = 5.8 Hz, 6H). ^{13}C NMR (101 MHz, DMSO) δ 201.42, 173.89, 173.29, 172.67, 171.96, 171.64, 171.32, 170.44, 167.39, 167.32,

164.58, 134.43, 131.55, 131.47, 125.80, 123.32, 121.16, 115.31, 115.18, 114.98, 109.29, 69.22, 57.86, 55.21, 48.58, 41.63, 36.89, 35.48, 31.42, 31.02, 28.10, 26.07, 25.75, 24.94, 22.54, 20.98, 19.57, 18.66, 18.60. LC-MS: $C_{42}H_{51}FN_6O_{10}$ $[M+H]^+$, calcd: 819.37, found: 819.33.

8-((2-(2,6-dioxopiperidin-3-yl)-1,3-dioxoisoindolin-5-yl)oxy)-N-((S)-1-(((S)-3-(4-fluorophenyl)-1-oxo-1-(((S)-1-oxo-3-((S)-2-oxopiperidin-3-yl)propan-2-yl)amino)propan-2-yl)amino)-3-methyl-1-oxobutan-2-yl)octanamide (**D35**): ^{1}H NMR (400 MHz, DMSO-d_6) δ 11.14 (s, 1H), 9.23 (s, 1H), 8.46 (d, J = 8.0 Hz, 1H), 8.18 (d, J = 7.8 Hz, 1H), 7.83 (d, J = 8.3 Hz, 1H), 7.79 – 7.70 (m, 1H), 7.45 – 7.41 (m, 1H), 7.34 (dd, J = 8.3, 2.3 Hz, 1H), 7.28 (dd, J = 8.3, 5.4 Hz, 2H), 7.09 – 7.00 (m, 2H), 5.13 (dd, J = 12.9, 5.3 Hz, 1H), 4.56 – 4.46 (m, 1H), 4.16 (t, J = 6.6 Hz, 3H), 3.18 (d, J = 13.1 Hz, 1H), 3.10 (t, J = 5.9 Hz, 2H), 3.00 – 2.73 (m, 3H), 2.66 – 2.53 (m, 2H), 2.22 – 2.00 (m, 5H), 1.89 (dq, J = 13.3, 7.1 Hz, 2H), 1.79 – 1.66 (m, 3H), 1.59 – 1.41 (m, 5H), 1.36 – 1.22 (m, 6H), 0.78 (t, J = 6.5 Hz, 6H). ^{13}C NMR (101 MHz, DMSO) δ 201.41, 173.43, 173.28, 173.09, 172.67, 171.95, 171.64, 170.43, 167.37, 167.30, 164.58, 160.27, 135.14, 131.54, 131.46, 125.77, 123.31, 121.17, 115.30, 115.17, 115.10, 114.97, 109.88, 69.23, 64.67, 57.84, 55.79, 55.38, 54.52, 49.41, 41.66, 36.96, 35.52, 33.20, 31.42, 31.03, 30.13, 29.01, 28.89, 28.81, 25.65, 24.89, 22.54, 21.77, 19.62, 19.57, 18.66, 18.59. LC-MS: $C_{43}H_{53}FN_6O_{10}$ $[M+H]^+$, calcd: 833.38, found: 833.36.

N-((S)-1-(((S)-3-(4-fluorophenyl)-1-oxo-1-(((S)-1-oxo-3-((S)-2-oxopiperidin-3-yl)propan-2-yl)amino)propan-2-yl)amino)-3-methyl-1-oxobutan-2-yl)-7-((2-(1-methyl-2,6-dioxopiperidin-3-yl)-1,3-dioxoisoindolin-5-yl)oxy)heptanamide (**D34-Neg**): ^{1}H NMR (400 MHz, DMSO-d_6) δ 9.23 (s, 1H), 8.45 (d, J = 8.0 Hz, 1H), 8.17 (d, J = 7.8 Hz, 1H), 7.83 (d, J = 8.3 Hz, 1H), 7.74 (d, J = 13.4 Hz, 1H), 7.43 – 7.40 (m, 1H), 7.35 (dd, J = 8.3, 2.3 Hz, 1H), 7.28 (dd, J = 8.6, 5.6 Hz, 2H), 7.04 (td, J = 8.9, 6.6 Hz, 2H), 5.19 (dd, J = 13.1, 5.3 Hz, 1H), 4.56 – 4.43 (m, 1H), 4.21 – 4.11 (m, 3H), 3.09 (d, J = 4.3 Hz, 2H), 3.02 (s, 3H), 2.99 – 2.85 (m, 2H), 2.81 – 2.74 (m, 1H), 2.56 (dd, J = 13.2, 4.4 Hz, 1H), 2.24 – 1.99 (m, 5H), 1.95 – 1.82 (m, 2H), 1.73 (q, J = 7.7 Hz, 3H), 1.47 (dq, J = 31.1, 7.6 Hz, 5H), 1.32 – 1.22 (m, 6H), 0.84 – 0.73 (m, 6H). ^{13}C NMR (101 MHz, DMSO) δ 201.41, 173.10, 172.67, 172.25, 171.96, 171.64, 170.18, 167.37, 167.30, 164.60, 160.11, 134.40, 131.55, 131.47, 125.81, 123.30, 121.17, 115.90, 115.10, 109.32, 69.22, 57.87, 55.80, 54.52, 50.00, 43.00, 37.18, 36.97, 35.48, 31.58, 31.01, 29.46, 28.73, 27.08, 26.07, 25.75, 25.55, 21.78, 21.73, 19.57, 18.66, 18.60. LC-MS: $C_{43}H_{53}FN_6O_{10}$ $[M+H]^+$, calcd: 833.38, found: 833.35.

Part C. Copies of Spectra

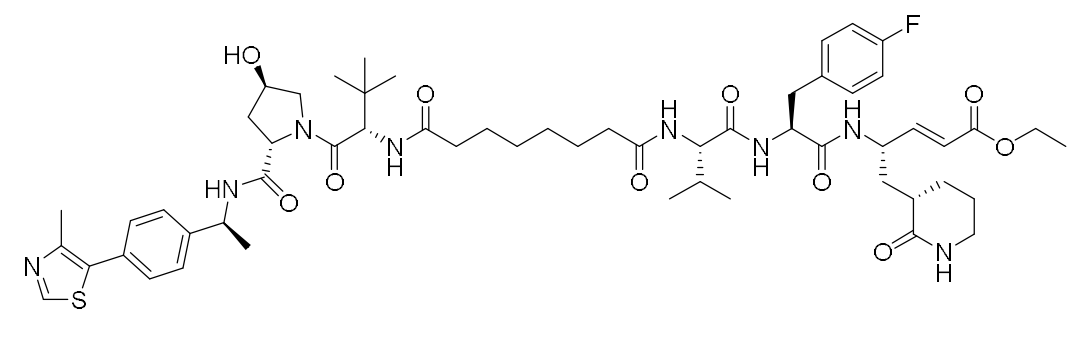

D1

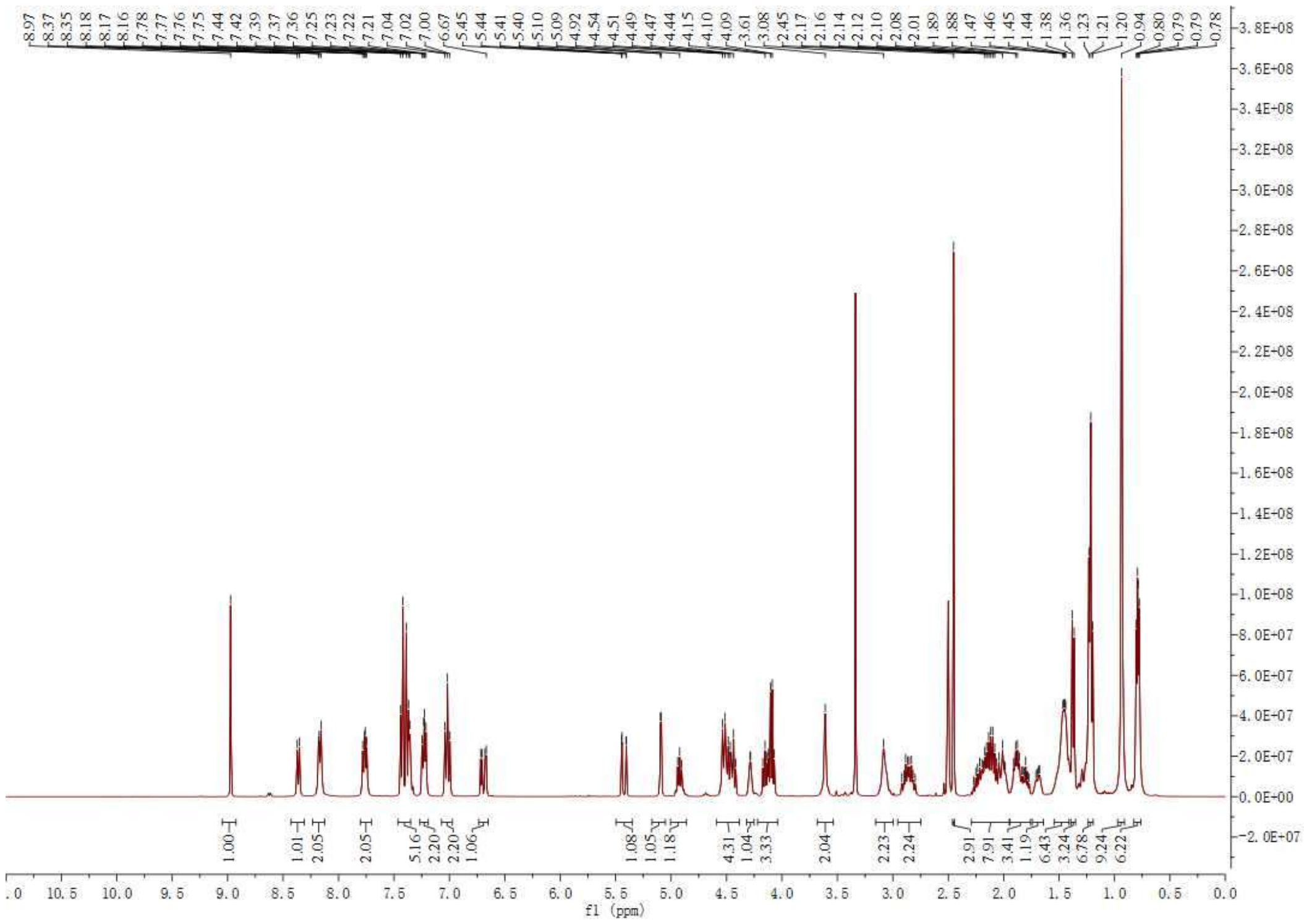
8.97 8.37 8.35 8.18 8.17 8.16 7.78 7.77 7.76 7.75 7.44 7.42 7.39 7.37 7.36 7.25 7.23 7.22 7.21 7.04 7.02 7.00 6.67 5.45 5.44 5.41 5.40 5.10 5.09 4.92 4.54 4.51 4.49 4.47 4.44 4.15 4.10 4.09 3.61 3.08 2.45 2.17 2.16 2.14 2.12 2.10 2.08 2.01 1.89 1.88 1.47 1.46 1.45 1.44 1.38 1.36 1.23 1.21 1.20 0.94 0.80 0.79 0.79 0.78
1.00 1.01 2.05 2.05 5.16 2.20 2.20 1.06 1.08 1.05 1.18 4.31 1.04 3.33 2.04 2.23 2.24 2.91 7.91 3.41 1.19 6.43 3.24 6.78 9.24 6.22
f1 (ppm)

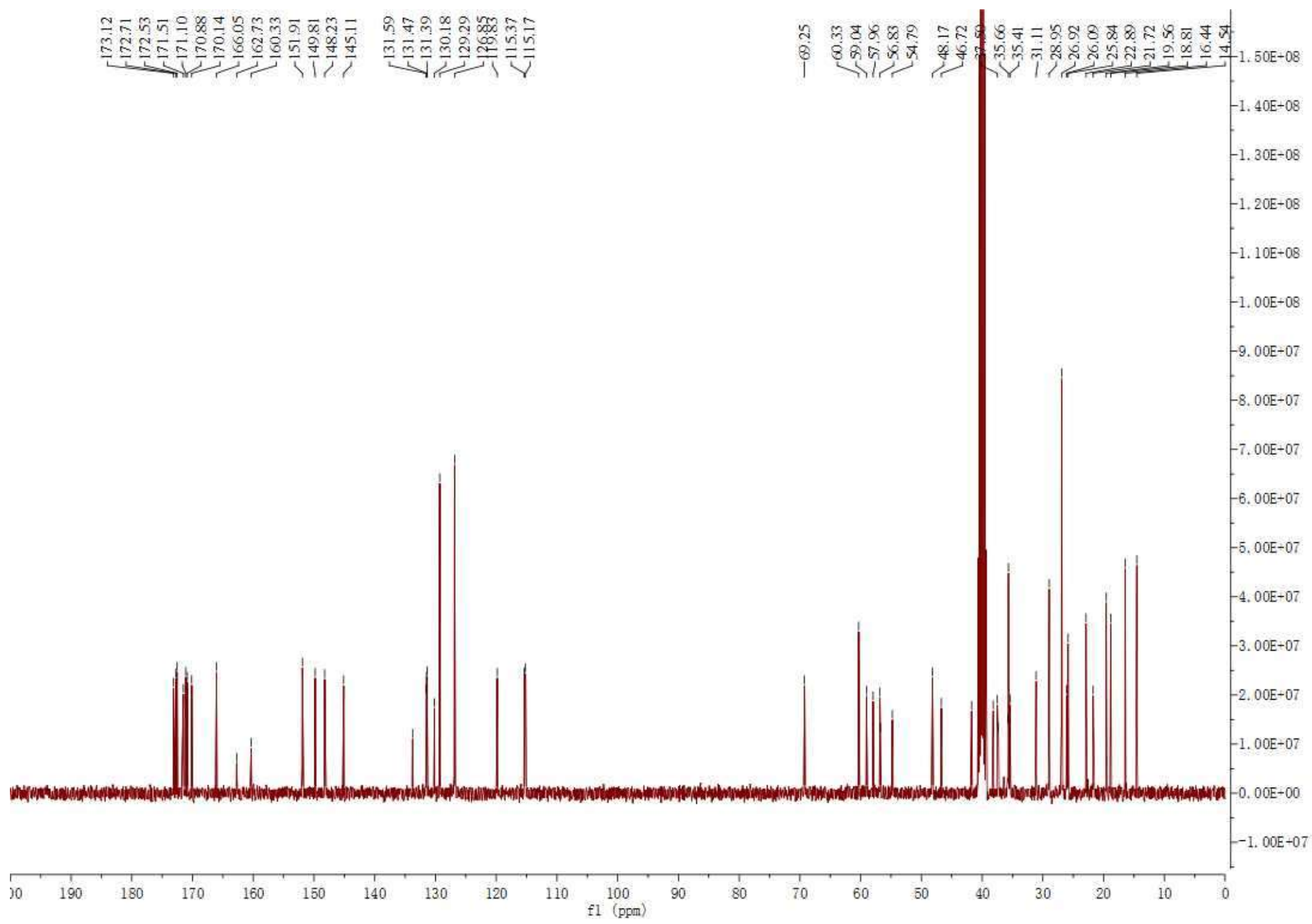
173.12 172.71 172.53 171.51 171.10 170.88 170.14 166.05 162.73 160.33 151.91 149.81 148.23 145.11 131.59 131.47 131.39 130.18 129.29 126.85 119.83 115.37 115.17
69.25 60.33 59.04 57.96 56.83 54.79 48.17 46.72 35.66 35.41 31.11 28.95 26.92 26.09 25.84 22.89 21.72 19.56 18.81 16.44 14.54
f1 (ppm)

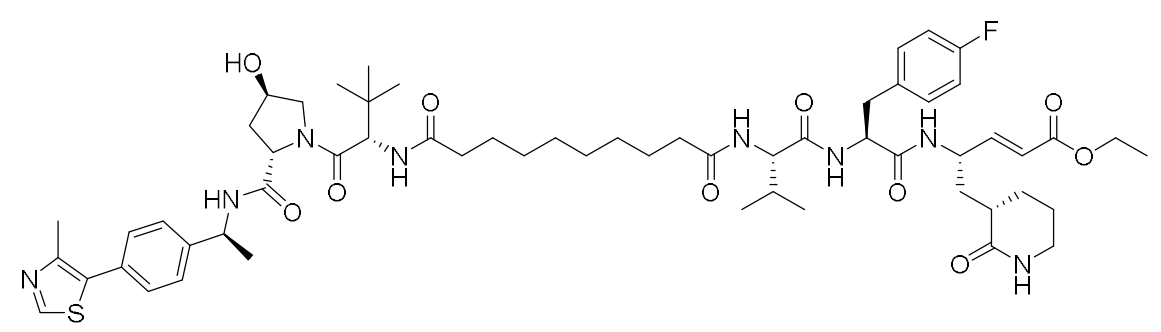

HO
HN
N
S
O
N
H
F

D2

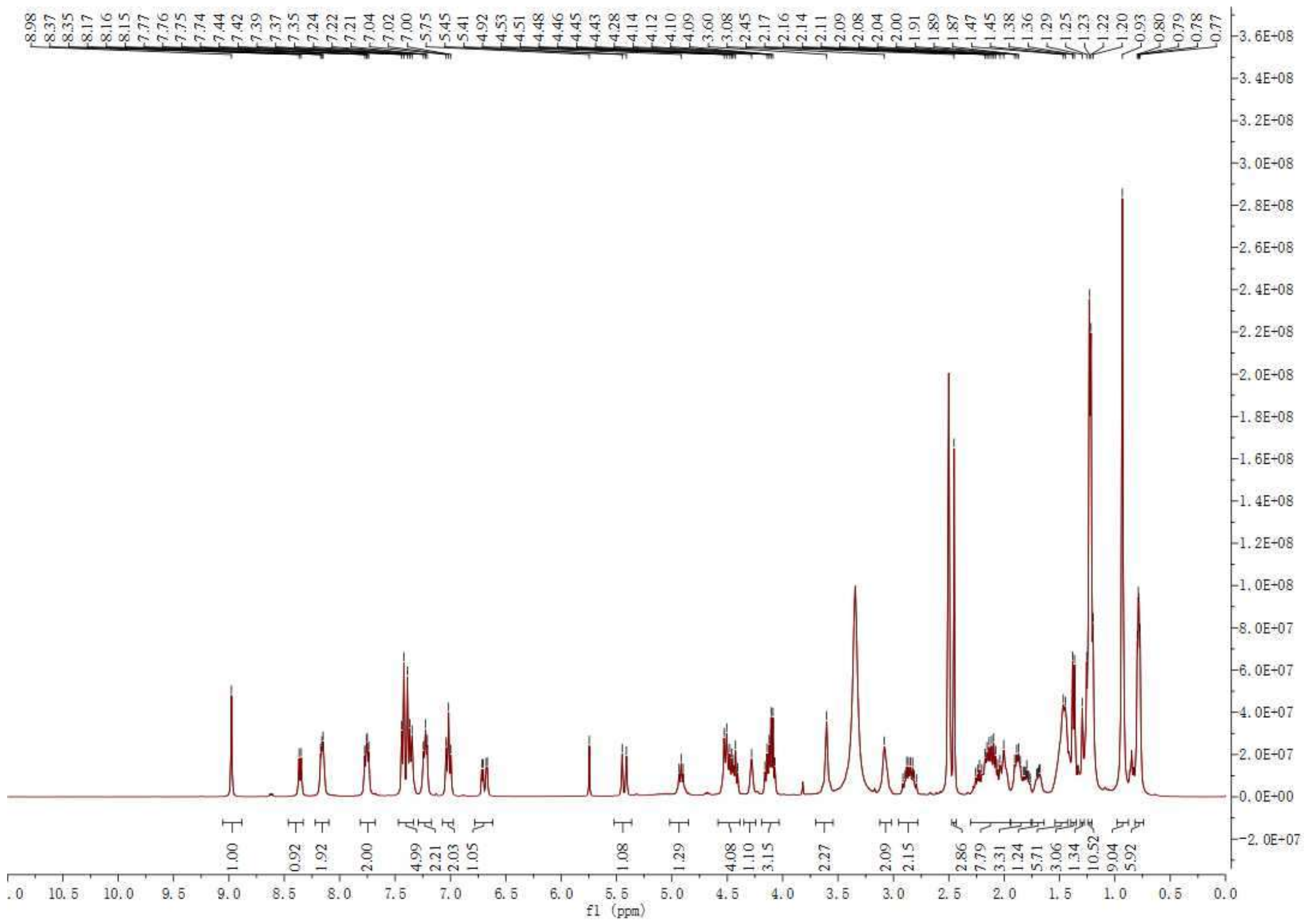

8.98 8.37 8.35 8.17 8.16 8.15 7.77 7.76 7.75 7.74 7.44 7.42 7.39 7.37 7.35 7.24 7.22 7.21 7.04 7.02 7.00 5.75 5.45 5.41 4.92 4.53 4.51 4.48 4.46 4.45 4.43 4.28 4.14 4.12 4.10 4.09 3.60 3.08 2.45 2.17 2.16 2.14 2.11 2.09 2.08 2.04 2.00 1.91 1.89 1.87 1.47 1.45 1.38 1.36 1.29 1.25 1.23 1.22 1.20 0.93 0.80 0.79 0.78 0.77
1.00 0.92 1.92 2.00 4.99 2.21 2.03 1.05 1.08 1.29 4.08 1.10 3.15 2.27 2.09 2.15 2.86 7.79 3.31 1.24 5.71 3.06 1.34 10.52 9.04 5.92
f1 (ppm)

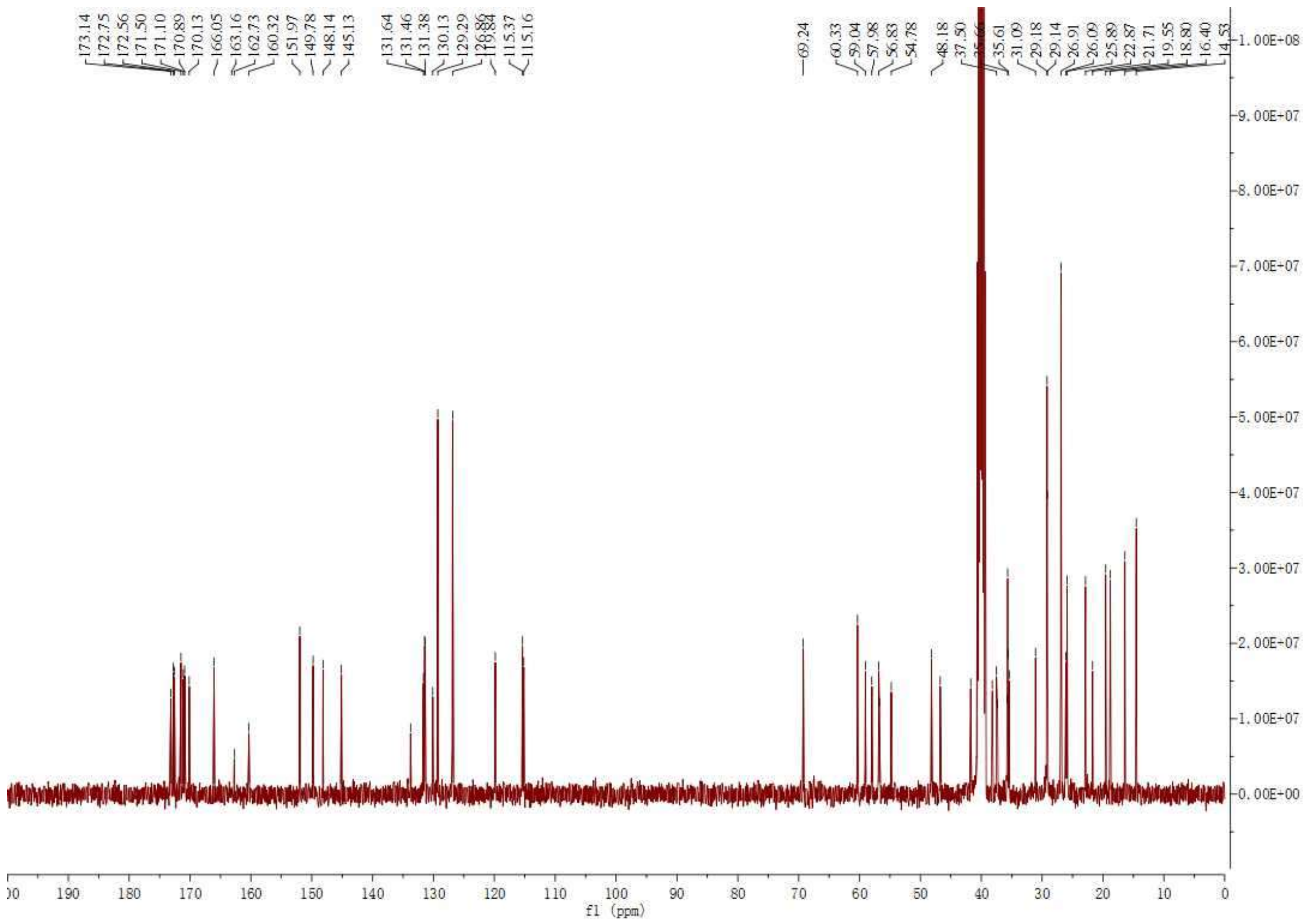

173.14 172.75 172.56 171.50 171.10 170.89 170.13 166.05 163.16 162.73 160.32 151.97 149.78 148.14 145.13 131.64 131.46 131.38 130.13 129.29 115.37 115.16
69.24 60.33 59.04 57.98 56.83 54.78 48.18 37.50 35.61 31.09 29.18 29.14 26.91 26.09 25.89 22.87 21.71 19.55 18.80 16.40
f1 (ppm)

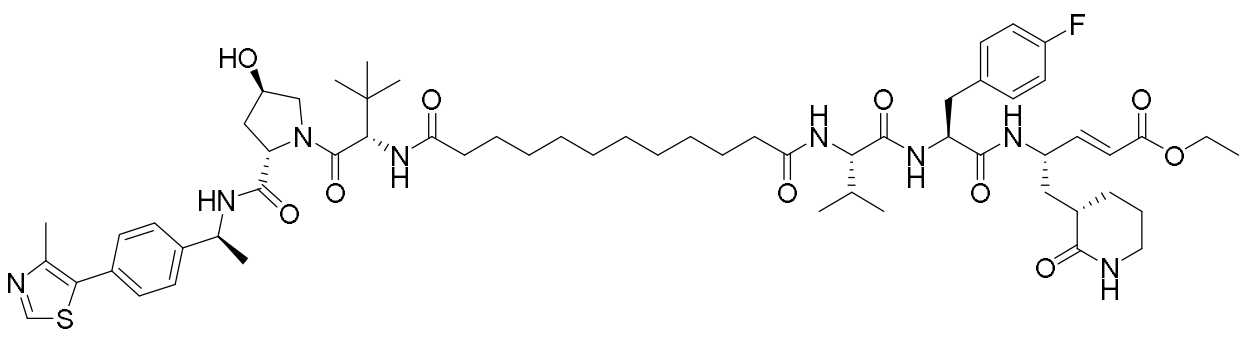
HO
N
O
HN
N
S
H
F
NH
O

D3

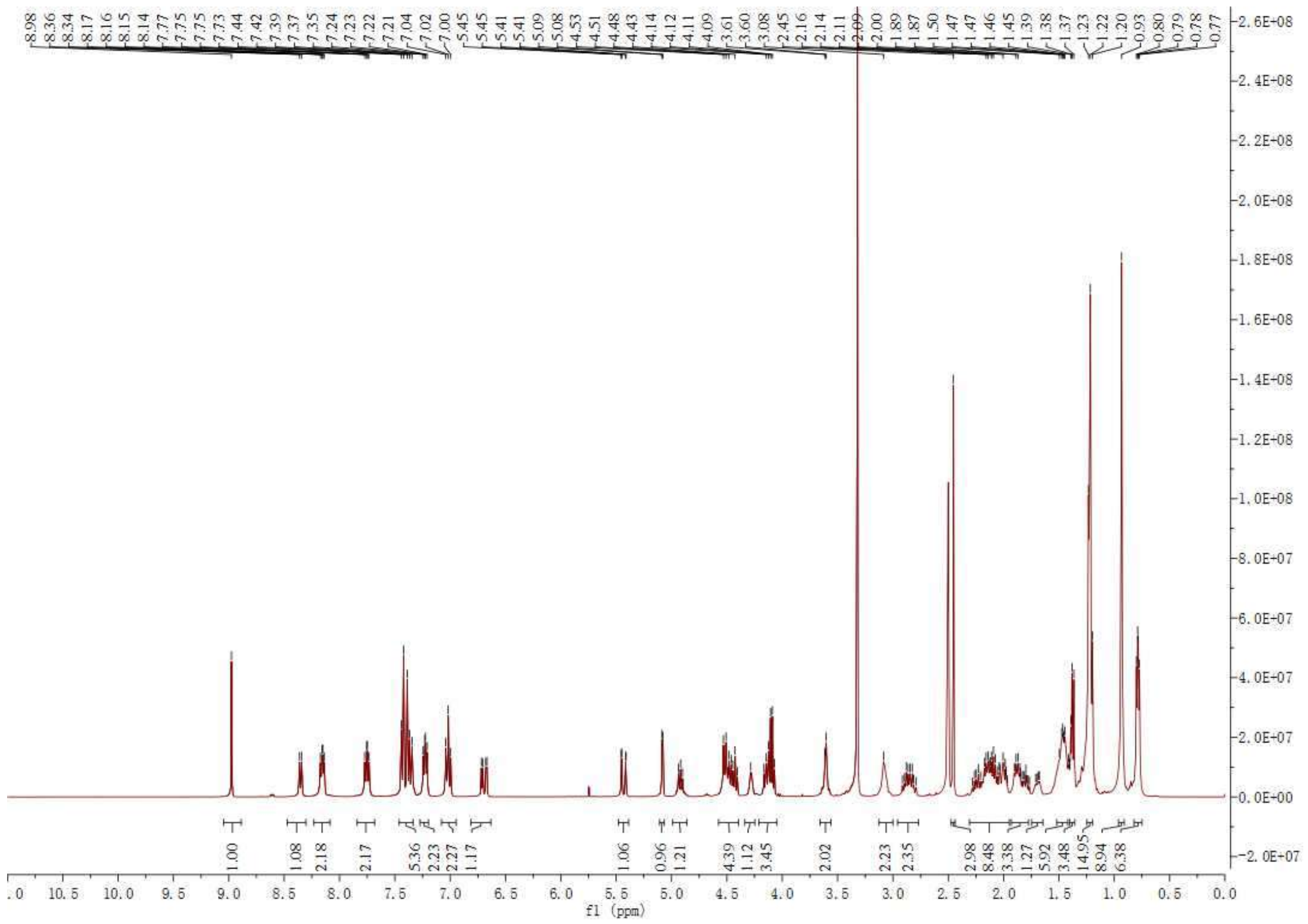
8.98 8.36 8.34 8.17 8.16 8.15 8.14 7.77 7.75 7.75 7.73 7.44 7.42 7.39 7.37 7.35 7.24 7.23 7.22 7.21 7.04 7.02 7.00 5.45 5.41 5.41 5.09 5.08 4.53 4.51 4.48 4.43 4.14 4.12 4.11 4.09 3.61 3.60 3.08 2.45 2.16 2.14 2.11 2.09 2.00 1.89 1.87 1.50 1.47 1.47 1.46 1.45 1.39 1.38 1.37 1.23 1.22 1.20 0.93 0.80 0.79 0.78 0.77
1.00 1.08 2.18 2.17 5.36 2.23 2.27 1.17 1.06 0.96 1.21 4.39 1.12 3.45 2.02 2.23 2.35 2.98 8.48 3.38 1.27 5.92 3.48 14.95 8.94 6.38
f1 (ppm)

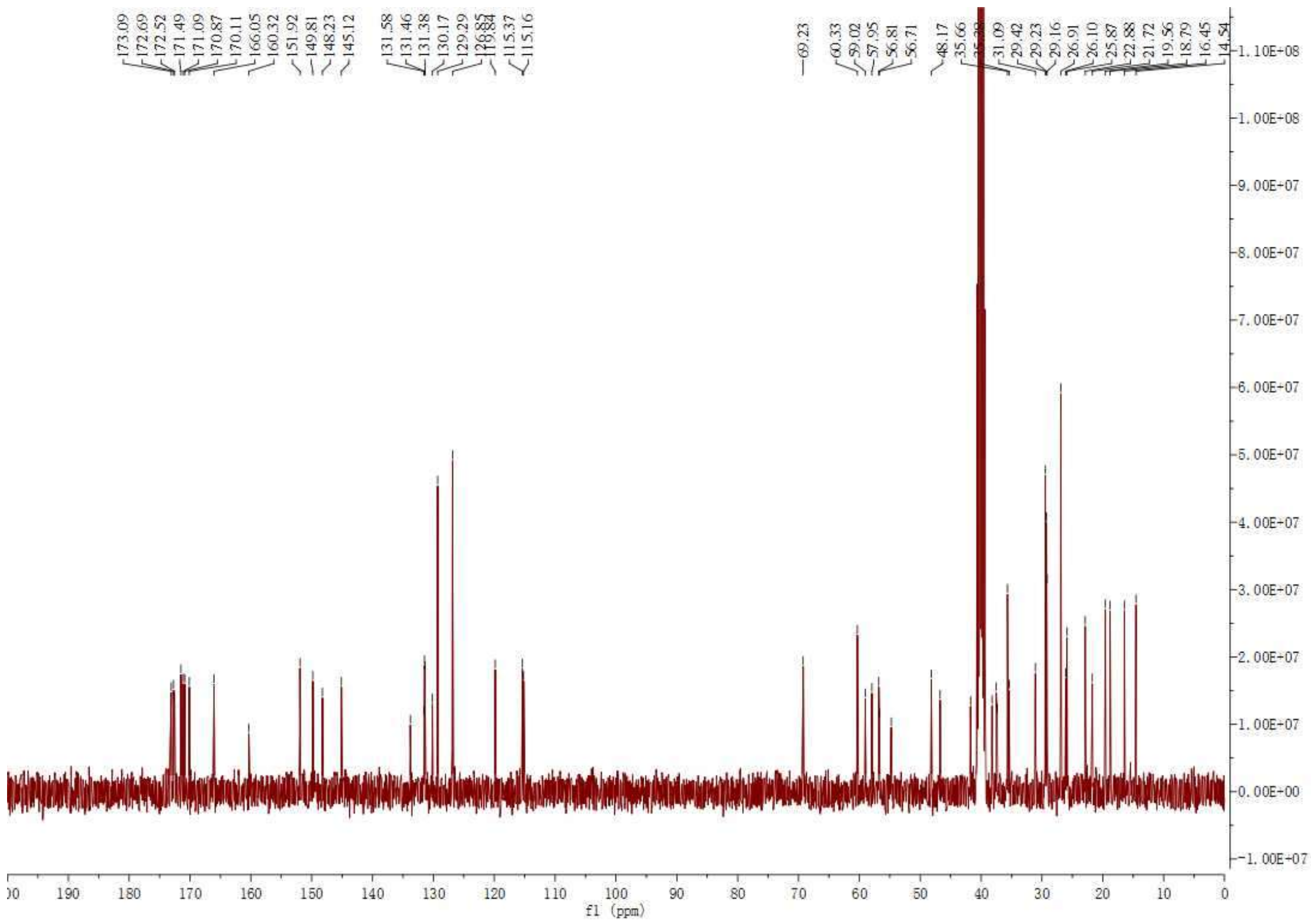
173.09 172.69 172.52 171.49 171.09 170.87 170.11 166.05 160.32 151.92 149.81 148.23 145.12 131.58 131.46 131.38 130.17 129.29 126.85 119.84 115.37 115.16 69.23 60.33 59.02 57.95 56.81 56.71 48.17 35.66 31.09 29.42 29.23 29.16 26.91 26.10 25.87 22.88 21.72 19.56 18.79 16.45 14.54
f1 (ppm)

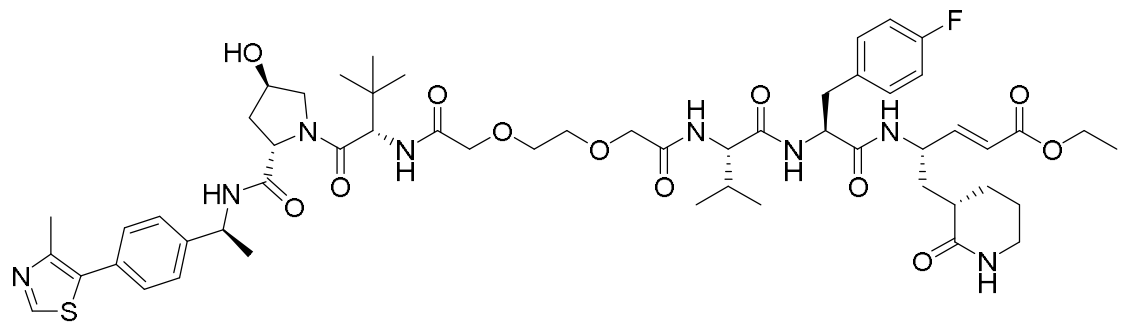
HO
HN
N
S
N
O
H
O
O
O
N
H
O
N
H
O
F
N
H
O
O
O
NH

D4

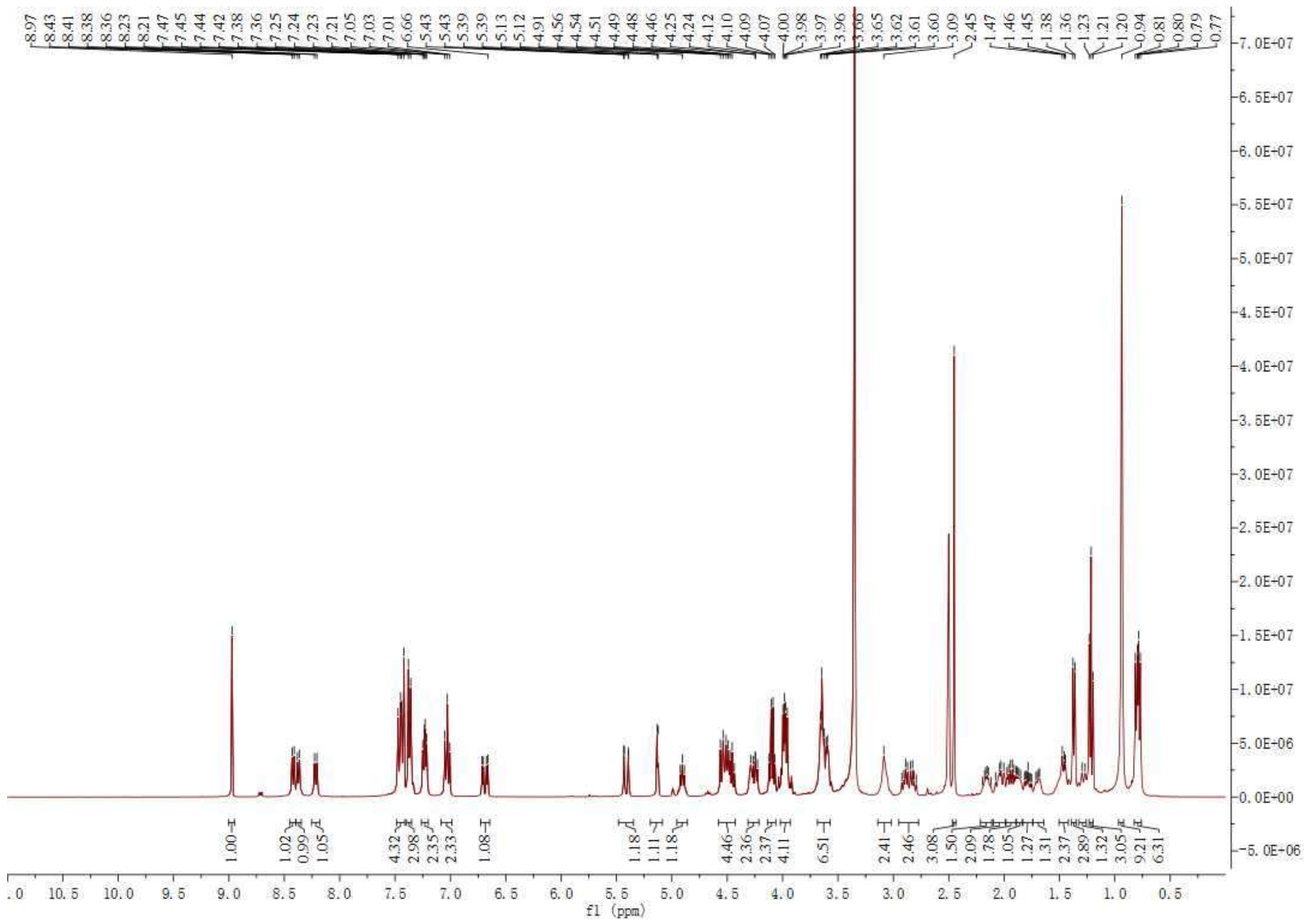
8.97 8.43 8.41 8.38 8.36 8.23 8.21 7.47 7.45 7.44 7.42 7.38 7.36 7.25 7.24 7.23 7.21 7.05 7.03 7.01 6.66 5.43 5.43 5.39 5.13 5.12 4.91 4.56 4.54 4.51 4.49 4.48 4.46 4.25 4.24 4.12 4.10 4.09 4.07 4.00 3.98 3.97 3.96 3.66 3.65 3.62 3.61 3.60 3.09 2.45 1.47 1.46 1.45 1.38 1.36 1.23 1.21 1.20 0.94 0.81 0.80 0.79 0.77
1.00 1.02 0.99 1.05 4.32 2.98 2.35 2.33 1.08 1.18 1.11 1.18 4.46 2.36 2.37 4.11 6.51 2.41 2.46 3.08 1.50 2.09 1.78 1.05 1.27 1.31 2.37 2.89 1.32 3.05 9.21 6.31
f1 (ppm)

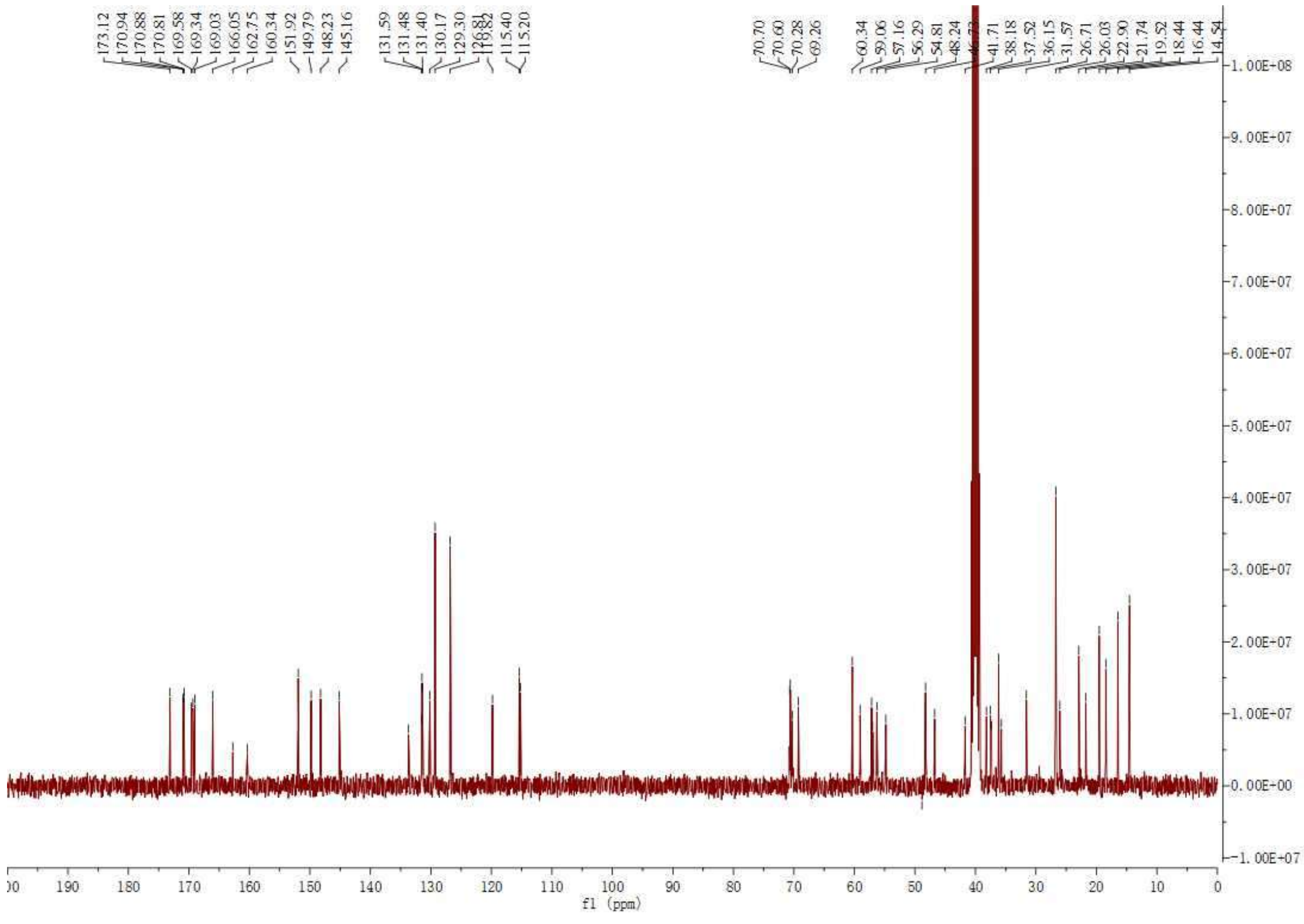
173.12 170.94 170.88 170.81 169.58 169.34 169.03 166.05 162.75 160.34 151.92 149.79 148.23 145.16 131.59 131.48 131.40 130.17 129.30 126.81 119.82 115.40 115.20 70.70 70.60 70.28 69.26 60.34 59.06 57.16 56.29 54.81 48.24 41.71 38.18 37.52 36.15 31.57 26.71 26.03 22.90 21.74 19.52 18.44 16.44 14.54
f1 (ppm)

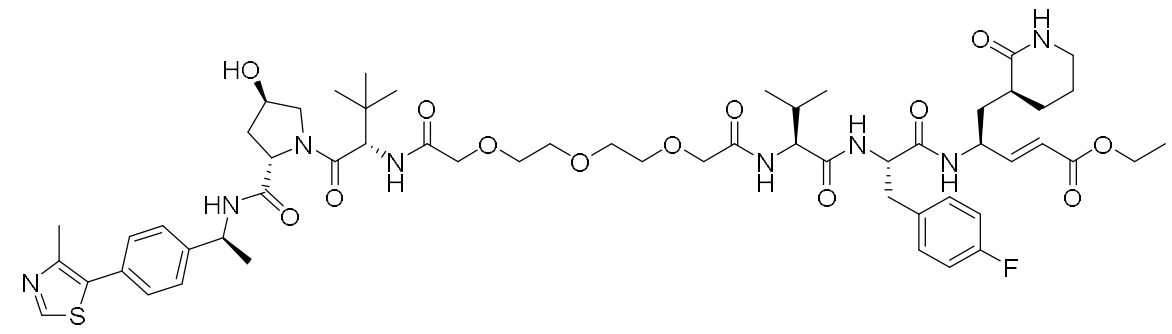

D5

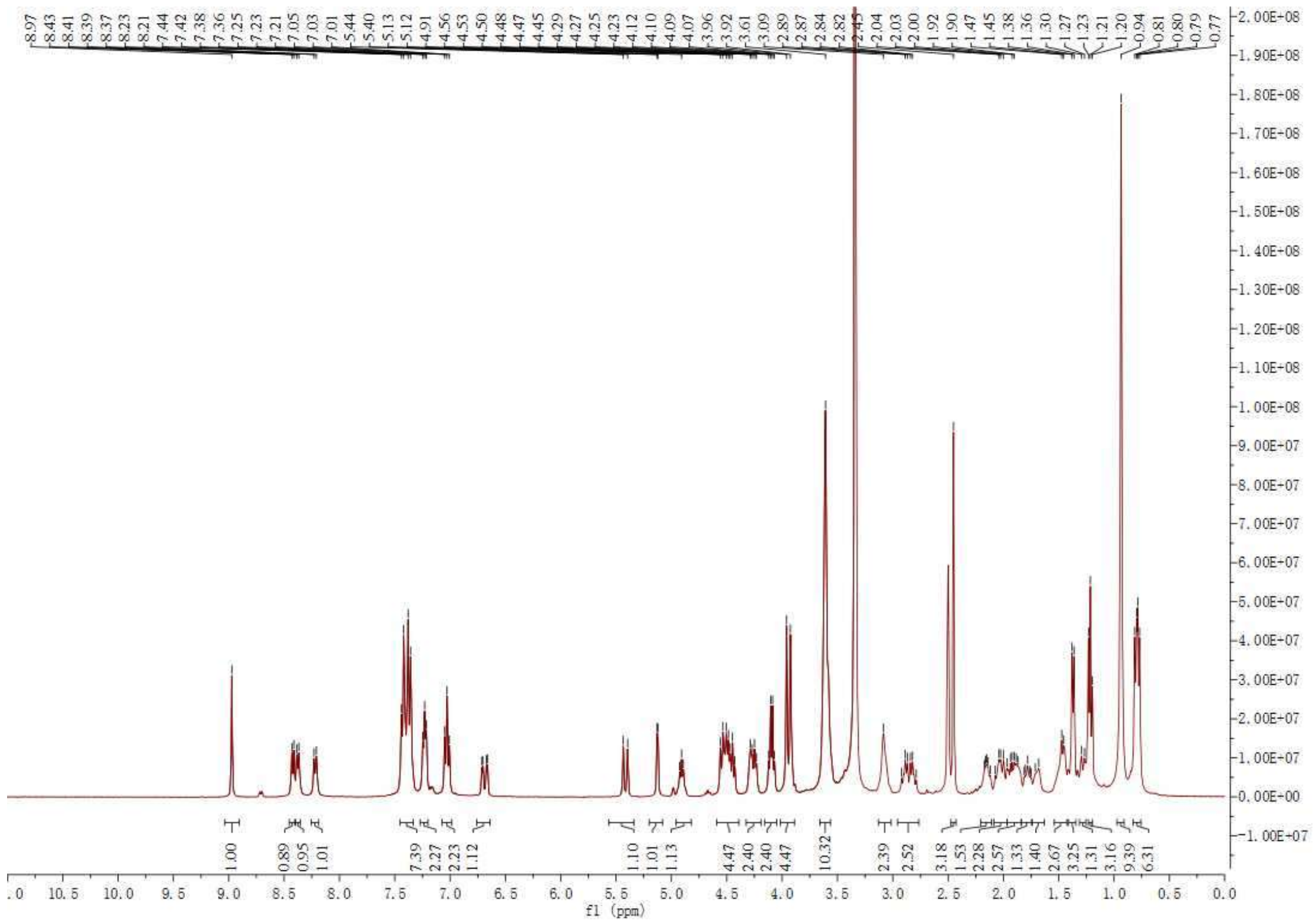

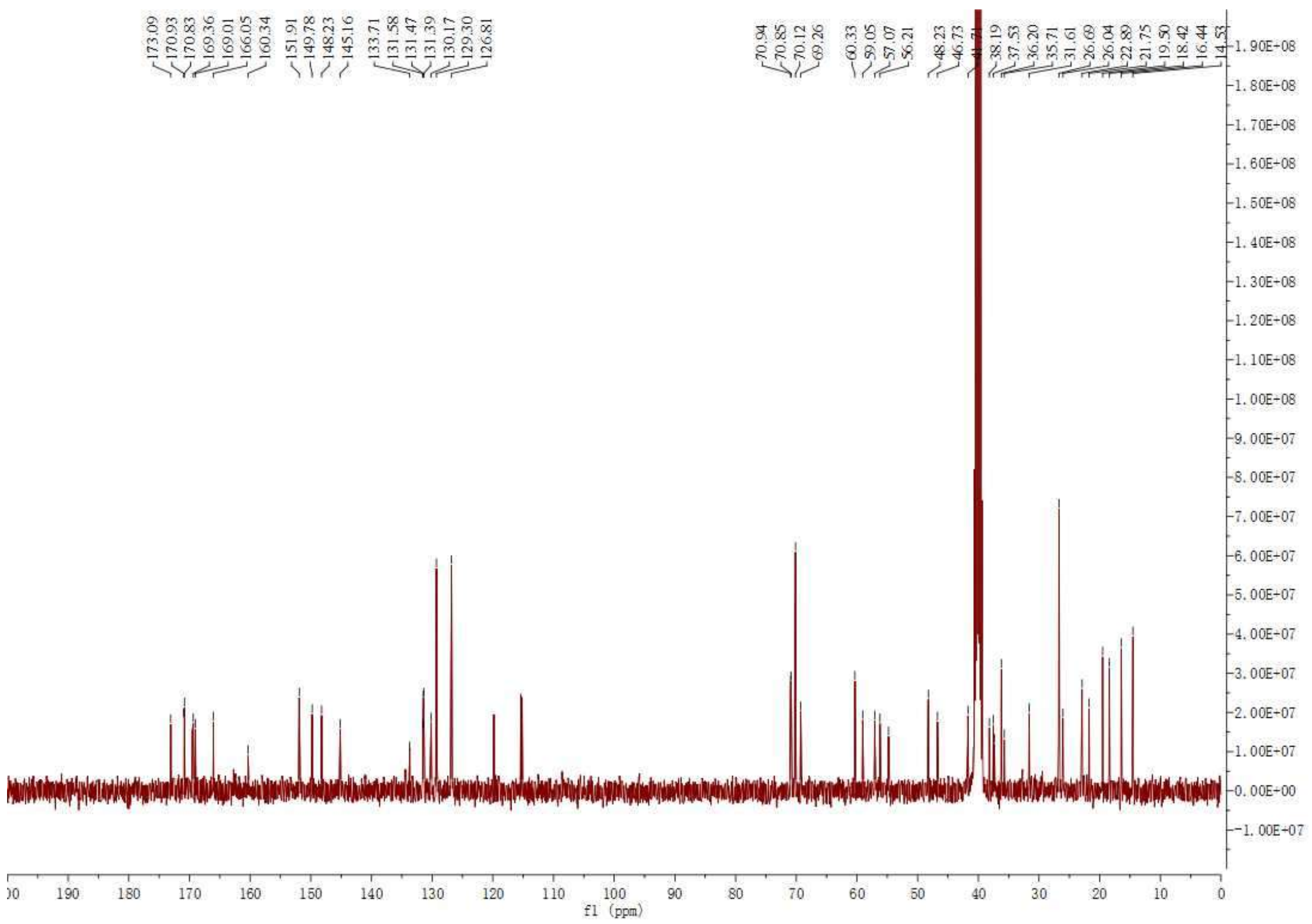

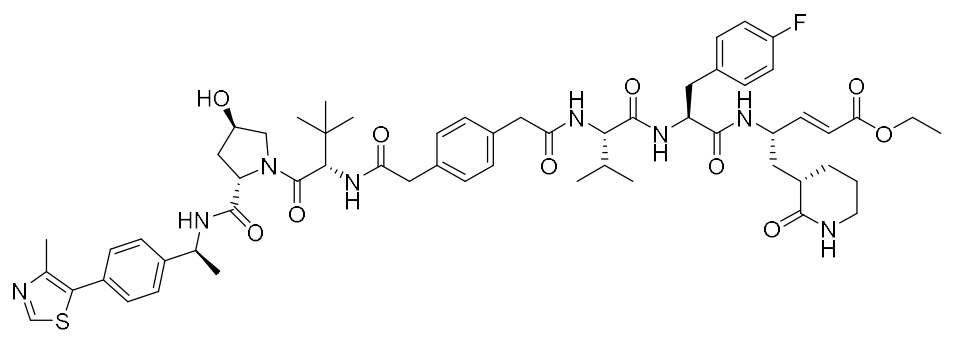

D6

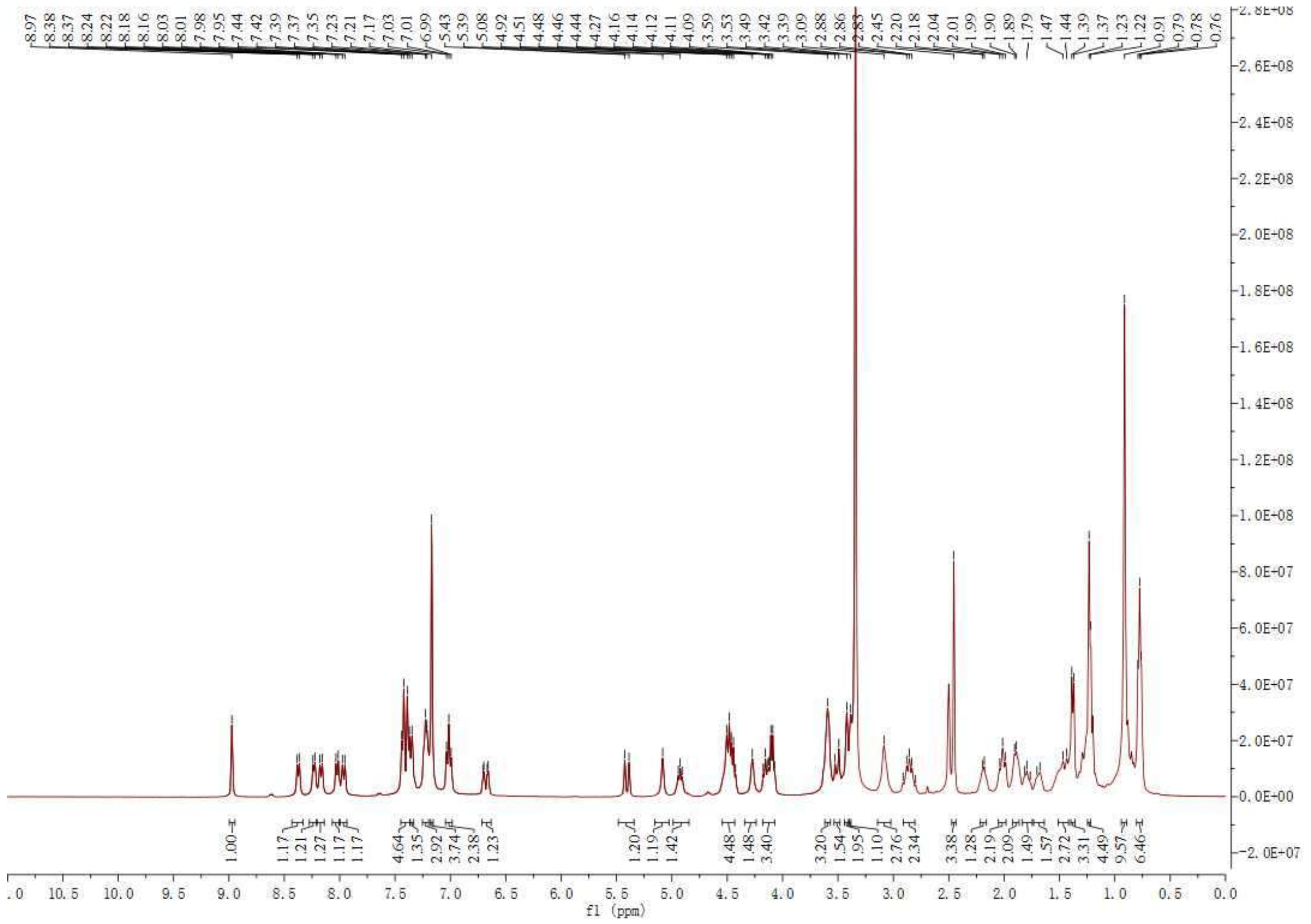

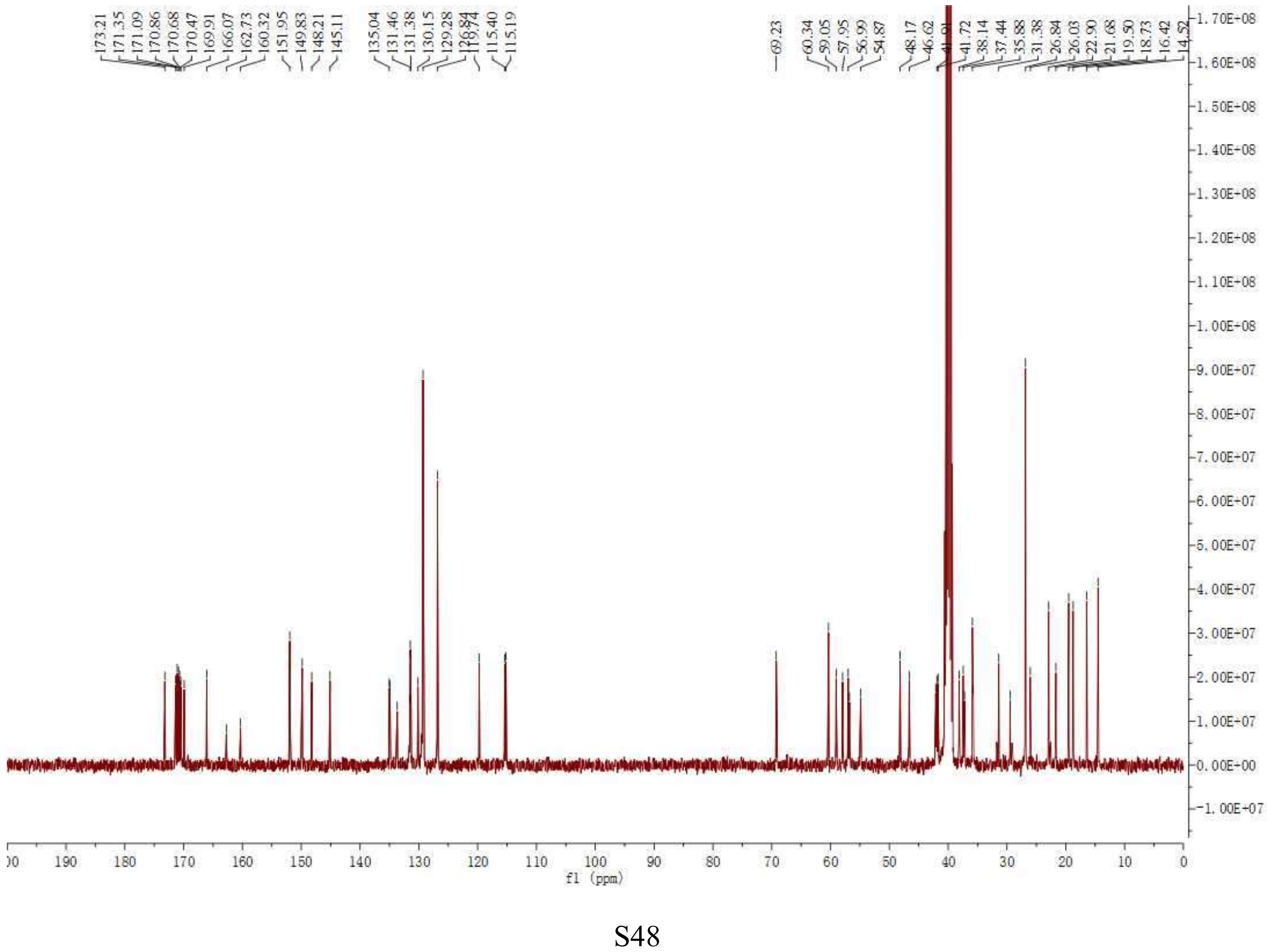

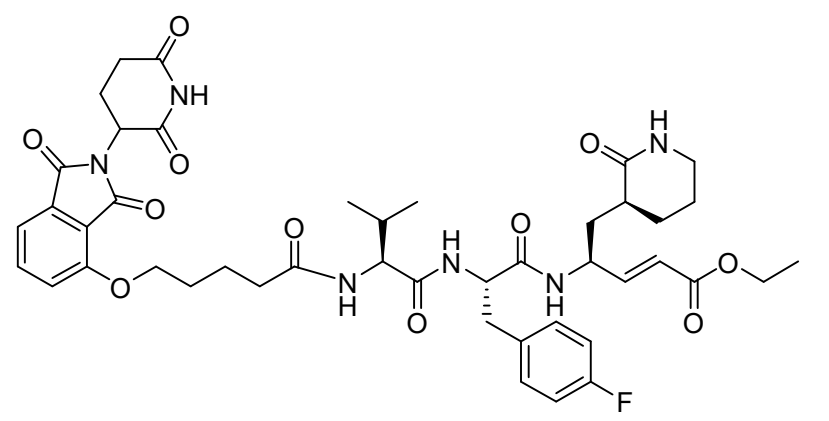

D7

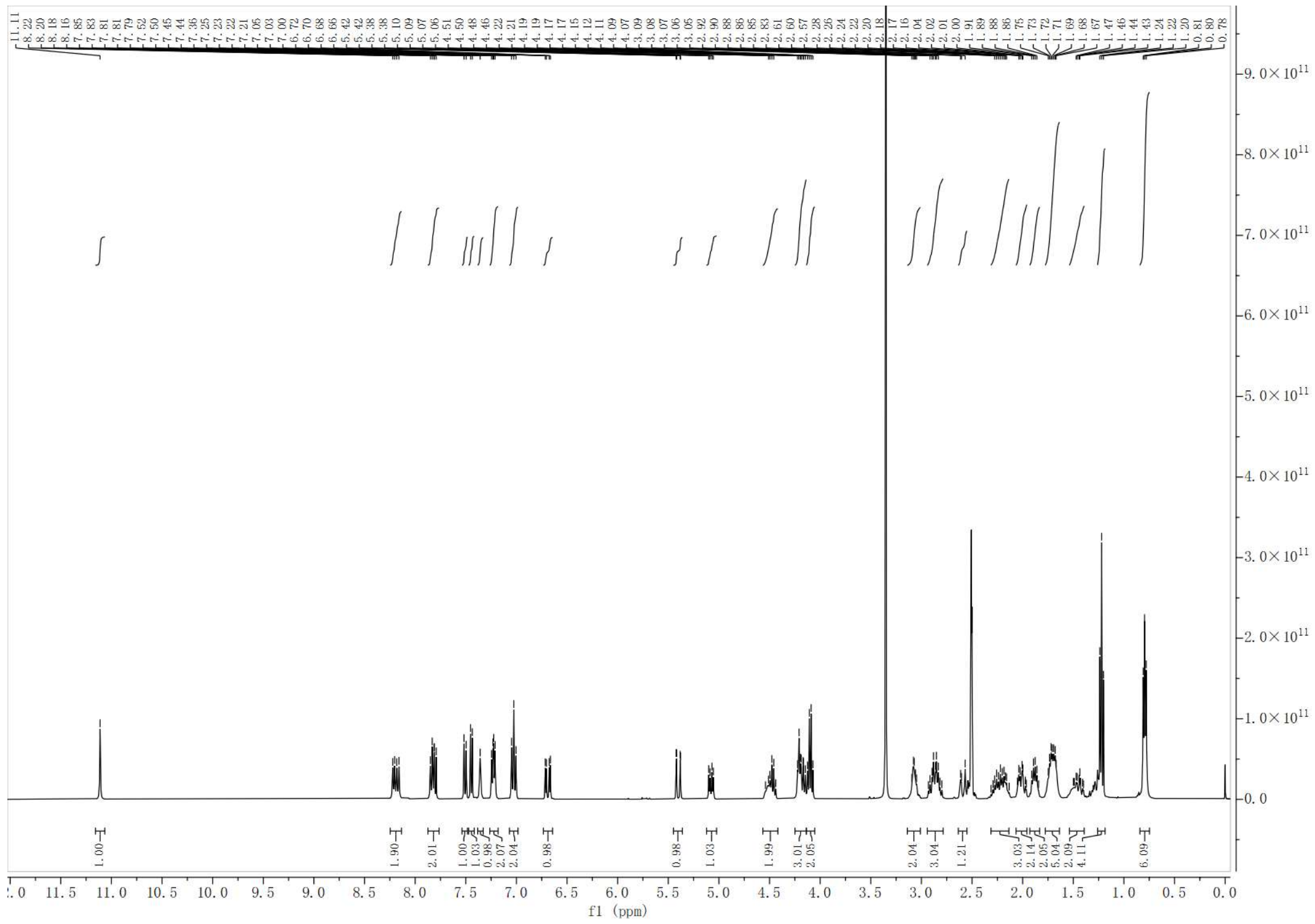

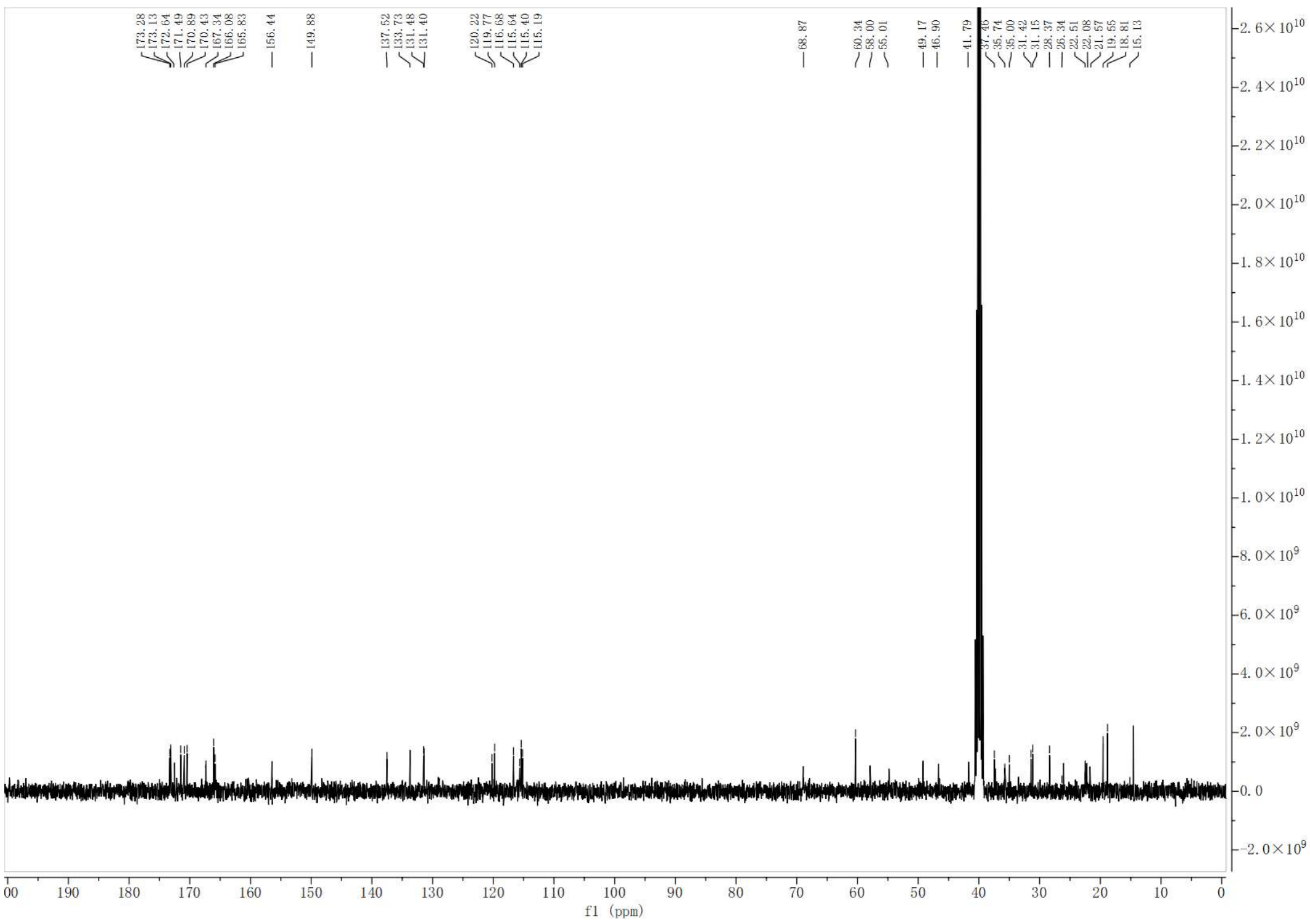

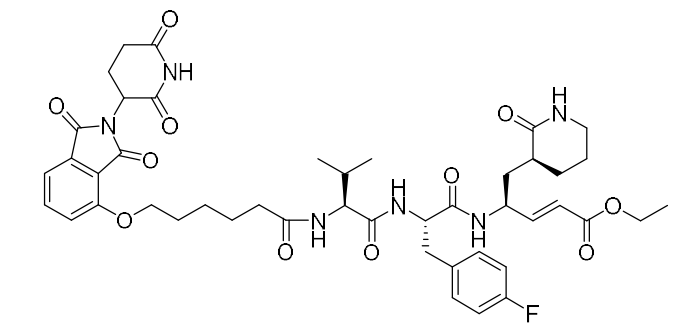

D8

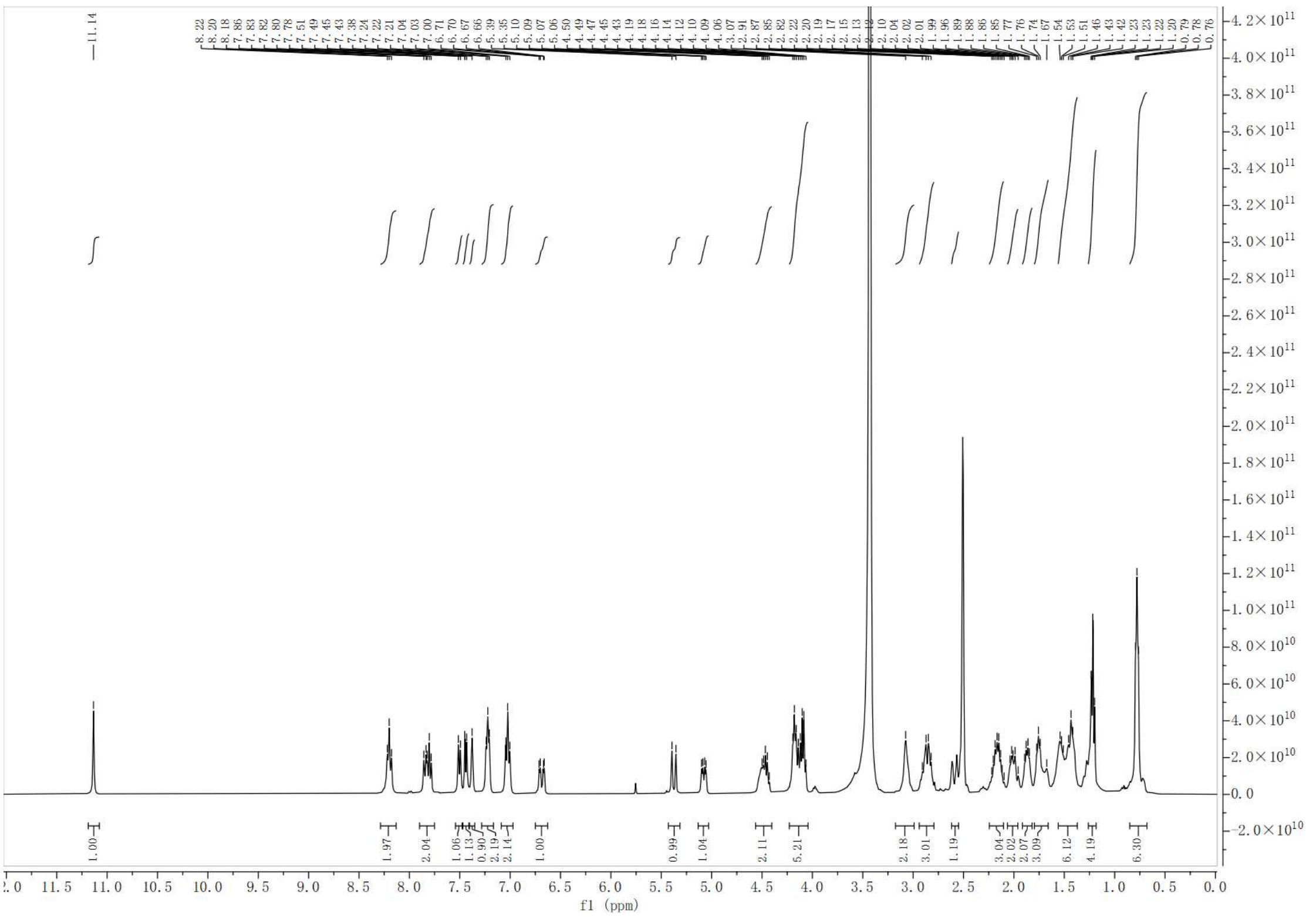

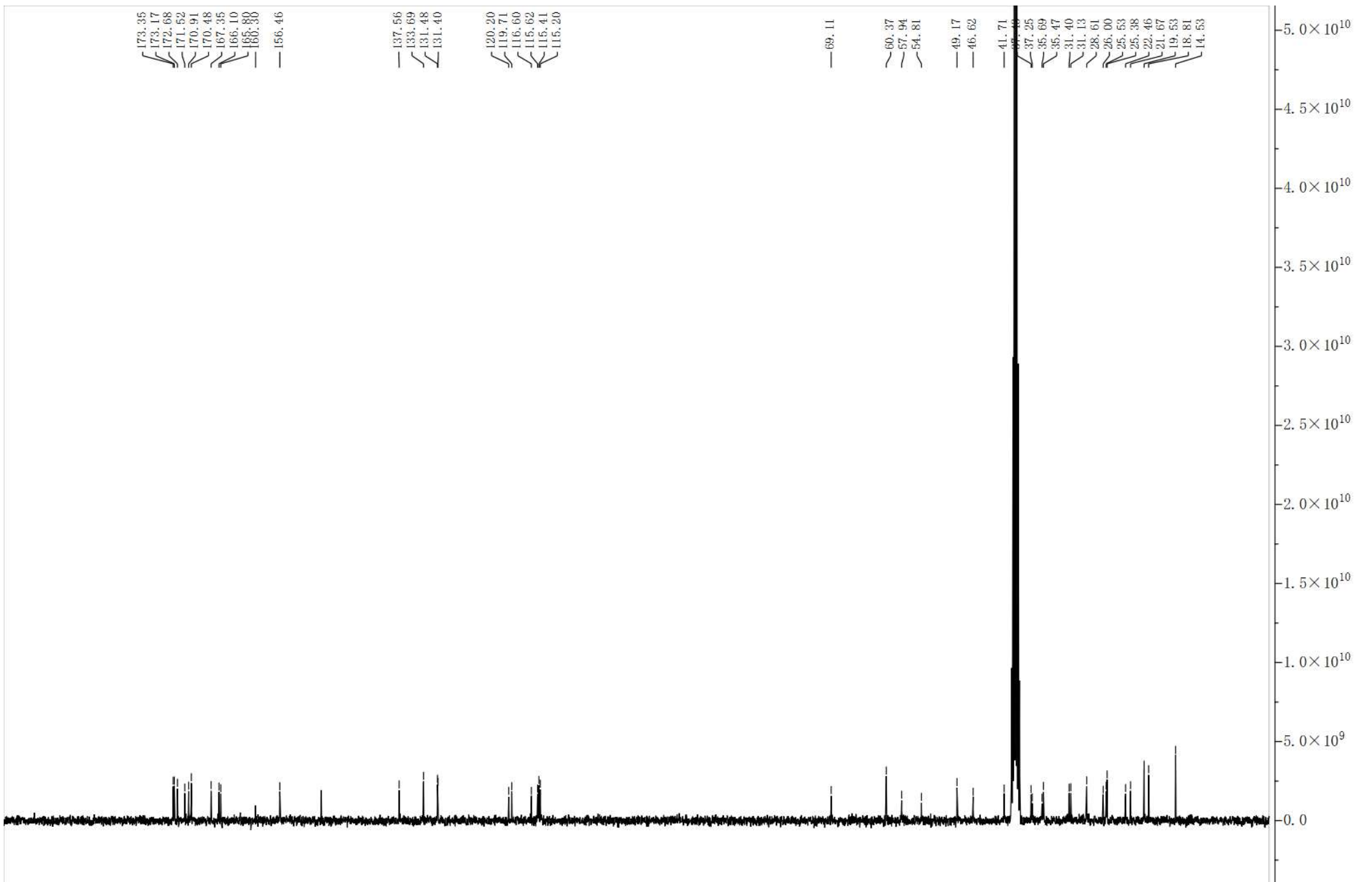

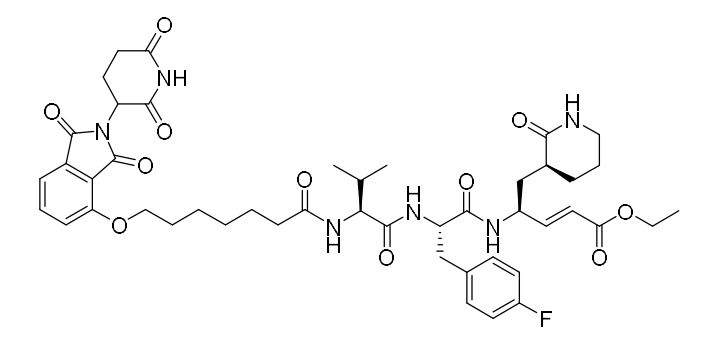

D9

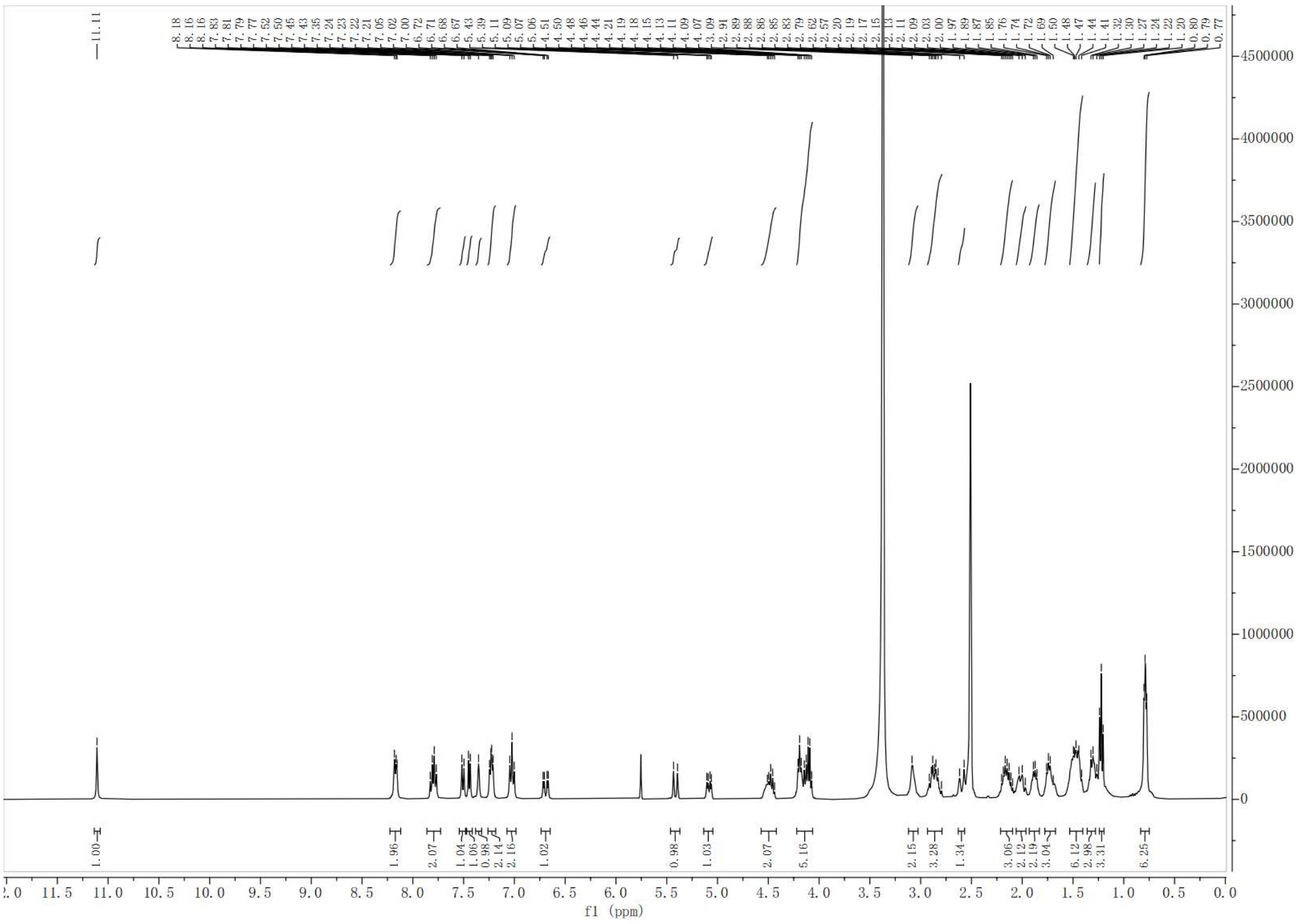
f1 (ppm)
12.0 11.5 11.0 10.5 10.0 9.5 9.0 8.5 8.0 7.5 7.0 6.5 6.0 5.5 5.0 4.5 4.0 3.5 3.0 2.5 2.0 1.5 1.0 0.5 0.0
4500000 4000000 3500000 3000000 2500000 2000000 1500000 1000000 500000 0

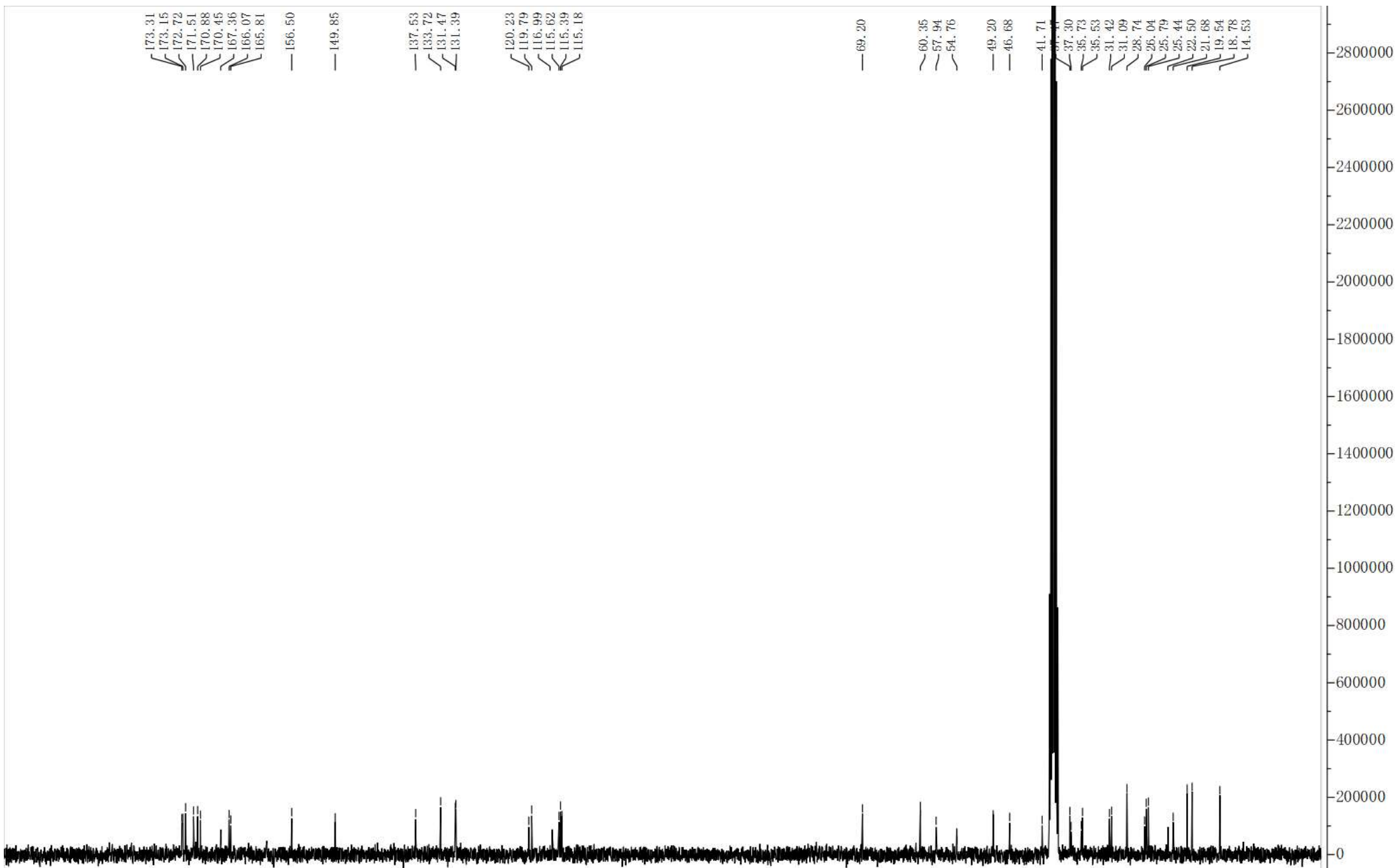
f1 (ppm)
200 190 180 170 160 150 140 130 120 110 100 90 80 70 60 50 40 30 20 10 0
2800000 2600000 2400000 2200000 2000000 1800000 1600000 1400000 1200000 1000000 800000 600000 400000 200000 0 -200000

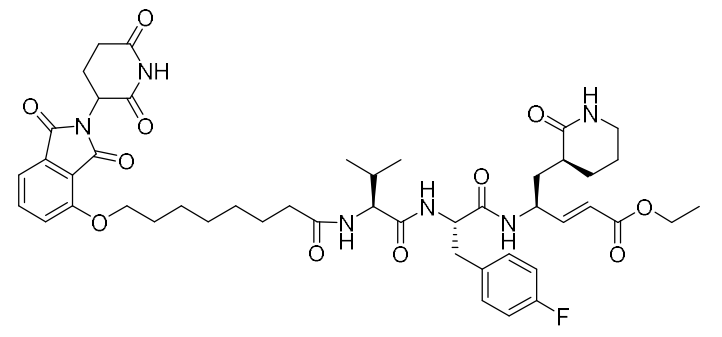

D10

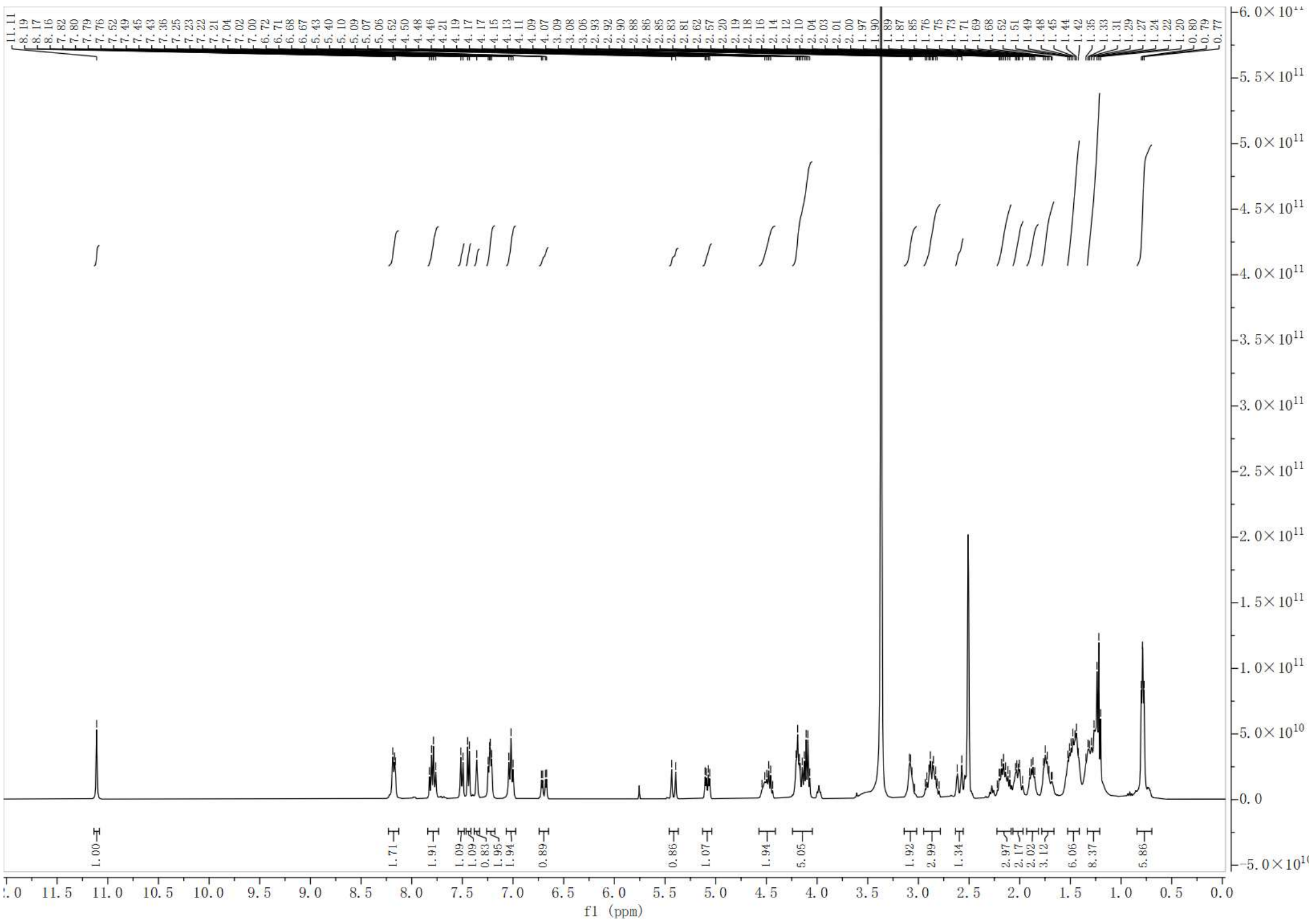

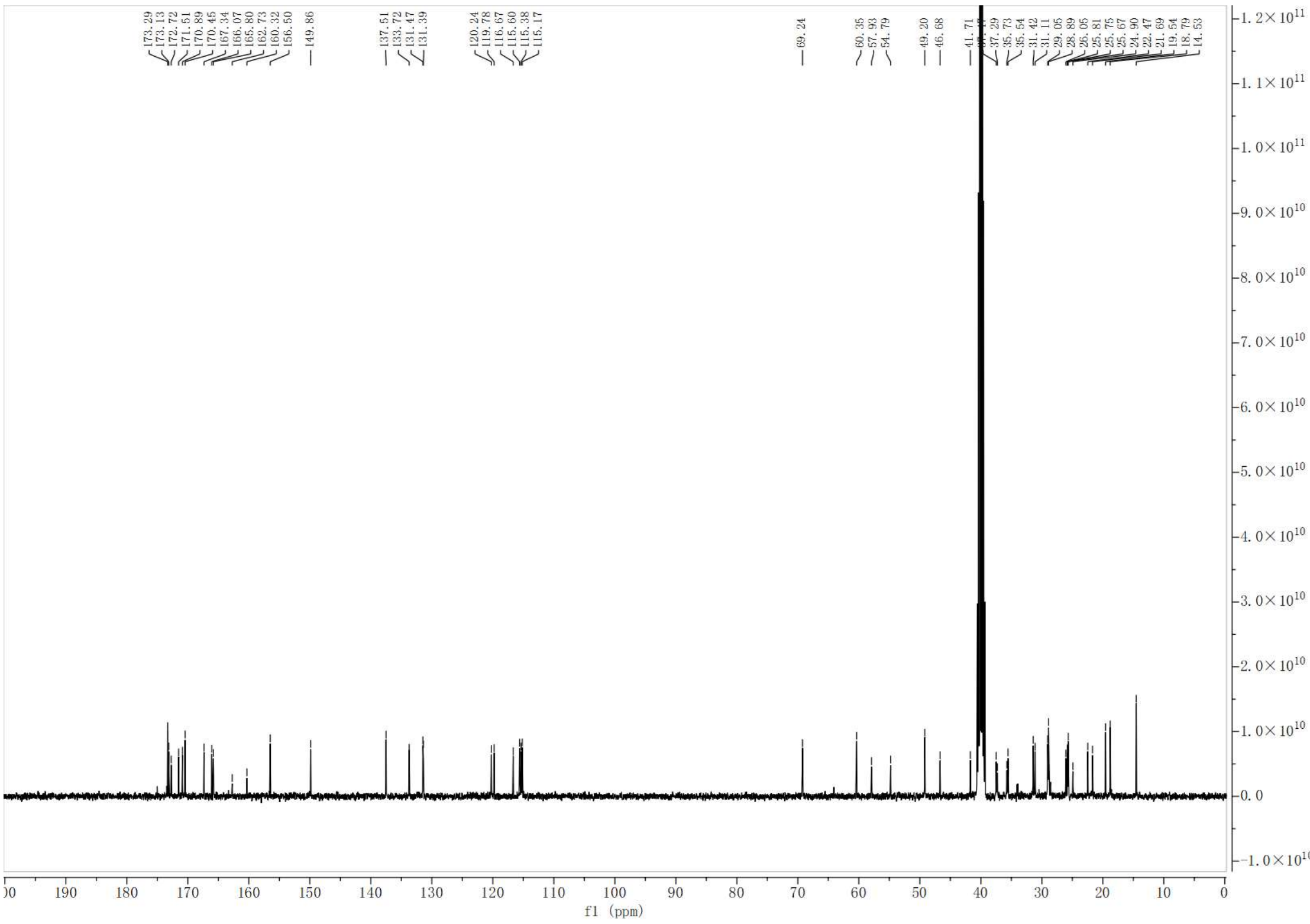

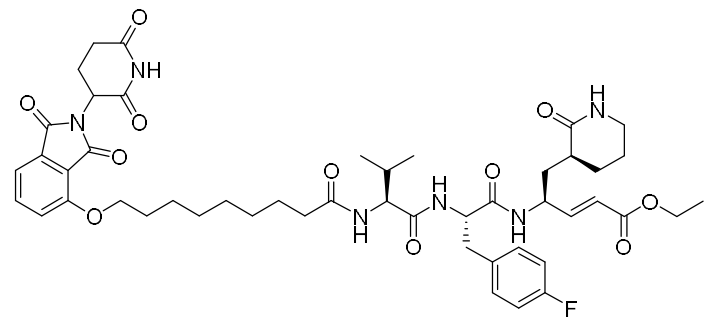

D11

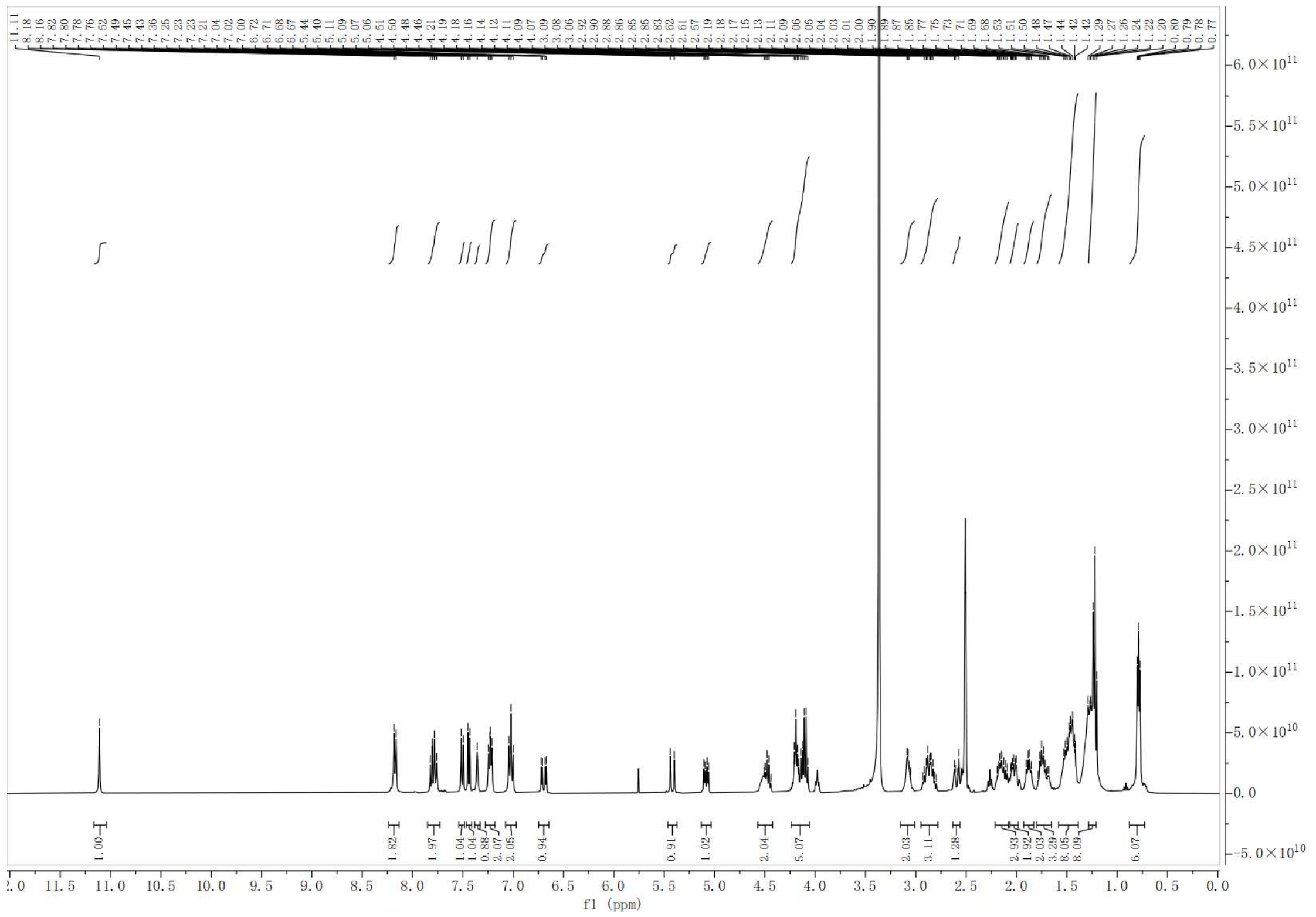

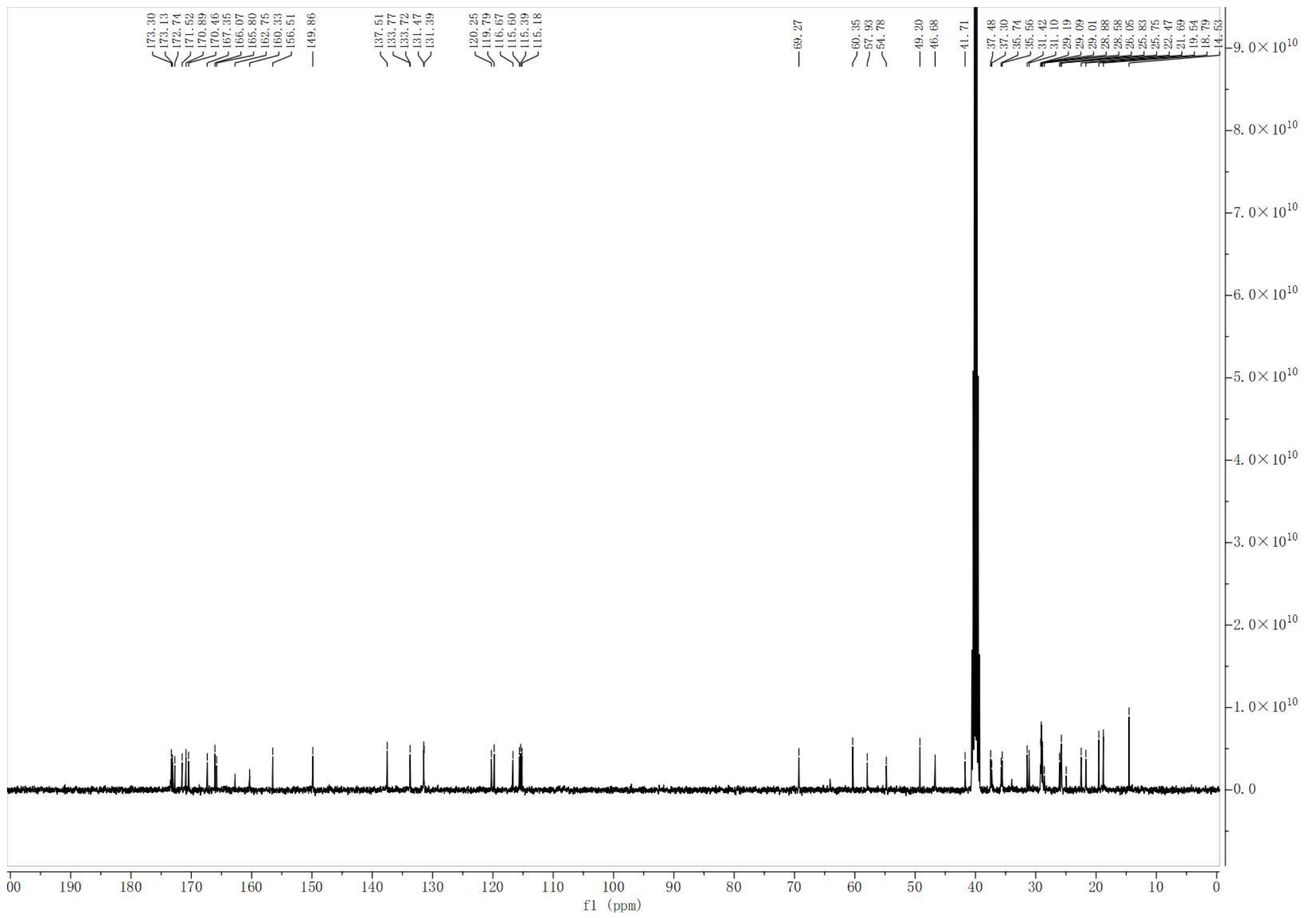

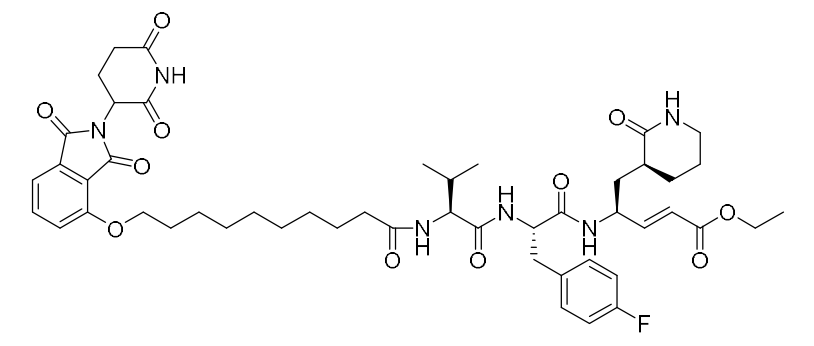

D12

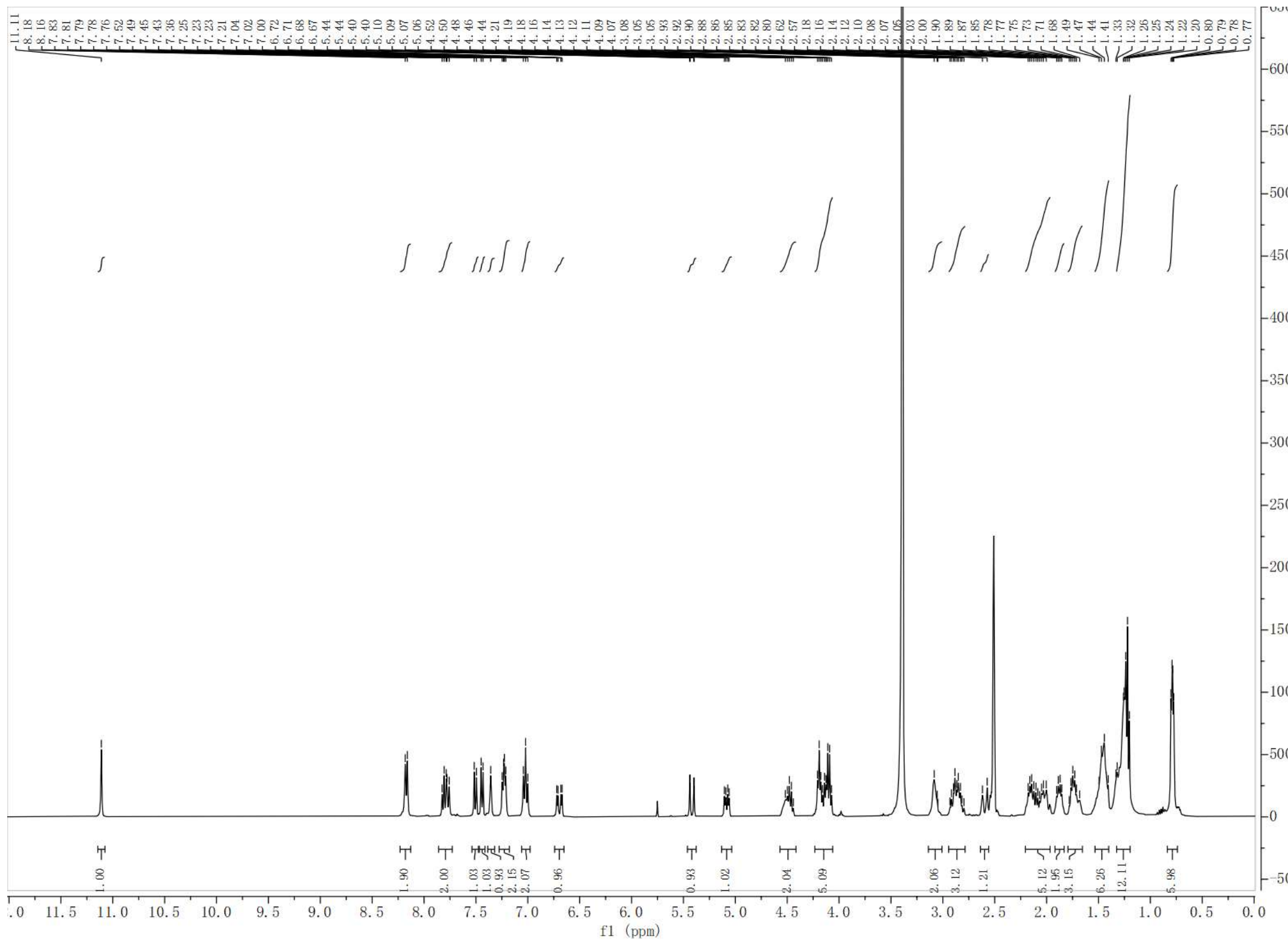

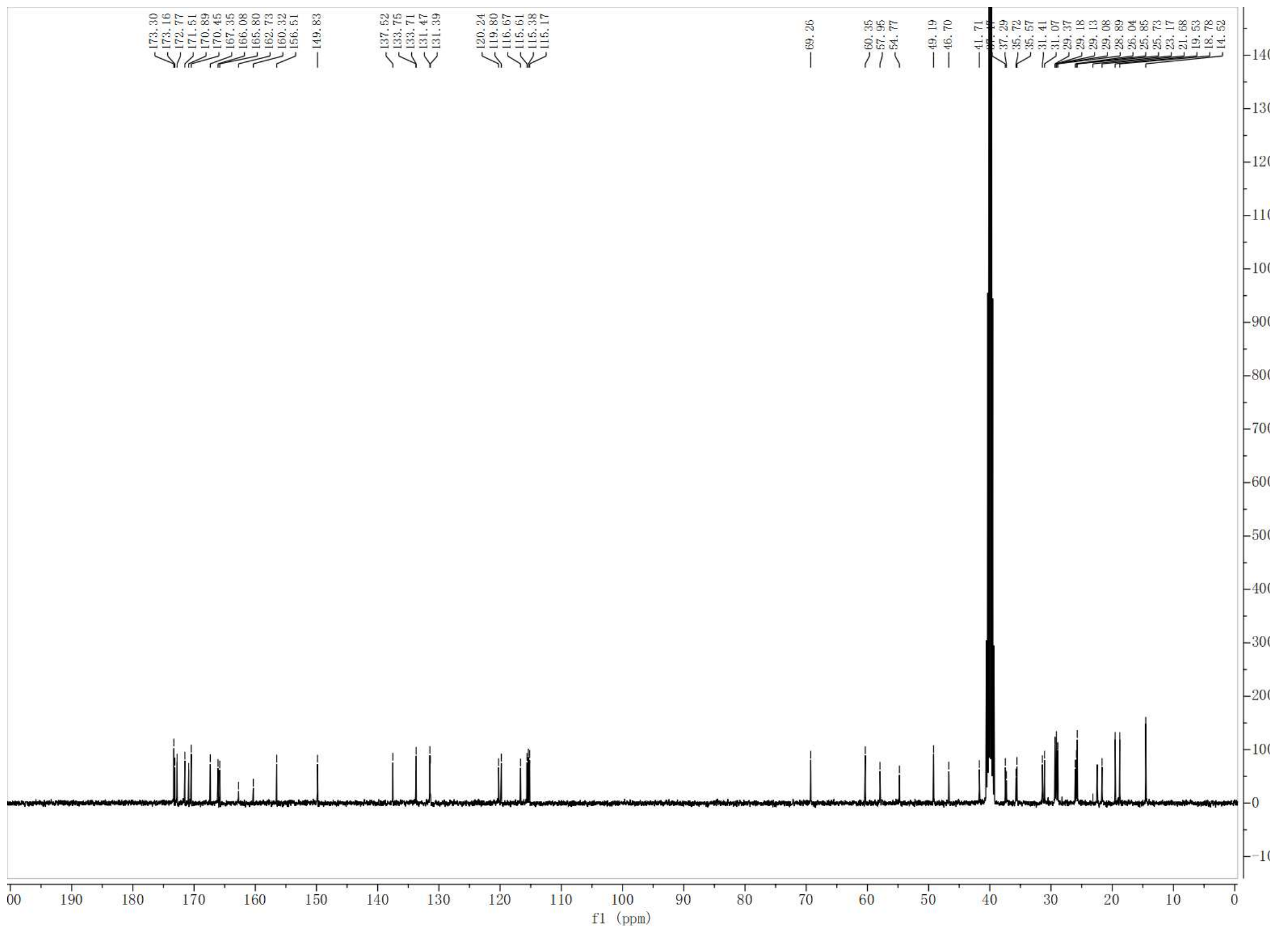

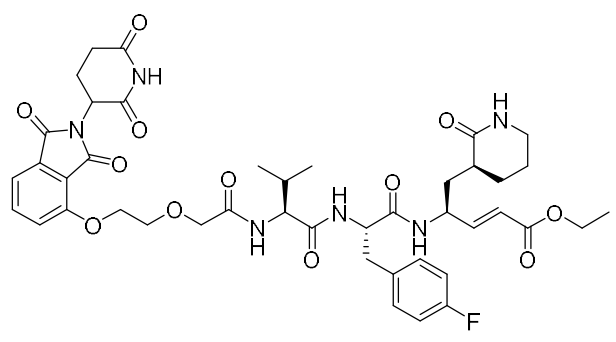

D13

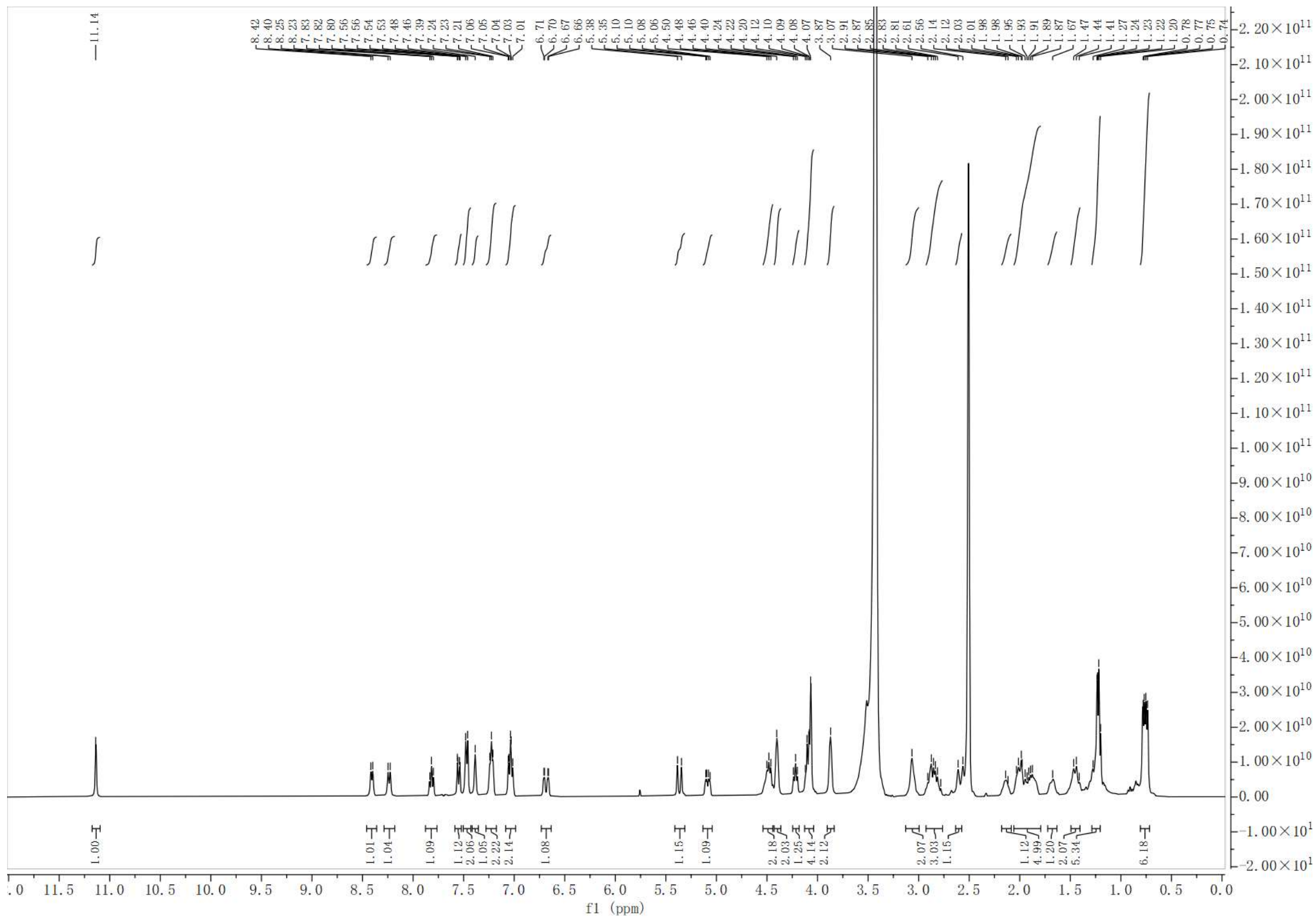

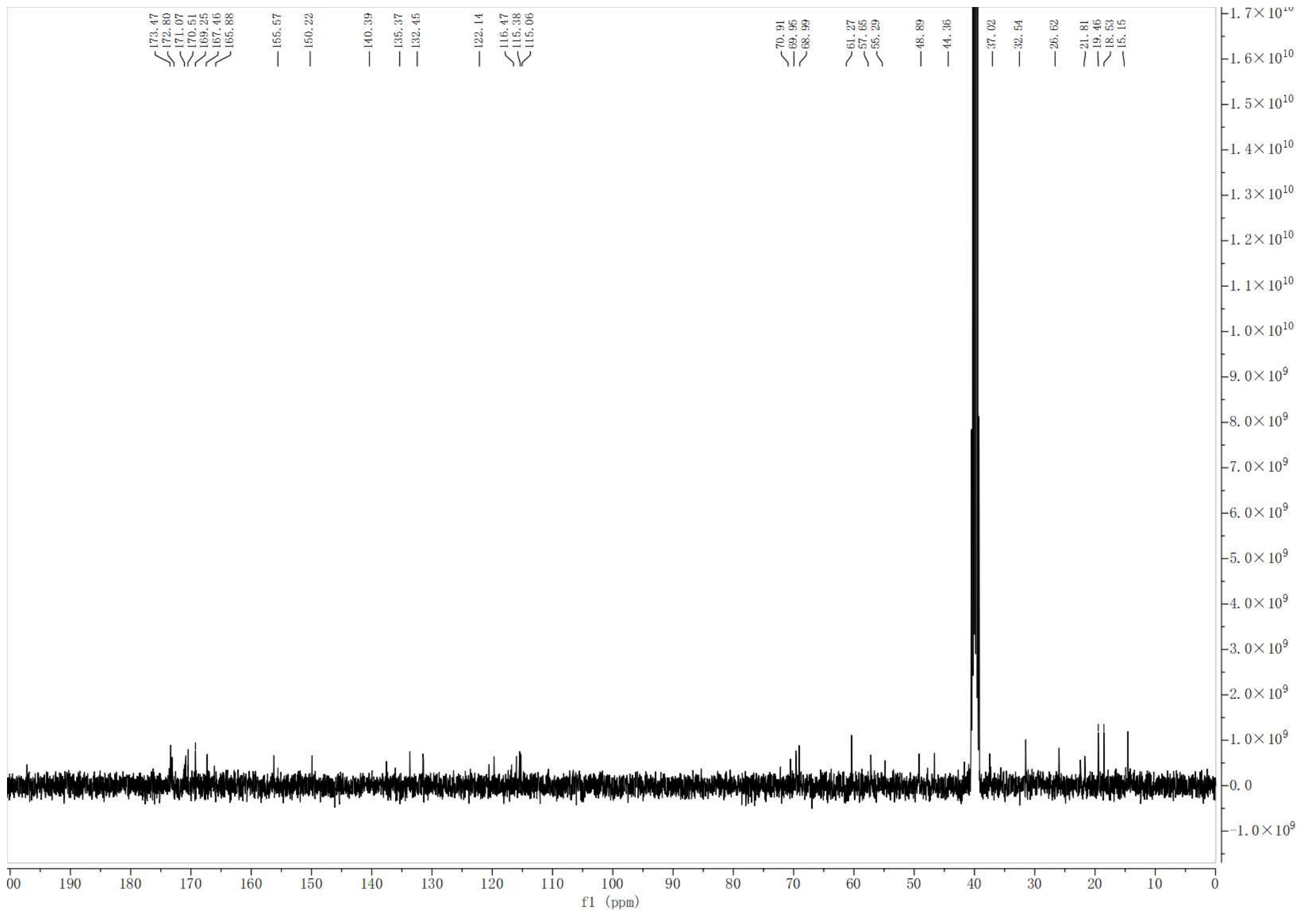

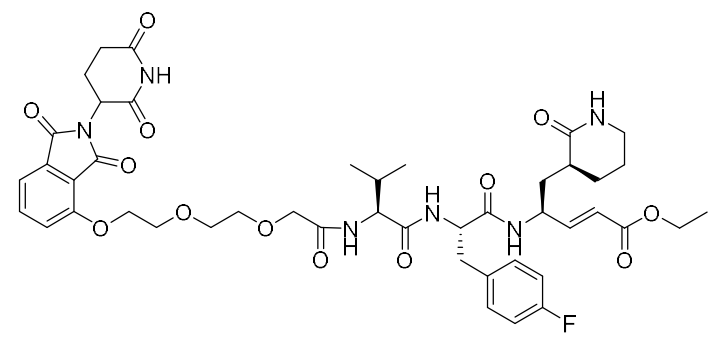

D14

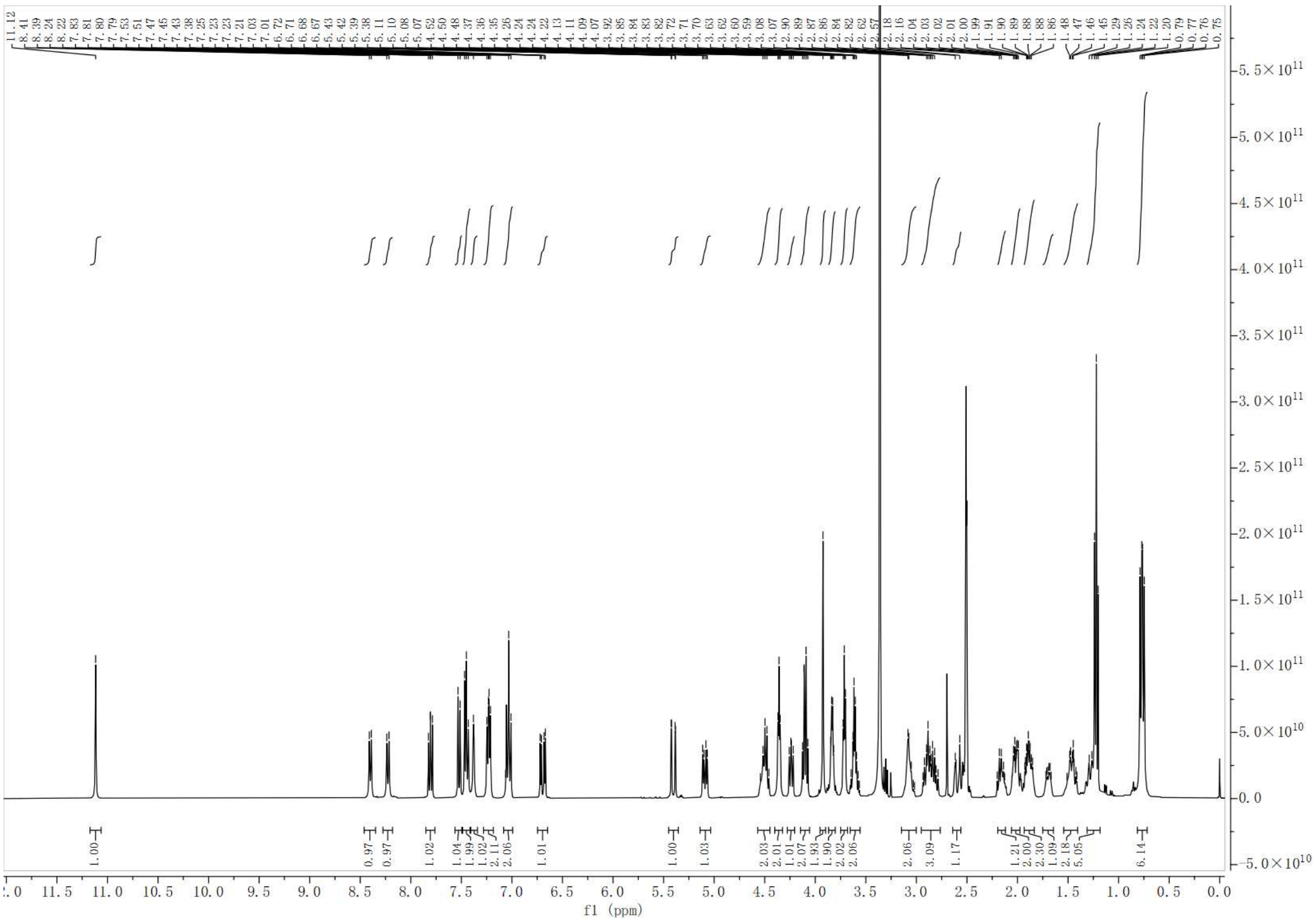

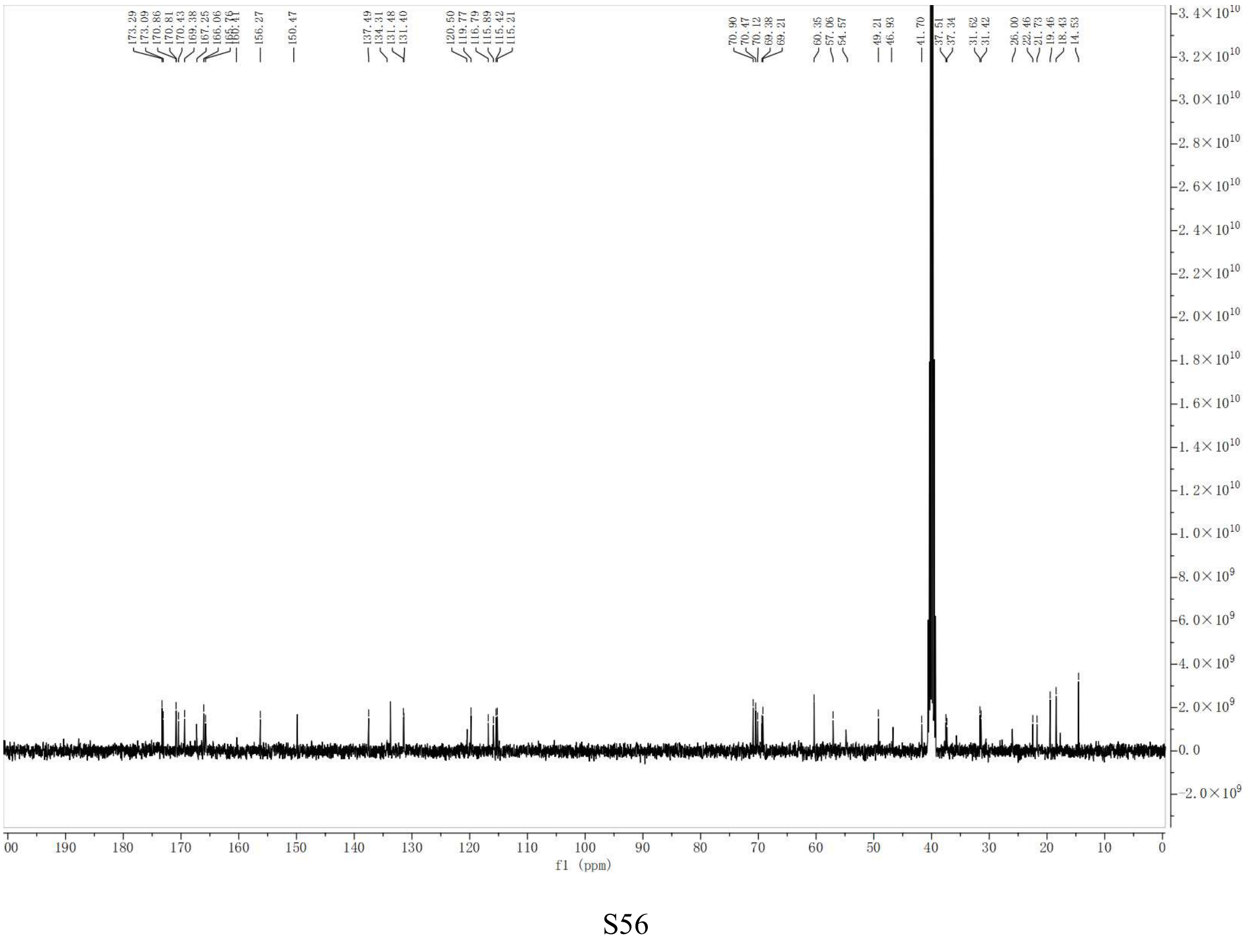

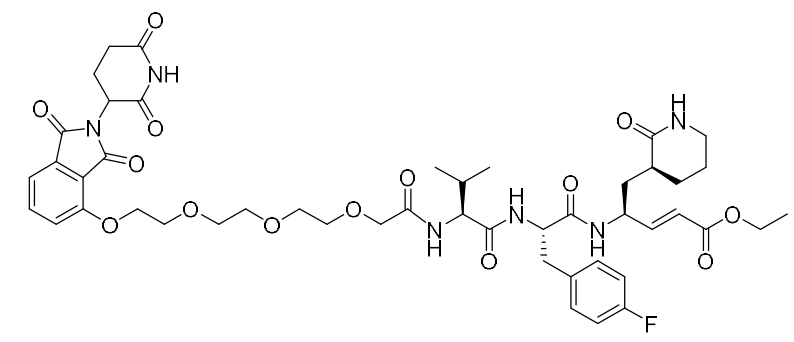

D15

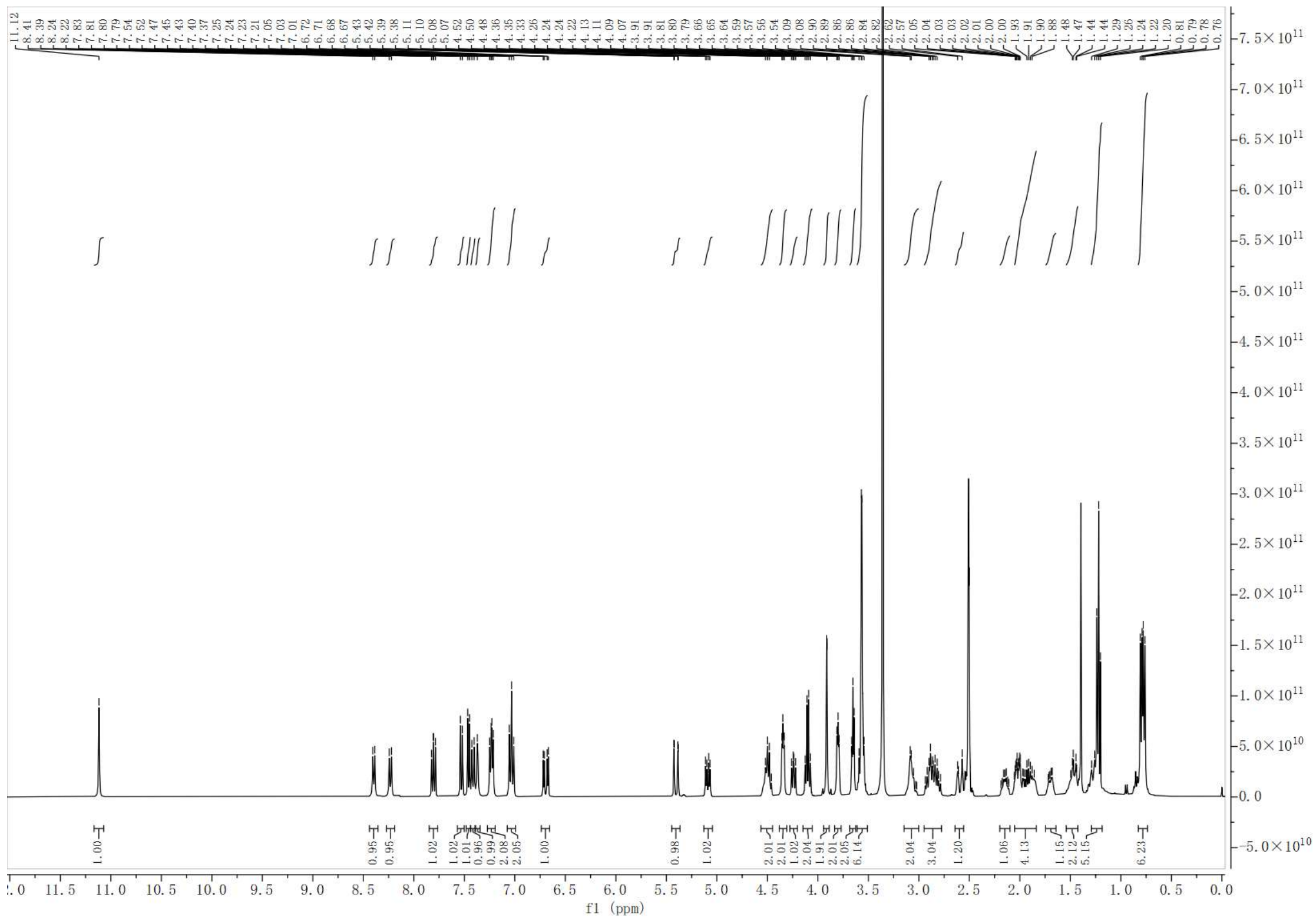

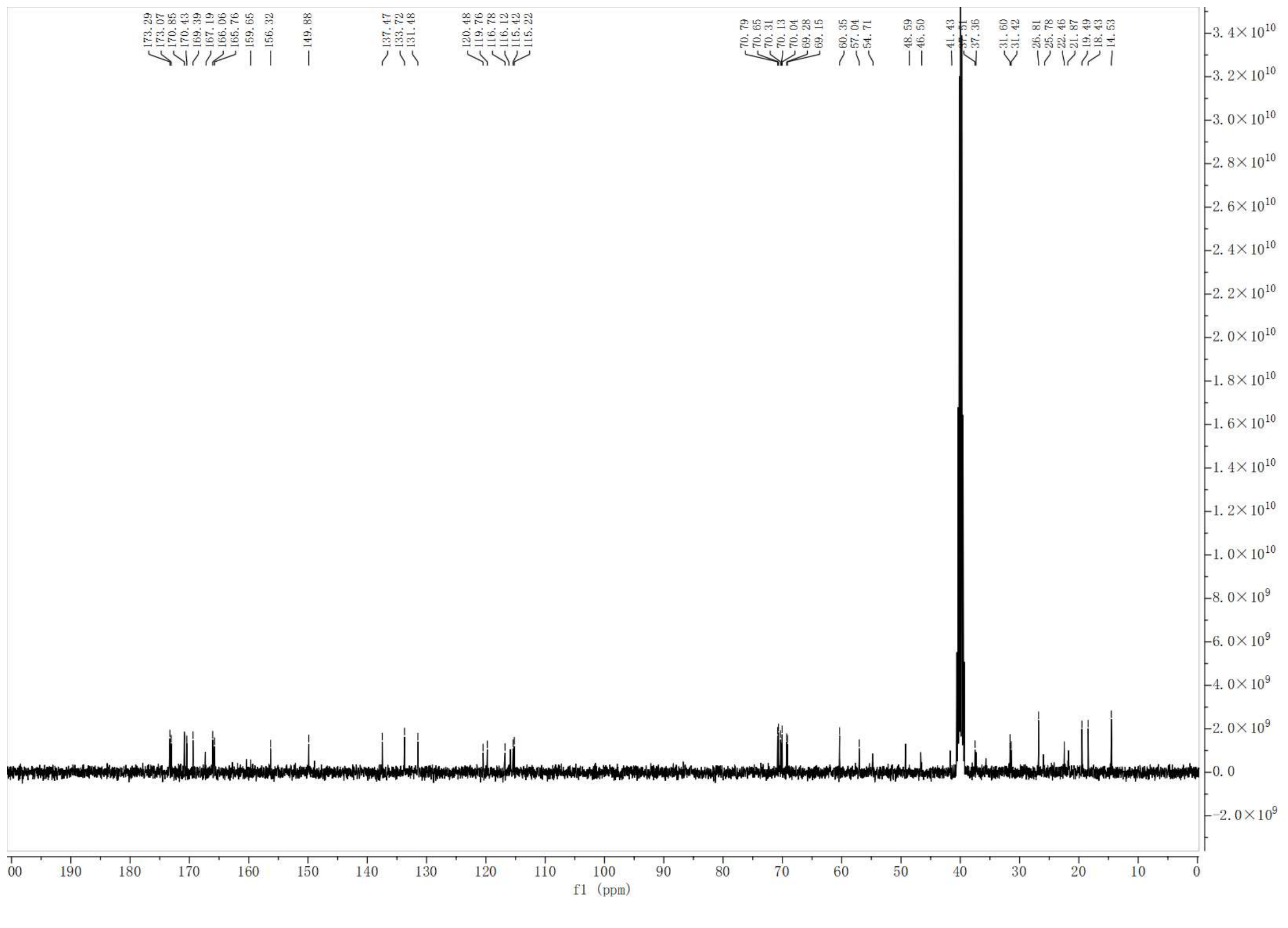

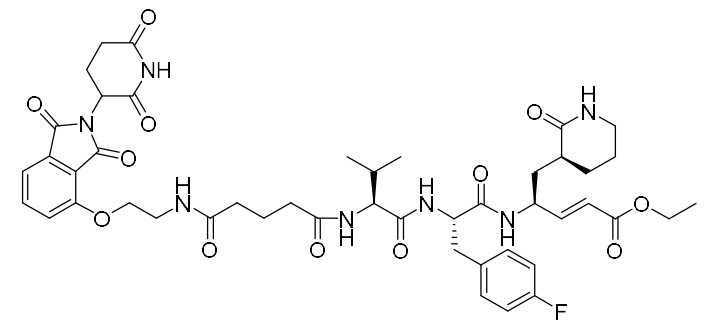

D16

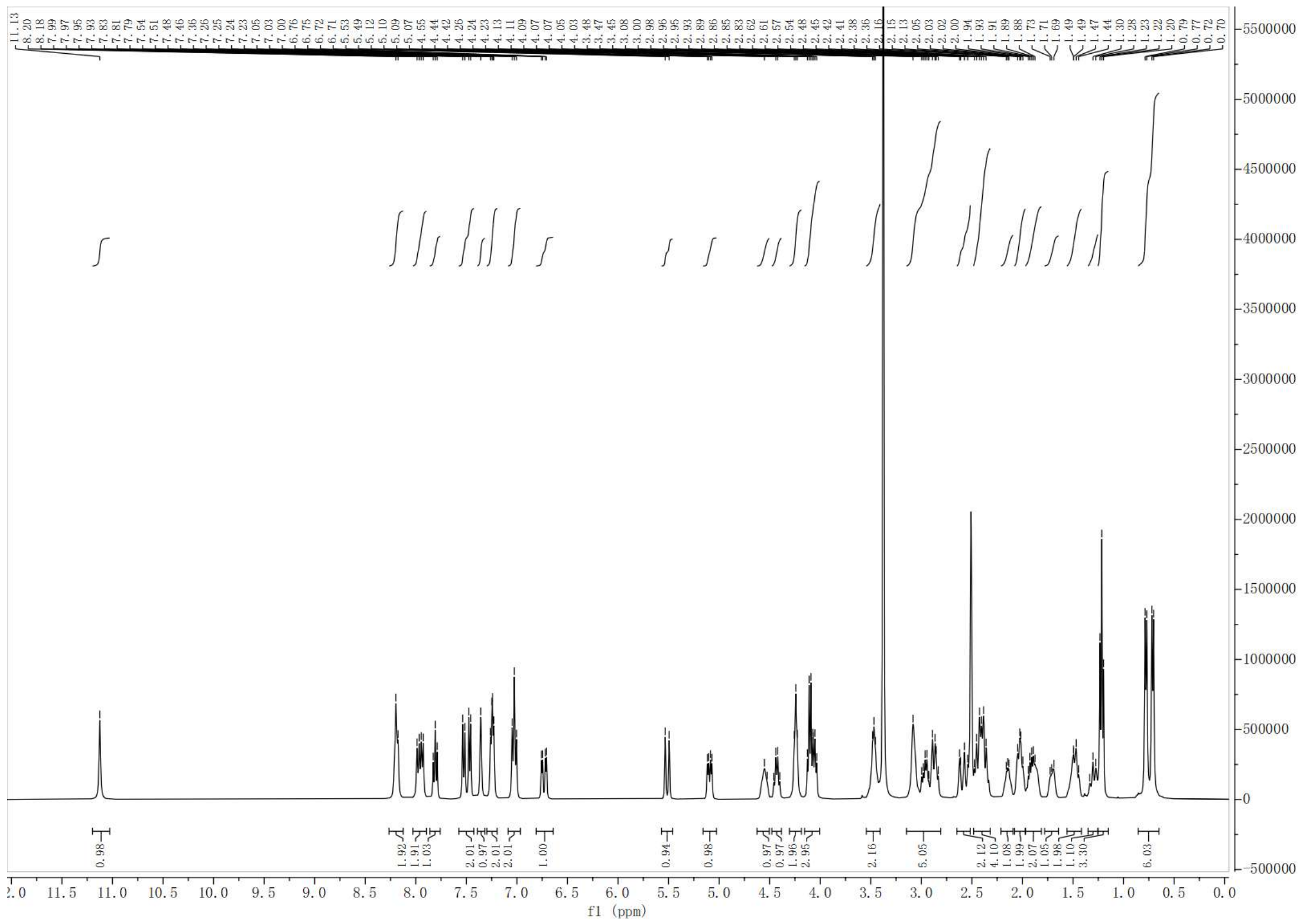

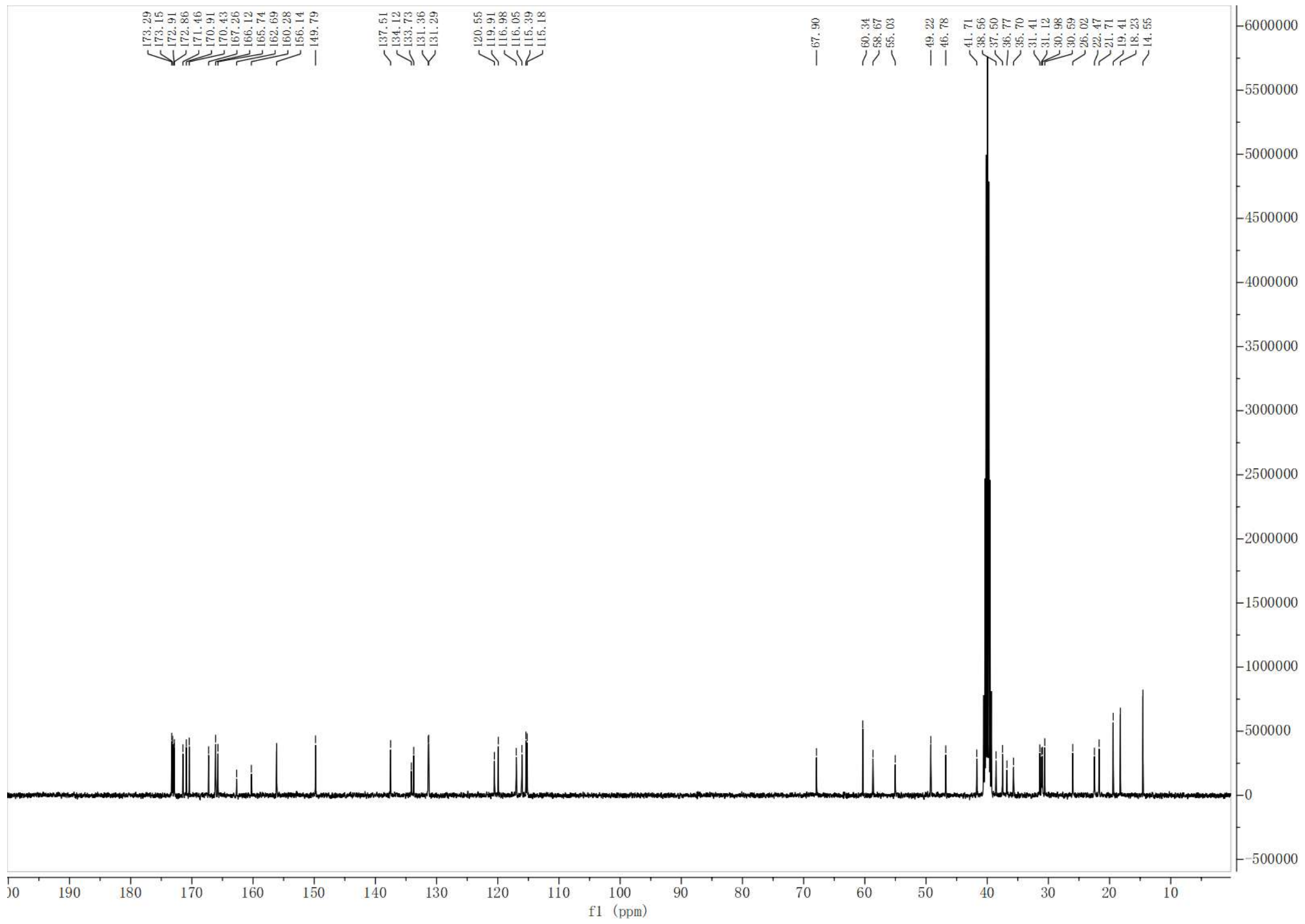

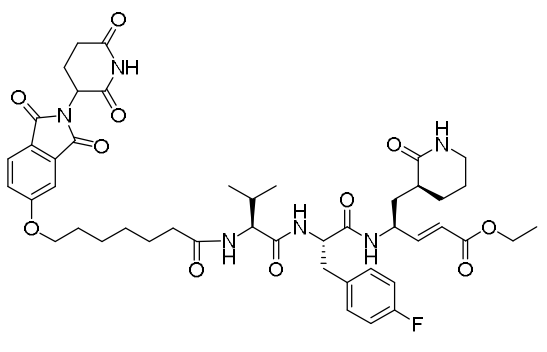

D17

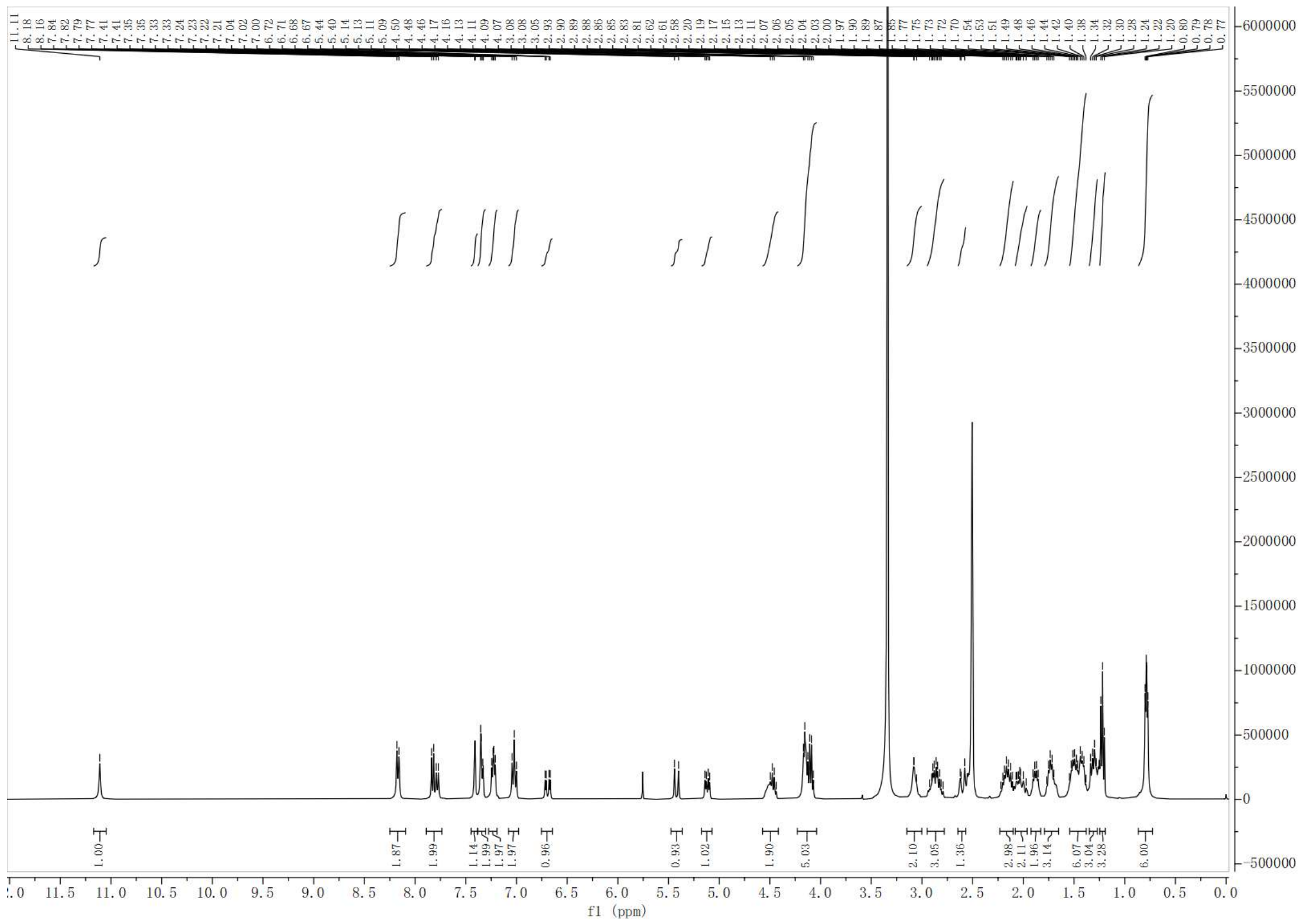

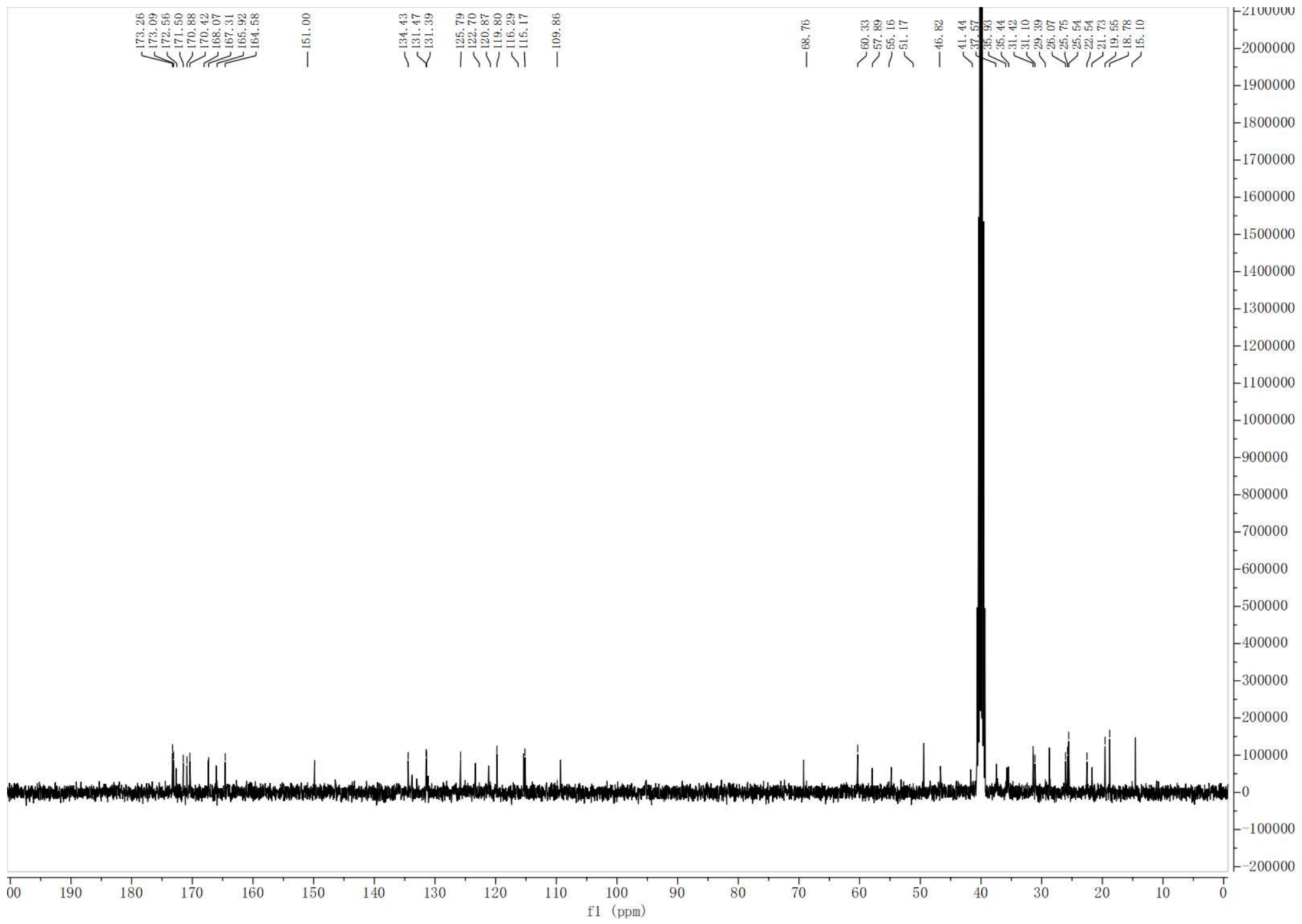

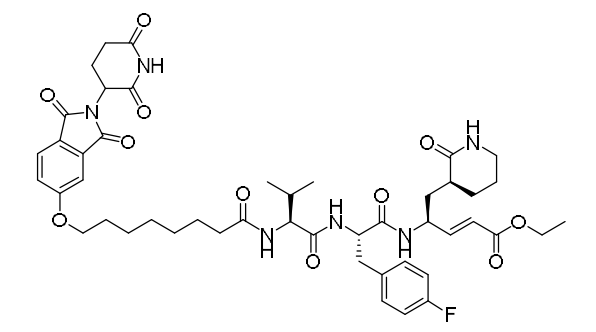

D18

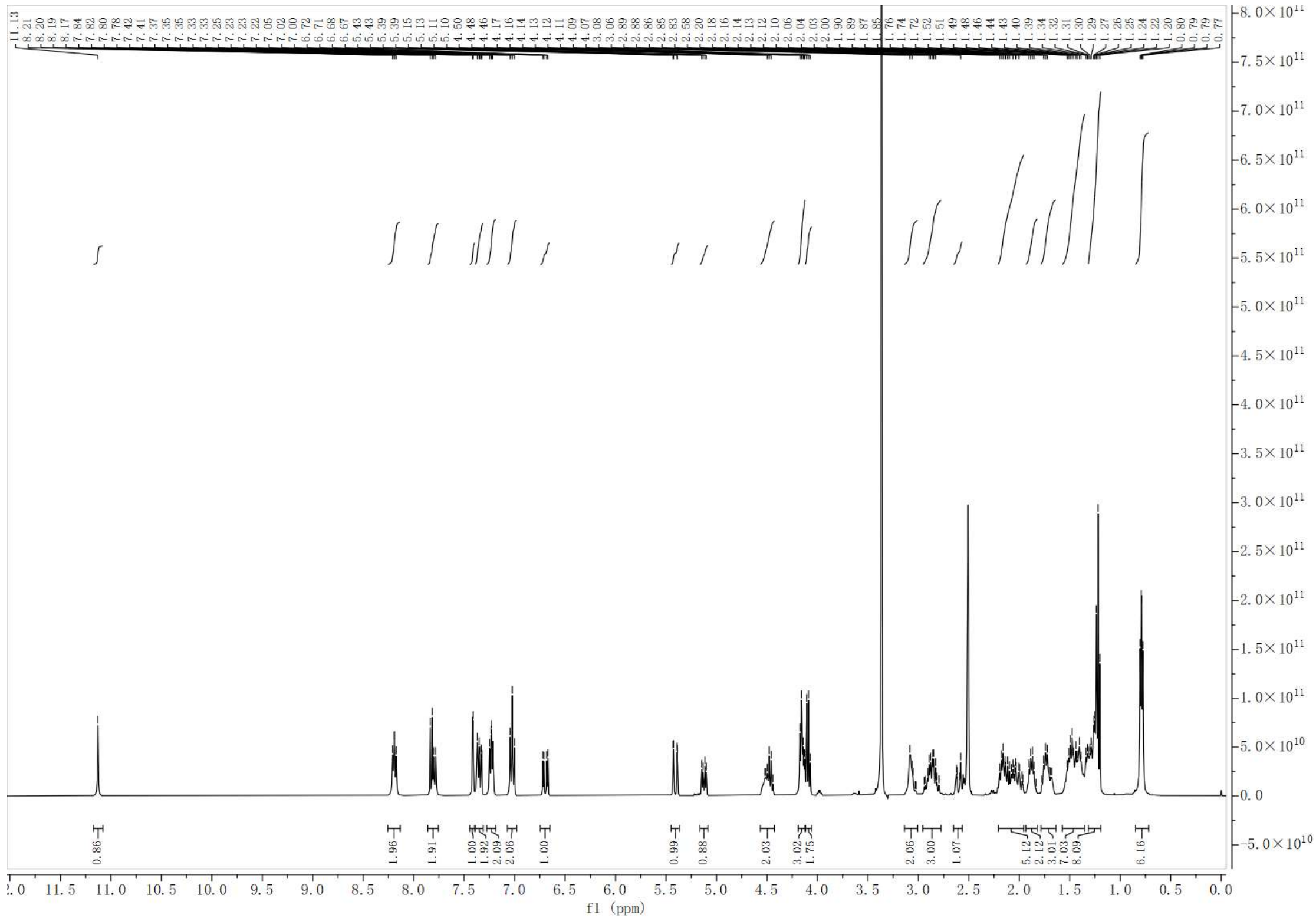

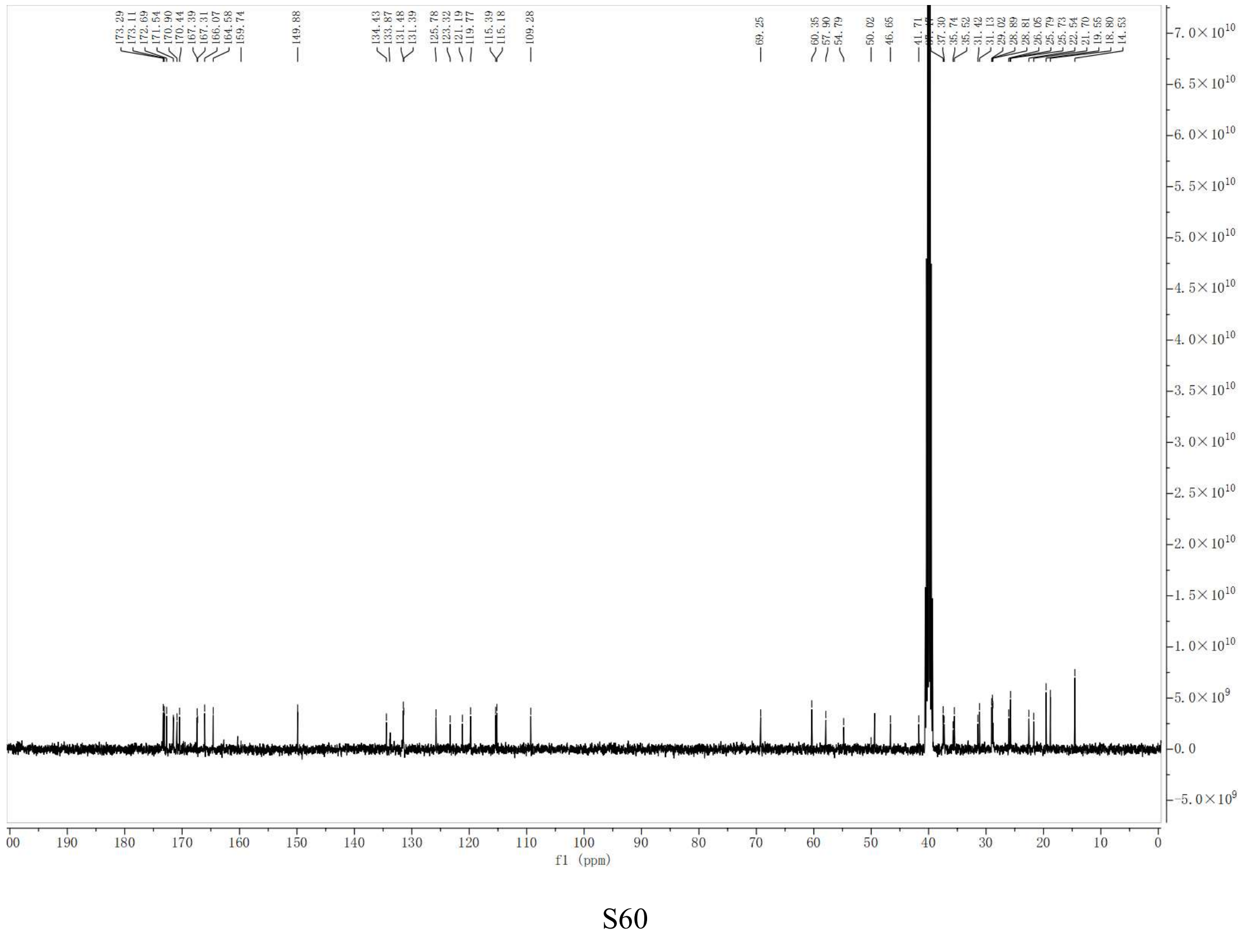

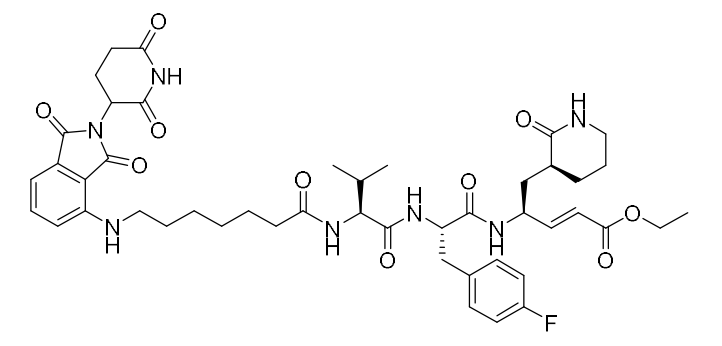

D19

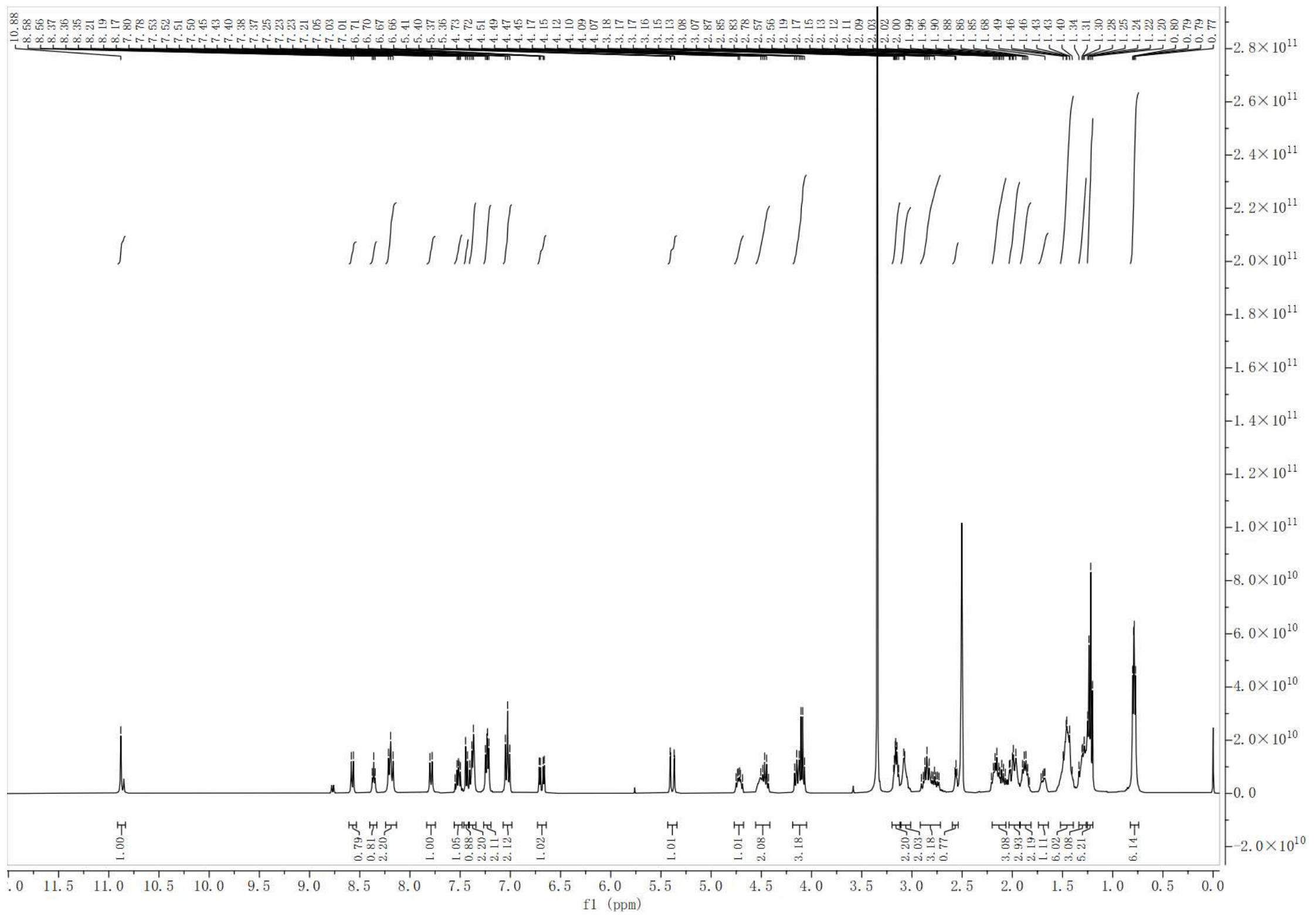

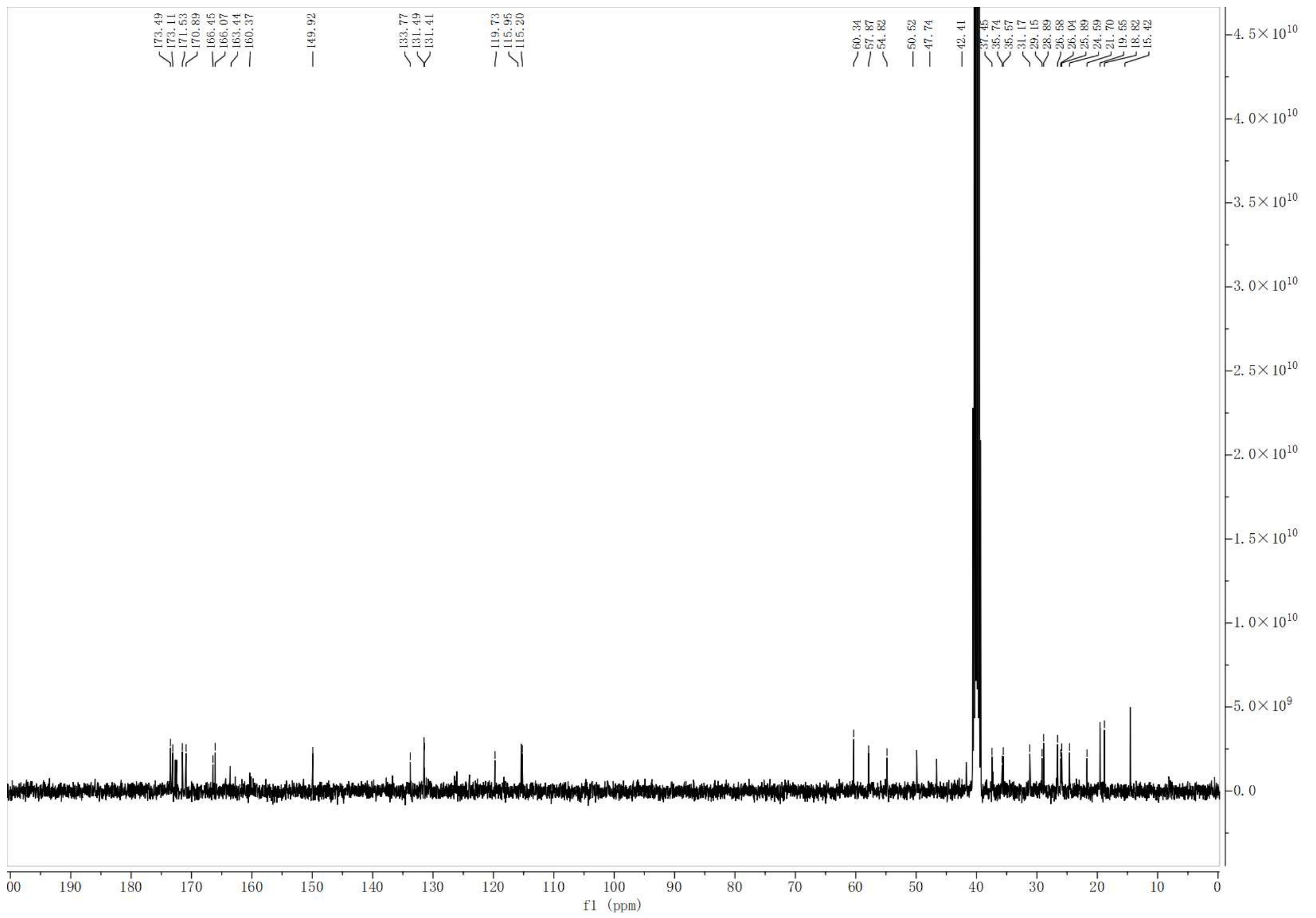

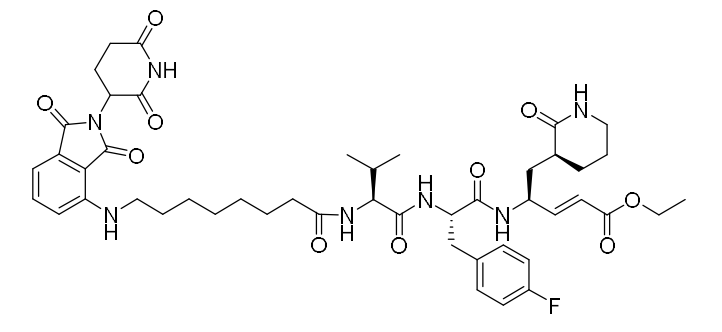

D20

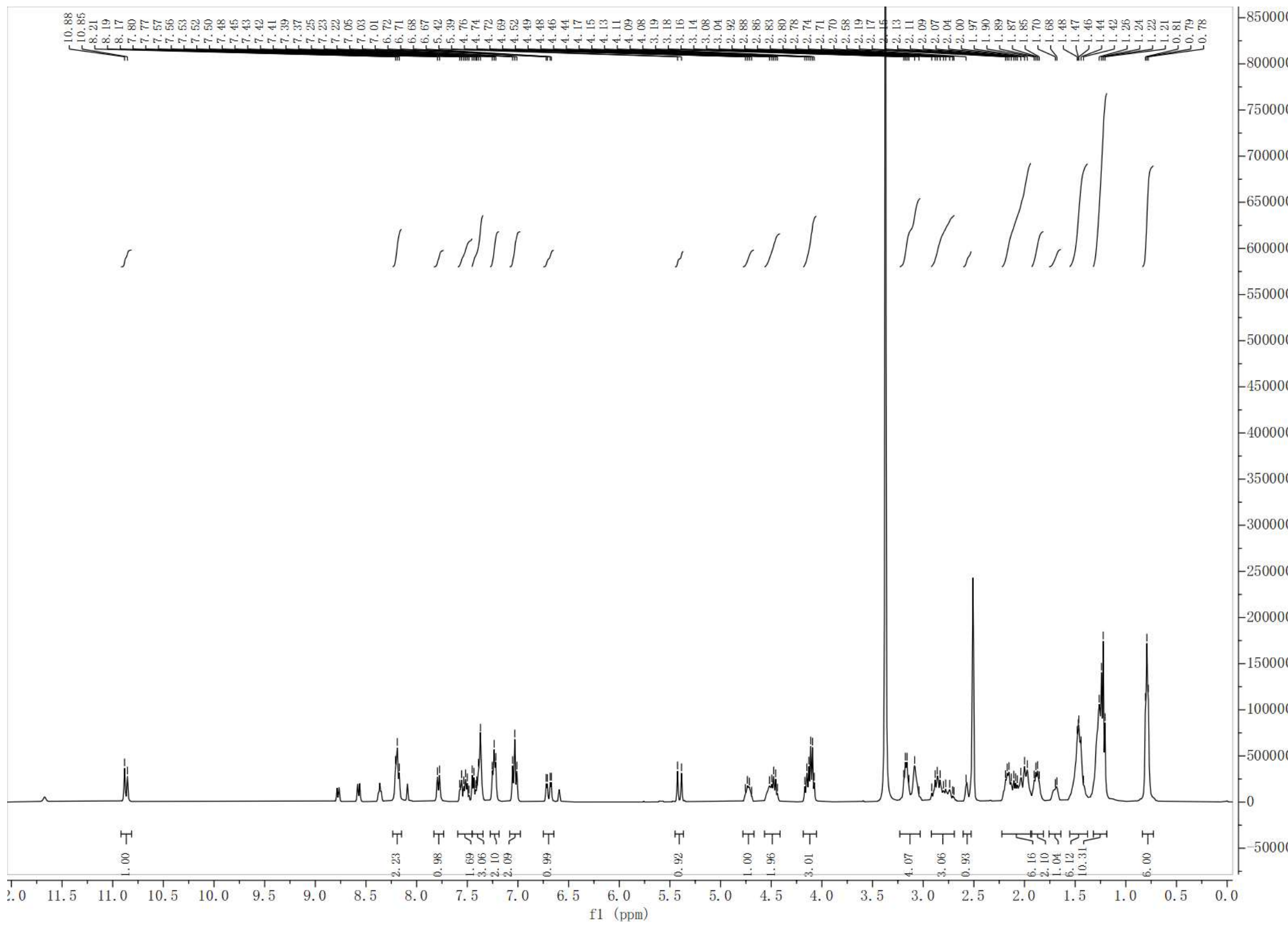

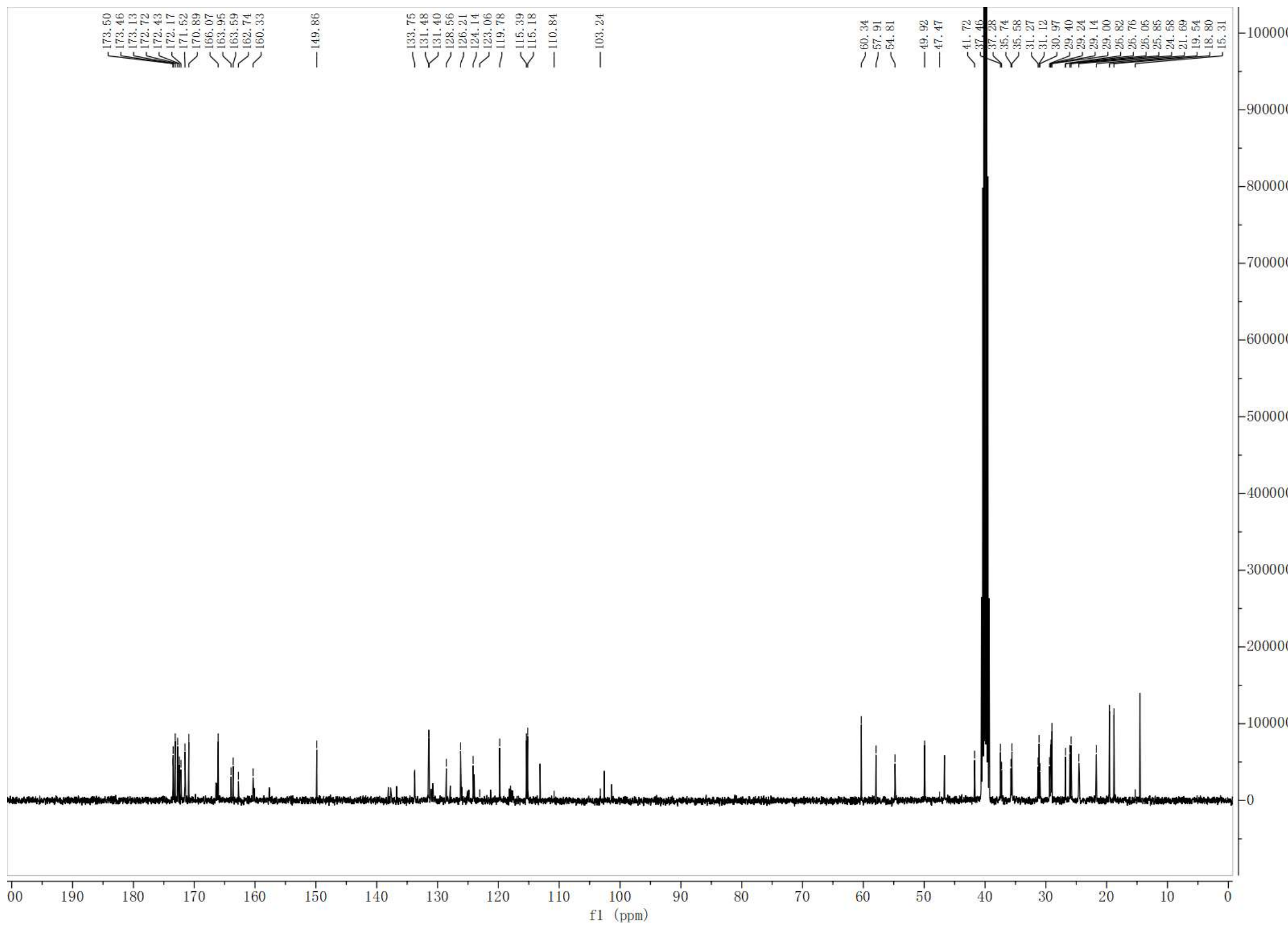

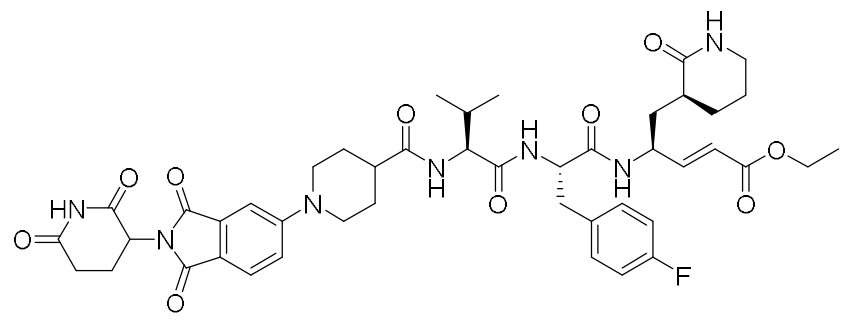

D21

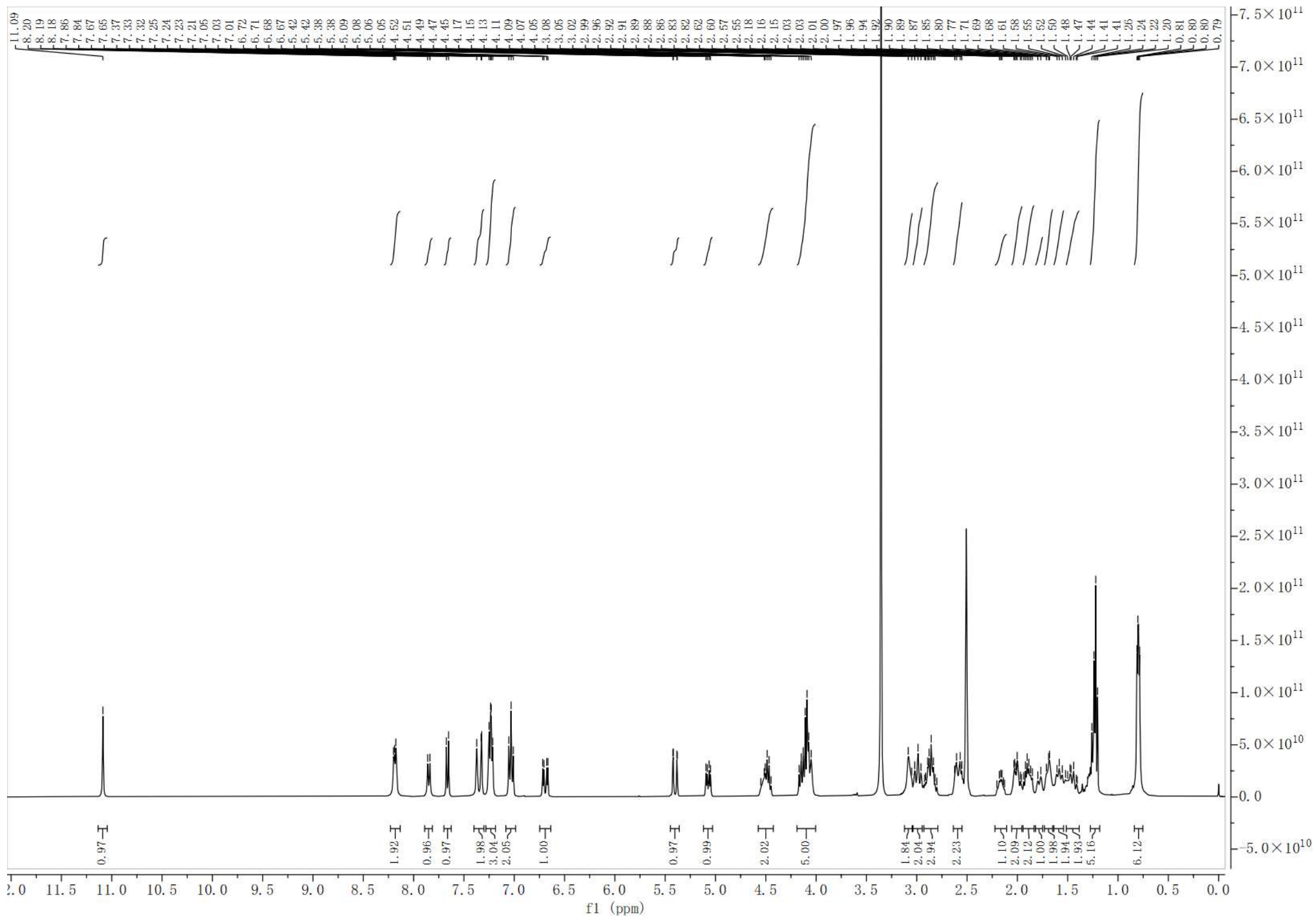

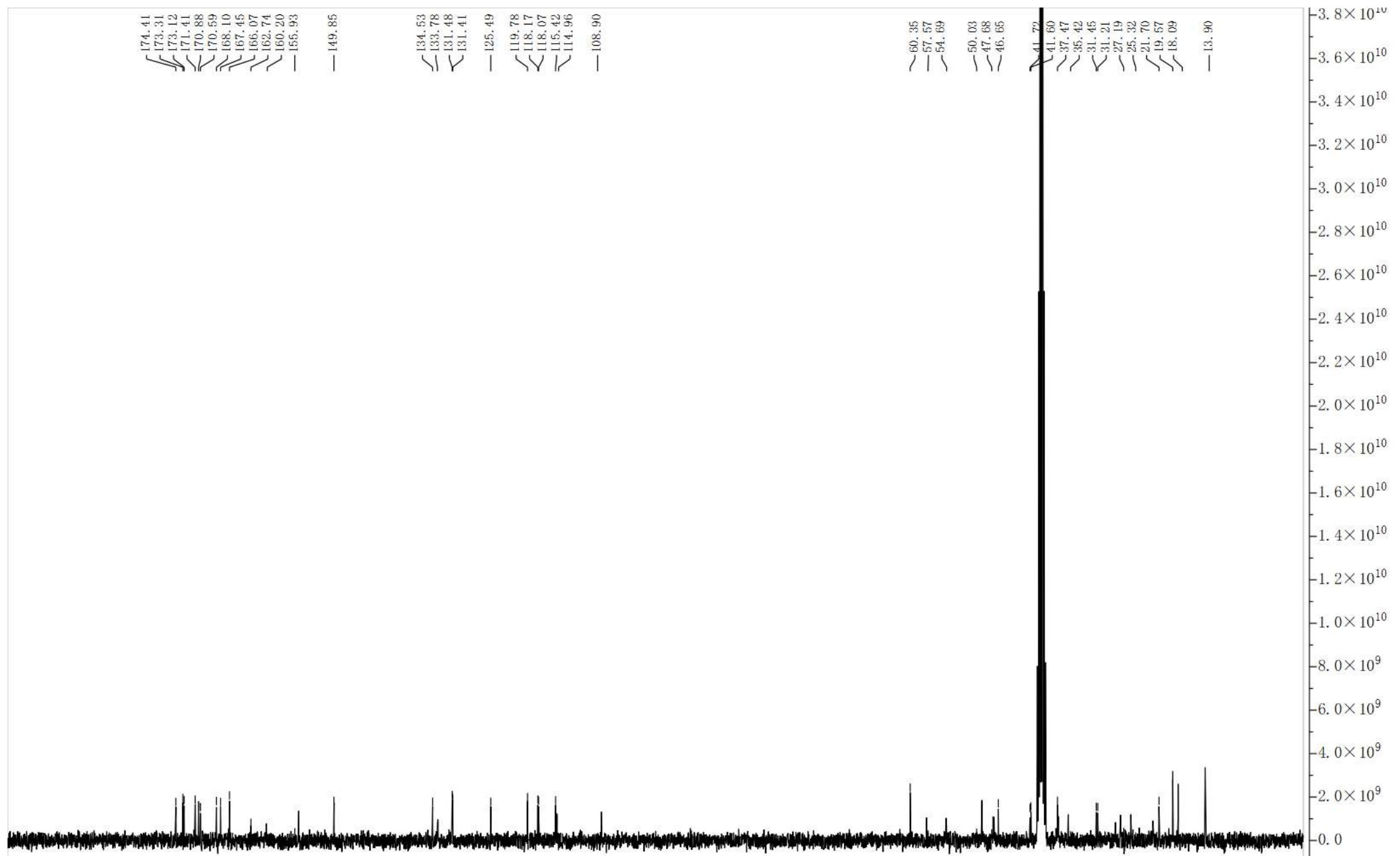

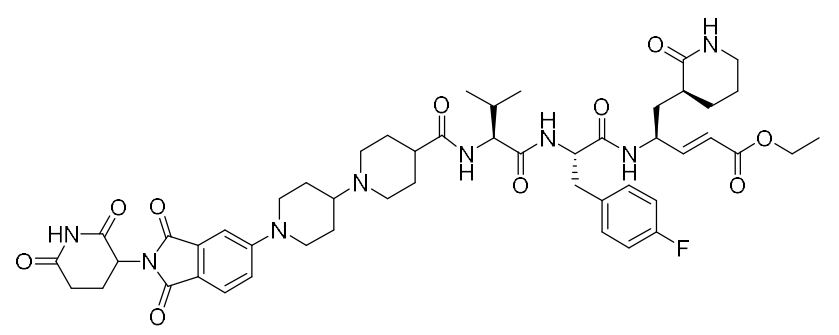

D22

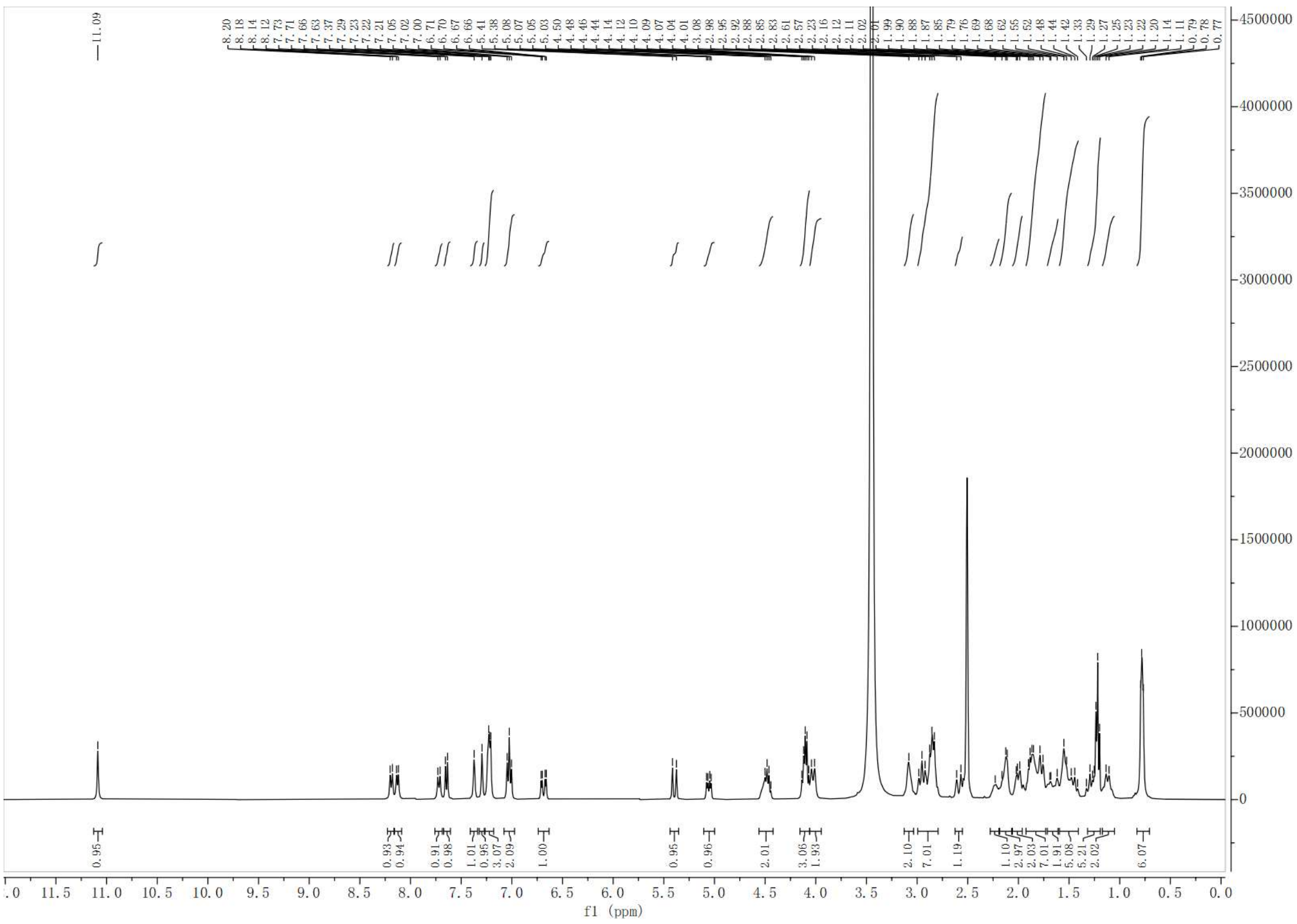

11.09
8.20 8.18 8.14 8.12 7.73 7.71 7.71 7.66 7.63 7.37 7.29 7.23 7.22 7.21 7.05 7.02 7.00 6.71 6.70 6.67 6.66 5.41 5.38 5.08 5.07 5.05 5.03 4.50 4.48 4.46 4.44 4.14 4.12 4.10 4.09 4.07 4.04 4.01 3.08 2.98 2.95 2.92 2.88 2.85 2.83 2.61 2.57 2.23 2.16 2.12 2.11 2.02 2.01 1.99 1.90 1.88 1.87 1.85 1.79 1.76 1.69 1.68 1.62 1.55 1.52 1.48 1.44 1.42 1.33 1.29 1.27 1.25 1.23 1.22 1.20 1.14 1.11 0.79 0.78 0.77
0.95 0.93 0.94 0.91 0.98 1.01 0.95 3.07 2.09 1.00 0.95 0.96 2.01 3.06 1.93 2.10 7.01 1.19 1.10 2.97 2.03 7.01 1.91 5.08 5.21 2.02 6.07
f1 (ppm)

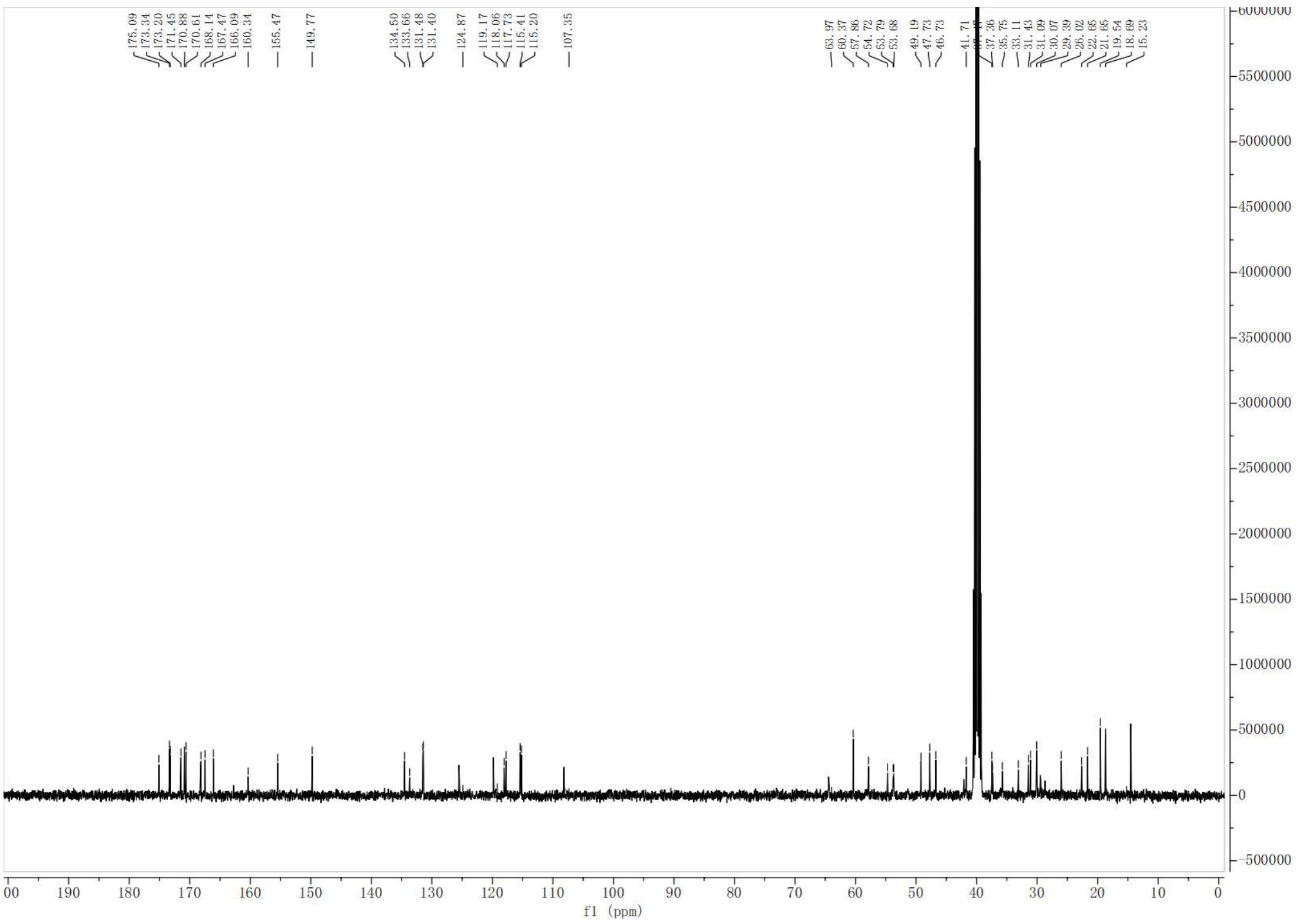

175.09 173.34 173.20 171.45 170.88 170.61 168.14 167.47 166.09 160.34 155.47 149.77 134.50 133.66 131.48 131.40 124.87 119.17 118.06 117.73 115.41 115.20 107.35
63.97 60.37 57.86 54.72 53.79 53.68 49.19 47.73 46.73 41.71 37.36 35.75 33.11 31.43 31.09 30.07 29.39 26.02 22.65 21.65 19.54 18.69 15.23
f1 (ppm)

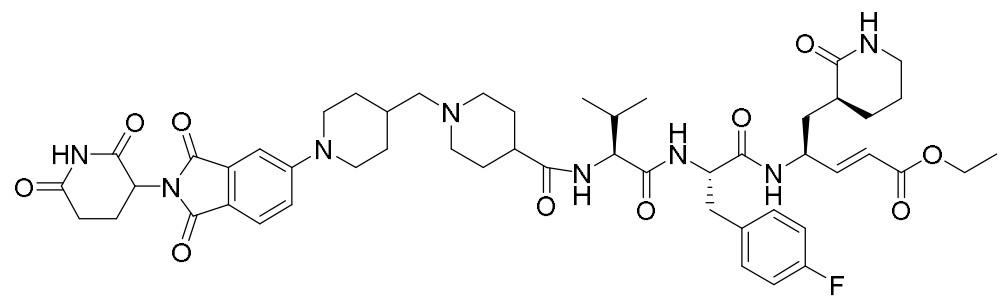

D23

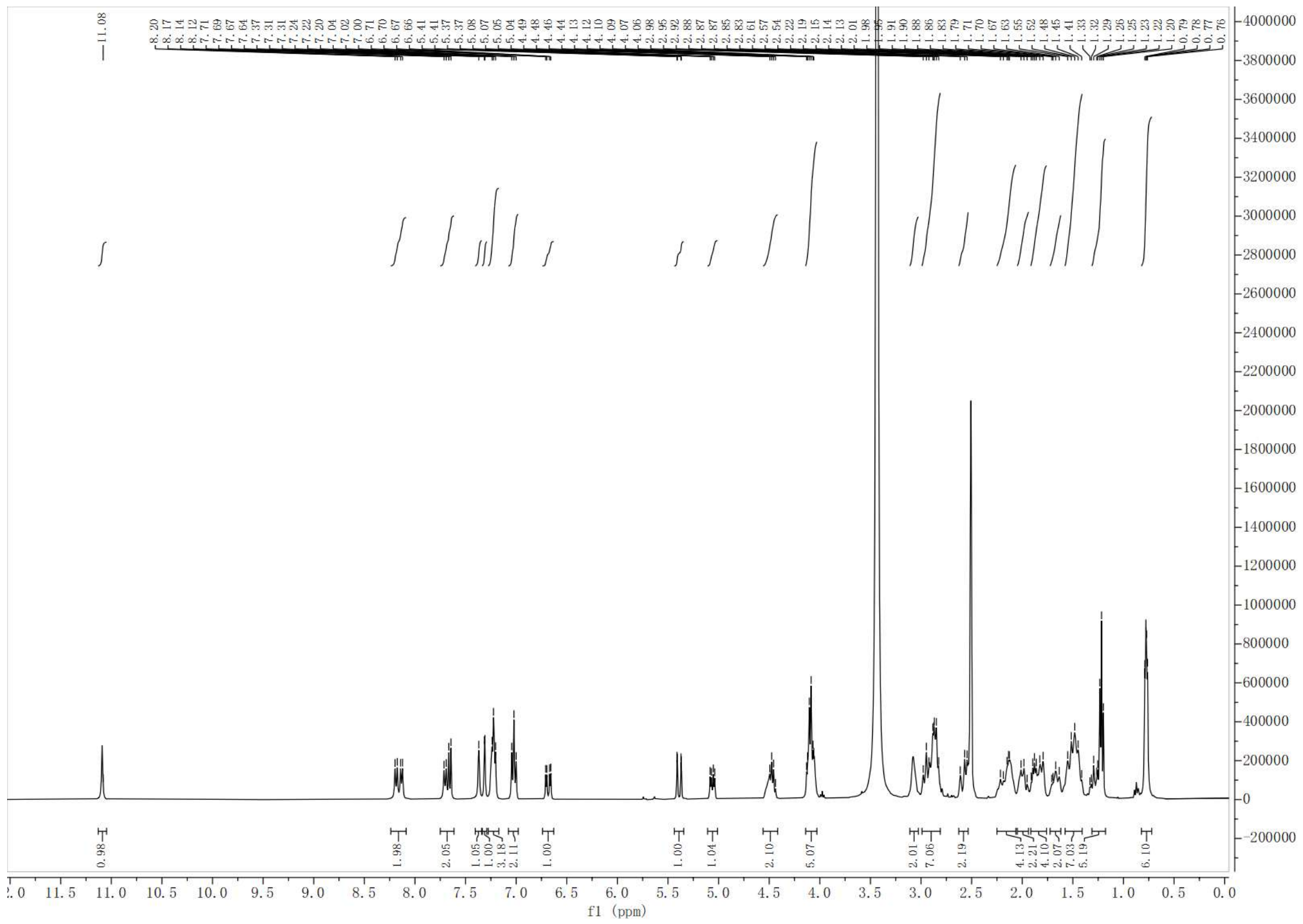

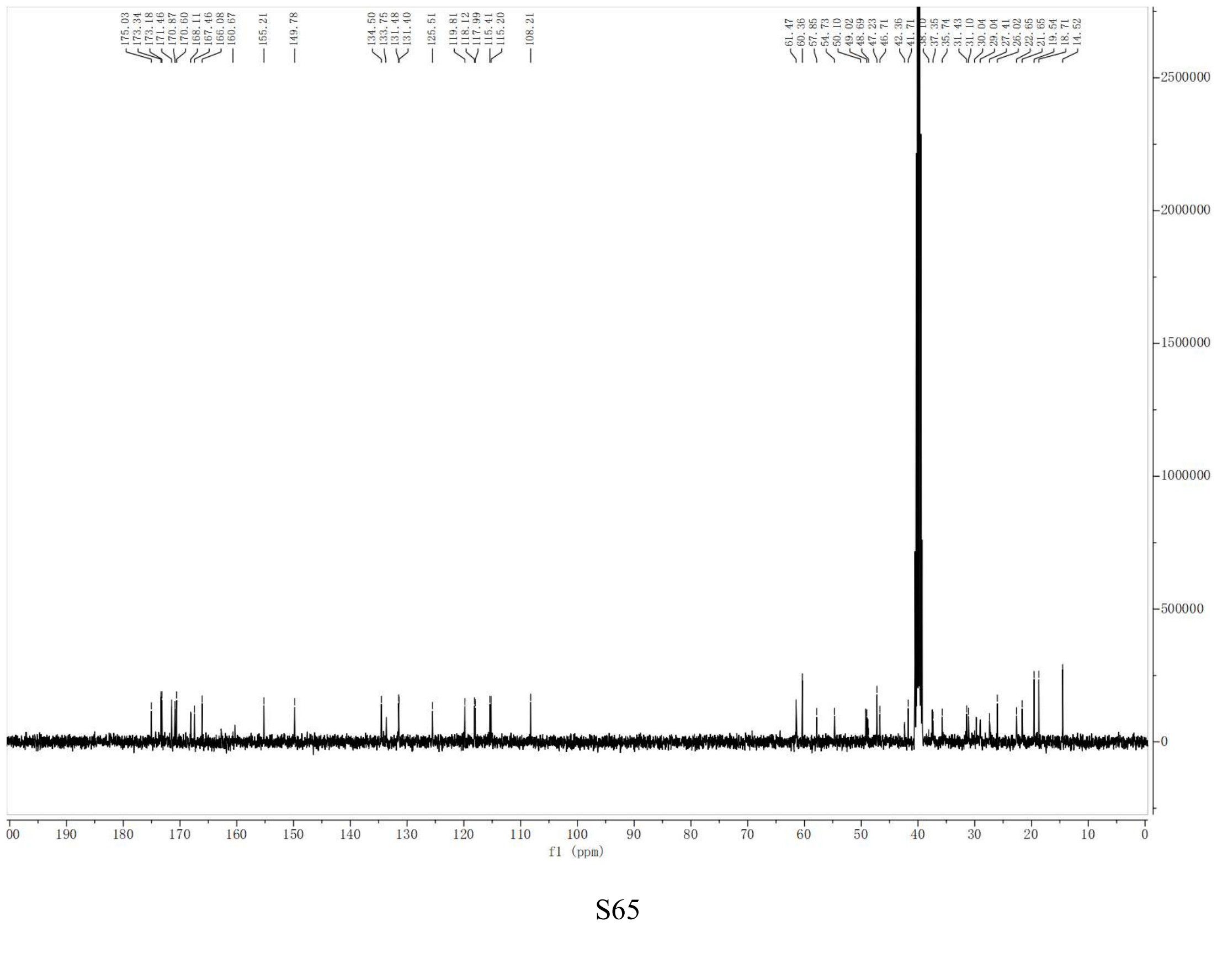

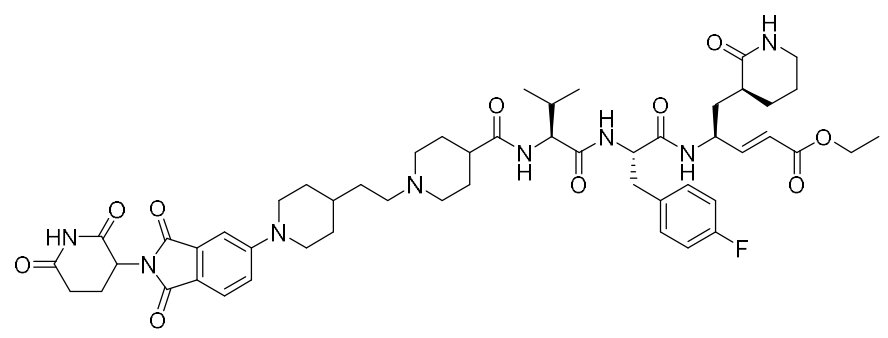

D24

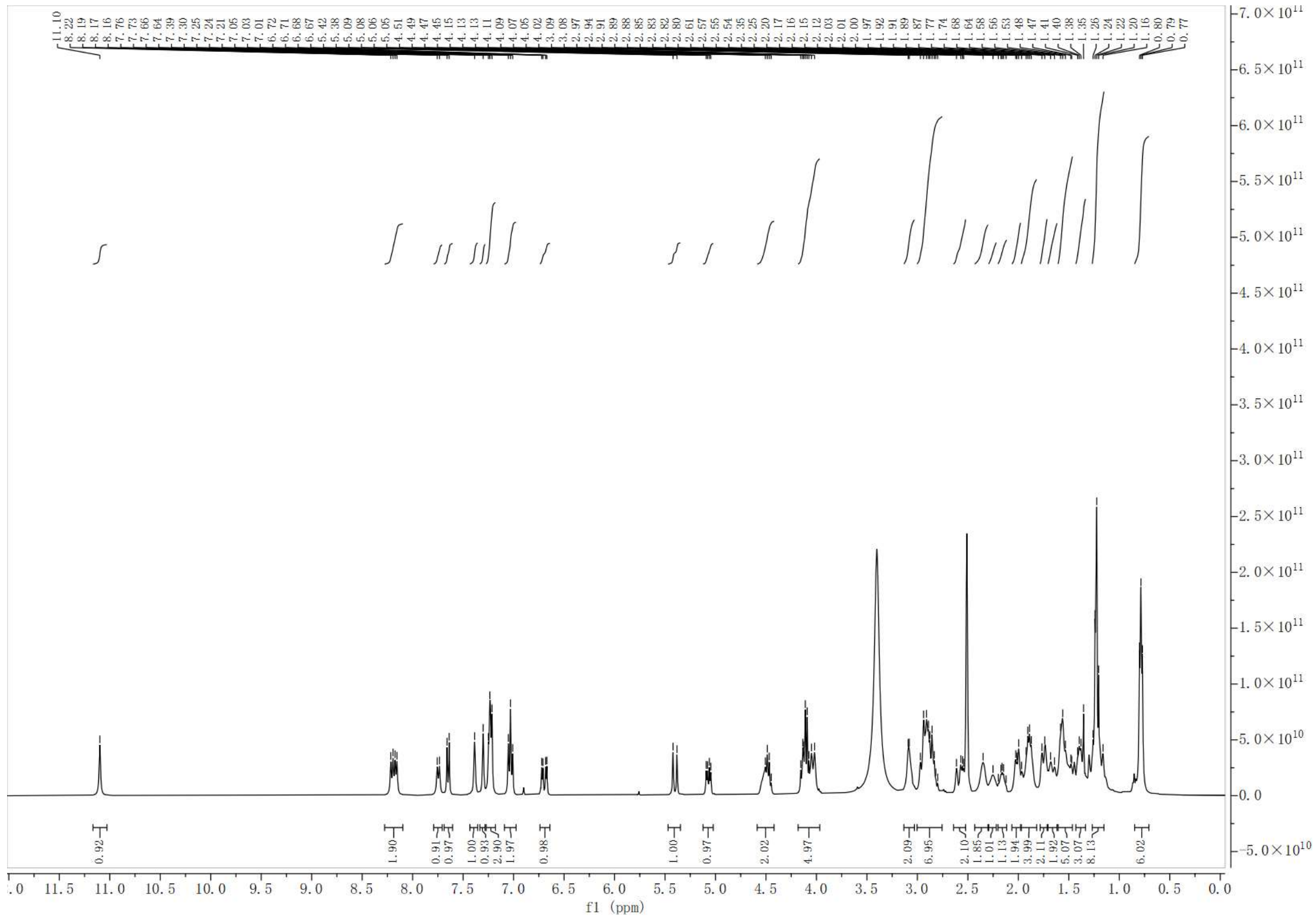

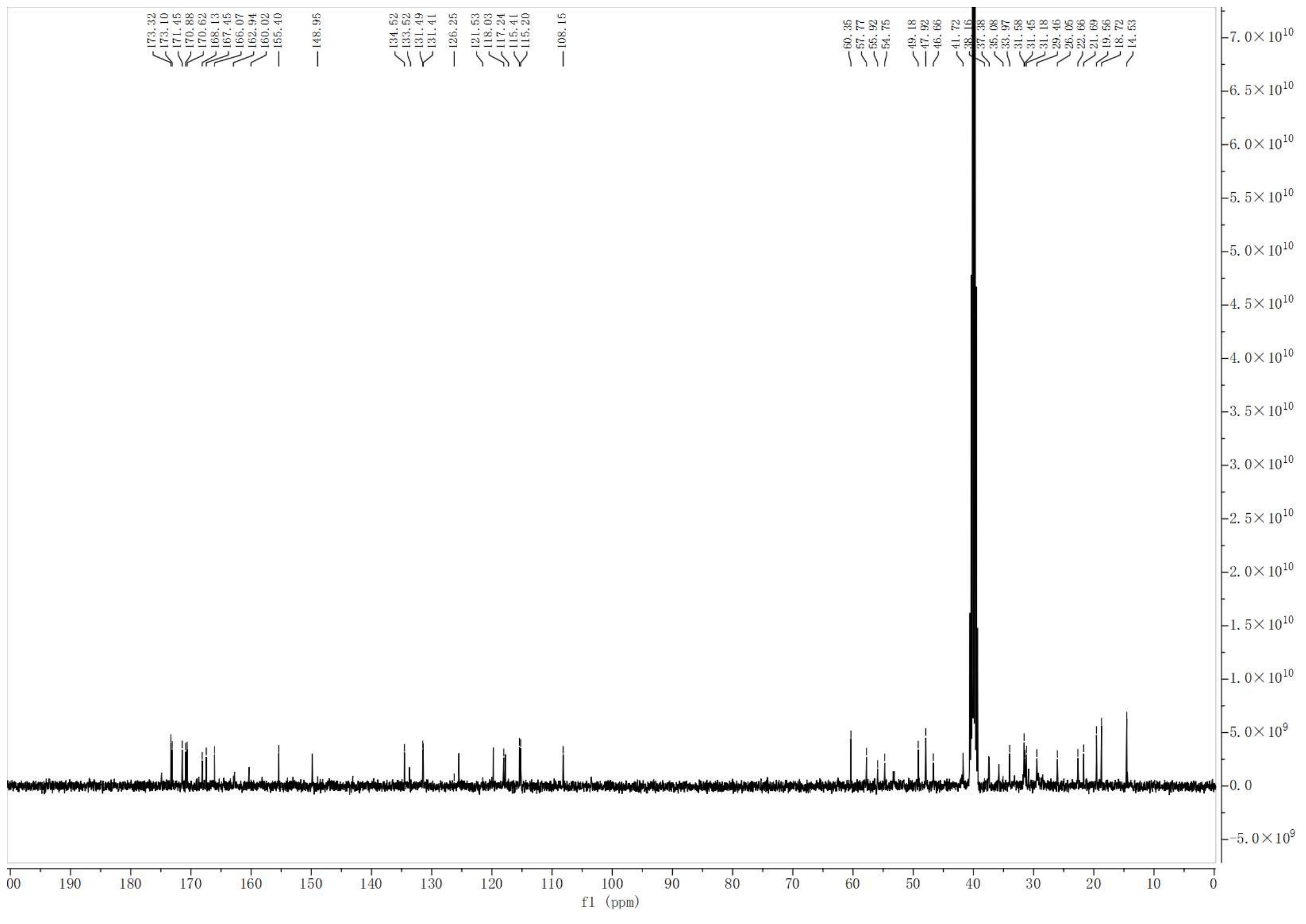

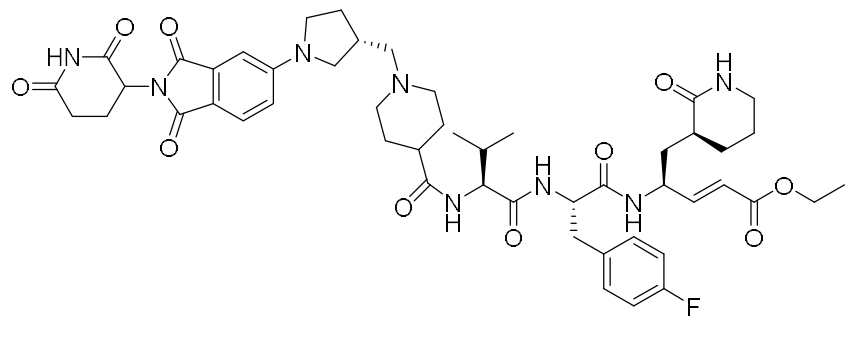

D25

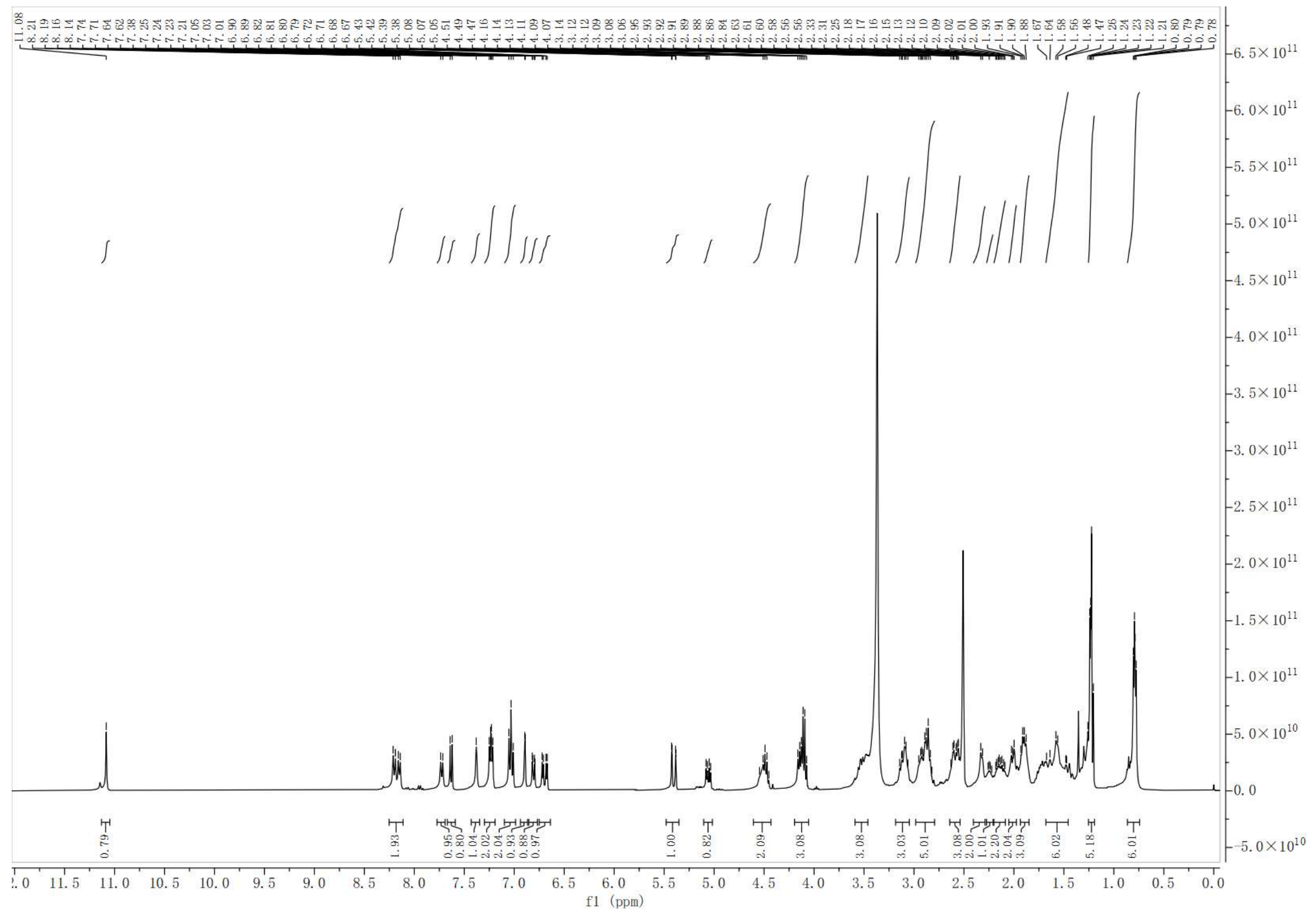

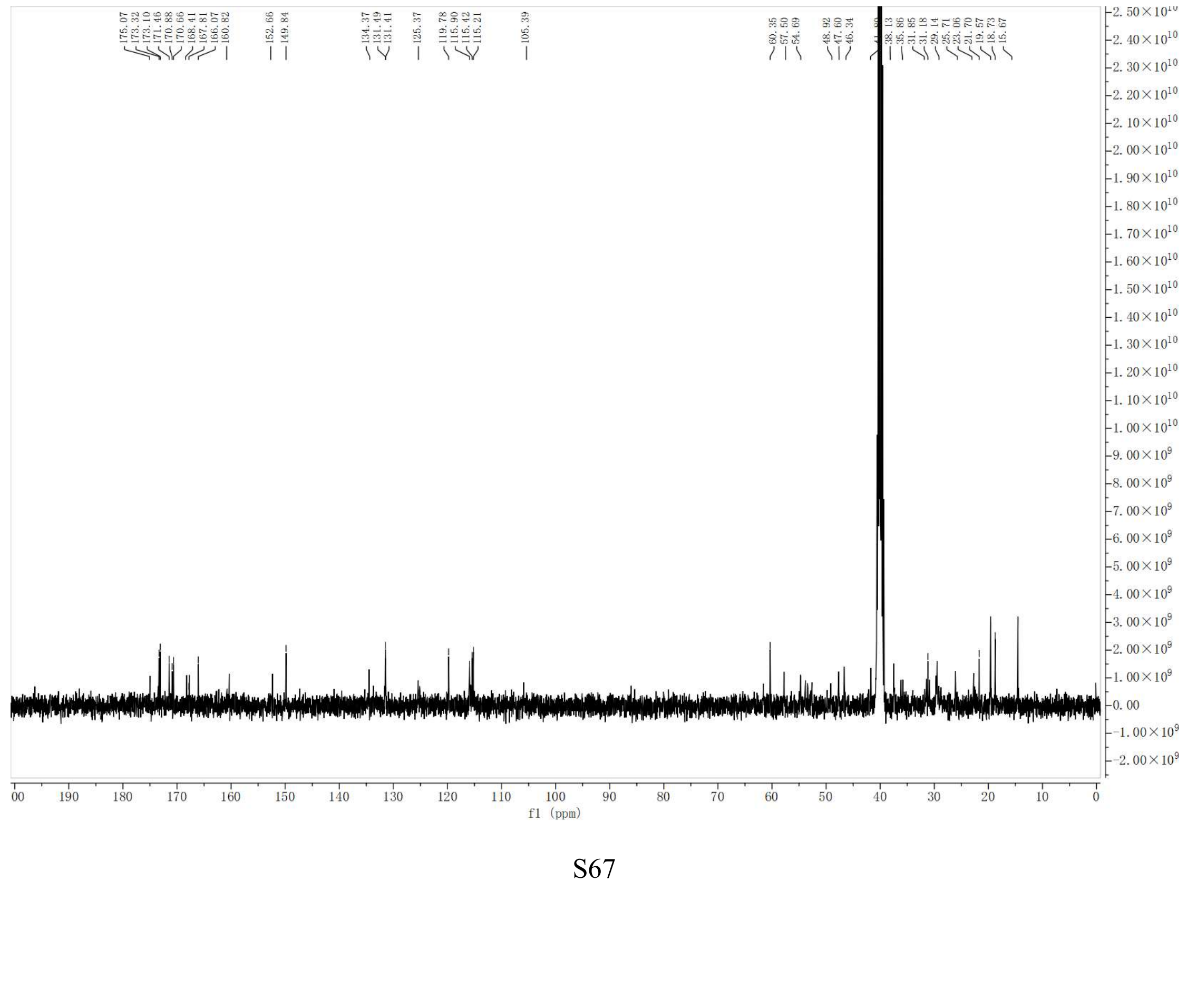

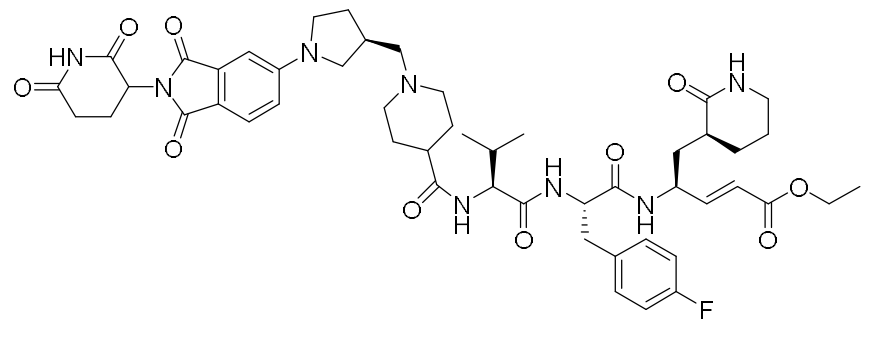

D26

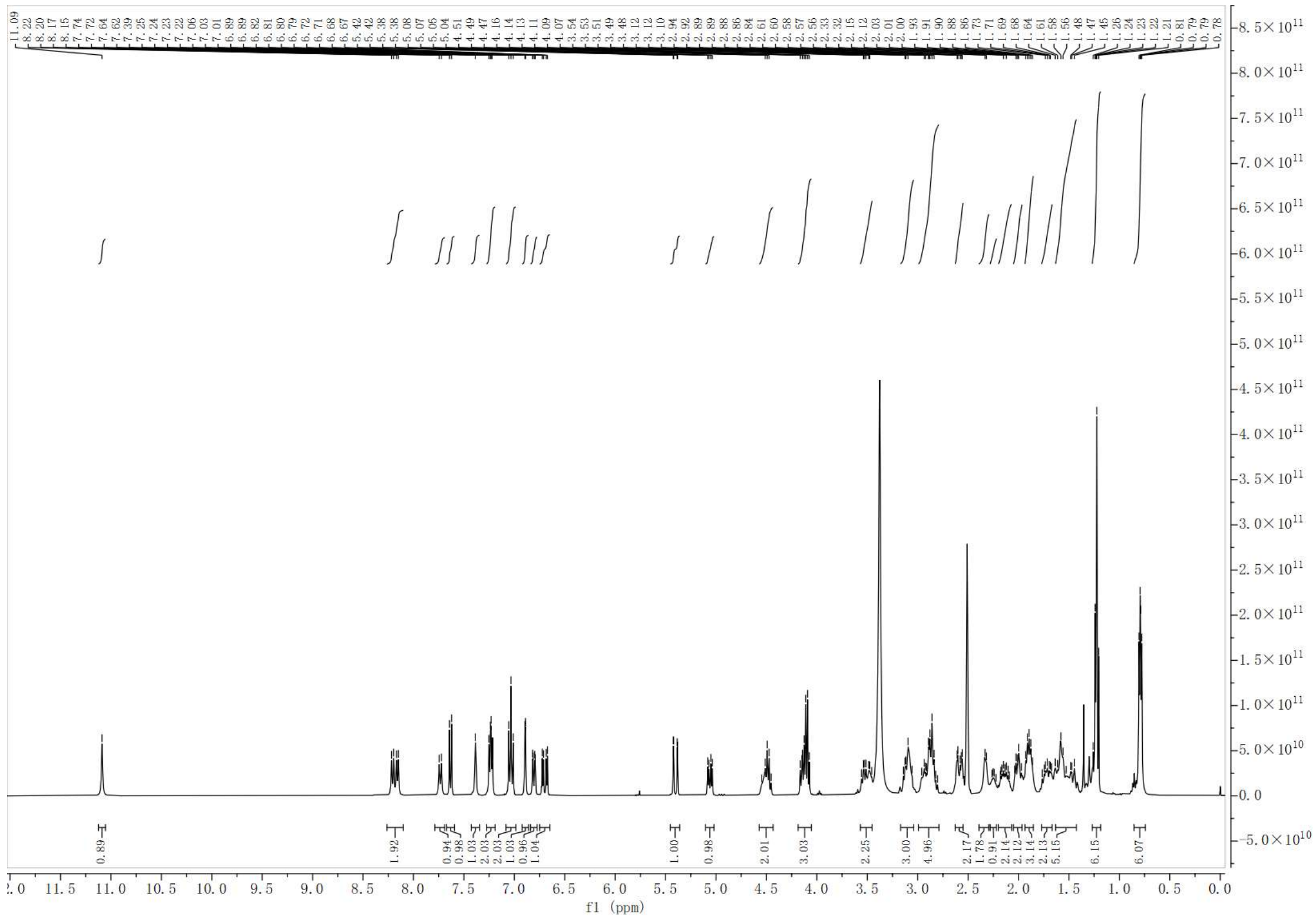

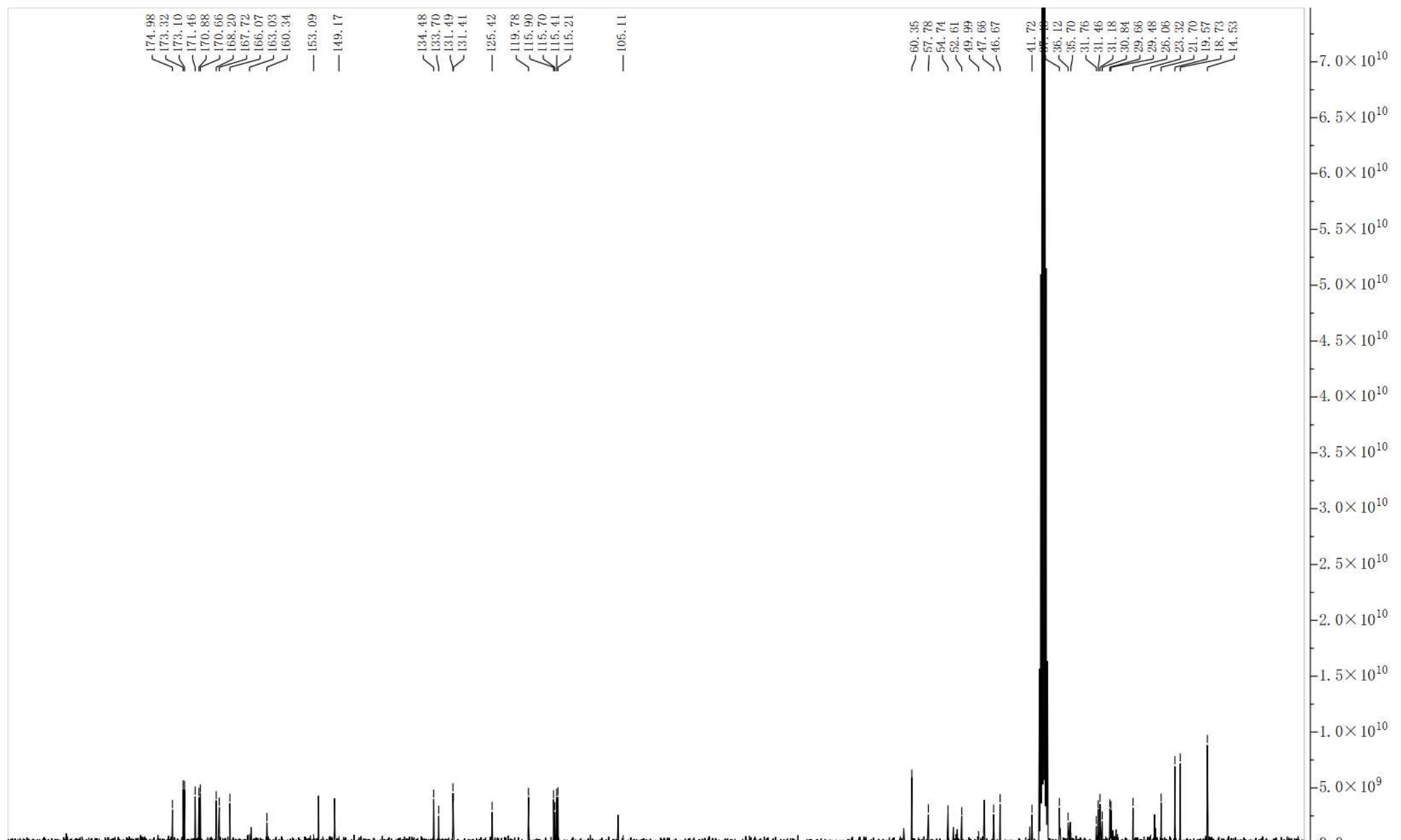

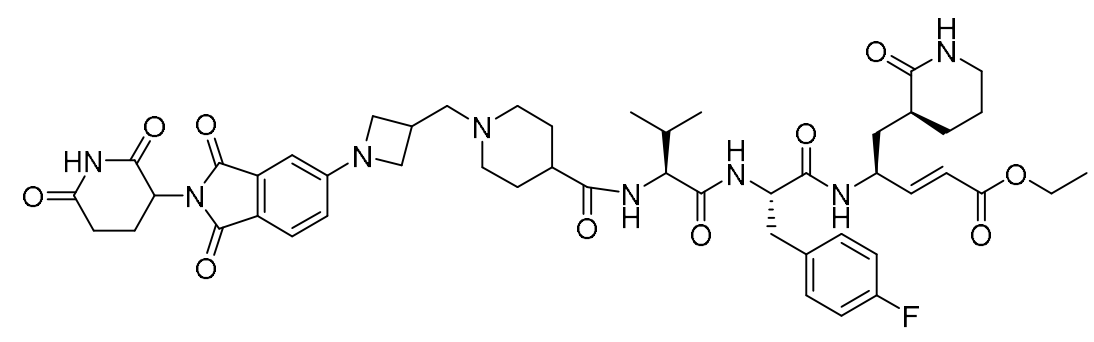

D27

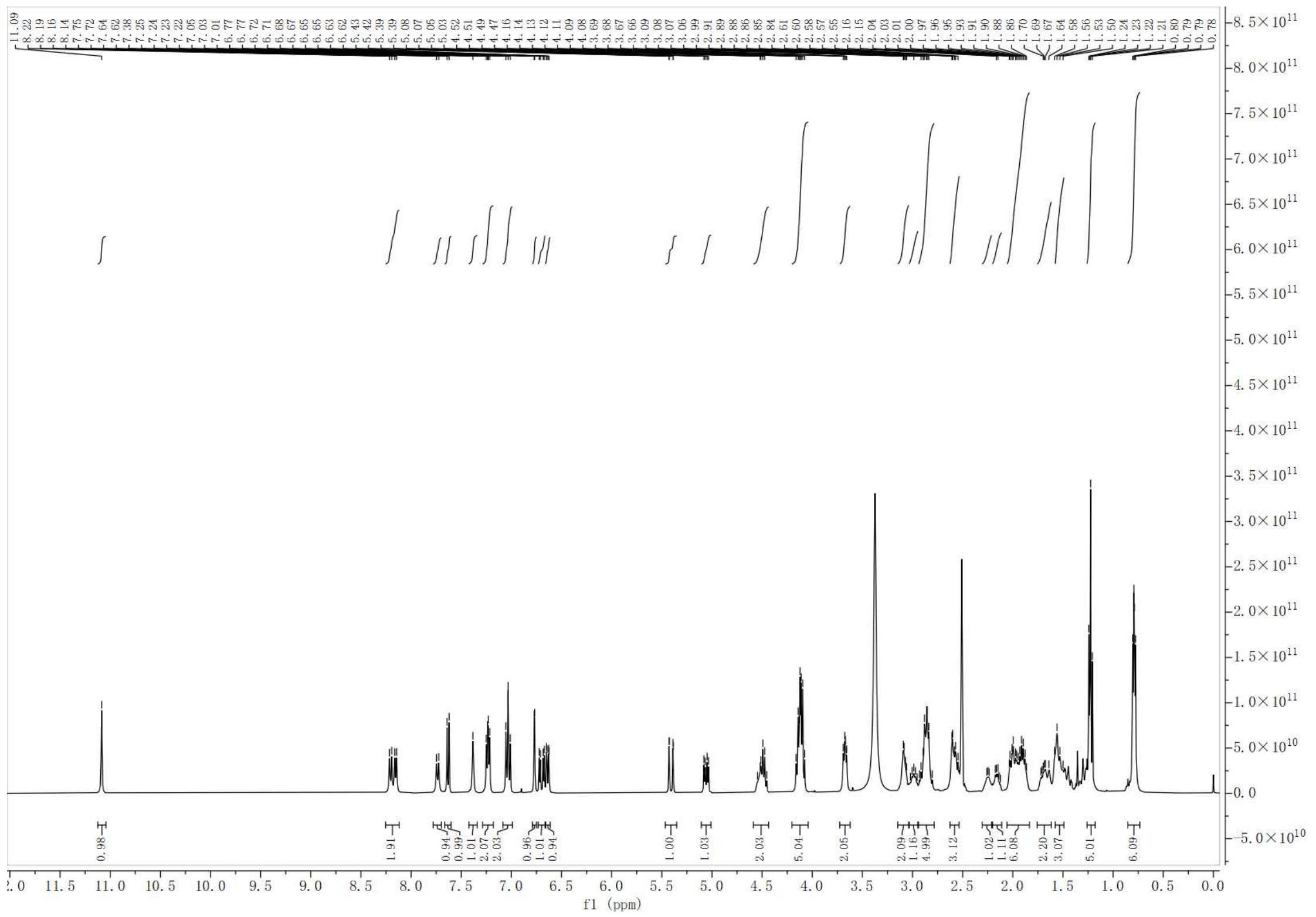

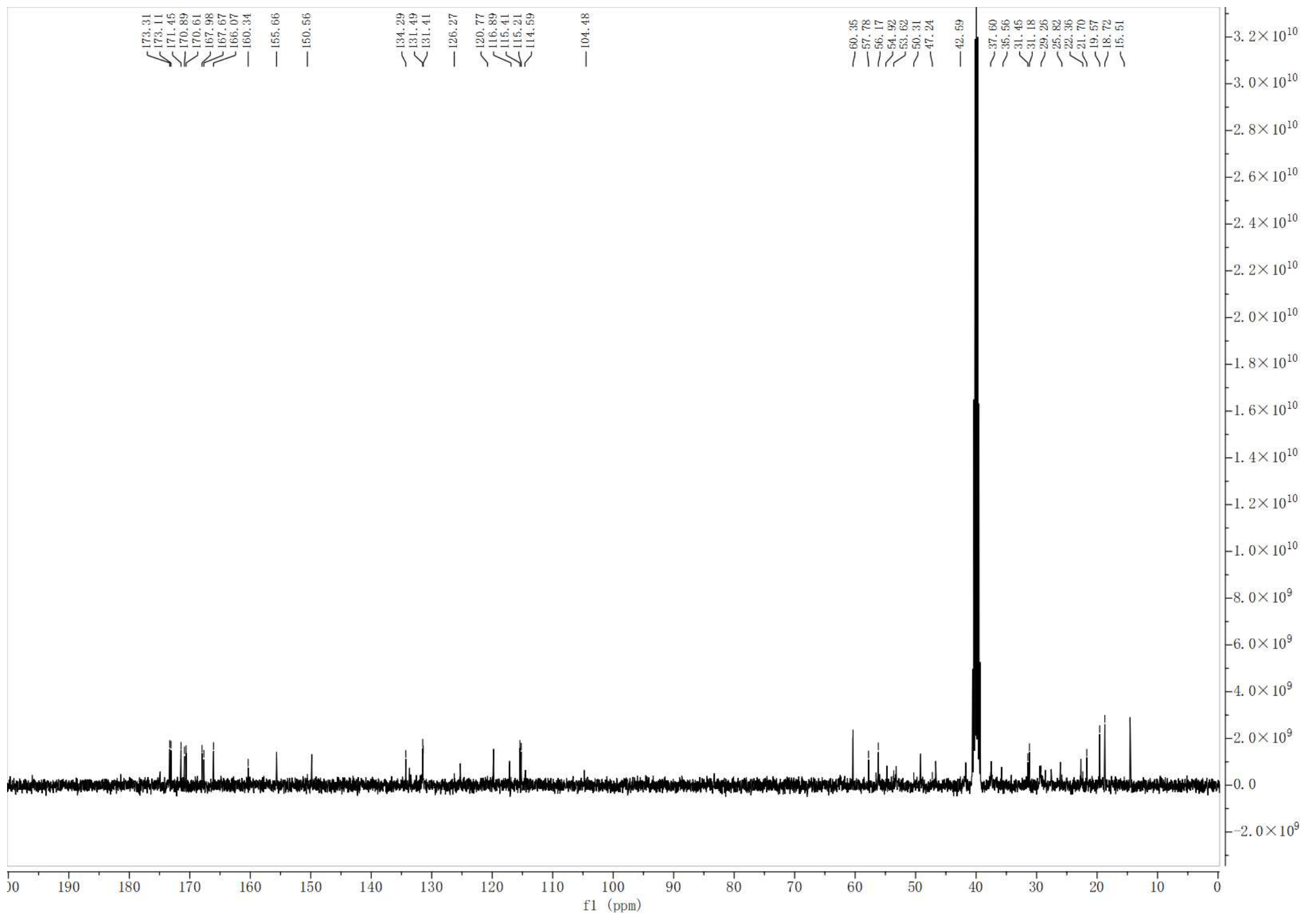

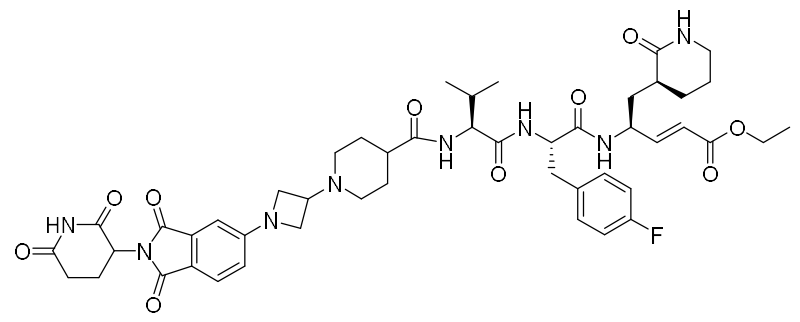

D28

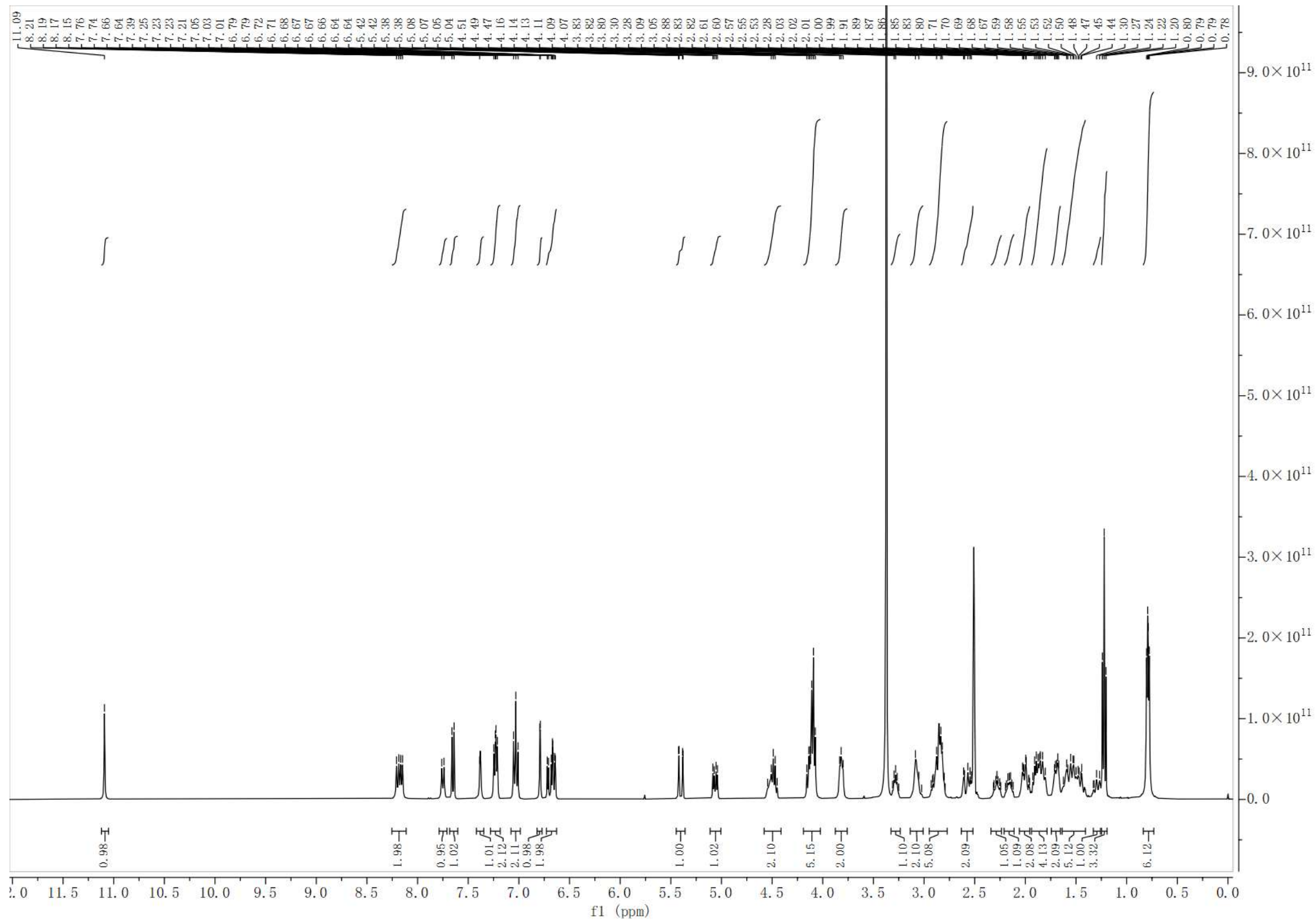

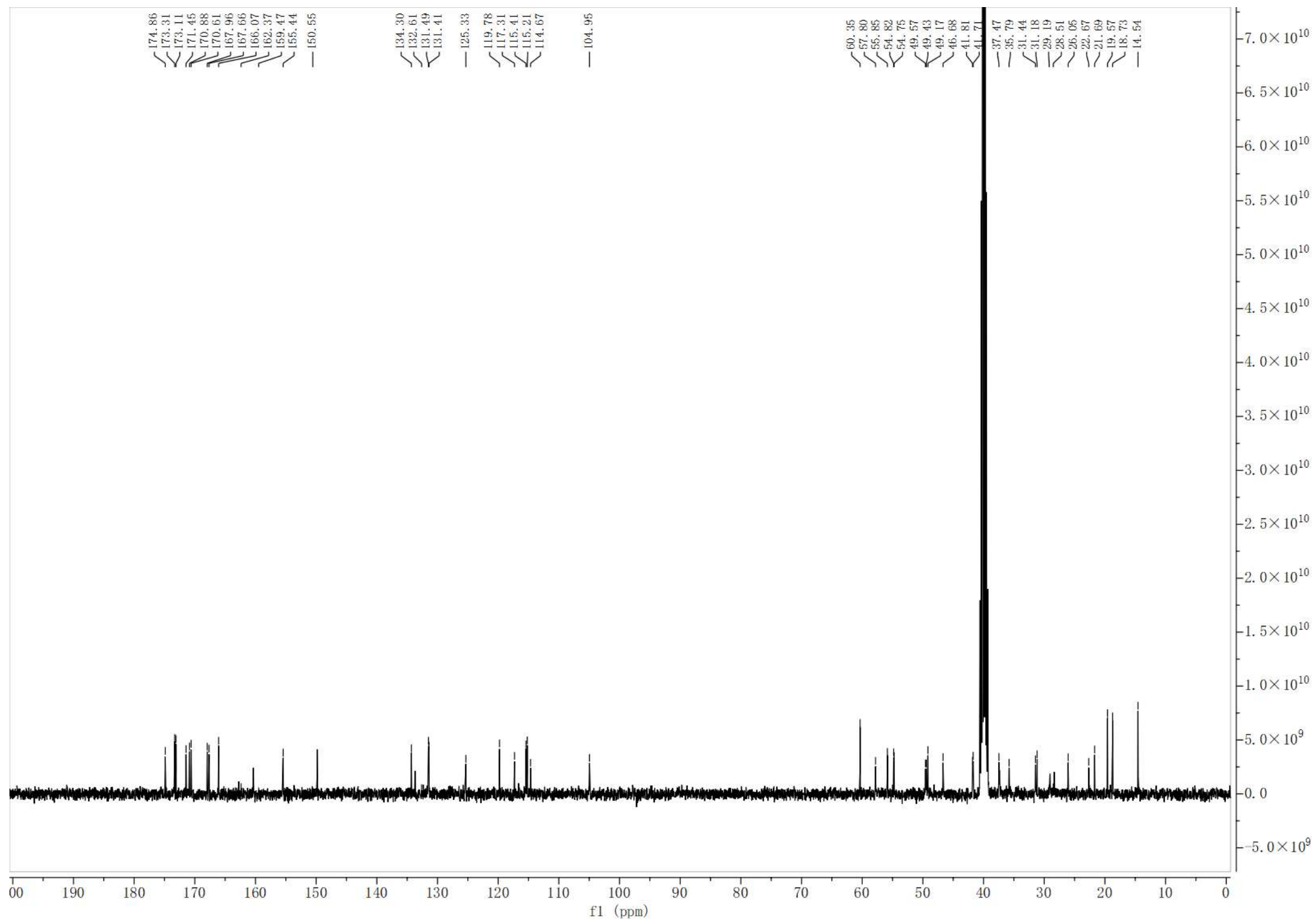

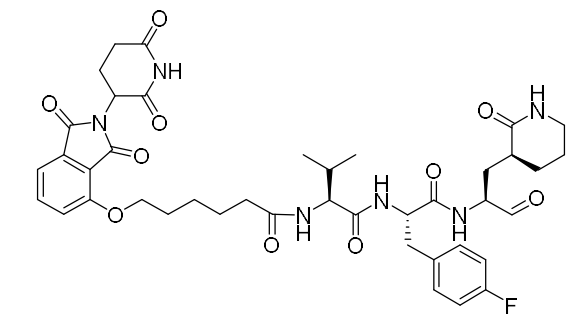

D29

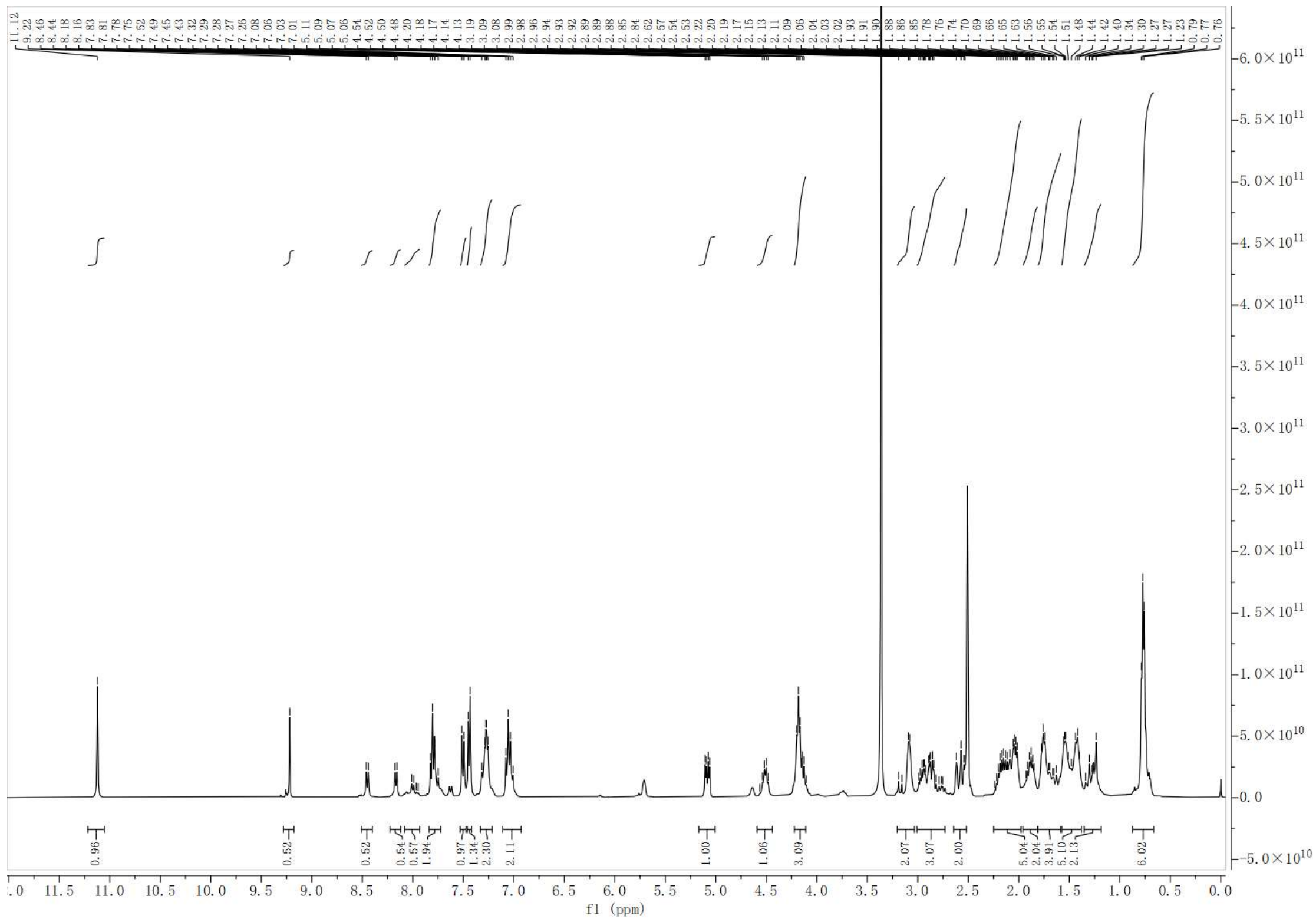

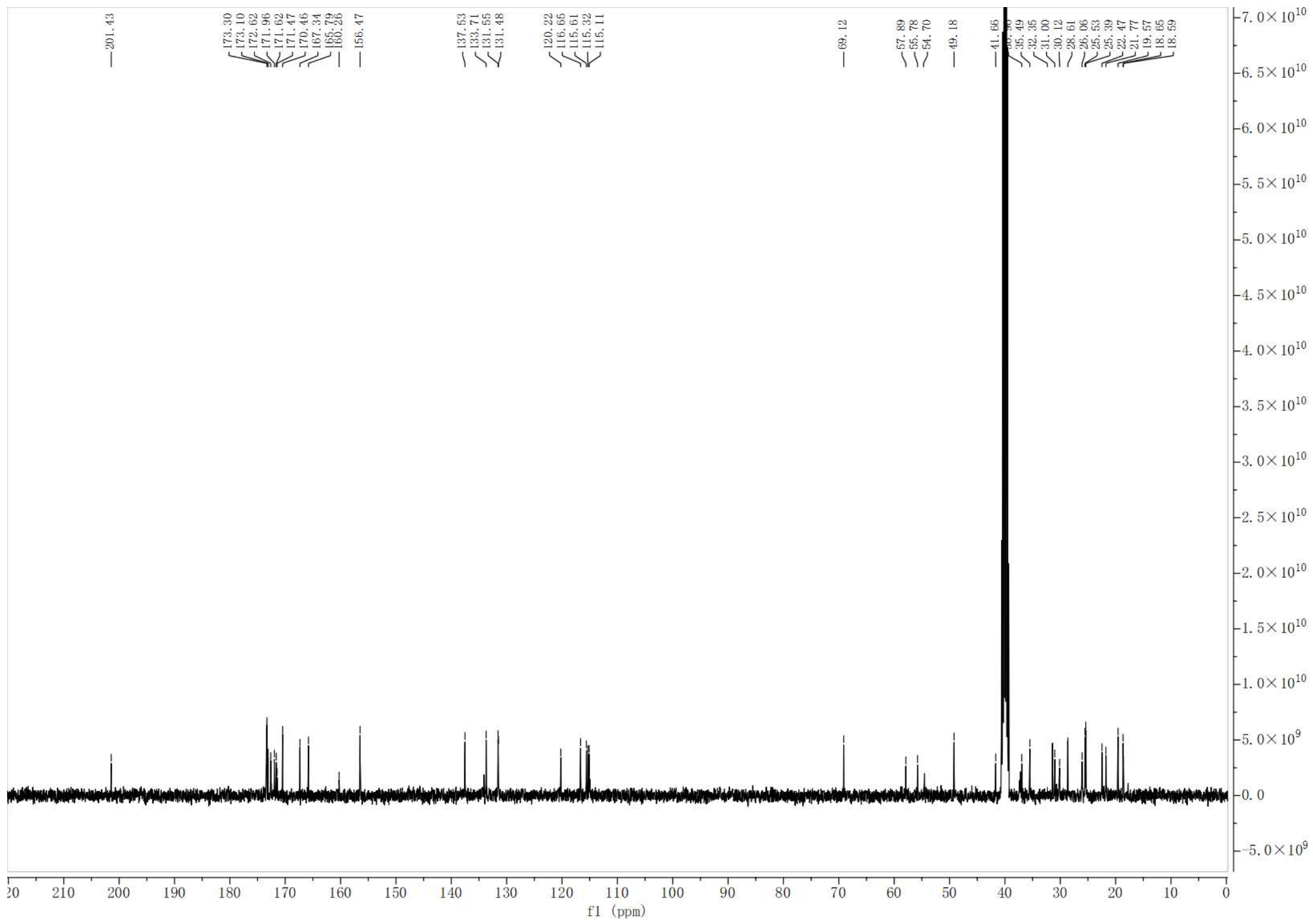

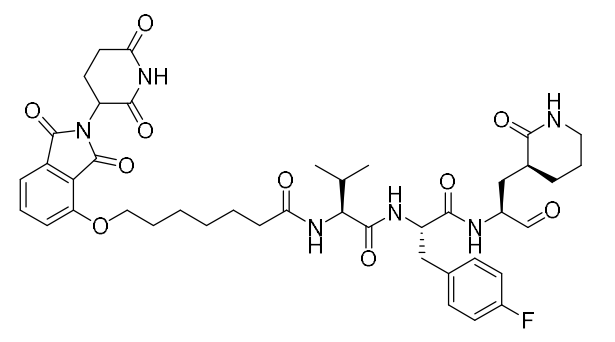

D30

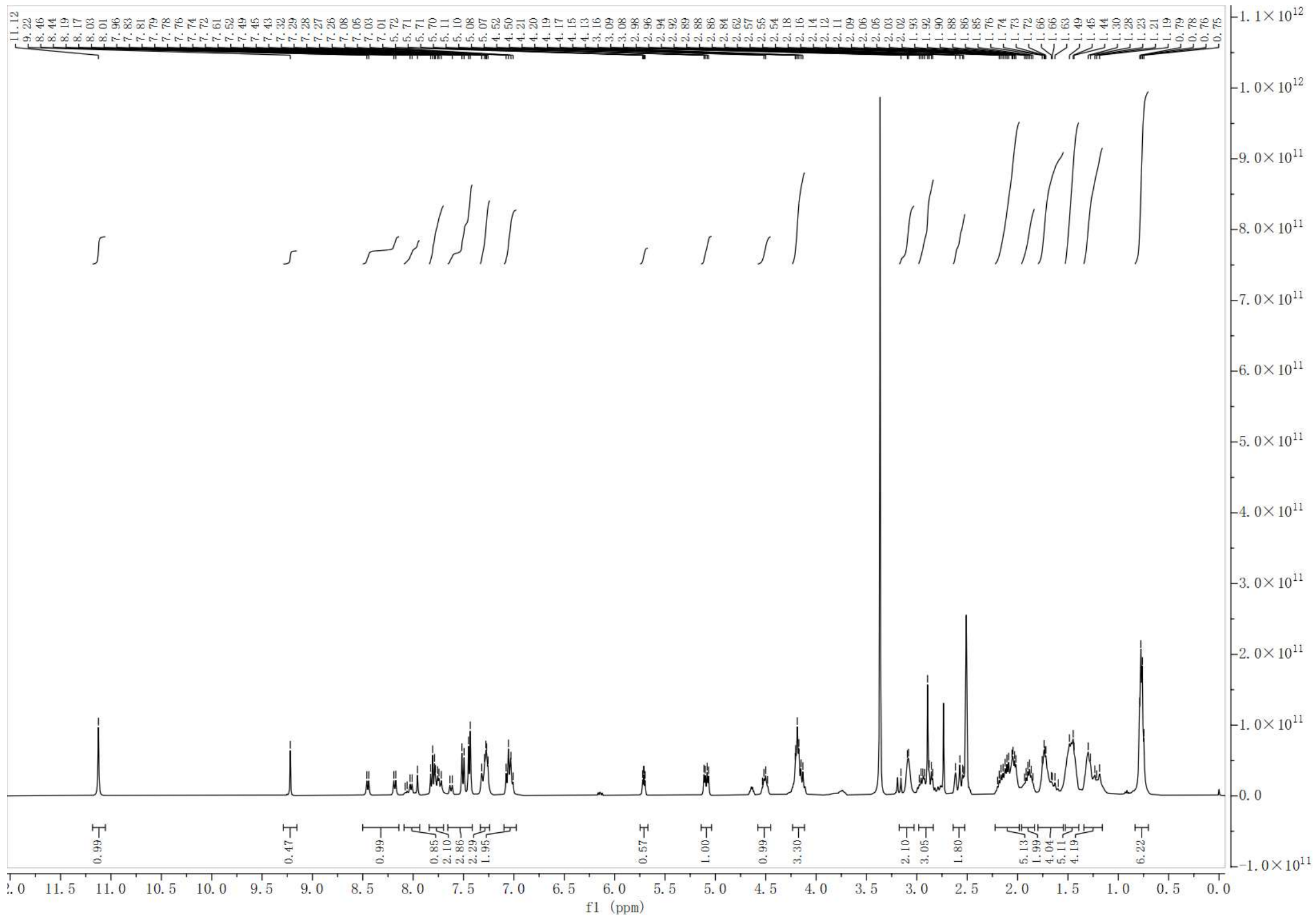

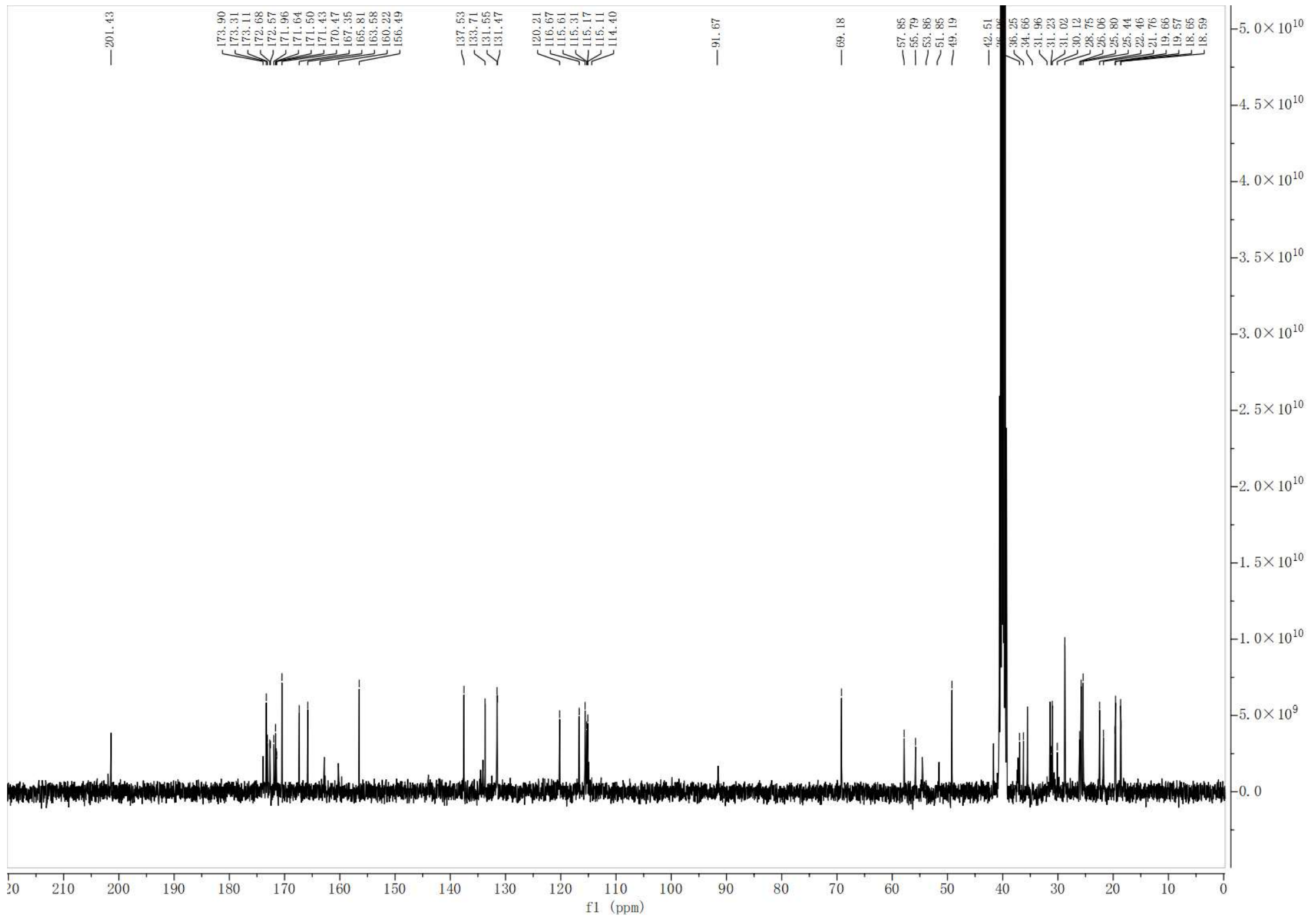

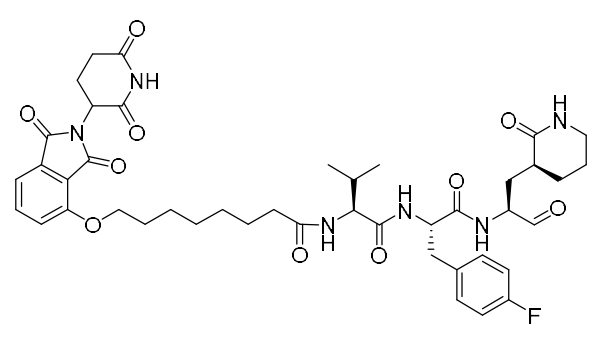

D31

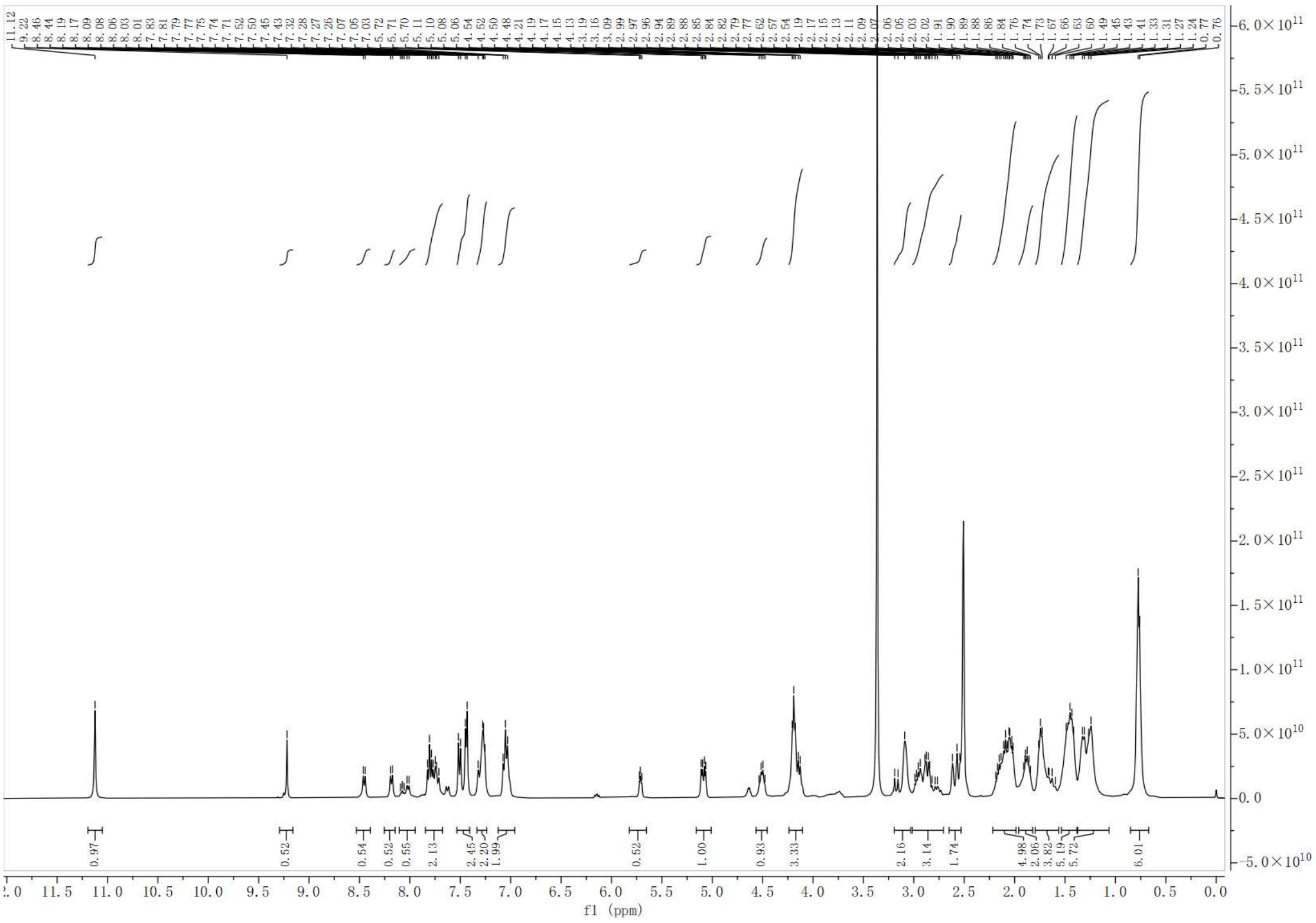

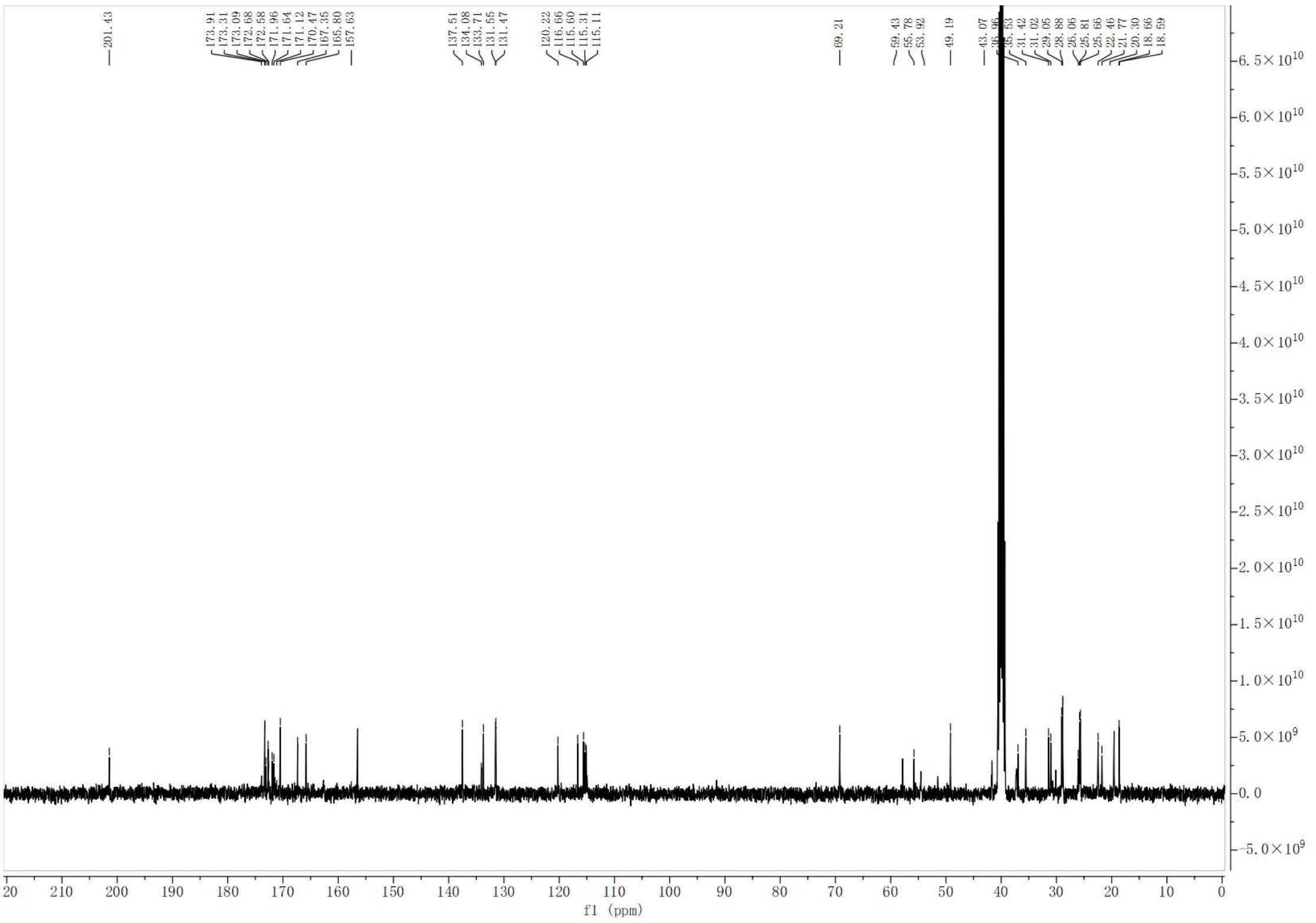

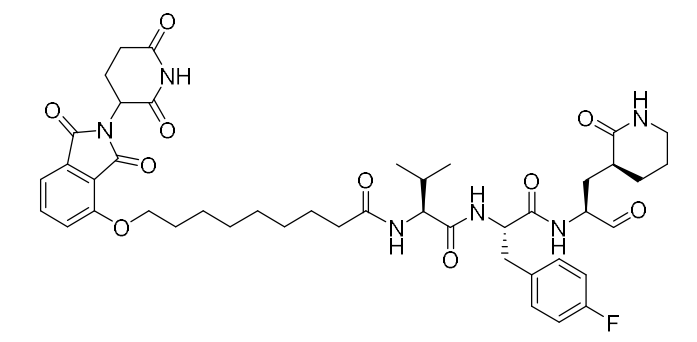

D32

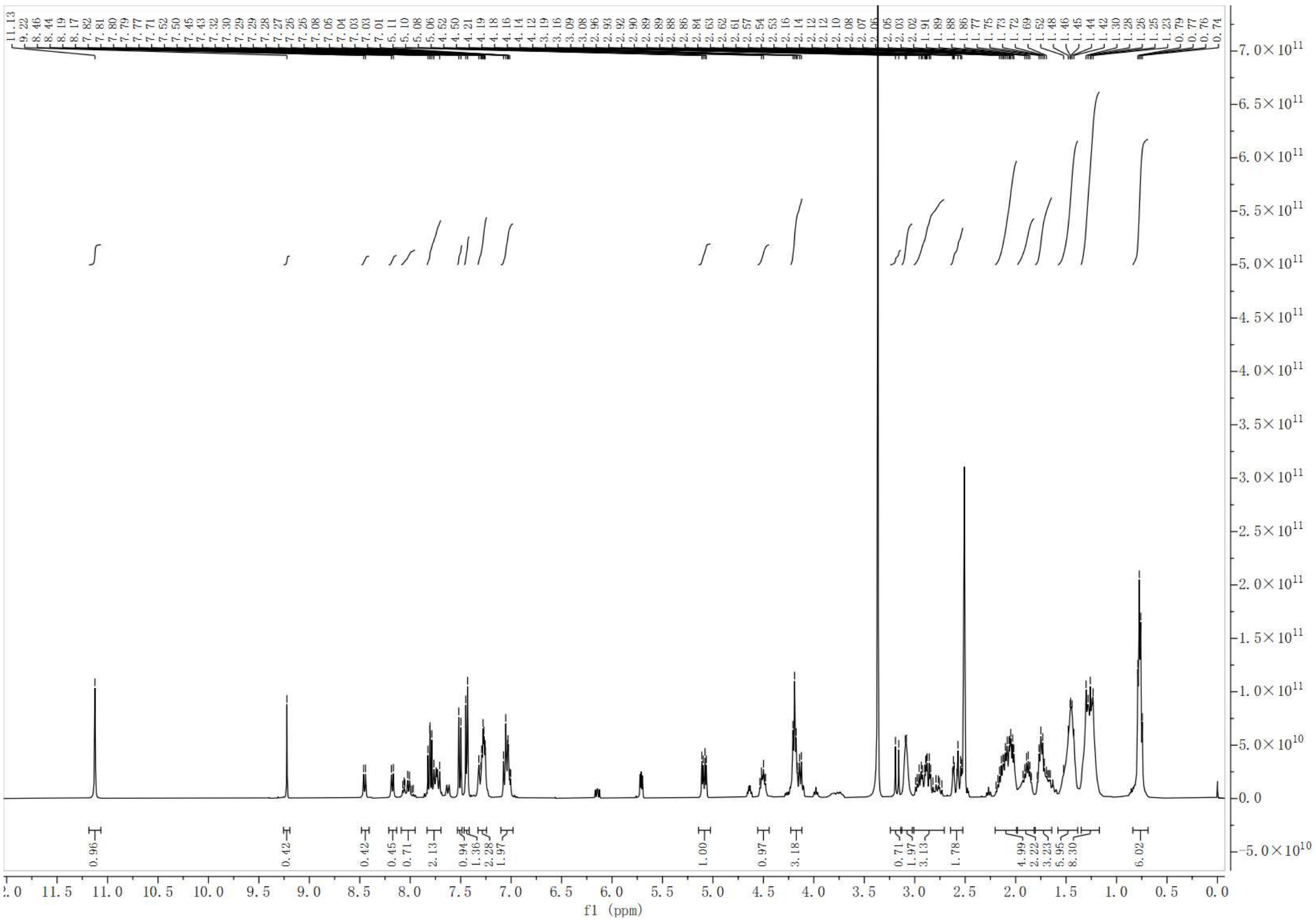

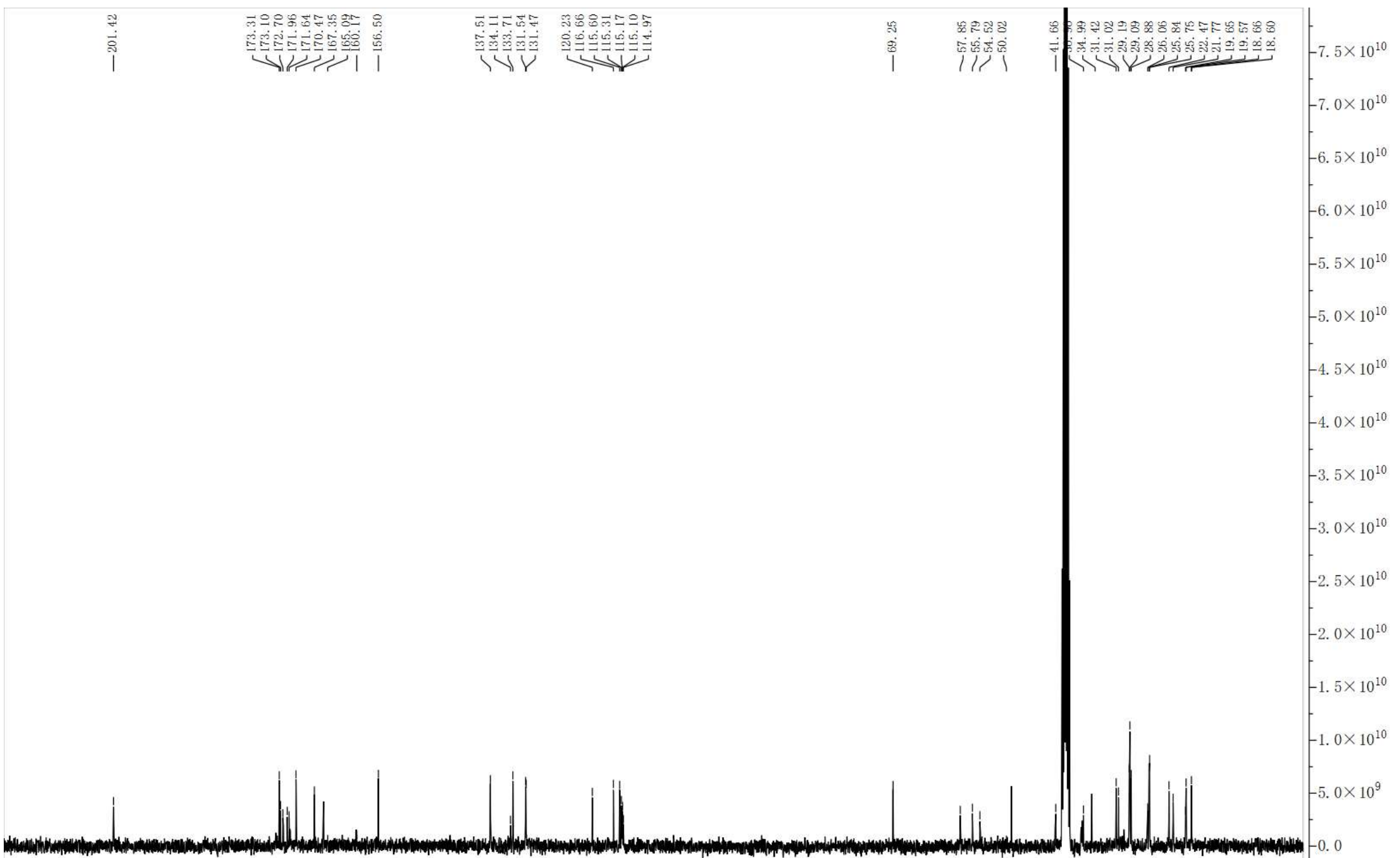

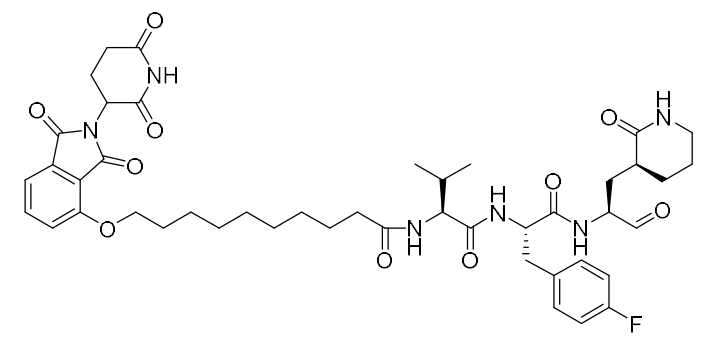

D33

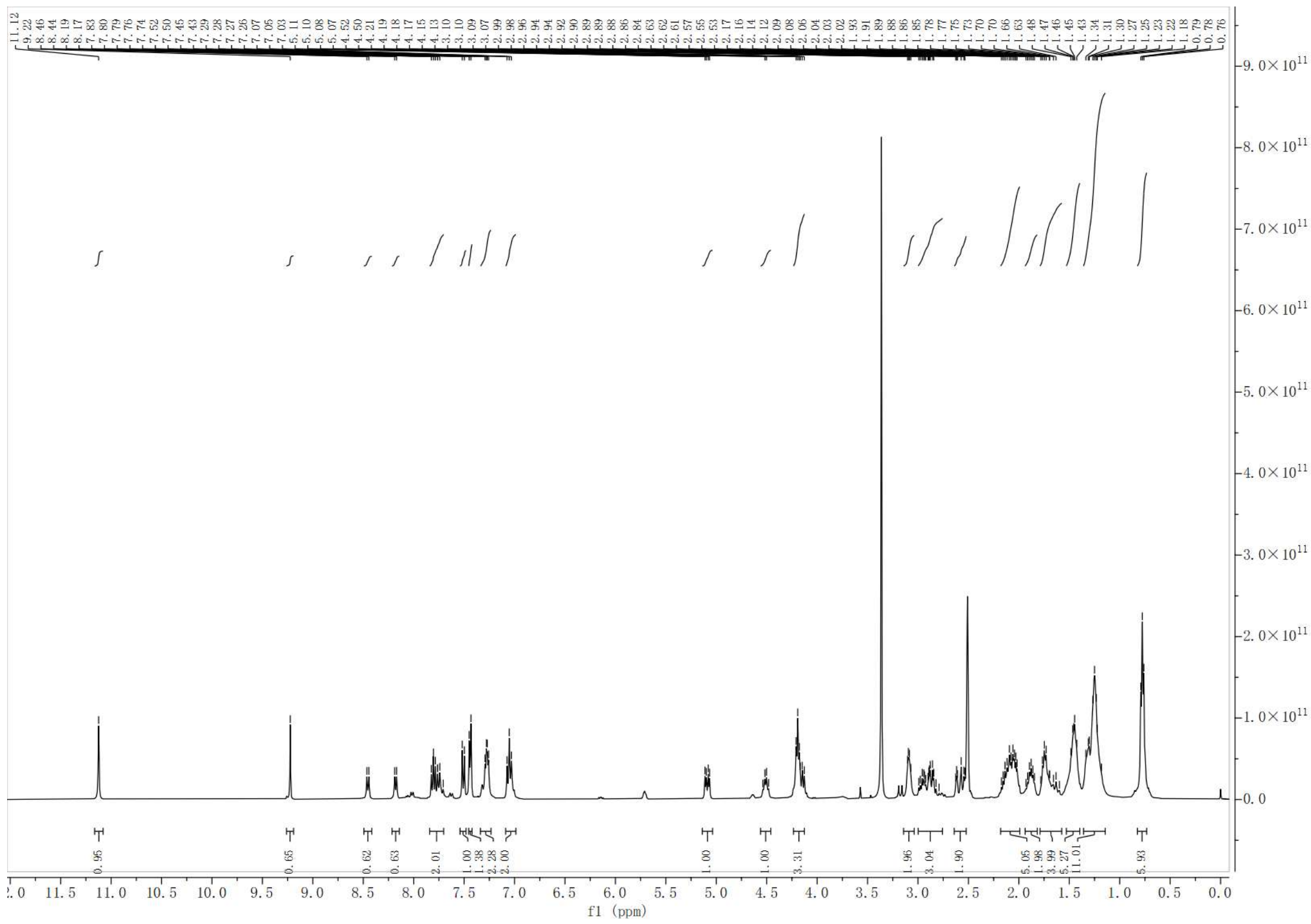

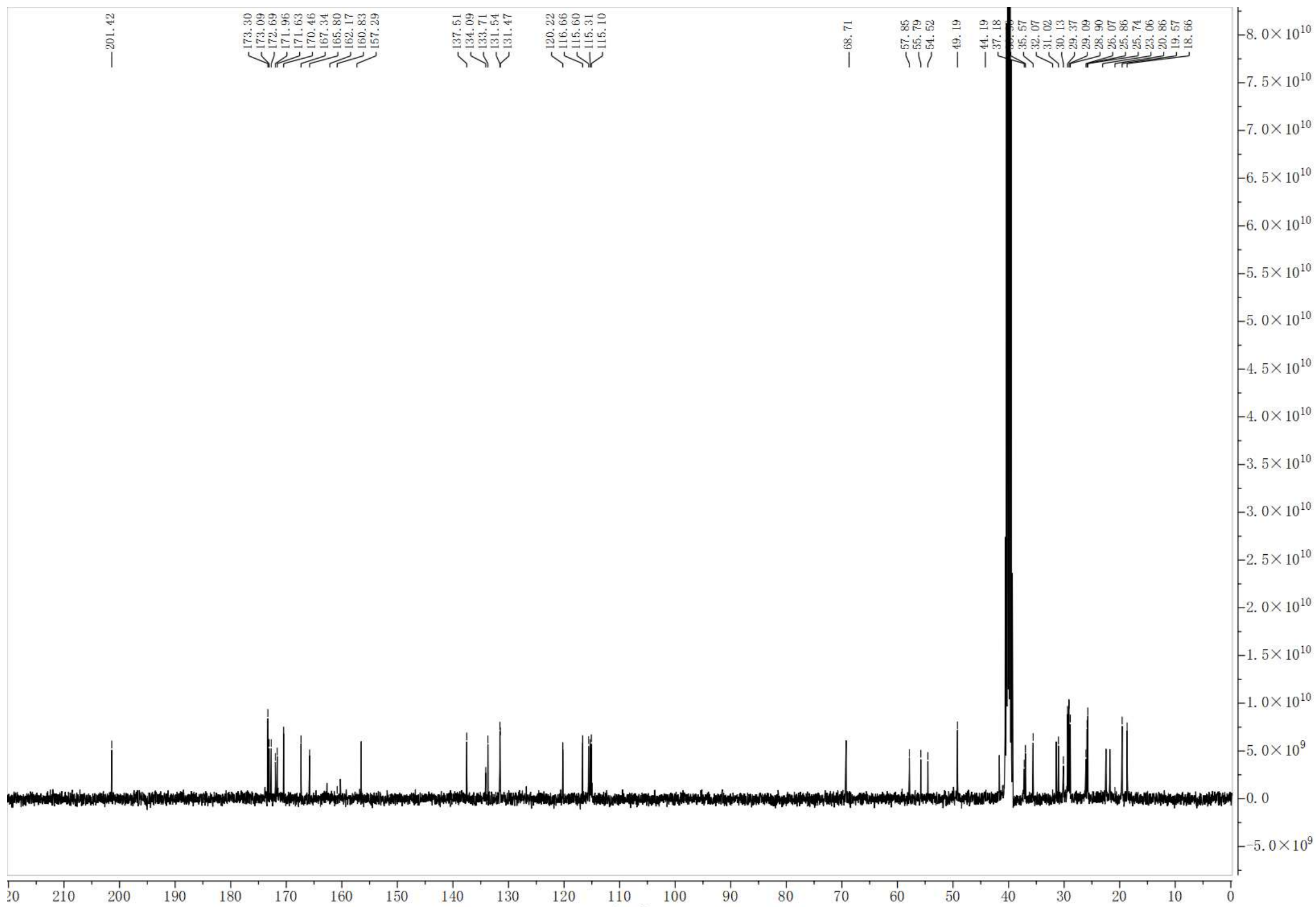

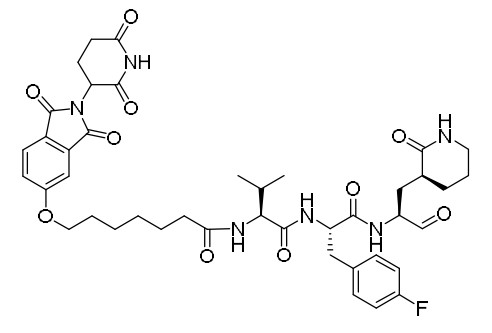

D34

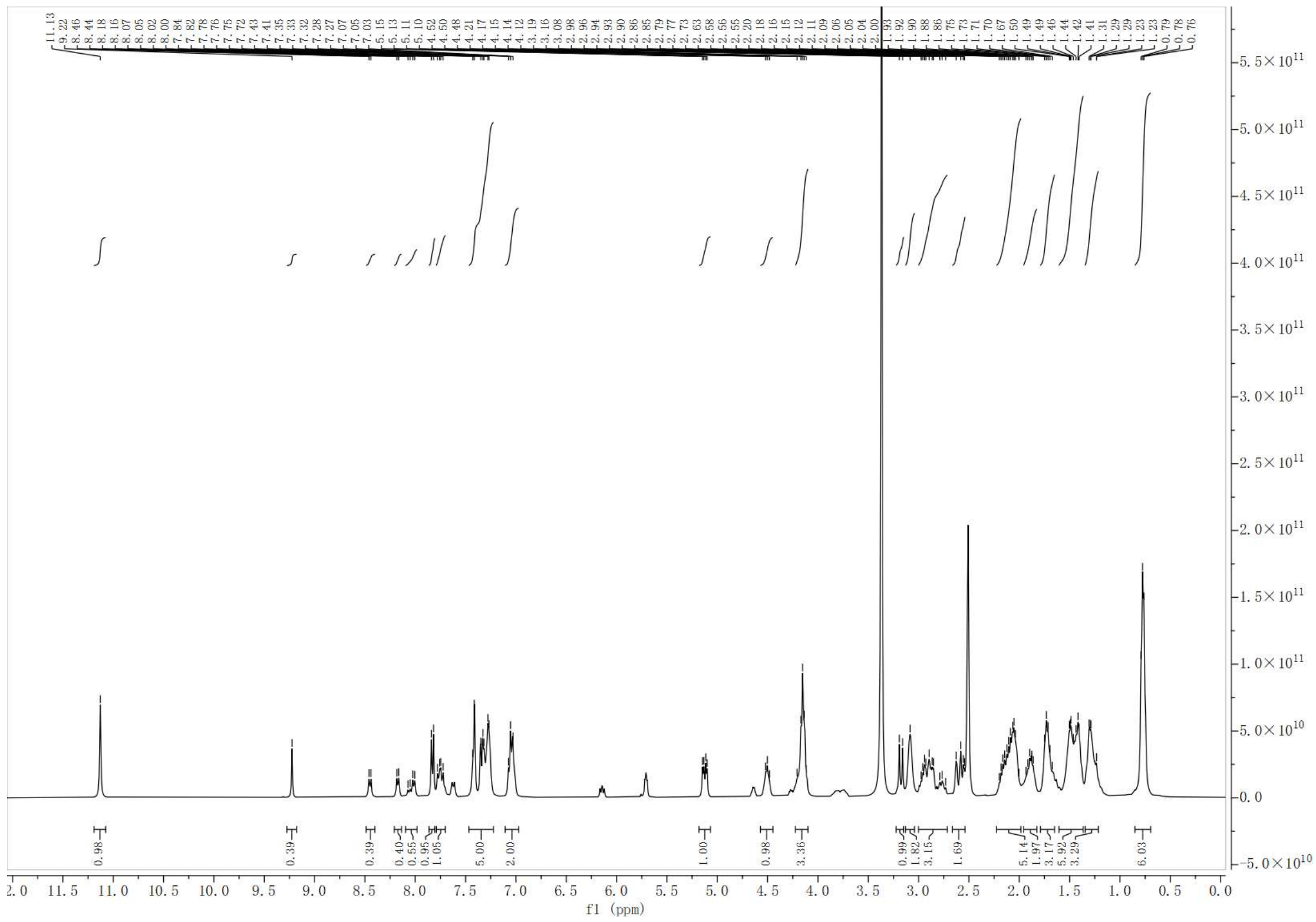

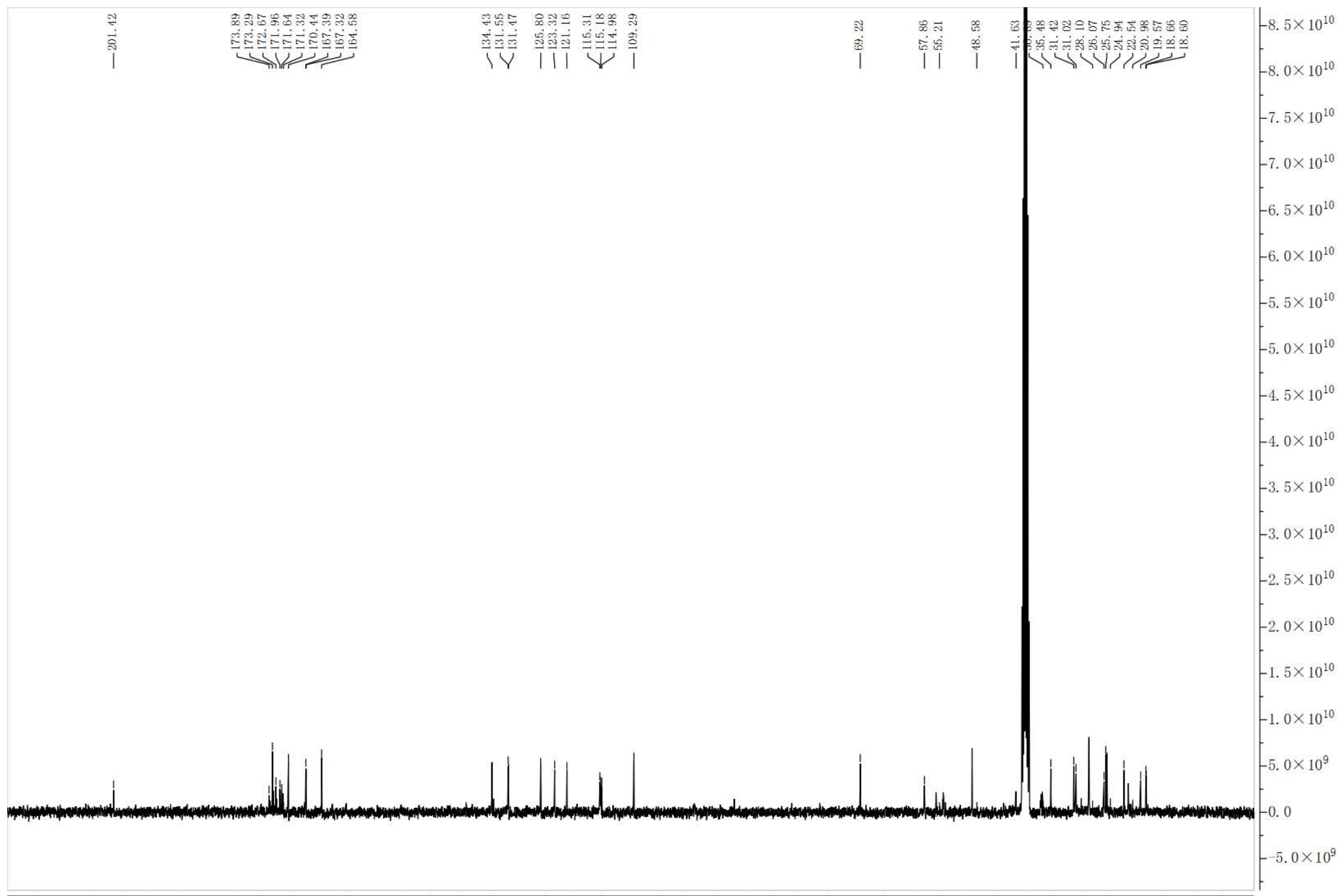

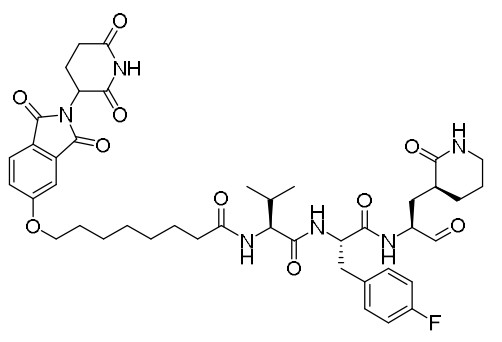

D35

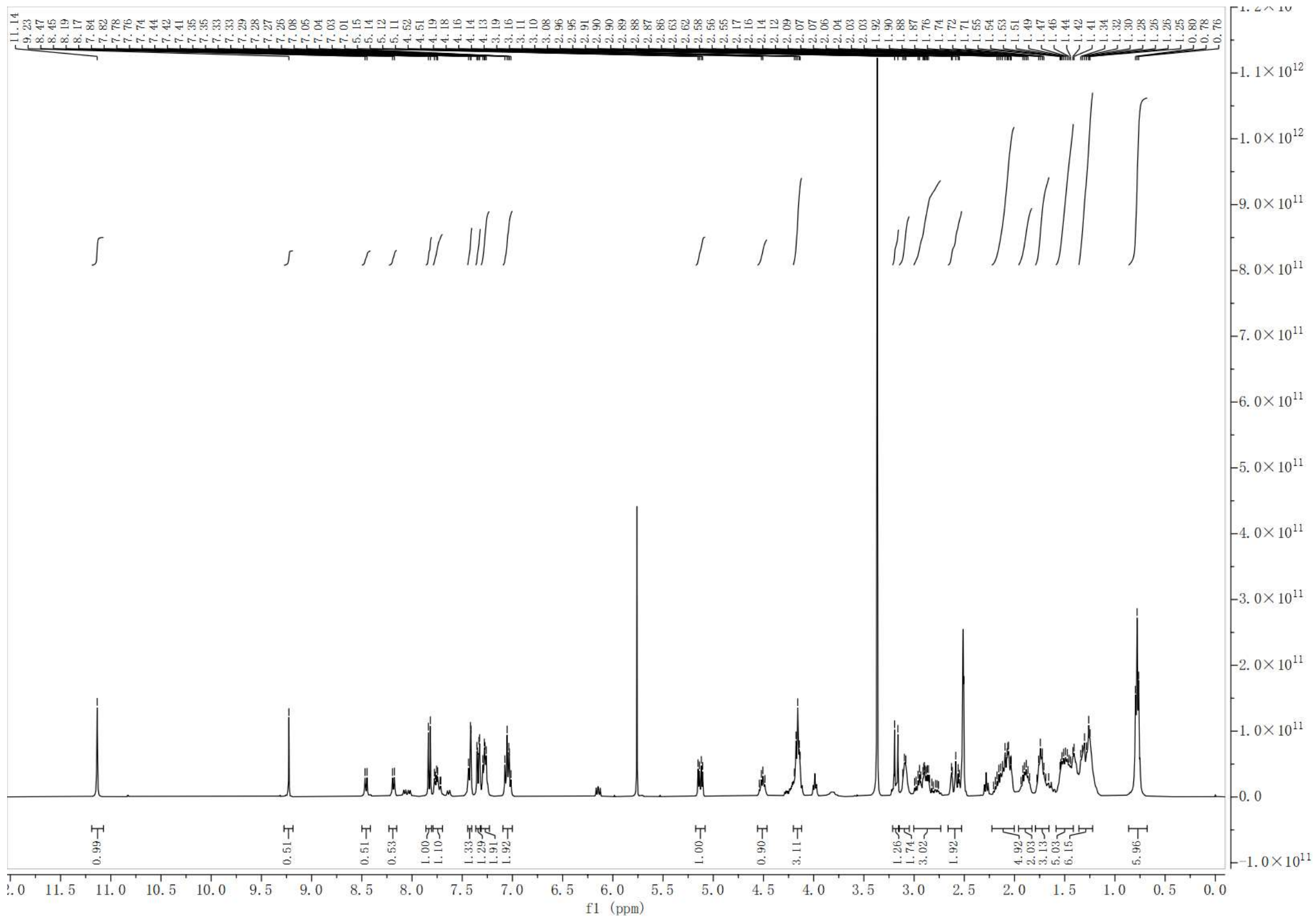

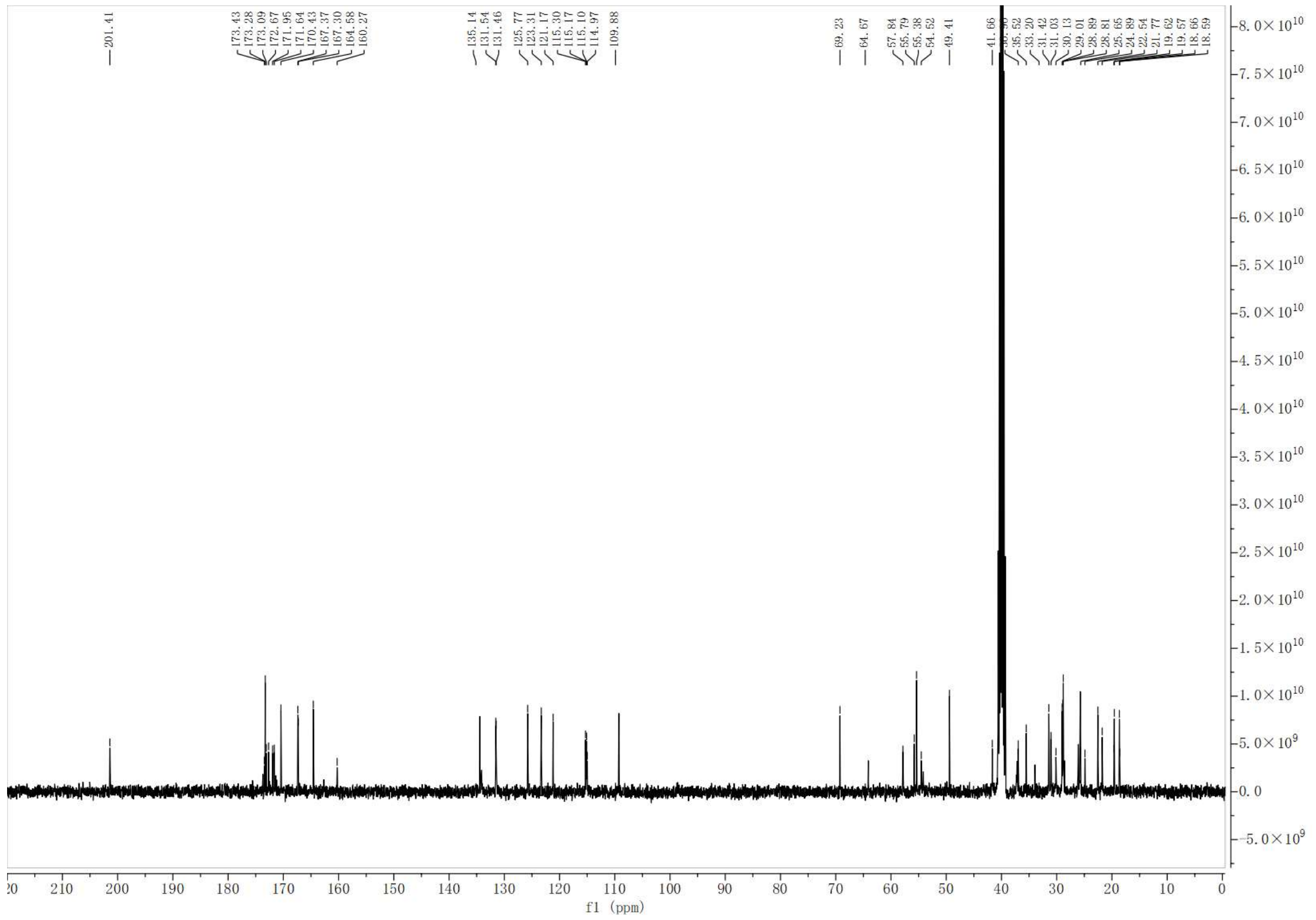

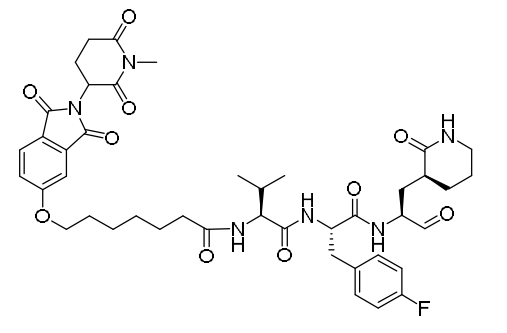

D34-Neg

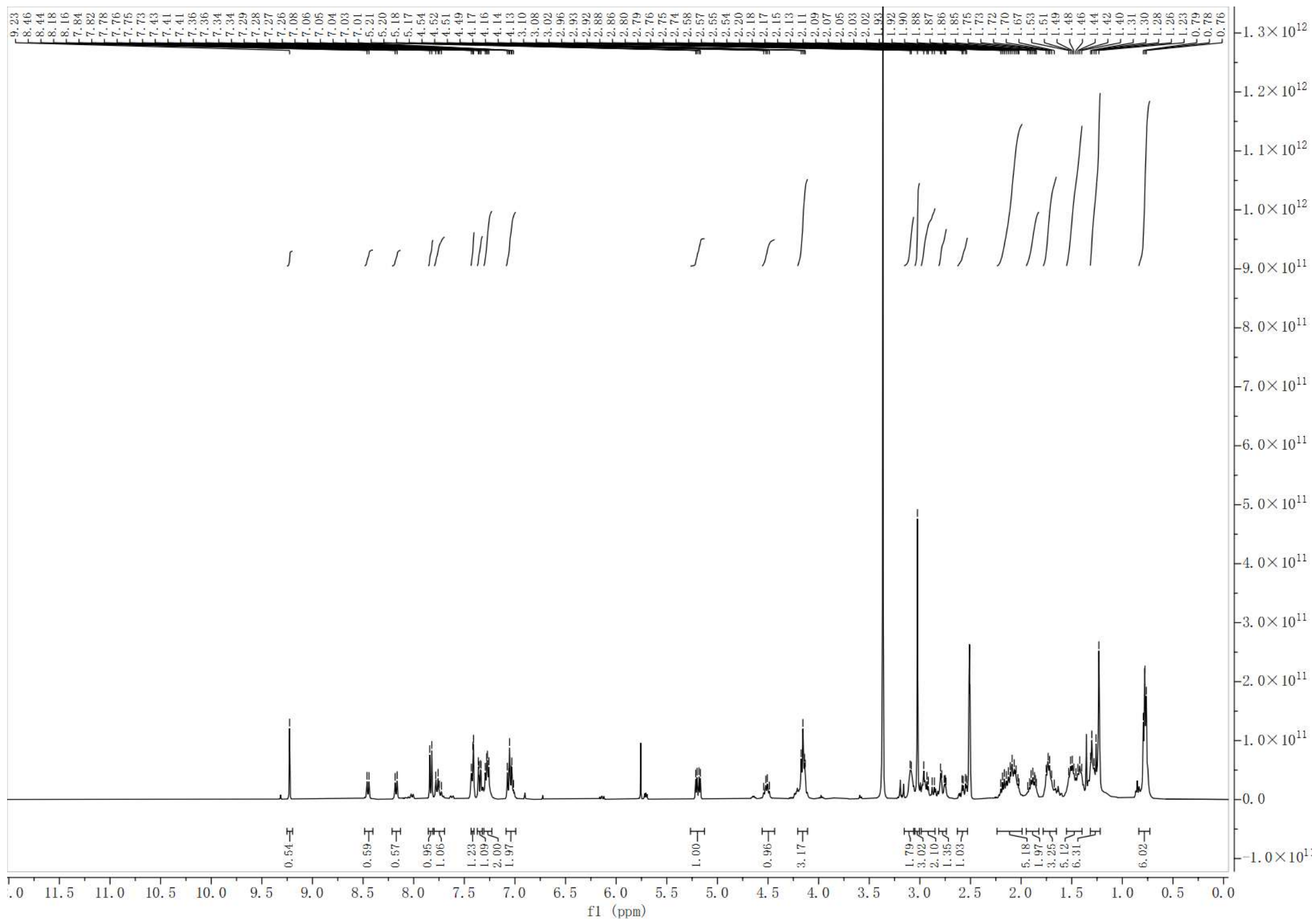

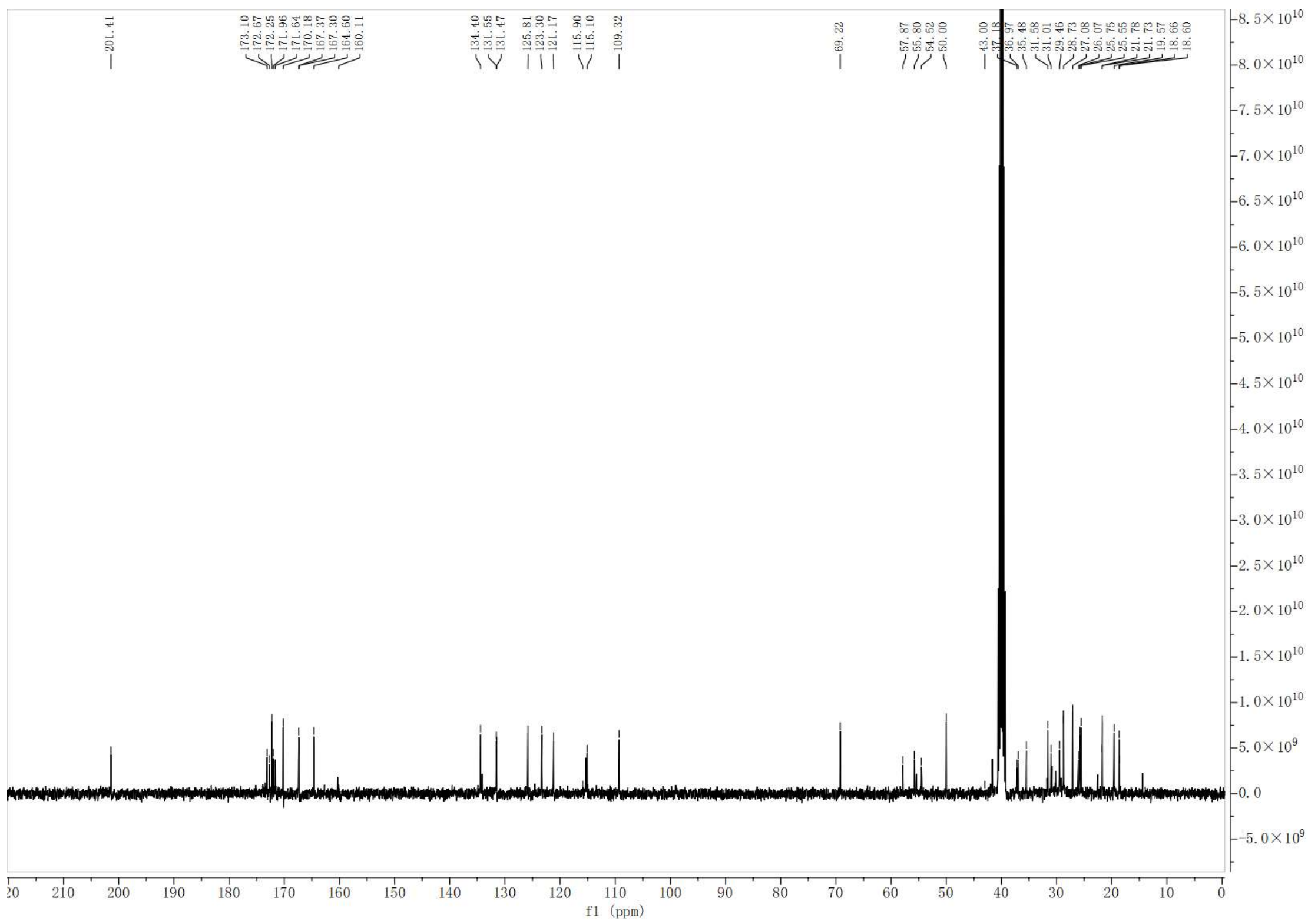

Supplement: Supplementary file 1 [file ADVS-13-e76662-s001.pdf]
